# Deciphering the autophagy regulatory network via single-cell transcriptome analysis reveals a requirement for autophagy homeostasis in spermatogenesis

**DOI:** 10.7150/thno.55645

**Published:** 2021-03-05

**Authors:** Mei Wang, Yanwen Xu, Yuncong Zhang, Yuhan Chen, Gang Chang, Geng An, Xinyan Yang, Caihong Zheng, Jiexiang Zhao, Zhaoting Liu, Dazhuang Wang, Kai Miao, Shuan Rao, Meng Dai, Dong Wang, Xiao-Yang Zhao

**Affiliations:** 1 State Key Laboratory of Organ Failure Research, Department of Developmental Biology, School of Basic Medical Sciences, Southern Medical University, Guangzhou, Guangdong, 510515, P. R. China.; 2 Department of Biochemistry and Molecular Biology, Shenzhen University Health Science Center, Shenzhen, Guangdong, 518060, P. R. China.; 3 Department of Bioinformatics, School of Basic Medical Sciences, Southern Medical University, Guangzhou, Guangdong, 510515, P. R. China.; 4 Department of Health Management, Nanfang Hospital, Southern Medical University, Guangzhou, Guangdong, 510515, P. R. China.; 5 Guangdong Key Laboratory of Construction and Detection in Tissue Engineering, Southern Medical University, Guangzhou, Guangdong, 510515, P. R. China.; 6 Zhuhai Precision Medical Center, Zhuhai People's Hospital (Zhuhai Hospital affiliated with Jinan University), Zhuhai, Guangdong, 519000, P. R. China.; 7 Reproductive Medicine Center of The Third Affiliated Hospital of Guangzhou Medical University, Guangzhou 510150, P. R. China.; 8 Department of Thoracic Surgery, Nanfang Hospital, Southern Medical University, Guangzhou, Guangdong, 510515, P. R. China.; 9 Cancer Center, Faculty of Health Sciences, University of Macau, Macau SAR, China.; 10 Bioland Laboratory (Gangzhou Regenerative Medicine and Health Guangdong Laboratory), Guangzhou, Guangdong, 510005, P. R. China.; 11 Department of Gynecology, Zhujiang Hospital, Southern Medical University, Guangzhou, Guangdong, 510280, P. R. China.; 12 China National Center for Bioinformation Key Laboratory of Genomic and Precision Medicine, Beijing Institute of Genomics, Chinese Academy of Sciences, Beijing 100101, P. R. China.

**Keywords:** spermatogenesis, autophagy, single-cell RNA sequencing, spermatogonial stem cells, meiosis, male infertility.

## Abstract

**Background:** Autophagy has been implicated as a crucial component in spermatogenesis, and autophagy dysfunction can lead to reproductive disorders in animal models, including yeast, *C. elegans* and mice. However, the sophisticated transcriptional networks of autophagic genes throughout human spermatogenesis and their biological significance remain largely uncharacterized.

**Methods:** We profiled the transcriptional signatures of autophagy-related genes during human spermatogenesis by assessing specimens from nine fertile controls (including two normal persons and seven obstructive azoospermia (OA) patients) and one nonobstructive azoospermia (NOA) patient using single-cell RNA sequencing (scRNA-seq) analysis. Dysregulation of autophagy was confirmed in two additional NOA patients by immunofluorescence staining. Gene knockdown was used to identify the role of Cst3 in autophagy during spermatogenesis.

**Results:** Our data uncovered a unique, global stage-specific enrichment of autophagy-related genes. Human-mouse comparison analysis revealed that the stage-specific expression pattern of autophagy-related genes was highly conserved in mammals. More importantly, dysregulation of some clusters of autophagy-related genes was observed in NOA patients, suggesting the association of autophagy with male infertility. Cst3, a human-mouse conserved and autophagy-related gene that is actively expressed in spermatogonia and early spermatocytes, was found to regulate spermatogonial stem cell (SSC) maintenance and subsequent male germ cell development. Knockdown of Cst3 increased autophagic activity in mouse SSCs and subsequently suppressed the transcription of SSC core factors such as Oct4, Id1, and Nanos3, which could be efficiently rescued by manipulating autophagic activity.

**Conclusions:** Our study provides comprehensive insights into the global transcriptional signatures of autophagy-related genes and confirms the importance of autophagy homeostasis in SSC maintenance and normal spermatogenesis, opening new avenues for further dissecting the significance of the autophagy regulatory network in spermatogenesis as well as male infertility.

## Introduction

Spermatogenesis is pivotal for paternal genetic information transmission. In mammals, spermatogenesis can be divided into sequential steps, including mitosis, meiosis and spermiogenesis, all of which are crucial to proper male gamete production. It is known that spermatogenesis disorders can lead to severe male reproductive diseases, such as oligospermia, asthenospermia or azoospermia 1-3. Although some studies have uncovered the transcriptional signatures of human and mouse spermatogenesis via single-cell sequencing 4-12, the detailed regulatory networks involved in spermatogenesis remain to be comprehensively elucidated.

Autophagy is an evolutionarily conserved process by which cellular components can be transported into lysosomes for degradation and recycling, thereby facilitating cellular homeostasis and survival 13. Autophagy has been shown to be involved in many developmental processes, including male reproduction. For instance, the dysregulation of autophagy has been implicated in male infertility in mice; it affects testosterone synthesis in Leydig cells 14, ectoplasmic specialization in Sertoli cells 15, acrosome biogenesis 16-18 and round spermatid-specific chromatoid body integrity 19. Undoubtedly, autophagy plays important roles in germline development, as revealed by studies of gene knockout mouse models. However, the relationship between the autophagy network and spermatogenesis is still unclear. Most importantly, it is unknown whether autophagy dysfunction is involved in human male infertility.

Previously, we generated a dataset of all human autophagy-related genes 20, 21, providing a high-throughput and robust platform for autophagy analysis. Although more than 1000 autophagy-related genes have been discovered, the functions of these genes in spermatogenesis are largely unknown (Tables S1 and S2, Supplemental references 1 and 2). Since the transcriptome of humans and mice during spermatogenesis has been established at single-cell resolution 7, 11, it offers a useful reference for understanding the transcriptional regulatory network of autophagic genes at each step of spermatogenesis.

Here, we analyzed the transcriptome of autophagic genes throughout human and mouse spermatogenesis at single-cell resolution, aiming to provide a global view of how autophagy regulates spermatogenesis. Dysregulated expression of some clusters of autophagy-related genes was observed in testicular germ cells from nonobstructive azoospermia (NOA) patients compared to fertile controls, indicating the relationship between autophagy homeostasis and human male infertility. A unique stage-specific enrichment of autophagy-related genes was uncovered in human spermatogenesis, and this expression pattern was highly conserved between humans and mice, suggesting that the murine model is an ideal system for exploring the role of autophagy in human spermatogenesis, which would facilitate the diagnosis and treatment of male infertility in the clinic. Finally, Cst3 was uncovered to play important roles in the regulation of spermatogonial stem cell (SSC) maintenance and subsequent male germ cell development: Cst3 knockdown increased autophagic activity in mouse SSCs and repressed the transcription of SSC core factors.

## Materials and Methods

### Ethics approval and consent to participate

Every donor signed informed consent and voluntarily donated the testicular tissues for this study. The experiments performed in this study were approved by the Ethics Committee of the Third Affiliated Hospital of Guangzhou Medical University (2017-055). Animal procedures were performed according to the ethical guidelines of the South Medical University Animal Ethics Committee (L2016149).

### Experimental model and subject details

For transcriptional profiling, human testis samples were obtained from two fertile adult males from couples undergoing *in vitro* fertilization due to female causes (named normal persons) and seven males with obstructive azoospermia (OA) undergoing sperm isolation surgery for *in vitro* fertilization due to vas deferens obstruction. Detailed information on these samples was described in our previous study 7. Testicular samples from three NOA male patients (NOA1: 36 years old; NOA2: 50 years old; NOA3: 34 years old) diagnosed with severe oligospermia undergoing sperm isolation surgery for *in vitro* fertilization were also collected for this work. Single-cell RNA sequencing (scRNA-seq) of testicular cells from NOA1 was performed, and hematoxylin and eosin (H&E) and immunofluorescence staining were performed with paraffin testicular sections from NOA1, NOA2 and NOA3.

### Collection of autophagy-related genes

A total of 1,411 human autophagy-related genes 20 were collected from the Autophagy Database, Human Autophagy Database (HADb), Human Autophagy Modulator Database (HAMdb) and The Autophagy, Necrosis, ApopTosisOrchestratorS (THANATOS) database. A total of 709 mouse autophagy-related genes were retrieved from the Autophagy Database and THANATOS. The autophagy-related genes that were experimentally validated in the databases were then selected. Collectively, the autophagy-related gene lists were established via manual filtration based on their reported autophagic roles (Tables S1 and S2, Supplemental references 1 and 2).

### Identification of cell type-specific autophagy-related genes

The single-cell gene expression matrices of human and mouse testicular cells were derived from our previous study 7 and Prof. Minghan Tong's laboratory 11, respectively. The edgeR package (version 3.22.5) was used to identify cell type-specific genes based on the count expression matrix. Genes with log fold change (FC) > 1.5 and false discovery rate (FDR) < 0.01 and expression in at least 60% of cells in each cell type were selected.

### Self-organizing map (SOM) algorithm

1,411 human and 709 mouse autophagy-related genes were first overlapped with scRNA-seq datasets of human and mouse testicular cells from our previous study 7 and Prof. Minghan Tong's lab 11, respectively. A total of 1,362 human and 690 mouse autophagy-related genes expressed in testicular cells were retained for SOM analysis to know the dynamic expression signature of these genes, the remaining 49 human and 19 mouse autophagy-related genes were not detected by scRNA-seq in this study. Prior to SOM training, the expression level of each gene in each stage was recorded as the median value for each stage. The R package kohonen (version 3.0.7) was used to construct the SOM. To yield smooth toroidal boundary conditions, the map grid was reset with the first two principal components of the data multiplied by a sinusoidal function. Then, the visualization map was drawn by R package.

### Differentially expressed autophagic genes and k-means clustering during spermatogenesis

The edgeR package was used to categorize the differentially expressed genes (DEGs) of adjacent stages of human and mouse spermatogenesis. A total of 1,183 human and 486 mouse DEGs with |logFC| > 1 and FDR < 0.05 were retained. K-means clustering was used to categorize the differentially expressed autophagy-related genes between adjacent cell stages in spermatogenesis by using R; the following parameters were applied: number of clusters = 6, maximum number of iterations = 1,000, and number of random cluster center sets = 6. The ggplot2 package (version 3.0.0) was used with the output of the k-means function for visualization.

### Mouse testicular germ cell isolation by FACS

Spermatogonia, primary spermatocytes, secondary spermatocytes and spermatids were isolated from adult Kun Ming (KM) mice by FACS (MoFlo XDP, Beckman) as previously described 22.

### Weighted gene co-expression network analysis (WGCNA)

The R package WGCNA (version 1.66) was used to identify spermatogenesis-related modules for human-mouse homologous autophagy-related genes. Pearson correlations were determined for each pair of autophagy-related genes, and the soft threshold of the correlation matrix was calculated to ensure a good scale-free topology fit and large number of connections. The “blockwiseModules” function was implemented for weighted gene co-expression network construction and module detection in a groupwise manner. With similar expression profiles' minimum module size of 10 genes for the resulting dendrogram, the topological overlap was calculated, and genes were further clustered by average linkage hierarchical clustering. A module color was automatically allocated, and the regions/structures not classified into any module were then bundled as the “gray module”. Module eigengenes (MEs) were regarded as the major principal components for each gene module and were used to represent the gene expression profile in the module. Then, the Pearson correlation coefficients and *P* values between the modules and the spermatogenesis stages were calculated to identify the relevant modules of interest based on MEs. Gene Ontology (GO) analysis and Protein-Protein interaction (PPI) network analysis were performed using online webserver named Metascape 23.

### Conservation analysis of different autophagy-related gene clusters

We downloaded the base-by-base phastCons scores across 4 vertebrates (mouse, malayan flying lemur, human, and Chinese tree shrew) from the University of California Santa Cruz (UCSC) Genome Browser. We calculated the conservation of each gene using the following formula: the sum of each gene's exon phastCons scores divided by the length of each gene. For autophagy-related genes comparison between human and mouse, 1,411 human and 709 mouse autophagic genes were overlapped with 17,771 human-mouse 1 to 1 homologous genes, 531 genes were the homologous autophagic genes between human and mouse, 831 autophagic genes were the homologous autophagic genes only described in human-related studies (named as 831 human genes), 156 autophagic genes were the homologous autophagic genes only described in mouse-related studies (named as 156 mouse genes), 49 human genes and 22 mouse genes were not included in the 1 to 1 homologous autophagic genes. Based on human-mouse 1 to 1 homologous genes, 831 human and 156 mouse homologous autophagy-related genes were recognized as autophagic genes in mouse (831 mouse genes) and human (156 human genes), respectively. Genes that were not detected by scRNA-seq were excluded for further analyses. In addition, these genes were overlapped with all clusters of DEGs to obtain 167 (mouse) and 37 (human) DEGs with logFC > 1.5 and FDR < 0.01 that had not been characterized in previous mouse and human autophagy-related studies, respectively. We selected the remaining genes as widely expressed genes based on the criteria of expression in at least 1/5 of the samples and transcripts per million (TPM) values of more than 1. The remaining genes were expressed at low levels.

### Histological examination

Testicular tissues from donors with OA or NOA were fixed in Carnoy's fixative for 4-6 h at room temperature, embedded in paraffin and sectioned for subsequent use. Before staining, tissue sections were dewaxed and rehydrated. Then, the sections were stained with H&E. The staining results were captured using an OLYMPUS BX51 microscope.

### Isolation and capture of NOA testicular cells for single-cell transcriptional profiling

Testicular samples from NOA patients were minced with sterilized scissors after washing three times with phosphate-buffered saline (PBS) and then digested in 1 mg/ml collagenase type IV at 37°C for 15 min. The digestion process was stopped by adding Dulbecco's modification of Eagle's medium (DMEM) containing 10% fetal bovine serum (FBS). The cell suspension was filtered through 40-μm nylon mesh. After centrifugation, the cells were resuspended in DMEM containing 10% FBS for further single-cell collection**.**

### scRNA-seq library preparation and sequencing

The scRNA-seq library was prepared using the single-cell tagged reverse transcription sequencing (STRT-seq) protocol 24. scRNA-seq library sequencing was performed on the Illumina HiSeq XTEN platform.

### scRNA-seq data processing

scRNA-seq data analysis was performed as previously described 7. First, specific cell barcodes (8 bp) in read 2 were used to separate single-cell R2 reads, and read IDs were used to extract the corresponding R1 reads. Then, template switch oligo (TSO) sequences, polyA tails and low-quality bases were removed to obtain clean reads. The clean R1 reads were mapped to the human reference genome GRCh38 (downloaded from the ENSEMBL database) with STAR (v2.7.1a) 25. featureCounts (v1.6.4) 26 was used to count uniquely mapped reads, and cell-specific barcodes were used to group the uniquely mapped reads. The duplicated reads were removed based on the unique molecular identifier (UMI) information. Finally, distinct UMIs for each gene were counted as the transcript copy number and normalized to the TPM value. Gene expression levels were normalized by the log_2_(TPM/10+1) method as described previously 7.

We sequenced 480 cells from the NOA patient, and we retained the cells with more than 2,000 genes and 10,000 transcripts. In total, 432 cells from patient NOA1 were used for subsequent analysis.

### Cell type projection by random forest classifier

To project the cell types of the NOA patient, we adopted the random forest algorithm, a machine learning classifier based on the decision trees learned from a training dataset. First, we used 2,854 human testicular cells derived from nine fertile controls (including two normal persons and seven OA patients) from our previous study 7, which had definite cell type information. Then, 1,483 informative features were selected based on the specific expressed DEGs in the corresponding cell types. Finally, we obtained an expression matrix with 2,854 cells × 1,483 genes to train the decision trees in the random forest model (2,000 trees). The trained classifier was tested by using a subset of the training data with good accuracy. Then, it was used to predict the cell types of the NOA patient. The analysis was performed by the randomForest R package version 4.6-14 in R studio.

### Dimensionality reduction of scRNA-seq data

Dimensionality reduction of normal and NOA scRNA-seq data was performed using the Seurat R package (version 3.1.2) 27. We integrated scRNA-seq data from the NOA patient (432 single cells) in this study and previously published fertile donors (2,854 single cells) 7 using the FindIntergrationAnchors (dims = 1:13) and IntegrateData (dims = 1:13) functions. Two thousand highly variable genes in the integration analysis were used to perform the RunPCA function. We selected principal components (PCs) 1-13 to perform the RunUMAP function and obtained the uniform manifold approximation and projection (UMAP) diagram.

### Identification of DEGs between the testicular cells of fertile persons and NOA patient

We used the Seurat FindMarkers function (test.use = wilcox, min.pct = 0.1) based on normalized TPM expression values (log_2_(TPM/10+1)) to identify the DEGs of each cell cluster between testicular cells from fertile and NOA patients. Genes with |avg_logFC| > 1 and p_val_adj < 0.05 were defined as DEGs in Figure 2 and Figure S2.

### Immunostaining of mouse SSCs and paraffin sections of testes

Immunostaining was performed as described previously 7, 28. Cells and testicular paraffin sections were blocked with 2% bovine serum albumin (BSA) for 1 h and then incubated with primary antibodies overnight at 4°C. The following primary antibodies were used: mouse anti-MAP1LC3A (also named as LC3A, Abcam, ab168803), rabbit anti-KIT to human (Abcam, ab32363), rabbit anti-Kit to mouse (Cell Signaling Technology, 3074), mouse anti-DMC1 (Abcam, ab11054), rabbit anti-SYCP3 (Abcam, ab15093), chicken anti-GFP (Abcam, ab13970), rabbit anti-UTF1 (Abcam, ab105090), rabbit anti-CST3 (Abcam, ab109508), mouse anti-HSPD1 (Abcam, ab13532), mouse anti-γH2AX (Abcam, ab26350), rabbit anti-DRAM1 (Abcam, ab188648), rabbit anti-DDX4 (Abcam, ab13840), mouse anti-DDX4 (Abcam, ab27591), rabbit anti-SQSTM1 (Abcam, ab91526), mouse anti-FGFR3 (Santa Cruz, sc-13121), mouse anti-Hspd1 (Santa Cruz, sc-13115), mouse anti-Zbtb16 (Santa Cruz, sc-28319), and goat anti-Gpr125 (Novus Biological, NBP1-03561). Subsequently, the samples were washed for three times with PBS, followed by incubation with secondary antibodies (Jackson ImmunoResearch, Alexa 488-, Alexa 594-, Alexa 647-labeled IgG or FITC) or/and peanut agglutinin (PNA, Thermo Fisher, L32460 and L21409) for 1 h at room temperature. The nuclei were counterstained with 10 μg/ml Hoechst 33342 for 15 min at room temperature, followed by washing with PBS. Images were captured with a ZEISS LSM880 confocal microscope.

### Validation of candidate autophagy-related genes in other human and mouse testicular datasets at single-cell resolution

Human and mouse testicular scRNA-seq datasets, including GSE120508 (6,490 testicular cells from three adult males) and E-MTAB-6946 (53,510 testicular cells from juvenile (postnatal days 5-35) and adult (8-9 weeks) C57BL/6J mice), were used to validate the transcriptional dynamics of the candidate autophagy-related genes 6, 9. The R package Seurat (v.3.1.0) was applied for the analysis of GSE120508 and E-MTAB-6946. To diminish these batch effects, the FindIntegrationAnchors and IntegrateData functions were performed to effectively integrate these two datasets. The RunUMAP function was applied to perform dimensional reduction using read counts. The expression patterns of selected candidate autophagy-related genes were projected on the UMAP plot.

### Mouse spermatogonial stem cell (mSSC) culture and derivation of Cst3 knockdown mSSCs

mSSCs were derived from 5.5 days postpartum (dpp) B6D2F1 male mice. GFP^+^ SSCs were derived from 5.5 dpp male mice generated by mating female EGFP-C57BL/6 mice with male DBA/2 mice. mSSC culture was performed according to a previous study, with culture supplemented with 20 ng/ml mouse epidermal growth factor (EGF) (Thermo Fisher), 10 ng/ml human basic fibroblast growth factor (bFGF) (R&D), 10 ng/ml rat glial cell line-derived neurotrophic factor (R&D) and 10^3^ U/ml ESGRO (Millipore) 29.

To establish stable *Cst3* knockdown mSSC lines, small hairpin RNAs (shRNAs) against *Cst3* were constructed using the pLKO.1-puro shRNA system (Sigma). The sequences were as follows: negative control shRNA (shNC): 5'-CAACAAGATGAAGAGCACCAACTCGAGT-TGGTGCTCTTCATCTTGTTGTTTT-3'; *Cst3*-KD shRNA #1, 5'-CCGGCGTGGCTGGAG-TGAACTATTTCTCGAGAAATAGTTCACTCCAGCCACGTTTTTG-3'; *Cst3*-KD shRNA #2, 5'-CCGGCCATCTGATGAGGAAGGCACTCTCGAGAGTGCCTTCCTCATCAGATG-GTTTTTG-3'. For lentivirus packaging, plasmids were transiently transfected into HEK293T cells. Viral supernatant containing a final concentration of 4 μg/ml polybrene (Sigma) was used to infect mSSCs. Positively transduced cells were selected using puromycin (Thermo Fisher) at a final concentration of 0.4 μg/ml.

### Quantitative PCR (q-PCR)

mSSCs and GFP^+^ cells sorted by flow cytometry were lysed in TRIzol reagent (Invitrogen), and total RNA was isolated using chloroform extraction and treated with DNase. cDNA was synthesized using HiScript®ⅡReverse Transcriptase (Vazyme, R233-01). q-PCR was performed using 2× RealStar Green Fast Mixture (GenStar, A301-01) on a LightCycler® Real-Time PCR System (Roche). The data were analyzed using the delta-delta Ct method. *Gapdh* was used as an internal control to normalize the expression of target genes. q-PCR was performed with gene-specific primers (Table S11).

### Autophagy vesicle scan by transmission electron microscopy (TEM)

Approximately 10^6^ cells were fixed in 2.5% glutaraldehyde in 0.1 M PBS buffer at 4°C overnight and then postfixed with 1% osmium tetroxide at room temperature for 2 h. After dehydration in a graded series of acetone, the cells were embedded in Epon 812 resin. Ultrathin (60 nm) sections were collected on 200 mesh copper grids, stained with 2% uranyl acetate in 50% methanol for 10 min, followed by 1% lead citrate for 7 min. Subsequently, the sections were stained with uranyl acetate and lead citrate and finally examined with a Hitachi-H7500 transmission electron microscope.

### Chloroquine (CQ) and 3-methyladenine (3-MA) treatment of mSSCs

mSSCs were treated with dimethylsulfoxide (DMSO)-dissolved CQ (TargetMol, T8689) at final concentrations of 5, 10, and 20 μM at 37°C, and DMSO was used as the control. mSSCs were treated with ddH_2_O-dissolved 3-MA (Merck Millipore, 189490) at final concentrations of 0.5, 1, 2 and 4 mM at 37°C, and ddH_2_O was used as the control. After inhibitor treatment, mSSCs without feeder cells were collected for q-PCR or western blotting assays.

### Western blotting analysis

Western blotting analysis was performed as previously described 28. Cell extracts were prepared using cold RIPA buffer (25 mM Tris-HCl, pH 7.6, 150 mM NaCl, 1% NP-40, 1% sodium deoxycholate, 0.1% sodium dodecyl sulfate) supplemented with 1 mM phenylmethylsulfonyl fluoride and protein inhibitor cocktail (Roche) for 30 min. The homogenates were centrifuged at 12,000 rpm for 15 min, and the supernatants were maintained for further use. The protein concentrations of the samples were determined using the Pierce® BCA Protein Assay Kit (Thermo, NCI3225CH). Protein lysates were separated by SDS-PAGE and transferred to nitrocellulose membranes. Specific proteins were analyzed using rabbit anti-Tnp1 (Abcam, ab73135), rabbit anti-CST3 (Abcam, ab109508), rabbit anti-Lc3a/b (Cell Signaling Technology, 12741S), rabbit anti-SQSTM1 (Abcam, ab91526), rabbit anti-ATG5 (Abcam, ab108327), mouse anti-Zbtb16 (Santa Cruz, sc-28319), rabbit anti-DDX4 (Abcam, ab13840), rabbit anti-SYCP3 (Abcam, ab15093), mouse anti-Oct4 (Santa Cruz, sc-5279), rabbit anti-Nanos3 (Abcam, ab70001) and rabbit anti-actin (Proteintech, 20536-1-AP) antibodies. Enhanced chemiluminescence peroxidase-labeled anti-mouse or rabbit antibodies (ZSGB-BIO) were used for further detection. The intense analysis was performed by ImageJ (National Institutes of Health).

### Cell death analysis of mSSCs

mSSCs were collected, washed, and resuspended in PBS. The cell death rate was examined with annexin V-fluorescin isothiocyanate (FITC) and propidium iodide (PI) double staining using an annexin V-FITC detection kit (YEASEN, 40302ES60) according to the manufacturer's instructions. Flow cytometry and data analysis were performed with CytoFLEX (Beckman).

### Proliferation curve of mSSCs

For the Cell Counting Kit-8 (CCK-8) assay, the cells were first plated at 5,000 cells/per well in 96-well dishes. After culture for 24, 48, 72, 96, 120, 144, and 168 h, CCK-8 solution (Beyotime, C0038) was added and incubated for 2 h at 37°C. The absorbance at 450 nm was detected using a microplate reader (Tecan Infinite® M200).

### Cell cycle analysis of mSSCs

Cultured mSSCs were collected and fixed with 70% alcohol in a refrigerator at 4°C for 24 h. After PI staining, the DNA content of cells was measured by flow cytometry. Finally, the percentage of cells in each phase of the cell cycle was analyzed with Beckman CytoFLEX.

### Animals and testicular transplantation of mSSCs

KM mice were purchased from the Laboratory Animal Center of South Medical University. To obtain the busulfan treated neonatal male mice, busulfan was injected into 12.5-day post coitum (dpc) pregnant mice to destroy fetal germ cells of the unborn fetus at a dosage of 50 mg/kg. Nearly 1 × 10^5^ mSSCs were injected into each testis of 6- to 12-day-old male mice treated with busulfan as described previously 30. The shNC-, sh*Cst3* #1- and sh*Cst3* #2 transfected mSSC were injected into four, one, and three male mice, respectively, who were from five busulfan-treated pregnant mice. Ten weeks later, the transplanted mice were sacrificed, and the seminiferous tubules were isolated for immunostaining or flow cytometry analysis of the proportion of GFP^+^ cells and DNA content as described previously 28.

### Statistical analysis

GraphPad Prism 5 (GraphPad Software, Inc., La Jolla, CA, USA) was used to analyze the data of q-PCR, western blotting, immunostaining, TEM and FACS. Statistical differences were evaluated using Student's t test. The standard deviation (SD) was used to assess the biological significance.

## Results

### Global transcriptional signatures of autophagic genes in spermatogenesis

**Figure 1:**
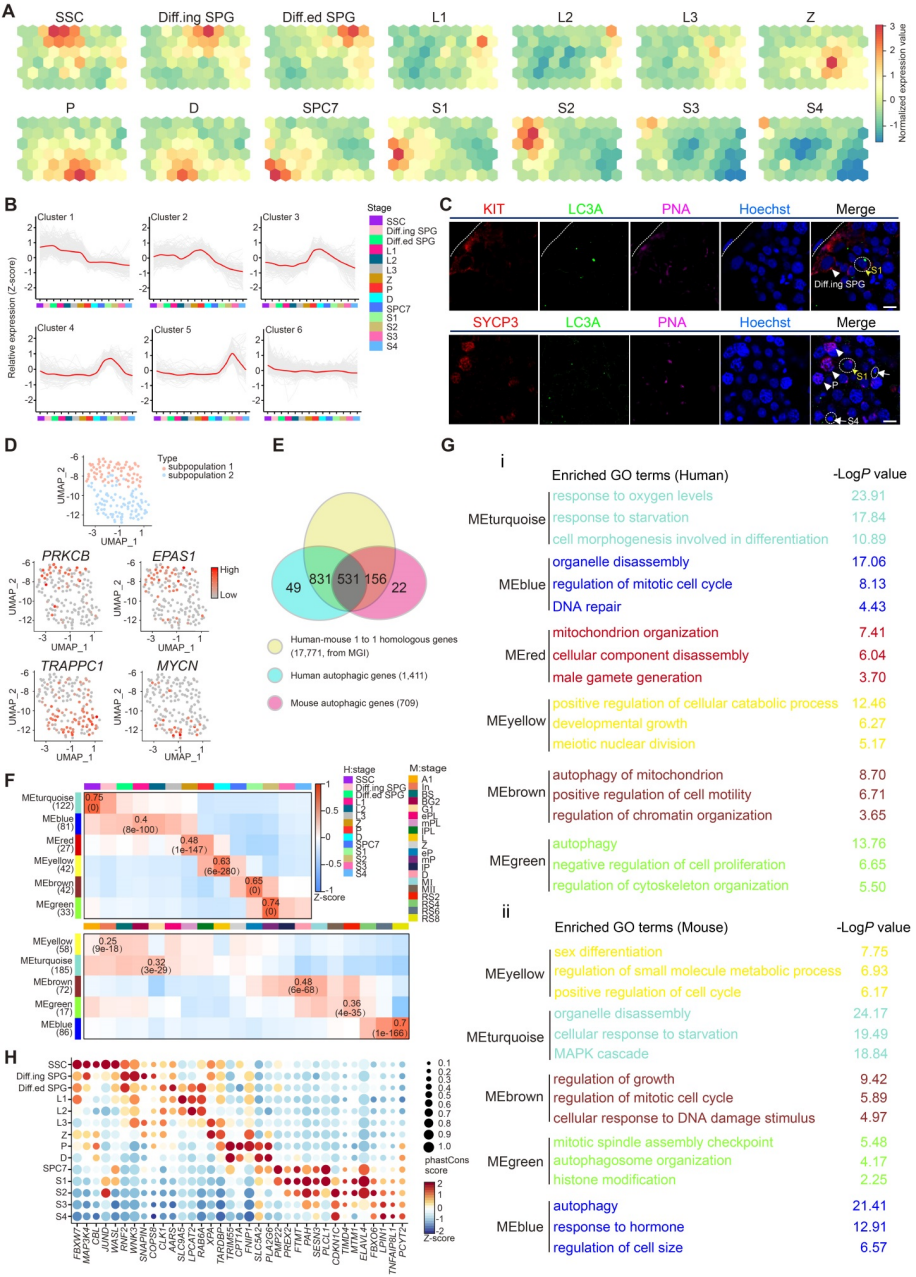
** Global transcriptional signatures of autophagic genes in human spermatogenesis. (A)** Temporal expression of autophagic genes represented by the SOM algorithm; a distinct set of autophagy-related genes are enriched in each cell cluster. A gradient of green to red indicates low to high normalized expression values. Spermatogonial stem cell (SSC), differentiating spermatogonia (Diff.ing SPG), differentiated spermatogonia (Diff.ed SPG), leptotene 1 (L1), leptotene 2 (L2), leptotene 3 (L3), zygotene (Z), pachytene (P), diplotene (D), spermatocyte 7 (SPC7), spermatid 1 (S1), spermatid 2 (S2), spermatid 3 (S3), spermatid 4 (S4). **(B)** K-means clustering of differentially expressed autophagic genes throughout human spermatogenesis into six individual clusters. **(C)** Immunofluorescence of KIT (red, top) and SYCP3 (red, bottom) co-stained with LC3A (green) and PNA (pink) in adult human testicular paraffin sections from one OA donor as a control. Differentiating spermatogonia (Diff.ing SPG), pachytene (P), spermatid 1 (S1), spermatid 4 (S4). The scale bars represent 10 μm. **(D)** UMAP showing two subpopulations of human SSCs (top). Gene expression patterns of specific autophagic genes on UMAP plots. *PRKCB* and *EPAS1* were enriched in subpopulation 1, and *TRAPPC1* and *MYCN* were enriched in subpopulation 2 (middle and bottom, respectively). A gradient of gray to red indicates low to high expression levels. **(E)** Venn diagram showing the distribution and relationship between human-mouse homologous genes (17,771) and human (1,411)- and mouse (709)- autophagic genes. **(F)** WGCNA map showing modules derived from 531 homologous autophagic genes enriched in each human (top)- and mouse (bottom)- spermatogenic cell cluster. Pearson correlation coefficients (top) and *P* values (bottom) to calculate the correlations between gene modules and cell clusters were highlighted in some cells. **(G)** Enriched GO terms and *P* values of modules in Figure 1F are indicated by distinct colors. **(H)** Bubble plot showing the top five autophagic genes derived from 37 DEGs of 14 human spermatogenic cell clusters in Figure S1G. The size of each circle represents each gene's phastCons score. A gradient of blue to red indicates low to high expression levels.

To unbiasedly explore the global transcriptional profiles of autophagic genes in both human and mouse spermatogenesis, testicular scRNA-seq datasets established by the same library construction platform were selected for analyses 7, 11. SOM, an unsupervised learning algorithm for data clustering, dimensionality reduction and image analysis, was performed to know the dynamic transcriptional signature of autophagy-related genes by classifying the global autophagy-related genes expressed in human and mouse testis into distinct gene sets as represented in different cell clusters. Dynamic expression patterns of autophagy-related genes were observed in spermatogonia, late stage of primary spermatocytes and early stage of spermatids (Figures 1A and S1A). Furthermore, k-means was used to categorize the differentially expressed autophagy-related genes between adjacent cell stages for further dissecting the expression signatures of autophagic genes with statistical significance (Figures 1B and S1B). Autophagic flux was evaluated by the expression of classical autophagy markers, wherein the immunostaining of LC3A puncta (Abcam, ab168803) and western blotting of Lc3a/b-Ⅰ and Lc3a/b-Ⅱ (Cell Signaling Technology, 12741S) and Sqstm1 were performed. Our results showed relative enrichment of autophagy activity (intense LC3A puncta of immunostaining, high Lc3a/b-Ⅰ and Lc3a/b-Ⅱ as well as low Sqstm1 signals of western blotting) in spermatids (PNA^+^) compared to spermatogonia (KIT^+^) and spermatocytes (SYCP3^+^) (Figures 1C, S1C, and S1D). Taken together, the temporal and cell type-specific expression patterns of autophagy-related genes as well as autophagic flux in both human and mouse spermatogenesis suggested that each cluster of autophagy-related genes may exert distinct roles in different stages of spermatogenesis.

Previously, we defined the heterogenicity of human spermatogonial stem cells (hSSCs) using scRNA-seq 7, and specific markers such as *UTF1* and *GFRA1* can be used to distinguish different subpopulations of hSSCs (subpopulation 1: SSC1 and subpopulation 2: SSC2). Interestingly, we observed a distinct expression patterns of autophagic genes in each SSC subpopulation (Figures 1D and S1E, Table S3). Specifically, both *PRKCB*, a modulator of mitochondrial energy homeostasis and autophagy 31, and *EPAS1*, a driver of autophagy 32, were identified in the *UTF1*^high^*GFRA1*^low^ SSC subpopulation, whereas two other important autophagy regulators, *TRAPPC1* 33 and *MYCN* 34, were exclusively expressed in the *UTF1*^low^*GFRA1*^high^ SSC subpopulation (Figures 1D and S1E).

### Human-mouse comparison of autophagy in spermatogenesis

Since the global transcriptional profiles of autophagy-related genes during spermatogenesis exhibit similar patterns between humans and mice, we then explored the evolutionary conservation of autophagy in spermatogenesis. To systematically analyze autophagy in the context of spermatogenesis, 17,771 one-to-one homologous genes with phenotype annotations between humans and mice were extracted from the Mouse Genome Informatics (MGI) database. A total of 1,411 human and 709 mouse autophagy-related genes (defined in the Methods section) overlapped with the 17,771 homologous genes (Figure 1E). Among them, 531 one-to-one human-mouse homologous autophagy-related genes have been reported to regulate both human and mouse autophagy processes, and 831 human and 156 mouse one-to-one human-mouse homologous autophagy-related genes were recognized in only one species. To further categorize the co-expressed autophagy-related transcripts in the process of spermatogenesis, WGCNA was applied to the 531 genes that were homologous in human and mouse to evaluate the conservation of autophagic regulatory network and function annotations as well as the identification of key regulatory genes in the candidate modules throughout spermatogenesis between human and mouse. Notably, 6 human and 5 mouse modules exhibited stage-specific expression in spermatogenesis, and Pearson correlation coefficients (top) and *P* values (bottom) were shown in some cells (Figure 1F, Table S4).

To better understand the gene modules in WGCNA, Gene Ontology analysis and hub gene identification were performed on these modules both in human and mouse, we found that these gene clusters were associated with “response to starvation” in spermatogonia, “cell cycle” and “DNA damage and repair” in spermatocytes, “chromatin modification” and “autophagy” in spermatids (Figure 1G, Table S5 for GO analysis and Table S6 for hub gene identification). Similar results were also observed in 831 human and 156 mouse homologous autophagy-related genes (Figure S1F). Again, these findings confirmed the previously described stage-specific enrichment of autophagy-related genes during human and mouse spermatogenesis.

We further evaluated the conservation of 831 human and 156 mouse autophagy-related genes by phastCons score cumulative distributions, and the results suggested that the 531 one-to-one human-mouse homologous autophagy-related genes were relatively conserved, while the nonhomologous autophagy-related genes (49 in humans and 22 in mice) were much less conserved (Figure S1G). Importantly, the 831 human and 156 mouse autophagy-related genes could be divided into 4 clusters according to their expression patterns, including DEGs, widely expressed genes, rarely expressed genes and undetectable genes (Figure S1H). Of note, the top 5 conserved DEGs in each stage of human and mouse spermatogenesis were highlighted in Figure 1H and Figure S1I, respectively (Table S7), providing a reference for further analysis of autophagy-related candidates involved in spermatogenesis.

### Dysregulation of the autophagic transcriptome in male infertility

**Figure 2:**
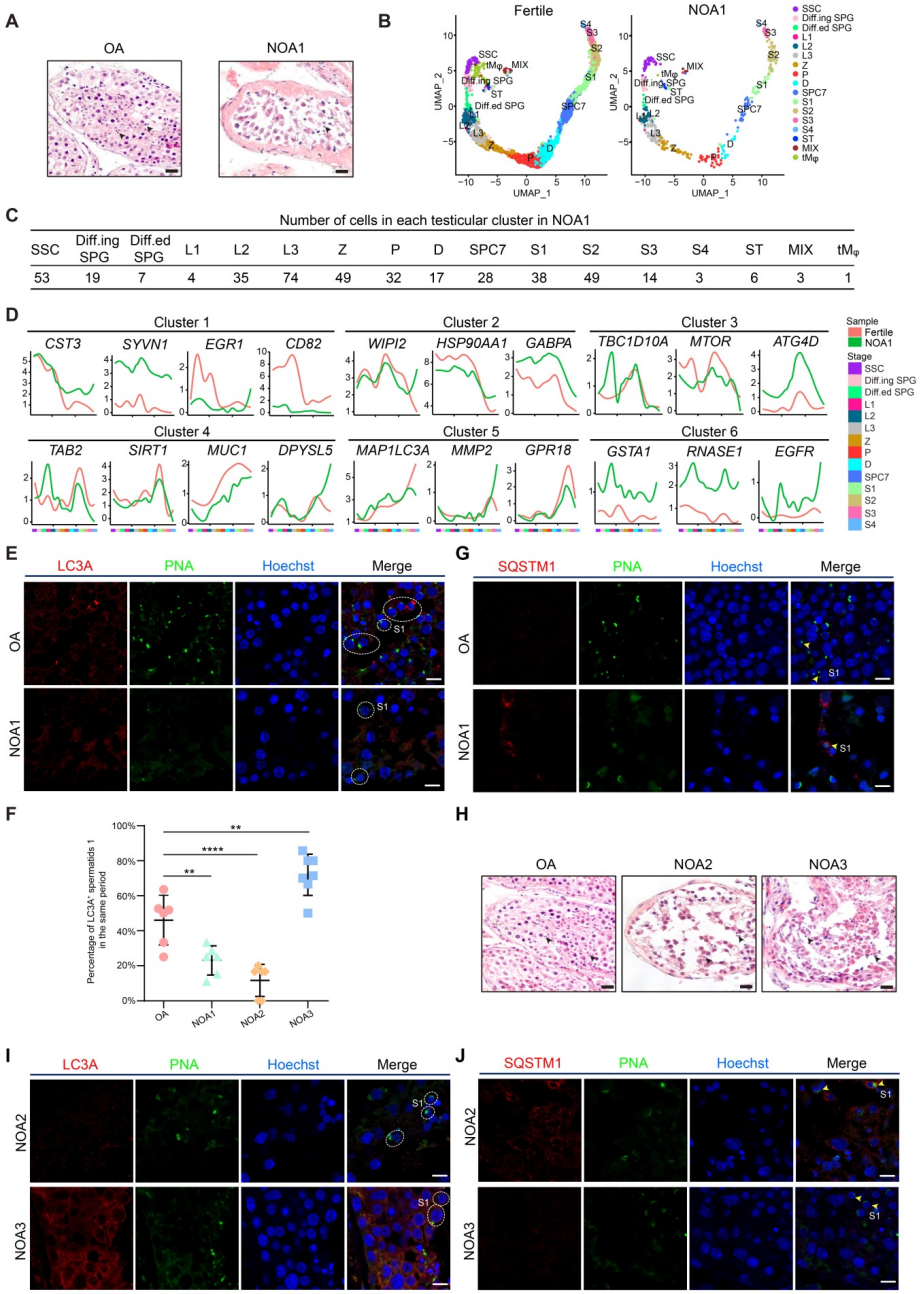
** Dysregulation of the autophagic transcriptome in male infertility. (A)** H&E staining of testicular sections from one donor with OA (left) and one donor with NOA (NOA1, right). Arrowheads indicate spermatids. The scale bars represent 40 μm. **(B)** UMAP plots of adult human testicular cells of donors with normal fertility (left) and one NOA patient (right). **(C)** Number of testicular cells in each cluster from patient NOA1. Spermatogonial stem cell (SSC), differentiating spermatogonia (Diff.ing SPG), differentiated spermatogonia (Diff.ed SPG), leptotene 1 (L1), leptotene 2 (L2), leptotene 3 (L3), zygotene (Z), pachytene (P), diplotene (D), spermatocyte 7 (SPC7), spermatid 1 (S1), spermatid 2 (S2), spermatid 3 (S3), spermatid 4 (S4), Sertoli cells (ST), peritubular myoid cells and Leydig cells (MIX), testicular macrophages (tMφ). **(D)** Line chart showing the relative expression patterns of the stage-specific autophagic genes in each testicular cell cluster between fertile donors and patient NOA1. **(E)** Immunofluorescence of LC3A (red) and PNA (green) in adult human testicular paraffin sections from one OA donor as a control (top) and one NOA patient (NOA1, bottom), spermatid 1 (S1). The scale bars represent 10 μm. **(F)** Dot plot showing the percentage of LC3A^+^ spermatids 1 (S1) among testicular cells within the same vision in the sections of one OA donor as a control and 3 NOA patients (NOA1, NOA2 and NOA3) referring to Figures 2E and 2I. **P < 0.01, ****P < 0.0001. **(G)** Immunofluorescence of SQSTM1 (red) and PNA (green) in adult human testicular paraffin sections from one OA donor as a control (top) and from patient NOA1 (bottom); yellow arrowheads indicate spermatids 1 (S1). The scale bars represent 10 μm. **(H)** H&E staining of testicular sections from one OA donor as a control (left) and from patient NOA2 (middle) and patient NOA3 (right). The scale bars represent 40 μm. **(I)** Immunofluorescence of LC3A (red) and PNA (green) in adult human testicular paraffin sections from patient NOA2 (top) and NOA3 patients (bottom). The scale bars represent 10 μm. **(J)** Immunofluorescence of SQSTM1 (red) and PNA (green) in adult human testicular paraffin sections from NOA2 (top) and NOA3 (bottom) patients; yellow arrowheads indicate spermatids 1 (S1). The scale bars represent 10 μm.

We then questioned whether the proper enrichment of autophagic genes in a stage-specific manner is functionally relevant; thus, the global expression patterns of autophagic genes in nonobstructive azoospermia (NOA) patients were analyzed. A total of 432 quality-controlled testicular cells from the NOA patient were collected for scRNA-seq analysis (Figure 2A). We detected 10,127 genes and 116,938 UMIs on average in each individual cell of the NOA patient (Figure S2A). Dimension reduction and clustering analysis were then performed to classify these testicular cells according to the global transcriptional profiles in an unbiased manner. We found that although nearly all testicular cell types (from SSC to spermatid 4 (S4) and 3 clusters of testicular somatic cells) could be detected in this patient (Figures 2B, 2C, S2B and Table S8), the proportion of spermatids (S4) was significantly decreased compared to that in fertile controls (Figure S2C), as confirmed by H&E staining and immunostaining analysis (Figures 2A, S2D). Then, we compared the differences in the expression of autophagy-related genes between testicular cells from fertile and NOA1 donors; notably, we observed significant variations between patient NOA1 and fertile control in autophagic genes but not that of germ cell stage-specific genes in all defined clusters in each stage of spermatogenesis (Figures 2D, S2E and Table S9). This finding indicates that transcriptional alteration of autophagic genes might contribute to NOA pathogenesis.

Among these genes, we noticed that *LC3A*, which was enriched in round and elongating spermatids, exhibited low transcript levels in patient NOA1 (Figure 2D). The immunostaining results confirmed that the protein level of LC3A was much lower in the spermatids of NOA1 than in those of fertile persons (Figures 2E and 2F). Consistently, we found that the level of SQSTM1 (p62) was increased in patient NOA1 (Figure 2G). Similar autophagy suppression was also detected in another NOA patient's spermatids (NOA2). Moreover, we noticed an exception: in patient NOA3, an increased LC3A and declined SQSTM1 signals were observed (Figures 2H, 2I, 2F and 2J, Figure S2F). These findings indicate that dysregulated autophagy activity is highly associated with spermatogenesis impairment and human male infertility.

Moreover, scRNA-seq data obtained throughout human spermatogenesis have allowed the evaluation of their transcriptional signatures of certain classical regulatory genes that have been implicated in autophagy in mice.

For instance, the key regulator of autophagy *MTOR* was found to be mainly expressed in human spermatogonia and late-stage spermatocytes (Figure S2G). *LAMP2*, a key component of chaperone-mediated autophagy, was found to be highly expressed in human spermatogonia (Figure S2G). Moreover, several classical autophagic genes were found to be actively expressed in human spermatogonia (*AKT1*, *ATG5*, *EPG5*, *TBC1D20*), spermatocytes (*PRKACA*, *ATG7*, *SIRT1*, *RARA*) and spermatids (*TSC1*) (Figure S2G). These results provide an overview of classical autophagic gene transcription in spermatogenesis at single-cell resolution.

### Validation of autophagic gene expression in human and mouse spermatogenesis

**Figure 3:**
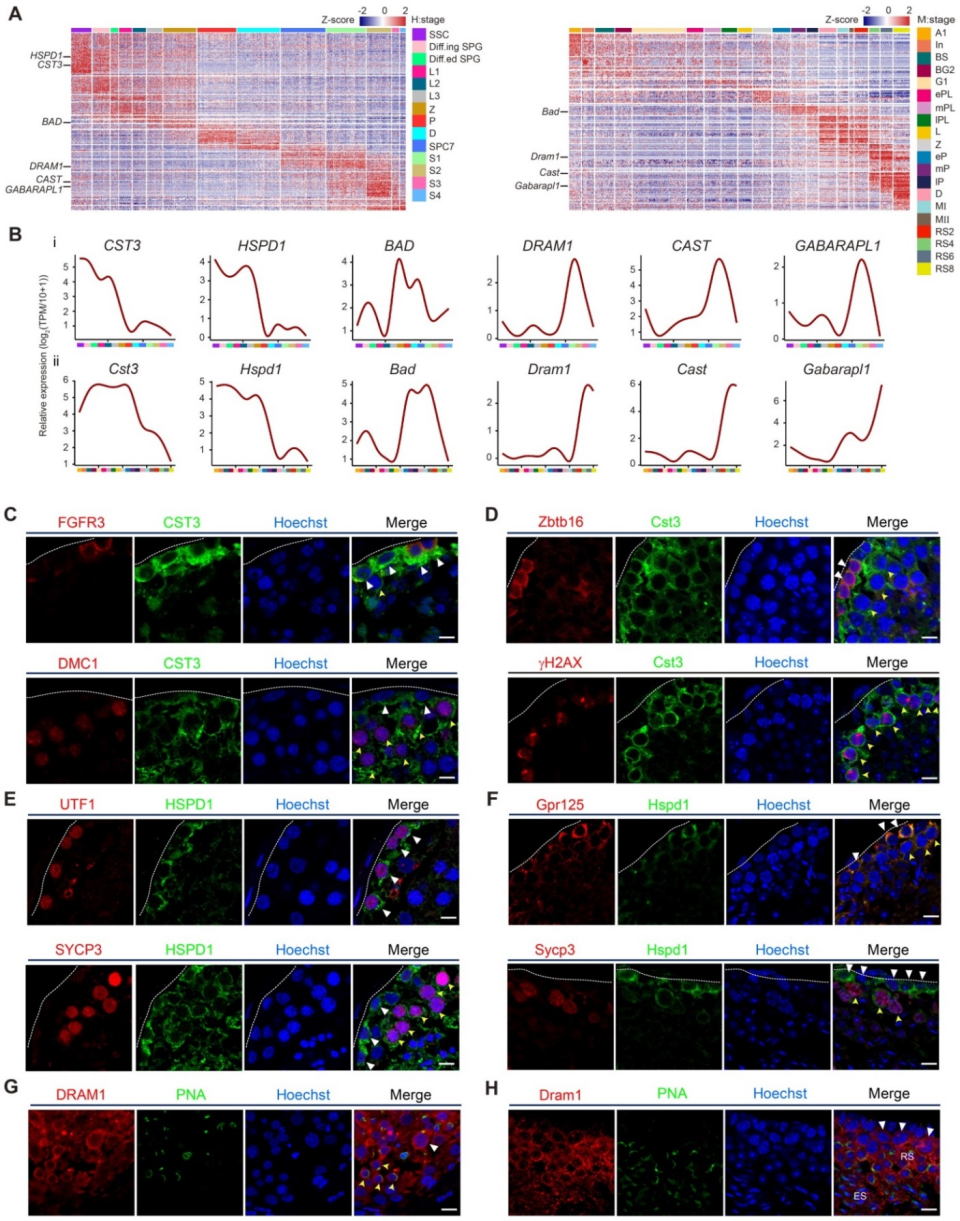
** Validation of autophagic gene expression in human and mouse spermatogenesis. (A)** Heatmap of differentially expressed autophagic genes in adult human (left) and mouse (right) spermatogenic cells. The color key from blue to red indicates low to high gene expression levels. **(B)** Line chart showing the relative expression patterns of novel autophagic genes in each human- and mouse-spermatogenic cell cluster (i: human, ii: mouse). **(C)** Immunofluorescence of FGFR3 (red, top) and DMC1 (red, bottom) co-stained with CST3 (green) in adult human testicular paraffin sections from one donor with OA. Triangles indicate SSCs, yellow arrowheads indicate spermatocytes. The scale bars represent 10 μm. **(D)** Immunofluorescence of Zbtb16 (red, top) and γH2AX (red, bottom) co-stained with Cst3 (green) in adult mouse testicular paraffin sections from 8-week-old mice. Triangles indicate spermatogonia, and yellow arrowheads indicate spermatocytes. The scale bars represent 10 μm. **(E)** Immunofluorescence of UTF1 (red, top) and SYCP3 (red, bottom) co-stained with HSPD1 (green) in adult human testicular paraffin sections from one donor with OA. Triangles indicate SSCs, yellow arrowheads indicate spermatocytes. The scale bars represent 10 μm. **(F)** Immunofluorescence of Gpr125 (red, top) and Sycp3 (red, bottom) co-stained with Hspd1 (green) in adult mouse testicular paraffin sections from 8-week-old mice. Triangles indicate spermatogonia, yellow arrowheads indicate spermatocytes. The scale bars represent 10 μm. **(G)** Immunofluorescence of DRAM1 (red) co-stained with PNA (green) in adult human testicular paraffin sections from one donor with OA. Triangle indicates spermatocyte, and yellow arrowheads indicate round spermatids. The scale bar represents 10 μm. **(H)** Immunofluorescence of Dram1 (red) co-stained with PNA (green) in adult mouse testicular paraffin sections from 8-week-old mice. Triangles indicate spermatocytes; RS, round spermatids; ES, elongated spermatids. The scale bar represents 10 μm.

Among 531 one-to-one human-mouse homologous autophagy-related genes, we were particularly interested in stage-specific autophagy-related genes in spermatogenesis. The transcriptional patterns of these genes were generally consistent between humans and mice (Figure 3A, Table S10). Moreover, several autophagy-related homologs were selected for further analyses in the context of human and mouse spermatogenesis. Interestingly, *CST3* and *HSPD1* were highly expressed from human SSCs to the early stage of spermatocytes, and *BAD* was significantly upregulated in zygotene spermatocytes; however, *DRAM1*, *CAST*, and *GABARAPL1* were only actively expressed in spermatids (Figures 3B, S3A). Similar expression patterns were also observed in mouse spermatogenesis (Figures 3B, S3B).

To visualize the expression and localization of autophagy-related genes *in situ*, we performed immunostaining analysis. Indeed, our results indicated that CST3 and HSPD1 were highly expressed in both human and mouse spermatogonia and leptotene spermatocytes, as confirmed by co-staining with each of their stage-specific germ cell markers (Figures 3C, 3D, 3E and 3F). DRAM1 was highly expressed in late spermatocytes and round spermatids, as indicated by co-staining with PNA (Figures 3G, 3H).

### Cst3 plays a critical role in the maintenance of mSSCs by regulating autophagy

The stage-specific enrichment of autophagy-related genes is highly conserved between human and mouse spermatogenesis, as identified by our scRNA-seq analysis. Previous studies have been focused on the role of autophagy in somatic cells and spermatids but not SSCs, which might be critical for the maintenance of SSC pool and subsequent meiosis. Then, we selected Cst3, an autophagy-related gene that showed very similar expression patterns between humans and mice, and explored its biological significance in spermatogenesis using a well-defined mouse model.

**Figure 4:**
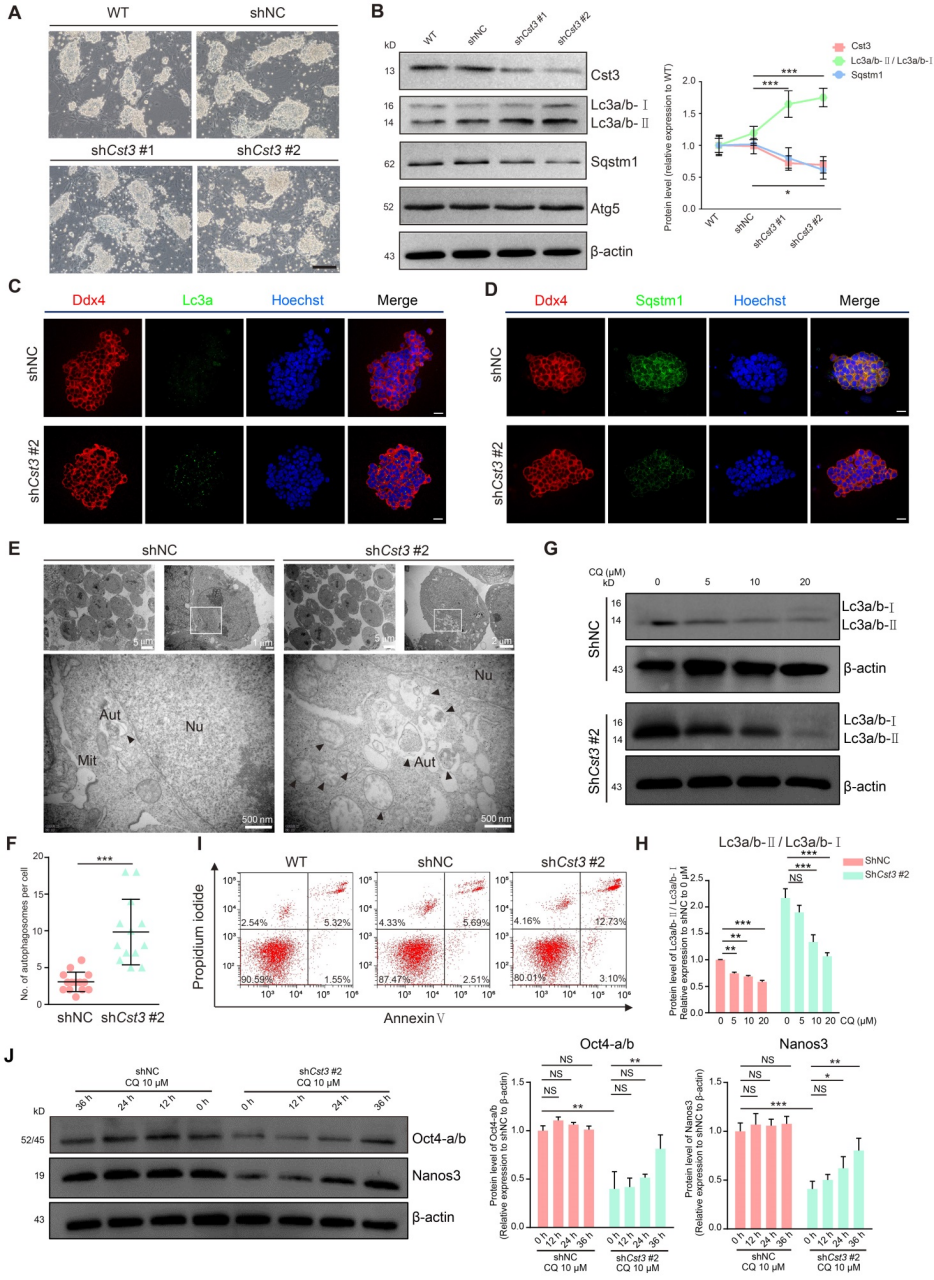
** Cst3 plays a critical role in the maintenance of mSSCs by regulating autophagy. (A)** Bright field images of wild-type (WT) mSSCs, mSSCs transfected with shNC and *Cst3*-targeted shRNAs (sh*Cst3* #1 and sh*Cst3* #2). The scale bars represent 200 μm. **(B)** Western blotting analysis of the protein levels of Cst3, two types of Lc3a/b, Sqstm1 and Atg5 in WT and shNC-, sh*Cst3* #1- and sh*Cst3* #2-transfected mSSCs (left). Line chart showing the grayscale intensity analysis of the bands for Cst3, two types of Lc3a/b and Sqstm1 with β-actin as the internal control (right). * *P* < 0.05; ***P < 0.001. **(C)** Immunofluorescence of Ddx4 (red) and Lc3a (green) in mouse SSCs transfected with shNC and sh*Cst3* #2. The scale bars represent 20 μm. **(D)** Immunofluorescence of Ddx4 (red) and Sqstm1 (green) in mouse SSCs transfected with shNC and sh*Cst3* #2. The scale bars represent 20 μm. **(E)** TEM analysis of autophagosomes in mouse SSCs transfected with shNC and sh*Cst3* #2. Nu, nucleus; Mit, mitochondrion; AUT, autophagosomes. **(F)** Dot plot showing the average numbers of autophagosomes in each mouse SSC transfected with shNC and sh*Cst3* #2. ***P < 0.001. **(G)** Western blotting analysis of the protein levels of two types of Lc3a/b in shNC- and sh*Cst3* #2-transfected mSSCs after treatment with different concentrations of CQ for 24 h. **(H)** Bar plot showing the grayscale intensity analysis of two types of Lc3a/b in shNC- and sh*Cst3* #2-transfected mSSCs after treatment with different concentrations of CQ for 24 h, with β-actin as the internal control. NS, not significant; **P < 0.01; ***P < 0. 001. **(I)** Flow cytometry analysis of cell death in WT and shNC- and sh*Cst3* #2-transfected mSSCs through double staining with annexin V-FITC and PI. **(J)** Western blotting analysis of the protein levels of Oct4 and Nanos3 in shNC- and sh*Cst3* #2-transfected SSCs treated with CQ (left) for different time periods. Bar plot showing the grayscale intensity analysis of Oct4 and Nanos3, with β-actin as the internal control (right). NS, not significant; *P < 0.05; **P < 0.01; ***P < 0. 001.

To examine the function of Cst3 in mSSCs, we generated two independent *Cst3* knockdown (*Cst3-*KD) mSSC lines using shRNAs; in both these cell lines, the mRNA and protein levels of Cst3 were dramatically decreased (Figures 4A, 4B and S4A). Since *Cst3* serves as an autophagic player in other systems 35-37, we evaluated the autophagy activity of both *Cst3*-KD SSCs and control SSCs. Indeed, we found that both the transcript level of *Lc3a* and the protein ratio of Lc3a/b-Ⅱ/Lc3a/b-Ⅰ (the density of membrane-associated Lc3a/b-Ⅱ versus cytosolic Lc3a/b-Ⅰ) were upregulated in *Cst3*-KD SSCs compared to control SSCs, while Sqstm1 expression was decreased in *Cst3*-KD SSCs, as revealed by western blotting analysis. However, the expression of classical autophagy genes (Atg3, Atg5 and Atg7) exhibited no significant change (Figures 4B and S4B). Immunostaining analysis further verified the increase in Lc3a puncta and the decrease in Sqstm1 expression in *Cst3*-KD SSCs compared to that in controls (Figures 4C, 4D). In line with this, TEM analysis showed that Cst3 knockdown led to obvious autophagosome accumulation in *Cst3*-KD SSCs (Figures 4E and 4F). Furthermore, CQ and 3-MA, inhibitors of autophagy, significantly suppressed Cst3 knockdown-induced autophagy induction in mSSCs (Figures 4G and 4H, Figure S4C). Thus, we demonstrated that autophagy is activated by Cst3 knockdown, suggesting that Cst3 is a negative autophagy regulator in mSSCs.

Moreover, after propagation, *Cst3*-KD SSCs formed much smaller colonies than control SSCs (Figure 4A), which was further confirmed by the cell proliferation inhibition and G1-phase cell cycle arrest observed in *Cst3*-KD SSCs (Figures S4D, S4E, S4F). Finally, increased cell death (Annexin V^+^ PI^+^ cells, the indicator of late-stage apoptotic cells and other kinds of dead cells) was detected in *Cst3*-KD SSCs (Figure 4I), suggesting a critical role of Cst3 in mSSC maintenance. Recently, autophagy has been identified as an important regulator of embryonic stem cell pluripotency 38. To determine whether CST3-regulated autophagy affects SSC maintenance in germline stem cells, core factors were analyzed in *Cst3*-KD SSCs. The expression of SSC markers (*Zbtb16*, *Gfra1* and *Ddx4*) was comparable between *Cst3*-KD SSCs and control SSCs (Figures S4G, S4H); however, the expression of SSC maintenance-related genes, including *Oct4*, *Id1* and *Nanos3,* was significantly decreased in CST3-deficient cells (Figure 4J, Figures S4I, S4J, S4K). Interestingly, the autophagy inhibitors CQ and 3-MA partially restored the expression of these genes in *Cst3*-KD SSCs (Figures 4J, S4K). Together, these findings demonstrate that Cst3-mediated autophagy plays important roles in SSC maintenance by regulating the expression of core factors.

### Cst3 regulates male meiosis

**Figure 5:**
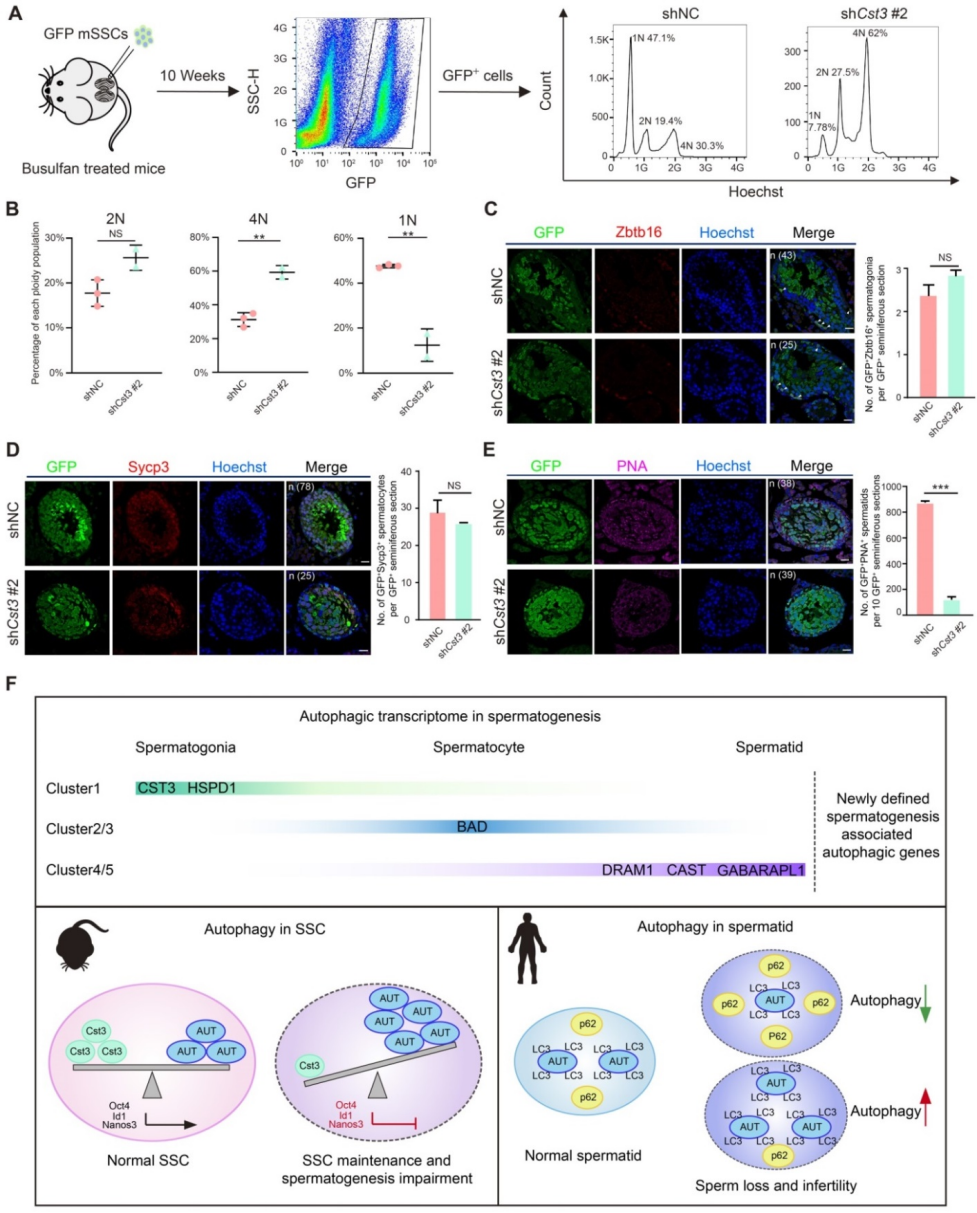
** Cst3 regulates male meiosis. (A)** Flow chart showing the strategy for characterizing the functions of Cst3 in mouse spermatogenesis via flow cytometry analysis of the distribution of each ploidy population in the sorted GFP^+^ cells after testicular transplantation with shNC- and sh*Cst3* #2-transfected SSCs labeled with GFP. **(B)** Dot plots showing the percentage of each ploidy population among the sorted GFP^+^ cells after testicular transplantation with shNC- and shCst3 #2-transfected mSSCs labeled with GFP. NS, not significant; **P < 0.01. **(C)** Immunofluorescence of GFP (green) and Zbtb16 (red) in testicular paraffin sections transplanted with shNC- and shCst3 #2-transfected mSSCs. 'n' represents the number of testicular sections calculated in this panel. Triangles indicate double positive SSCs. The scale bars represent 20 μm. Box plot showing the number of GFP^+^Zbtb16^+^ spermatogonia per GFP^+^ seminiferous section, NS, not significant (right). **(D)** Immunofluorescence of GFP (green) and Sycp3 (red) in testicular paraffin sections transplanted with shNC- and shCst3 #2-transfected mSSCs. 'n' represents the number of testicular sections calculated in this panel. The scale bars represent 20 μm. Box plot showing the number of GFP^+^Sycp3^+^ spermatocytes per GFP^+^ seminiferous section, NS, not significant (right). **(E)** Immunofluorescence of GFP (green) and PNA (pink) in testicular paraffin sections transplanted with shNC- and shCst3 #2-transfected mSSCs. 'n' represents the number of testicular sections calculated in this panel. The scale bars represent 20 μm. Box plot showing the number of GFP^+^PNA^+^ spermatids per 10 GFP^+^ seminiferous sections, ***P < 0.001 (right). **(F)** Summary of the study. The expression patterns of newly identified spermatogenesis-associated autophagic genes throughout spermatogenesis. Cst3 is a negative autophagy regulator in mSSCs. Cst3-mediated autophagy plays important roles in SSC maintenance by regulating the expression of core factors (*Oct4, Id1,* and* Nanos3*). Data from NOA patients revealed that autophagy homeostasis is required for normal spermatogenesis and that dysfunctions in autophagy activity are highly associated with human male infertility.

Since *Cst3* was expressed in SSCs and throughout differentiation from spermatogonia to early spermatocytes (Figures 3B, 3C, 3D), we next questioned whether Cst3 contributes to meiosis. Therefore, a transplantation assay was performed with GFP-labeled *Cst3*-KD SSCs, which were injected into busulfan-treated male mice to explore the function of Cst3 *in vivo*. Ten weeks after seminiferous tubule transplantation, flow cytometry analysis revealed that the distribution of each ploidy population among the GFP-positive cells in recipient testes was dramatically changed in *Cst3*-KD groups compared to that in Cst3-competent controls. Namely, the diploid cell proportion was slightly increased; however, the proportion of tetraploid cells was significantly increased, while the proportion of haploid cells was obviously decreased (Figures 5A, 5B, S5A), indicating that meiosis was perturbed upon Cst3 knockdown.

To further analyze the meiotic progression of transplanted SSCs, we examined the expression of classical stage-specific markers for spermatogenesis. Upregulation of spermatogonia and early spermatocyte marker genes (*Zbtb16*, *Nanos3*, *Kit*, *Dazl*, *Stra8*, *Dmc1*) and downregulation of late spermatocyte and spermatid marker genes (*Sycp3* and *Pou5f2* for spermatocytes, *Acr*, *Trim36*, *Tnp1* and *Prm1* for spermatids) were observed in germ cells derived from GFP^+^ *Cst3*-KD SSCs (Figure S5B). Moreover, the number of GFP^+^PNA^+^ cells but not that of GFP^+^Zbtb16^+^ and GFP^+^Sycp3^+^ cells in each GFP^+^ seminiferous tubule was significantly decreased in testes transplanted with *Cst3*-KD SSCs compared to those transplanted with control SSCs, indicating that the suppression of Cst3 in SSCs impairs the production of spermatid or sperm (Figures 5C, 5D, 5E, S5C, S5D, S5E). Thus, Cst3 also serves as an important regulator of late-stage germ cell development.

## Discussion

Spermatogenesis is critical for the generation of sperm during almost the whole lifespan of male mammals. Autophagy, a resource allocation process regulated by a series of factors, plays important roles in spermatogenesis. For instance, Atg7 is associated with acrosome formation, flagella biogenesis and cytoplasm removal 16, 39, and Atg5 plays roles in head and tail construction 40; Atg7 and Atg5 are also involved in testosterone synthesis 14. However, the transcriptional signature of other autophagic genes and its significance in each cell type during human and mouse spermatogenesis remain unclear.

In the present study, the transcriptional profiles of autophagy-related genes throughout normal spermatogenesis and male infertility were explored via scRNA-seq analysis. Although the cell number used in this NOA patient (432 cells) for scRNA-seq was relatively low compared to other studies; however, the library construction strategy used in this study was STRT-seq, which could capture around 8,000-12,000 genes versus 5,000-6,000 genes by 10 × Genomics Chromium or Drop-seq in each individual cell.

Therefore, our scRNA-seq method required less single cell number (three or more single cell per cluster) for bioinformatic analysis. In addition, although 432 cells were used in NOA1 patient analysis, nearly all testicular cell types throughout human spermatogenesis, including 14 types of germ cells and 3 clusters of somatic cells, could be captured. In this study, we found a unique stage-specific enrichment of autophagy-related genes in human and mouse spermatogenesis, which was highly conserved in mammals. However, dysregulation of this stage-specific expression pattern of autophagy-related genes was found in one infertile patient, and dysregulated autophagy activity was also observed in two additional NOA patients. Among these three NOA samples, testicular cells of NOA3 showed increased autophagy activity by immunostaining of LC3A and SQSTM1, while it was contrast to that of NOA1 and NOA2. These findings showed that both increased or decreased autophagy activity was associated with spermatogenesis impairment and human male infertility, indicating the requirement of autophagy homeostasis for normal spermatogenesis (Figure 5F).

Moreover, we have tried to find whether dysregulated transcription of autophagic genes in more NOA patients; however, based on the data of this study and another scRNA-seq project of our group, only one patient (NOA1) exhibited transcriptional dysregulation of autophagic genes. Similar autophagy abnormality was also observed in another two patients (NOA2, NOA3) by immunostaining of key autophagic marker genes. But NOA2 and NOA3 samples cannot be used for scRNA-seq analysis as they were derived from our specimen bank preserved in paraformaldehyde. Together, based on the results of three NOA samples in this study, we have revealed the relationship between autophagy dysregulation and human male infertility, and further studies will be aimed to establish the detailed autophagic regulatory networks in NOA patients.

Cst3 encodes a cysteine protease inhibitor that is mainly expressed from SSCs to early spermatocytes and plays critical roles in both mSSC maintenance *in vitro* and meiotic progression after mSSC transplantation by regulating autophagy. The protective role of Cst3 in other diseases, such as neurodegenerative and cardiovascular disease, has been proven: Cst3 induces autophagy in response to injury and stress 36, 37. However, a novel role of Cst3 as an autophagy suppressor that maintains autophagy homeostasis in mSSCs was uncovered in this study. Furthermore, crosstalk between CST3-mediated autophagy and SSC maintenance was uncovered. Core pluripotency factors (Oct4, Id1) and key RNA binding protein (Nanos3) were identified to relay the signals from Cst3-mediated autophagy. Compared with the reported roles of autophagy in round spermatids, Sertoli cells as well as Leydig cells, our study revealed the regulatory role of autophagy in SSC maintenance via the regulation of pluripotency and other core factors. Future studies will aim to establish the sophisticated regulatory mechanisms between autophagy and SSC pluripotency as that of a recent study, which uncovered the role of chaperone-mediated autophagy in embryonic stem cells 38. Our data suggest that proper autophagy activity is pivotal for spermatogenesis, providing new angles to reconsider in the context of SSC fate determination via autophagy manipulation (Figure 5F).

Traditional studies of human male infertility have mainly depended on genome-wide association studies (GWASs) or whole-exome sequencing (WES) of consanguineous families and bulk RNA based studies 41 to identify potential mutations followed by recapitulation of the phenotype via genetic manipulation in animal models 42. The current study revealed conserved and stage-specific patterns of autophagy-related genes during mouse and human spermatogenesis via single-cell transcriptome analysis. Besides, we provided an overview of classical autophagic gene transcription in spermatogenesis at single-cell resolution, mutations of these genes were reported to cause germ cell loss (*MTOR*) 43 or infertility (*LAMP2*) 44, suggesting the potential roles of other classical autophagic genes that were actively expressed in human spermatogonia (*AKT1* 45, *ATG5* 40, *EPG5* 46, *TBC1D20* 18), spermatocytes (*PRKACA* 47, *ATG7* 16, *SIRT1* 17, *RARA* 48) and spermatids (*TSC1*) 49. Moreover, comparison analysis of the stage-specific autophagic profiles between the testicular cells of fertile and infertile donors defined the pathologic factors contributing to dysregulated autophagy, providing potential targets for the development of new diagnostic and therapeutic approaches for male infertility 50. Most importantly, our study has now established a powerful platform for rapidly screening autophagy-associated candidates in various pathological conditions, which can be further investigated with cultured cells and animal models, shedding light on novel diagnosis and clinical management strategies for male infertility.

## Conclusion

Our study provides an overview of the regulatory network of autophagy in both human and mouse spermatogenesis. This network analysis and single-cell transcriptomic analysis highlighted the stage-specific autophagy-related genes involved in the regulation of each stage of spermatogenesis, and these transcriptome profiles were conserved between humans and mice. The expression patterns of autophagy-related genes were altered in infertile patients compared to fertile controls, suggesting the regulatory role of the autophagy-related network in human spermatogenesis and infertility. Among the newly defined autophagy-related genes, Cst3 was uncovered to be important for SSC maintenance and subsequent male germ cell development. Together, our study dissected the autophagy regulatory network in spermatogenesis and offered an example of the functional characterization of a newly defined autophagy-related gene, providing novel insights into spermatogenesis and male infertility. More work is needed to determine the detailed roles of other candidate autophagy-related genes in spermatogenesis.

## Supplementary Material

Supplementary figures and table legends.

Supplementary references 1.

Supplementary references 2.

Supplementary table 1.

Supplementary table 2.

Supplementary table 3.

Supplementary table 4.

Supplementary table 5.

Supplementary table 6.

Supplementary table 7.

Supplementary table 8.

Supplementary table 9.

Supplementary table 10.

Supplementary table 11.

## Abbreviations

3-MA: 3-Methyladenine
CQ: Chloroquine
Cst3: Cystatin C
DEGs: differentially expressed genes
DMSO: dimethylsulfoxide
FACS: fluorescence-activated cell sorting
H&E: hematoxylin and eosin
hSSCs: human spermatogonial stem cells
KD: knockdown
MGI: Mouse Genome Informatics
mSSCs: mouse spermatogonial stem cells
NOA: nonobstructive azoospermia
OA: obstructive azoospermia
q-PCR: quantitative polymerase chain reaction
scRNA-seq: single-cell RNA sequencing
shRNA: small hairpin RNA
SOM: self-organizing maps
TEM: transmission electron microscopy
TPM: transcripts per million
UMI: unique molecular identifier
WGCNA: weighted gene co-expression network analysis
GWASs: genome-wide association studies
WES: whole-exome sequencing
