# Supplementary material for: Deciphering the autophagy regulatory network via single-cell transcriptome analysis reveals a requirement for autophagy homeostasis in spermatogenesis
Source: Theranostics. 2021 Mar 5;11(10):5010–27. doi: 10.7150/thno.55645 (PMC7978313; doi:10.7150/thno.55645)
Supplement: Supplementary file 1 — Supplementary figures and table legends. [file thnov11p5010s1.pdf]

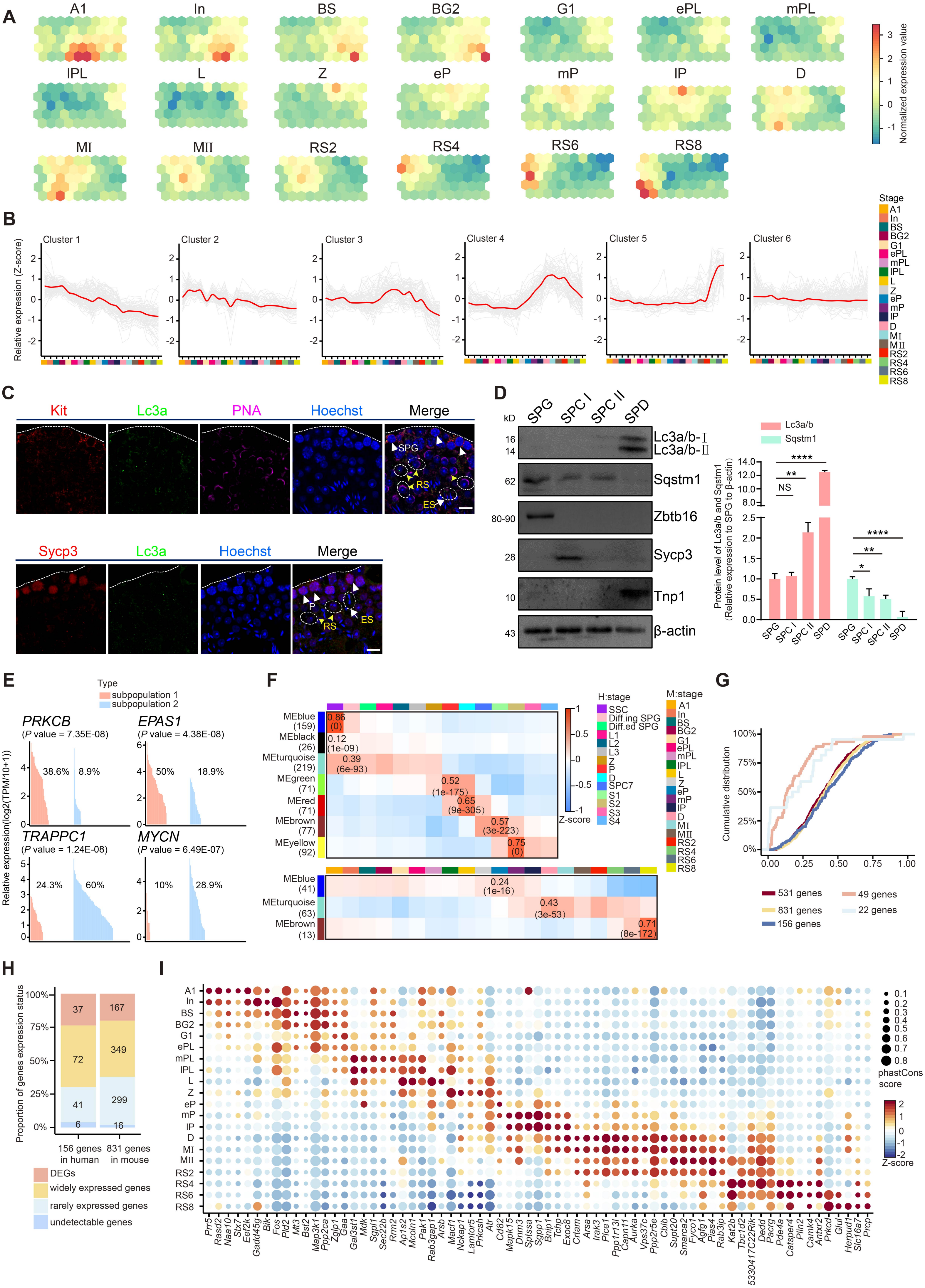
A
A1
In
BS
BG2
G1
ePL
mPL
lPL
L
Z
eP
mP
lP
D
MI
MII
RS2
RS4
RS6
RS8
Normalized expression value
B
Cluster 1
Cluster 2
Cluster 3
Cluster 4
Cluster 5
Cluster 6
Relative expression (Z-score)
Stage
C
Kit
Lc3a
PNA
Hoechst
Merge
SPG
RS
ES
Sycp3
P
D
kD
SPG
SPC I
SPC II
SPD
Lc3a/b-Ⅰ
Lc3a/b-Ⅱ
Sqstm1
Zbtb16
Sycp3
Tnp1
β-actin
Lc3a/b
Sqstm1
Protein level of Lc3a/b and Sqstm1
(Relative expression to SPG to β-actin)
NS
E
Type
subpopulation 1
subpopulation 2
PRKCB
(P value = 7.35E-08)
EPAS1
(P value = 4.38E-08)
TRAPPC1
(P value = 1.24E-08)
MYCN
(P value = 6.49E-07)
Relative expression(log2(TPM/10+1))
F
MEblue
MEblack
MEturquoise
MEgreen
MEred
MEbrown
MEyellow
H:stage
SSC
Diff.ing SPG
Diff.ed SPG
SPC7
M:stage
Z-score
G
Cumulative distribution
531 genes
831 genes
156 genes
49 genes
22 genes
H
Proportion of genes expression status
156 genes in human
831 genes in mouse
DEGs
widely expressed genes
rarely expressed genes
undetectable genes
I
phastCons score
Z-score

Figure S1. Global transcriptional dynamics of autophagic genes in spermatogenesis.

(**A**) Temporal expression of autophagic genes represented by a self-organizing map algorithm; a distinct set of autophagy-related genes are enriched in each cell cluster. A gradient of green to red indicates low to high normalized expression values. Type A1 spermatogonia (A1), intermediate spermatogonia (In), S phase type B spermatogonia (BS), G2/M phase type B spermatogonia (BG2), G1 phase preleptotene (G1), early S phase preleptotene (ePL), middle S phase preleptotene (mPL), late S phase preleptotene (lPL), leptotene (L), zygotene (Z), early pachytene (eP), middle pachytene (mP), late pachytene (lP), diplotene (D), metaphase I (MI), metaphase II (MII), steps 1–2 spermatids (RS2), steps 3–4 spermatids (RS4), steps 5–6 spermatids (RS6), steps 7–8 spermatids (RS8).

(**B**) K-means clustering of differentially expressed autophagic genes throughout mouse spermatogenesis into six individual clusters.

(**C**) Immunofluorescence of Kit (red, top) and Sycp3 (red, bottom) co-stained with Lc3a (green) and PNA (pink) in adult mouse testicular paraffin sections from 8-week-old mice. SPG, spermatogonia; P, pachytene; RS, round spermatids; ES, elongated spermatids. The scale bars represent 10 μm.

(**D**) Western blotting of Lc3a/b, Sqstm1, Zbtb16, Sycp3 and Tnp1 in the spermatogonium (SPG); primary spermatocyte (SPC I); secondary spermatocyte (SPC II); spermatid (SPD) stages (left); Bar plot showing the grayscale intensity of two types of Lc3a/b and Sqstm1 with β-actin as the internal control (right). NS, not significant; *$P < 0.05$; **$P < 0.01$; ****$P < 0.0001$.

(**E**) Bar plots showing the relative expression patterns of specific autophagic genes in two subpopulations of human SSCs. The percentage of cells expressing specific genes and *P* values in two subpopulations were shown in the plots.

(**F**) WGCNA showing gene modules derived from 831 and 156 homologous autophagic genes in Figure 1E enriched in each human (top) and mouse (bottom) spermatogenic cell cluster, respectively.

(**G**) Comparison of phastCons score cumulative distributions of the five gene sets in Figure 1E.

(**H**) Proportions of four types of genes from 831 mouse- and 156 human-homologous autophagic genes.

(**I**) Bubble plot showing the top five autophagic genes derived from 167 DEGs of 20 mouse-spermatogenic cell clusters in Figure S1G. The size of each circle represents each gene's phastCons score. The gradient of blue to red indicates low to high expression levels.

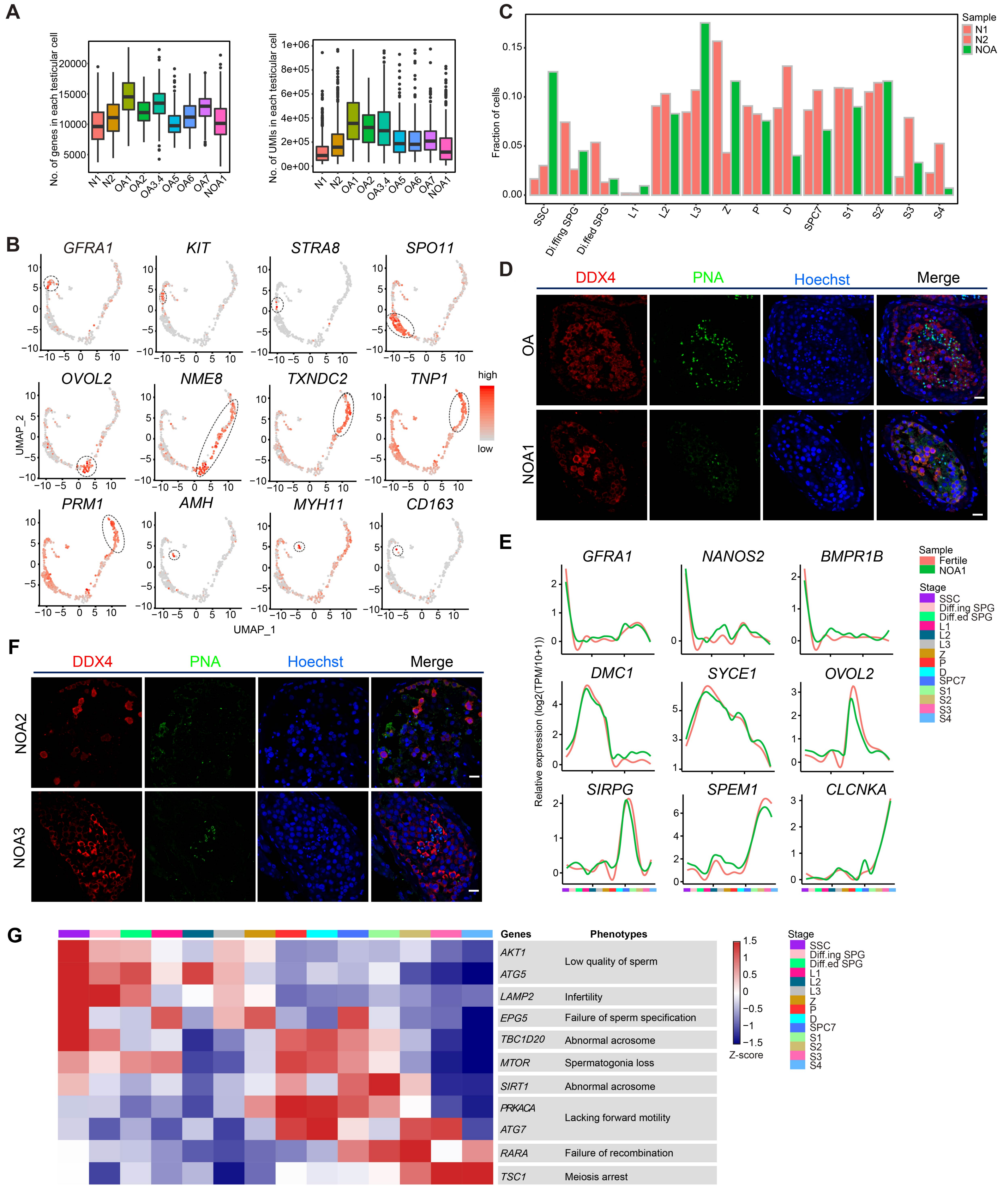

A
No. of genes in each testicular cell
No. of UMIs in each testicular cell
N1 N2 OA1 OA2 OA3.4 OA5 OA6 OA7 NOA1
B
GFRA1 KIT STRA8 SPO11
OVOL2 NME8 TXNDC2 TNP1
PRM1 AMH MYH11 CD163
UMAP_1
UMAP_2
high
low
C
Fraction of cells
Sample
N1
N2
NOA
SSC Di.ffing SPG Di.ffed SPG L1 L2 L3 Z P D SPC7 S1 S2 S3 S4
D
DDX4 PNA Hoechst Merge
OA
NOA1
E
GFRA1 NANOS2 BMPR1B
DMC1 SYCE1 OVOL2
SIRPG SPEM1 CLCNKA
Relative expression (log2(TPM/10+1))
Sample
Fertile
NOA1
Stage
SSC
Diff.ing SPG
Diff.ed SPG
L1
L2
L3
Z
P
D
SPC7
S1
S2
S3
S4
F
DDX4 PNA Hoechst Merge
NOA2
NOA3
G
Genes
Phenotypes
AKT1
ATG5
Low quality of sperm
LAMP2
Infertility
EPG5
Failure of sperm specification
TBC1D20
Abnormal acrosome
MTOR
Spermatogonia loss
SIRT1
Abnormal acrosome
PRKACA
ATG7
Lacking forward motility
RARA
Failure of recombination
TSC1
Meiosis arrest
Z-score
Stage
SSC
Diff.ing SPG
Diff.ed SPG
L1
L2
L3
Z
P
D
SPC7
S1
S2
S3
S4

Figure S2. scRNA-seq analysis of patient NOA1 and immunofluorescence staining of patients NOA2 and NOA3.

(**A**) Boxplot of the number of genes and UMIs detected in each single cell from fertile donors and patient NOA1.

(**B**) Gene expression patterns of marker genes on UMAP plots. The gradient of gray to red indicates low to high expression levels.

(**C**) Box plot showing the fraction of each germ cell stage from normal persons (N1 and N2) and one NOA patient (NOA1). Spermatogonial stem cell (SSC), differentiating spermatogonia (Diff.ing SPG), differentiated spermatogonia (Diff.ed SPG), leptotene 1 (L1), leptotene 2 (L2), leptotene 3 (L3), zygotene (Z), pachytene (P), diplotene (D), spermatocyte 7 (SPC7), spermatid 1 (S1), spermatid 2 (S2), spermatid 3 (S3), spermatid 4 (S4).

(**D**) Immunofluorescence of DDX4 (red) co-stained with PNA (green) in adult human testicular paraffin sections from one OA donor as a control (top) and one NOA patient (NOA1, bottom). The scale bars represent 20 μm.

(**E**) Line chart showing the relative expression patterns of stage-specific germ cell marker genes in each human testicular cell cluster.

(**F**) Immunofluorescence of DDX4 (red) co-stained with PNA (green) in testicular paraffin sections from NOA2 (left) and NOA3 patients (right). The scale bars represent 20 μm.

(**G**) Heatmap of autophagic genes related to male infertility throughout human spermatogenesis. The color key from blue to red indicates low to high gene expression levels, respectively.

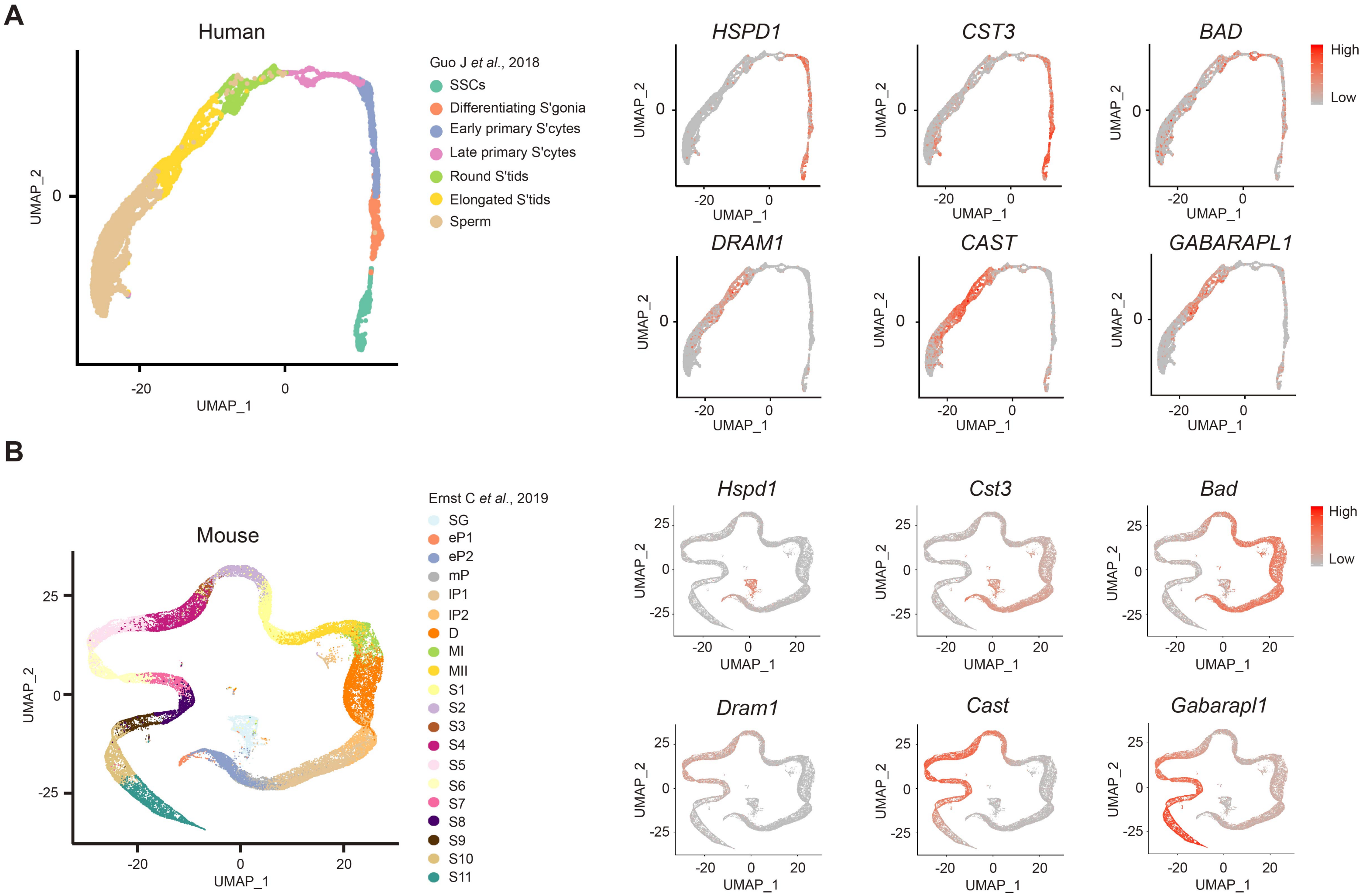
A
Human
Guo J et al., 2018
SSCs
Differentiating S'gonia
Early primary S'cytes
Late primary S'cytes
Round S'tids
Elongated S'tids
Sperm
UMAP_1
UMAP_2
HSPD1
CST3
BAD
DRAM1
CAST
GABARAPL1
High
Low
B
Mouse
Ernst C et al., 2019
SG
eP1
eP2
mP
lP1
lP2
D
MI
MII
S1
S2
S3
S4
S5
S6
S7
S8
S9
S10
S11
Hspd1
Cst3
Bad
Dram1
Cast
Gabarapl1

Figure S3. Validation of the gene expression profiles of autophagic genes using other human and mouse spermatogenesis-associated scRNA-seq datasets.

(**A**) UMAP showing the expression pattern of the six autophagic genes of Figure 3B in the human spermatogenesis-associated scRNA-seq dataset (Guo J *et al*., 2018).

(**B**) UMAP showing the expression pattern of the six autophagic genes of Figure 3B in the mouse spermatogenesis-associated scRNA-seq dataset (Emst C *et al*., 2019).

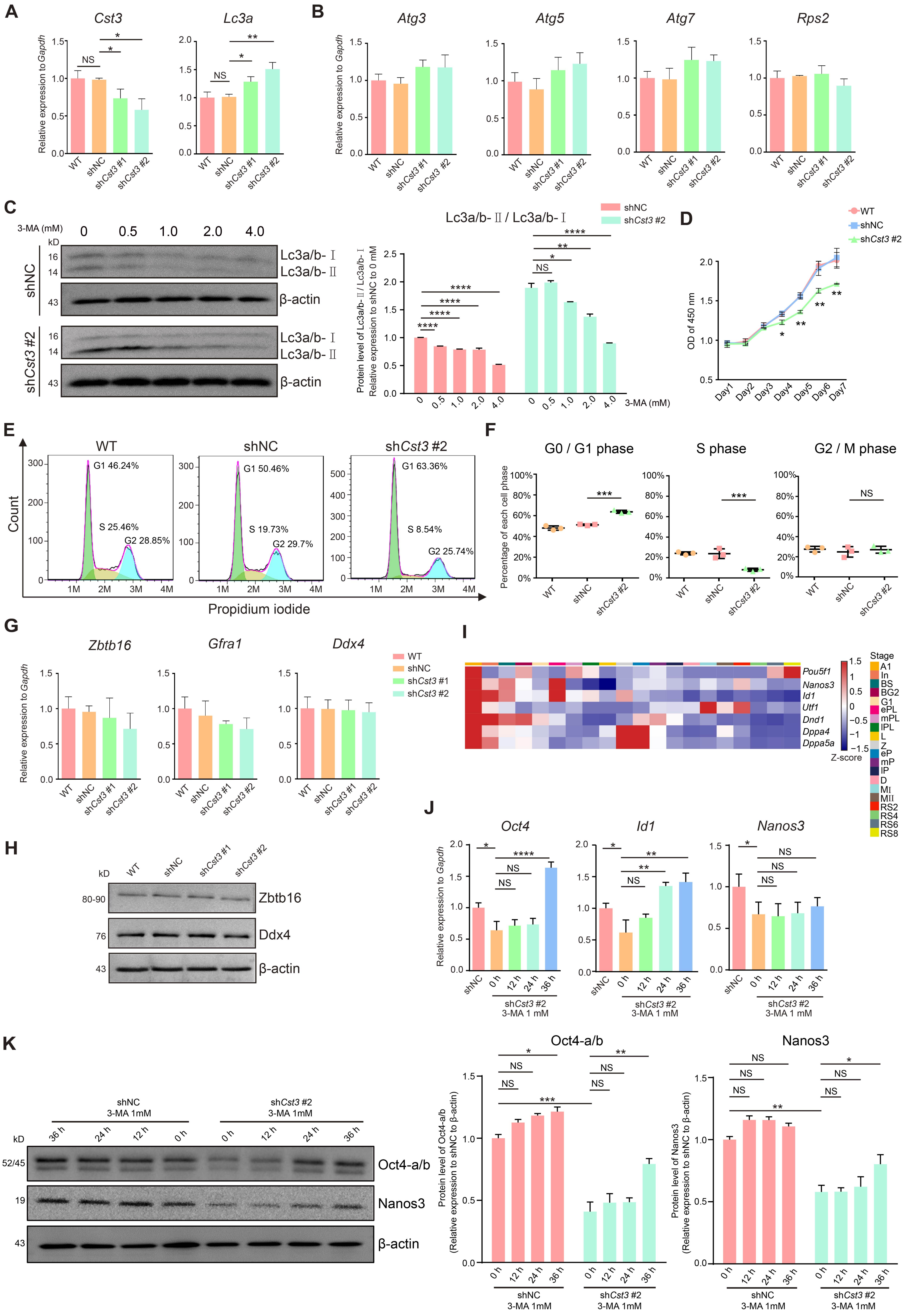

A
Cst3
Lc3a
Relative expression to Gapdh
B
Atg3
Atg5
Atg7
Rps2
WT
shNC
shCst3 #1
shCst3 #2
C
3-MA (mM)
0
0.5
1.0
2.0
4.0
kD
shNC
shCst3 #2
Lc3a/b- Ⅰ
Lc3a/b- Ⅱ
β-actin
Lc3a/b- Ⅱ / Lc3a/b- Ⅰ
Protein level of Lc3a/b- Ⅱ / Lc3a/b- Ⅰ
Relative expression to shNC to 0 mM
3-MA (mM)
D
OD of 450 nm
Day1
Day2
Day3
Day4
Day5
Day6
Day7
E
Count
WT
shNC
shCst3 #2
G1 46.24%
S 25.46%
G2 28.85%
G1 50.46%
S 19.73%
G2 29.7%
G1 63.36%
S 8.54%
G2 25.74%
Propidium iodide
F
G0 / G1 phase
S phase
G2 / M phase
Percentage of each cell phase
G
Zbtb16
Gfra1
Ddx4
H
Zbtb16
Ddx4
β-actin
I
Pou5f1
Nanos3
Id1
Utf1
Dnd1
Dppa4
Dppa5a
Z-score
Stage
A1
In
BS
BG2
G1
ePL
mPL
lPL
L
Z
eP
mP
lP
D
MI
MII
RS2
RS4
RS6
RS8
J
Oct4
Id1
Nanos3
shCst3 #2
3-MA 1 mM
K
shNC
3-MA 1mM
shCst3 #2
3-MA 1mM
Oct4-a/b
Nanos3
β-actin
Protein level of Oct4-a/b
(Relative expression to shNC to β-actin)
Protein level of Nanos3
(Relative expression to shNC to β-actin)

Figure S4. The expression signatures and maintenance impairment of *Cst3*-KD SSCs.

(**A**) Relative expression of *Cst3* and *Lc3a* measured by q-PCR in WT and shNC-, sh*Cst3* #1- and sh*Cst3* #2-transfected mSSCs. NS, not significant; *$P < 0.05$; **$P < 0.01$.

(**B**) Relative expression of *Atg3*, *Atg5*, *Atg7* and *Rps2* measured by q-PCR in WT and shNC-, sh*Cst3* #1- and sh*Cst3* #2-transfected mSSCs.

(**C**) Western blotting analysis of the protein levels of two types of Lc3a/b in shNC- and sh*Cst3* #2-trasnfected mSSCs treated with different concentrations of 3-MA for 24 h (left). Bar plot showing the grayscale intensity analysis of two types of Lc3a/b, with β-actin as the internal control (right). NS, not significant; *$P < 0.05$; **$P < 0.01$; ****$P < 0.0001$.

(**D**) Cell proliferation curve determined by CCK-8 assay of WT and shNC- and sh*Cst3* #2-transfected SSCs. *$P < 0.05$; **$P < 0.01$.

(**E**) Flow cytometry analysis of the cell cycle in WT and shNC- and sh*Cst3* #2-transfected mSSCs through propidium iodide (PI) staining.

(**F**) Dot plots showing the percentage of each cell phase of WT and shNC- and sh*Cst3* #2-transfected mSSCs. NS, not significant; ***$P < 0.001$.

(**G**) Relative expression of *Zbtb16*, *Gfra1* and *Ddx4* measured by q-PCR in WT and shNC-, sh*Cst3* #1- and sh*Cst3* #2-transfected mSSCs.

(**H**) Western blotting analysis showing the protein levels of Zbtb16 and Ddx4 in WT and shNC-, sh*Cst3* #1- and sh*Cst3* #2-transfected mSSCs.

(**I**) Heatmap of genes related to embryonic stem cell (ESC) and SSC maintenance. The color key from blue to red indicates low to high gene expression levels, respectively.

(**J**) Relative expression of *Oct4, Id1* and *Nanos3* measured by q-PCR in shNC-, sh*Cst3* #2-transfected mSSCs and sh*Cst3* #2-transfected SSCs treated with 3-MA. NS, not significant; *$P < 0.05$; **$P < 0.01$; ****$P < 0.0001$.

(**K**) Western blotting analysis of the protein levels of Oct4 and Nanos3 in shNC- and sh*Cst3* #2-transfected SSCs treated with 3-MA (left). Bar plot showing the grayscale intensity analysis of Oct4 and Nanos3, with β-actin as the inner control (right). NS, not significant; *$P < 0.05$; **$P < 0.01$; ***$P < 0.\ 001$.

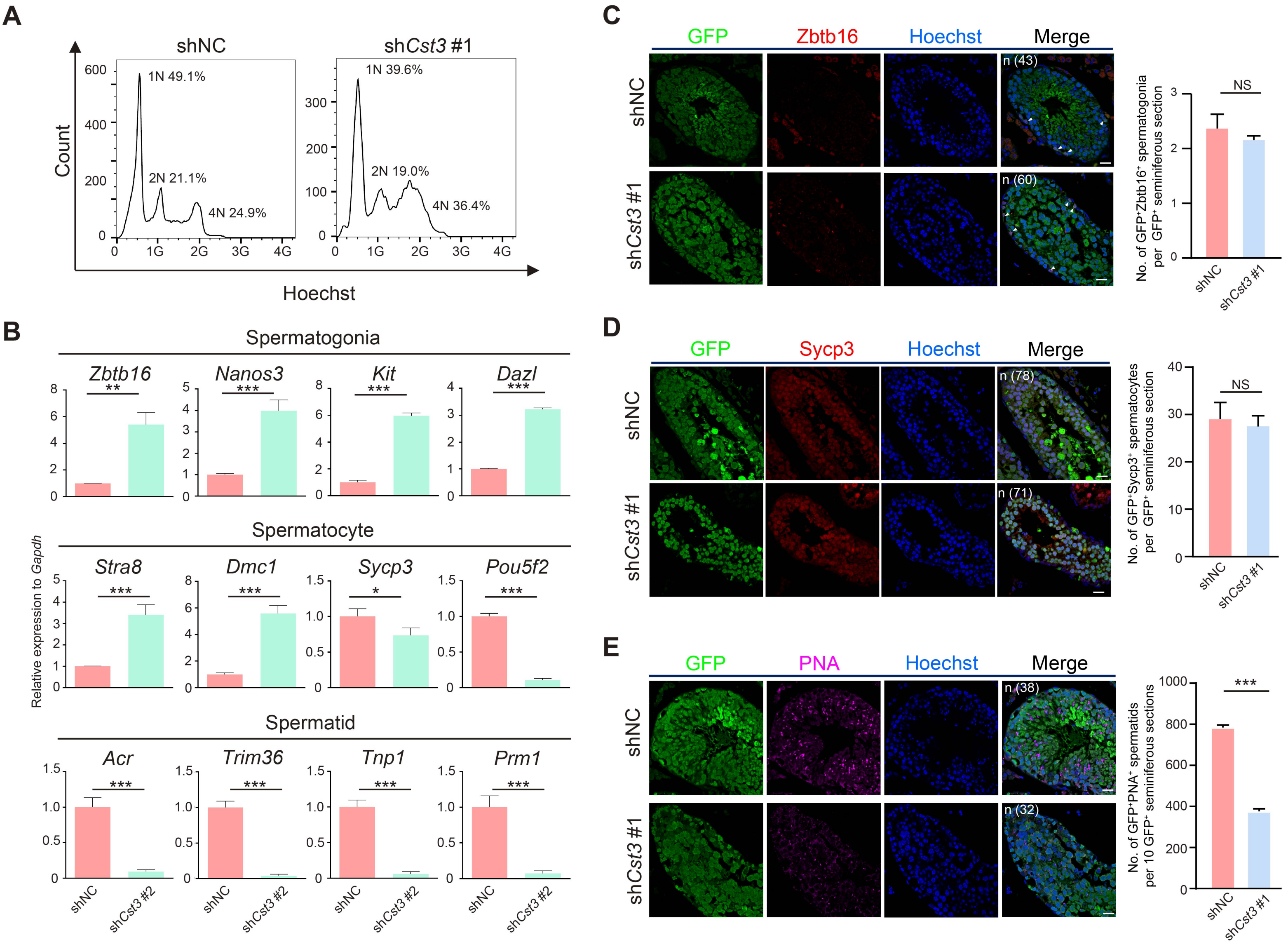
A
shNC
shCst3 #1
1N 49.1%
2N 21.1%
4N 24.9%
1N 39.6%
2N 19.0%
4N 36.4%
Count
Hoechst
B
Spermatogonia
Zbtb16
Nanos3
Kit
Dazl
Spermatocyte
Stra8
Dmc1
Sycp3
Pou5f2
Spermatid
Acr
Trim36
Tnp1
Prm1
Relative expression to Gapdh
shNC
shCst3 #2
C
GFP
Zbtb16
Hoechst
Merge
n (43)
n (60)
NS
No. of GFP+Zbtb16+ spermatogonia per GFP+ seminiferous section
D
Sycp3
n (78)
n (71)
No. of GFP+Sycp3+ spermatocytes per GFP+ seminiferous section
E
PNA
n (38)
n (32)
No. of GFP+PNA+ spermatids per 10 GFP+ seminiferous sections

Figure S5. Confirmation of the involvement of Cst3 in male meiosis in sh*Cst3* #1-transfected SSCs.

(**A**) Flow cytometry analysis of the distribution of each ploidy population among the sorted GFP^+ cells after testicular transplantation with shNC- and sh*Cst3* #1-transfected mSSCs labeled with GFP.

(**B**) Relative expression of specific marker genes by q-PCR in the sorted GFP^+ cells after testicular transplantation with shNC- and shCst3 #2-transfected SSCs labeled with GFP. *$P < 0.05$; **$P < 0.01$; ***$P < 0.001$.

(**C**) Immunofluorescence of GFP (green) and Zbtb16 (red) in testicular paraffin sections transplanted with shNC- and sh*Cst3* #1-transfected mSSCs (left). 'n' represents the number of testicular sections calculated in this panel. Triangles indicate double positive SSCs. The scale bars represent 20 μm. Boxplot showing the number of GFP^+PNA^+ spermatogonia per GFP^+ seminiferous section, NS, not significant (right).

(**D**) Immunofluorescence of GFP (green) and Sycp3 (red) in testicular paraffin sections transplanted with shNC- and sh*Cst3* #1-transfected mSSCs (left). 'n' represents the number of testicular sections calculated in this panel. The scale bars represent 20 μm. Boxplot showing the number of GFP^+Sycp3^+ spermatocytes per GFP^+ seminiferous section, NS, not significant (right).

(**E**) Immunofluorescence of GFP (green) and PNA (pink) in testicular paraffin sections transplanted with shNC- and sh*Cst3* #1-transfected mSSCs (left). 'n' represents the number of testicular sections calculated in this panel. The scale bars represent 20 μm. Boxplot showing the number of GFP^+PNA^+ spermatids per 10 GFP^+ seminiferous sections, ***$P < 0.001$ (right).

Table S1. 1,411 human autophagy-related genes collected from Human Autophagy Database, The Autophagy Database, THANATOS and Human Autophagy Modulators database.

Table S2. 709 mouse autophagy-related genes collected from The Autophagy Database and THANATOS.

Table S3. Differentially expressed autophagy-related genes between two subpopulations of human SSCs.

Table S4. Pearson correlation coefficients (top) and *P*-values (bottom) between gene modules and cell clusters.

Table S5. Gene Ontology Analysis to WGCNA modules of 531 human-mouse homologous autophagy-related genes referring to Figure 1F.

Table S6. Hub genes evaluated by Protein-protein interaction database (PPI) in each module referring to Figure 1F.

Table S7. Differentially expressed homologous autophagy-related genes in human and mouse spermatogenesis among 156 mouse- and 831 human homologous autophagy-related genes, respectively.

Table S8. Cell type identification of 432 testicular cells from NOA1 patient.

Table S9. Differentially expressed genes of NOA1 patient in each testicular cell cluster.

Table S10. Differentially expressed homologous autophagy-related genes in human and mouse spermatogenesis among 531 human-mouse homologous autophagy-related genes.

Table S11. Oligonucleotide primer sequences used in q-PCR.

Supplemental references 1. References related to Table S1.

Supplemental references 2. References related to Table S2.
