# Supplementary material for: Deciphering the autophagy regulatory network via single-cell transcriptome analysis reveals a requirement for autophagy homeostasis in spermatogenesis
Source: Theranostics. 2021 Mar 5;11(10):5010–27. doi: 10.7150/thno.55645 (PMC7978313; doi:10.7150/thno.55645)
Supplement: Supplementary file 2 — Supplementary references 1. [file thnov11p5010s2.pdf]

Supplemental references

1. Polson HE, de Lartigue J, Rigden DJ, et el. Mammalian Atg18 (WIPI2) localizes to omegasome-anchored phagophores and positively regulates LC3 lipidation. Autophagy. 2010 May;6(4):506-22.

2. Moussay E, Kaoma T, Baginska J, et el. The acquisition of resistance to TNFα in breast cancer cells is associated with constitutive activation of autophagy as revealed by a transcriptome analysis using a custom microarray. Autophagy. 2011 Jul;7(7):760-70.

3. Tsuyuki S, Takabayashi M, Kawazu M, et el. Detection of WIPI1 mRNA as an indicator of autophagosome formation. Autophagy. 2014 Mar;10(3):497-513.

4. Kraft C, Peter M, Hofmann K. Selective autophagy: ubiquitin-mediated recognition and beyond. Nat Cell Biol. 2010 Sep;12(9):836-41.

5. Ropolo A, Grasso D, Pardo R, et el. The pancreatitis-induced vacuole membrane protein 1 triggers autophagy in mammalian cells. J Biol Chem. 2007 Dec 21;282(51):37124-33.

6. Fader CM, Sánchez DG, Mestre MB, et el. TI-VAMP/VAMP7 and VAMP3/cellubrevin: two v-SNARE proteins involved in specific steps of the autophagy/multivesicular body pathways. Biochim Biophys Acta. 2009 Dec;1793(12):1901-16.

7. Sir D, Ou JH. Autophagy in viral replication and pathogenesis. Mol Cells. 2010 Jan;29(1):1-7.

8. Maiuri MC, Criollo A, Kroemer G. Crosstalk between apoptosis and autophagy within the Beclin 1 interactome. EMBO J. 2010 Feb 3;29(3):515-6.

9. Verfaillie T, Salazar M, Velasco G, et el. Linking ER Stress to Autophagy: Potential Implications for Cancer Therapy. Int J Cell Biol. 2010;2010:930509.

10. Balaburski GM, Hontz RD, Murphy ME. p53 and ARF: unexpected players in autophagy. Trends Cell Biol. 2010 Jun;20(6):363-9.

11. Funderburk SF, Wang QJ, Yue Z. The Beclin 1-VPS34 complex--at the crossroads of autophagy and beyond. Trends Cell Biol. 2010 Jun;20(6):355-62.

12. He S, Ni D, Ma B, et el. PtdIns(3)P-bound UVRAG coordinates Golgi-ER retrograde and Atg9 transport by differential interactions with the ER tether and the beclin 1 complex. Nat Cell Biol. 2013 Oct;15(10):1206-1219.

13. Young AR, Narita M, Ferreira M, et el. Autophagy mediates the mitotic senescence transition. Genes Dev. 2009 Apr 1;23(7):798-803.

14. Hara T, Takamura A, Kishi C, et el. FIP200, a ULK-interacting protein, is required for autophagosome formation in mammalian cells. J Cell Biol. 2008 May 5;181(3):497-510.

15. Chan EY, Longatti A, McKnight NC, et el. Kinase-inactivated ULK proteins inhibit autophagy via their conserved C-terminal domains using an Atg13-independent mechanism. Mol Cell Biol. 2009 Jan;29(1):157-71.

16. Cheong H, Lindsten T, Wu J, et el. Ammonia-induced autophagy is independent of ULK1/ULK2 kinases. Proc Natl Acad Sci U S A. 2011 Jul 5;108(27):11121-6.

17. Shukla S, Patric IR, Patil V, et el. Methylation silencing of ULK2, an autophagy gene, is essential for astrocyte transformation and tumor growth. J Biol Chem. 2014 Aug 8;289(32):22306-18.

18. Chan EY, Kir S, Tooze SA. siRNA screening of the kinome identifies ULK1 as a multidomain modulator of autophagy. J Biol Chem. 2007 Aug 31;282(35):25464-74.

19. Kundu M, Lindsten T, Yang CY, et el. Ulk1 plays a critical role in the autophagic clearance of mitochondria and ribosomes during reticulocyte maturation. Blood. 2008 Aug 15;112(4):1493-502.

20. Ganley IG, Lam du H, Wang J, et el. ULK1.ATG13.FIP200 complex mediates mTOR signaling and is essential for autophagy. J Biol Chem. 2009 May 1;284(18):12297-305.

21. Renna M, Jimenez-Sanchez M, Sarkar S, et el. Chemical inducers of autophagy that enhance the clearance of mutant proteins in neurodegenerative diseases. J Biol Chem. 2010 Apr 9;285(15):11061-7.

22. Kim J, Kundu M, Viollet B, et el. AMPK and mTOR regulate autophagy through direct phosphorylation of Ulk1. Nat Cell Biol. 2011 Feb;13(2):132-41.

23. Shang L, Chen S, Du F, et el. Nutrient starvation elicits an acute autophagic response mediated by Ulk1 dephosphorylation and its subsequent dissociation from AMPK. Proc Natl Acad Sci U S A. 2011 Mar 22;108(12):4788-93.

24. Joo JH, Dorsey FC, Joshi A, et el. Hsp90-Cdc37 chaperone complex regulates Ulk1- and Atg13-mediated mitophagy. Mol Cell. 2011 Aug 19;43(4):572-85.

25. McKnight NC, Jefferies HB, Alemu EA, et el. Genome-wide siRNA screen reveals amino acid starvation-induced autophagy requires SCOC and WAC. EMBO J. 2012 Apr 18;31(8):1931-46.

26. Rubinsztein DC, Codogno P, Levine B. Autophagy modulation as a potential therapeutic target for diverse diseases. Nat Rev Drug Discov. 2012 Sep;11(9):709-30.

27. Aguado C, Sarkar S, Korolchuk VI, et el. Laforin, the most common protein mutated in Lafora disease, regulates autophagy. Hum Mol Genet. 2010 Jul 15;19(14):2867-76.

28. Cook KL, Shajahan AN, Wärri A, et el. Glucose-regulated protein 78 controls cross-talk between apoptosis and autophagy to determine antiestrogen responsiveness. Cancer Res. 2012 Jul 1;72(13):3337-49.

29. Chen N, Debnath J. IκB kinase complex (IKK) triggers detachment-induced autophagy in mammary epithelial cells independently of the PI3K-AKT-MTORC1 pathway. Autophagy. 2013 Aug;9(8):1214-27.

30. Tripathi DN, Chowdhury R, Trudel LJ, et el. Reactive nitrogen species regulate autophagy through ATM-AMPK-TSC2-mediated suppression of mTORC1. Proc Natl Acad Sci U S A. 2013 Aug 6;110(32):E2950-7.

31. Di Nardo A, Wertz MH, Kwiatkowski E, et el. Neuronal Tsc1/2 complex controls autophagy through AMPK-dependent regulation of ULK1. Hum Mol Genet. 2014 Jul 15;23(14):3865-74.

32. Tang G, Gudsnuk K, Kuo SH, et el. Loss of mTOR-dependent macroautophagy causes autistic-like synaptic pruning deficits. Neuron. 2014 Sep 3;83(5):1131-43.

33. Papadakis M, Hadley G, Xilouri M, et el. Tsc1 (hamartin) confers neuroprotection against ischemia by inducing autophagy. Nat Med. 2013 Mar;19(3):351-7.

34. Sala D, Ivanova S, Plana N, et el. Autophagy-regulating TP53INP2 mediates muscle wasting and is repressed in diabetes. J Clin Invest. 2014 May;124(5):1914-27.

35. Yu C, Wang L, Lv B, et el. TMEM74, a lysosome and autophagosome protein, regulates autophagy. Biochem Biophys Res Commun. 2008 May 2;369(2):622-9.

36. He P, Peng Z, Luo Y, et el. High-throughput functional screening for autophagy-related genes and identification of TM9SF1 as an autophagosome-inducing gene. Autophagy. 2009 Jan;5(1):52-60.

37. Pilli M, Arko-Mensah J, Ponpuak M, et el. TBK-1 promotes autophagy-mediated antimicrobial defense by controlling autophagosome maturation. Immunity. 2012 Aug 24;37(2):223-34.

38. Moore AS, Holzbaur EL. Dynamic recruitment and activation of ALS-associated TBK1 with its target optineurin are required for efficient mitophagy. Proc Natl Acad Sci U S A. 2016 Jun 14;113(24):E3349-58.

39. Webber JL, Tooze SA. Coordinated regulation of autophagy by p38alpha MAPK through

mAtg9 and p38IP. EMBO J. 2010 Jan 6;29(1):27-40.

40. Huang Y, Ratovitski EA. Phospho-ΔNp63α/Rpn13-dependent regulation of LKB1 degradation modulates autophagy in cancer cells. Aging (Albany NY). 2010 Dec;2(12):959-68.

41. Pankiv S, Clausen TH, Lamark T, et el. p62/SQSTM1 binds directly to Atg8/LC3 to facilitate degradation of ubiquitinated protein aggregates by autophagy. J Biol Chem. 2007 Aug 17;282(33):24131-45.

42. Zheng YT, Shahnazari S, Brech A, et el. The adaptor protein p62/SQSTM1 targets invading bacteria to the autophagy pathway. J Immunol. 2009 Nov 1;183(9):5909-16.

43. Wang M, Ye R, Barron E, et el. Essential role of the unfolded protein response regulator GRP78/BiP in protection from neuronal apoptosis. Cell Death Differ. 2010 Mar;17(3):488-98.

44. Clausen TH, Lamark T, Isakson P, et el. p62/SQSTM1 and ALFY interact to facilitate the formation of p62 bodies/ALIS and their degradation by autophagy. Autophagy. 2010 Apr;6(3):330-44.

45. Ma B, Liu B, Cao W, et el. The Wnt Signaling Antagonist Dapper1 Accelerates Dishevelled2 Degradation via Promoting Its Ubiquitination and Aggregate-induced Autophagy. J Biol Chem. 2015 May 8;290(19):12346-54.

46. Lee IH, Cao L, Mostoslavsky R, et el. A role for the NAD-dependent deacetylase Sirt1 in the regulation of autophagy. Proc Natl Acad Sci U S A. 2008 Mar 4;105(9):3374-9.

47. Steeves MA, Dorsey FC, Cleveland JL. Targeting the autophagy pathway for cancer chemoprevention. Curr Opin Cell Biol. 2010 Apr;22(2):218-25.

48. Zhang Z, Lowry SF, Guarente L, et el. Roles of SIRT1 in the acute and restorative phases following induction of inflammation. J Biol Chem. 2010 Dec 31;285(53):41391-401.

49. Powell MJ, Casimiro MC, Cordon-Cardo C, et el. Disruption of a Sirt1-dependent autophagy checkpoint in the prostate results in prostatic intraepithelial neoplasia lesion formation. Cancer Res. 2011 Feb 1;71(3):964-75.

50. Malik SA, Orhon I, Morselli E, et el. BH3 mimetics activate multiple pro-autophagic pathways. Oncogene. 2011 Sep 15;30(37):3918-29.

51. Owczarczyk AB, Schaller MA, Reed M, et el. Sirtuin 1 Regulates Dendritic Cell Activation and Autophagy during Respiratory Syncytial Virus-Induced Immune Responses. J Immunol. 2015 Aug 15;195(4):1637-46.

52. Takahashi Y, Coppola D, Matsushita N, et el. Bif-1 interacts with Beclin 1 through UVRAG and regulates autophagy and tumorigenesis. Nat Cell Biol. 2007 Oct;9(10):1142-51.

53. Yang J, Takahashi Y, Cheng E, et el. GSK-3beta promotes cell survival by modulating Bif-1-dependent autophagy and cell death. J Cell Sci. 2010 Mar 15;123(Pt 6):861-70.

54. Takahashi Y, Hori T, Cooper TK, et el. Bif-1 haploinsufficiency promotes chromosomal instability and accelerates Myc-driven lymphomagenesis via suppression of mitophagy. Blood. 2013 Feb 28;121(9):1622-32.

55. Lee JH, Budanov AV, Talukdar S, et el. Maintenance of metabolic homeostasis by Sestrin2 and Sestrin3. Cell Metab. 2012 Sep 5;16(3):311-21.

56. Bae SH, Sung SH, Oh SY, et el. Sestrins activate Nrf2 by promoting p62-dependent autophagic degradation of Keap1 and prevent oxidative liver damage. Cell Metab. 2013 Jan 8;17(1):73-84.

57. Ishihara M, Urushido M, Hamada K, et el. Sestrin-2 and BNIP3 regulate autophagy and mitophagy in renal tubular cells in acute kidney injury. Am J Physiol Renal Physiol. 2013 Aug 15;305(4):F495-509.

58. Matsunaga K, Saitoh T, Tabata K, et el. Two Beclin 1-binding proteins, Atg14L and Rubicon, reciprocally regulate autophagy at different stages. Nat Cell Biol. 2009 Apr;11(4):385-96.

59. Nyfeler B, Bergman P, Triantafellow E, et el. Relieving autophagy and 4EBP1 from rapamycin resistance. Mol Cell Biol. 2011 Jul;31(14):2867-76.

60. Gleason CE, Lu D, Witters LA, et el. The role of AMPK and mTOR in nutrient sensing in pancreatic beta-cells. J Biol Chem. 2007 Apr 6;282(14):10341-51.

61. Armour SM, Baur JA, Hsieh SN, et el. Inhibition of mammalian S6 kinase by resveratrol suppresses autophagy. Aging (Albany NY). 2009 Jun 3;1(6):515-28.

62. Vingtdeux V, Chandakkar P, Zhao H, et el. Novel synthetic small-molecule activators of AMPK as enhancers of autophagy and amyloid-β peptide degradation. FASEB J. 2011 Jan;25(1):219-31.

63. Lee JH, Tecedor L, Chen YH, et el. Reinstating aberrant mTORC1 activity in Huntington's disease mice improves disease phenotypes. Neuron. 2015 Jan 21;85(2):303-15.

64. De Vries L, Zheng B, Fischer T, et el. The regulator of G protein signaling family. Annu

Rev Pharmacol Toxicol. 2000;40:235-71.

65. Pattingre S, Bauvy C, Codogno P. Amino acids interfere with the ERK1/2-dependent control of macroautophagy by controlling the activation of Raf-1 in human colon cancer HT-29 cells. J Biol Chem. 2003 May 9;278(19):16667-74.

66. Ceteci F, Xu J, Ceteci S, et el. Conditional expression of oncogenic C-RAF in mouse pulmonary epithelial cells reveals differential tumorigenesis and induction of autophagy leading to tumor regression. Neoplasia. 2011 Nov;13(11):1005-18.

67. Collins CA, Brown EJ. Cytosol as battleground: ubiquitin as a weapon for both host and pathogen. Trends Cell Biol. 2010 Apr;20(4):205-13.

68. Noda T, Yoshimori T. Between canonical and antibacterial autophagy: Rab7 is required for GAS-containing autophagosome-like vacuole formation. Autophagy. 2010 Apr;6(3):419-20.

69. Yang X, Guan Y, Tian S, et el. Mechanical and IL-1β Responsive miR-365 Contributes to Osteoarthritis Development by Targeting Histone Deacetylase 4. Int J Mol Sci. 2016 Mar 23;17(4):436.

70. Chandra M, Saran R, Datta S. Deciphering the role of Atg5 in nucleotide dependent interaction of Rab33B with the dimeric complex, Atg5-Atg16L1. Biochem Biophys Res Commun. 2016 Apr 22;473(1):8-16.

71. Arico S, Petiot A, Bauvy C, et el. The tumor suppressor PTEN positively regulates macroautophagy by inhibiting the phosphatidylinositol 3-kinase/protein kinase B pathway. J Biol Chem. 2001 Sep 21;276(38):35243-6.

72. Narendra D, Tanaka A, Suen DF, et el. Parkin is recruited selectively to impaired mitochondria and promotes their autophagy. J Cell Biol. 2008 Dec 1;183(5):795-803.

73. Lee JY, Nagano Y, Taylor JP, et el. Disease-causing mutations in parkin impair mitochondrial ubiquitination, aggregation, and HDAC6-dependent mitophagy. J Cell Biol. 2010 May 17;189(4):671-9.

74. Chen D, Gao F, Li B, et el. Parkin mono-ubiquitinates Bcl-2 and regulates autophagy. J Biol Chem. 2010 Dec 3;285(49):38214-23.

75. Kubli DA, Zhang X, Lee Y, et el. Parkin protein deficiency exacerbates cardiac injury and reduces survival following myocardial infarction. J Biol Chem. 2013 Jan 11;288(2):915-26.

76. Burchell VS, Nelson DE, Sanchez-Martinez A, et el. The Parkinson's disease-linked

proteins Fbxo7 and Parkin interact to mediate mitophagy. Nat Neurosci. 2013 Sep;16(9):1257-65.

77. Manzanillo PS, Ayres JS, Watson RO, et el. The ubiquitin ligase parkin mediates resistance to intracellular pathogens. Nature. 2013 Sep 26;501(7468):512-6.

78. Bingol B, Tea JS, Phu L, et el. The mitochondrial deubiquitinase USP30 opposes parkin-mediated mitophagy. Nature. 2014 Jun 19;510(7505):370-5.

79. Grenier K, Kontogiannea M, Fon EA. Short mitochondrial ARF triggers Parkin/PINK1-dependent mitophagy. J Biol Chem. 2014 Oct 24;289(43):29519-30.

80. Cunningham CN, Baughman JM, Phu L, et el. USP30 and parkin homeostatically regulate atypical ubiquitin chains on mitochondria. Nat Cell Biol. 2015 Feb;17(2):160-9.

81. Williams JA, Ni HM, Haynes A, et el. Chronic Deletion and Acute Knockdown of Parkin Have Differential Responses to Acetaminophen-induced Mitophagy and Liver Injury in Mice. J Biol Chem. 2015 Apr 24;290(17):10934-46.

82. Nezich CL, Wang C, Fogel AI, et el. MiT/TFE transcription factors are activated during mitophagy downstream of Parkin and Atg5. J Cell Biol. 2015 Aug 3;210(3):435-50.

83. Zhou ZD, Xie SP, Sathiyamoorthy S, et el. F-box protein 7 mutations promote protein aggregation in mitochondria and inhibit mitophagy. Hum Mol Genet. 2015 Nov 15;24(22):6314-30.

84. Shahnazari S, Yen WL, Birmingham CL, et el. A diacylglycerol-dependent signaling pathway contributes to regulation of antibacterial autophagy. Cell Host Microbe. 2010 Aug 19;8(2):137-46.

85. Dagda RK, Cherra SJ 3rd, Kulich SM, et el. Loss of PINK1 function promotes mitophagy through effects on oxidative stress and mitochondrial fission. J Biol Chem. 2009 May 15;284(20):13843-55.

86. Michiorri S, Gelmetti V, Giarda E, et el. The Parkinson-associated protein PINK1 interacts with Beclin1 and promotes autophagy. Cell Death Differ. 2010 Jun;17(6):962-74.

87. Geisler S, Holmström KM, Treis A, et el. The PINK1/Parkin-mediated mitophagy is compromised by PD-associated mutations. Autophagy. 2010 Oct;6(7):871-8.

88. Gegg ME, Cooper JM, Chau KY, et el. Mitofusin 1 and mitofusin 2 are ubiquitinated in a PINK1/parkin-dependent manner upon induction of mitophagy. Hum Mol Genet. 2010 Dec 15;19(24):4861-70.

89. Rao VA, Klein SR, Bonar SJ, et el. The antioxidant transcription factor Nrf2 negatively regulates autophagy and growth arrest induced by the anticancer redox agent mitoquinone. J Biol Chem. 2010 Nov 5;285(45):34447-59.

90. Gan L, Vargas MR, Johnson DA, et el. Astrocyte-specific overexpression of Nrf2 delays motor pathology and synuclein aggregation throughout the CNS in the alpha-synuclein mutant (A53T) mouse model. J Neurosci. 2012 Dec 5;32(49):17775-87.

91. Xie CM, Wei D, Zhao L, et el. Erbin is a novel substrate of the Sag-βTrCP E3 ligase that regulates KrasG12D-induced skin tumorigenesis. J Cell Biol. 2015 Jun 8;209(5):721-37.

92. Kirkin V, Lamark T, Sou YS, et el. A role for NBR1 in autophagosomal degradation of ubiquitinated substrates. Mol Cell. 2009 Feb 27;33(4):505-16.

93. Wu S, Mikhailov A, Kallo-Hosein H, et el. Characterization of ubiquilin 1, an mTOR-interacting protein. Biochim Biophys Acta. 2002 Jan 30;1542(1-3):41-56.

94. Yuan J, Lipinski M, Degterev A. Diversity in the mechanisms of neuronal cell death. Neuron. 2003 Oct 9;40(2):401-13.

95. Tanaka M, Machida Y, Nukina N. A novel therapeutic strategy for polyglutamine diseases by stabilizing aggregation-prone proteins with small molecules. J Mol Med (Berl). 2005 May;83(5):343-52.

96. Cao Y, Espinola JA, Fossale E, et el. Autophagy is disrupted in a knock-in mouse model of juvenile neuronal ceroid lipofuscinosis. J Biol Chem. 2006 Jul 21;281(29):20483-93.

97. Slow EJ, Graham RK, Hayden MR. To be or not to be toxic: aggregations in Huntington and Alzheimer disease. Trends Genet. 2006 Aug;22(8):408-11.

98. Ravikumar B, Rubinsztein DC. Role of autophagy in the clearance of mutant huntingtin: a step towards therapy. Mol Aspects Med. 2006 Oct-Dec;27(5-6):520-7.

99. Wang LH, Qin ZH. Animal models of Huntington's disease: implications in uncovering pathogenic mechanisms and developing therapies. Acta Pharmacol Sin. 2006 Oct;27(10):1287-302.

100. Rubinsztein DC. The roles of intracellular protein-degradation pathways in neurodegeneration. Nature. 2006 Oct 19;443(7113):780-6.

101. Truant R, Atwal R, Burtnik A. Hypothesis: Huntingtin may function in membrane association and vesicular trafficking. Biochem Cell Biol. 2006 Dec;84(6):912-7.

102. Rochet JC. Novel therapeutic strategies for the treatment of protein-misfolding diseases.

Expert Rev Mol Med. 2007 Jun 28;9(17):1-34.

103. Young JE, Martinez RA, La Spada AR. Nutrient deprivation induces neuronal autophagy and implicates reduced insulin signaling in neuroprotective autophagy activation. J Biol Chem. 2009 Jan 23;284(4):2363-73.

104. Zeng X, Kinsella TJ. BNIP3 is essential for mediating 6-thioguanine- and 5-fluorouracil-induced autophagy following DNA mismatch repair processing. Cell Res. 2010 Jun;20(6):665-75.

105. Yu L, McPhee CK, Zheng L, et el. Termination of autophagy and reformation of lysosomes regulated by mTOR. Nature. 2010 Jun 17;465(7300):942-6.

106. Huang S, Yang ZJ, Yu C, et el. Inhibition of mTOR kinase by AZD8055 can antagonize chemotherapy-induced cell death through autophagy induction and down-regulation of p62/sequestosome 1. J Biol Chem. 2011 Nov 18;286(46):40002-12.

107. Bachar-Wikstrom E, Wikstrom JD, Ariav Y, et el. Stimulation of autophagy improves endoplasmic reticulum stress-induced diabetes. Diabetes. 2013 Apr;62(4):1227-37.

108. Seldin MM, Lei X, Tan SY, et el. Skeletal muscle-derived myonectin activates the mammalian target of rapamycin (mTOR) pathway to suppress autophagy in liver. J Biol Chem. 2013 Dec 13;288(50):36073-82.

109. Hou X, Hu Z, Xu H, et el. Advanced glycation endproducts trigger autophagy in cadiomyocyte via RAGE/PI3K/AKT/mTOR pathway. Cardiovasc Diabetol. 2014 Apr 14;13:78.

110. Caccamo A, De Pinto V, Messina A, et el. Genetic reduction of mammalian target of rapamycin ameliorates Alzheimer's disease-like cognitive and pathological deficits by restoring hippocampal gene expression signature. J Neurosci. 2014 Jun 4;34(23):7988-98.

111. Zhang X, Howell GM, Guo L, et el. CaMKIV-dependent preservation of mTOR expression is required for autophagy during lipopolysaccharide-induced inflammation and acute kidney injury. J Immunol. 2014 Sep 1;193(5):2405-15.

112. Dowdle WE, Nyfeler B, Nagel J, et el. Selective VPS34 inhibitor blocks autophagy and uncovers a role for NCOA4 in ferritin degradation and iron homeostasis in vivo. Nat Cell Biol. 2014 Nov;16(11):1069-79.

113. Yu X, Long YC. Autophagy modulates amino acid signaling network in myotubes: differential effects on mTORC1 pathway and the integrated stress response. FASEB J. 2015 Feb;29(2):394-407.

114. Marchand B, Arsenault D, Raymond-Fleury A, et el. Glycogen synthase kinase-3 (GSK3) inhibition induces prosurvival autophagic signals in human pancreatic cancer cells. J Biol Chem. 2015 Feb 27;290(9):5592-605.

115. Colletti RB, Baldassano RN, Milov DE, et el. Variation in care in pediatric Crohn disease. J Pediatr Gastroenterol Nutr. 2009 Sep;49(3):297-303.

116. Fu MM, Holzbaur EL. MAPK8IP1/JIP1 regulates the trafficking of autophagosomes in neurons. Autophagy. 2014;10(11):2079-81.

117. Pattingre S, Bauvy C, Carpentier S, et el. Role of JNK1-dependent Bcl-2 phosphorylation in ceramide-induced macroautophagy. J Biol Chem. 2009 Jan 30;284(5):2719-28.

118. Shimizu S, Konishi A, Nishida Y, et el. Involvement of JNK in the regulation of autophagic cell death. Oncogene. 2010 Apr 8;29(14):2070-82.

119. Kim MJ, Woo SJ, Yoon CH, et el. Involvement of autophagy in oncogenic K-Ras-induced malignant cell transformation. J Biol Chem. 2011 Apr 15;286(15):12924-32.

120. Yoo BH, Khan IA, Koomson A, et el. Oncogenic RAS-induced downregulation of ATG12 is required for survival of malignant intestinal epithelial cells. Autophagy. 2018;14(1):134-151.

121. Kumar GS, Clarkson MW, Kunze MBA, et el. Dynamic activation and regulation of the mitogen-activated protein kinase p38. Proc Natl Acad Sci U S A. 2018 May 1;115(18):4655-4660.

122. Le Guerroué F, Eck F, Jung J, et el. Autophagosomal Content Profiling Reveals an LC3C-Dependent Piecemeal Mitophagy Pathway. Mol Cell. 2017 Nov 16;68(4):786-796.e6.

123. Köchl R, Hu XW, Chan EY, et el. Microtubules facilitate autophagosome formation and fusion of autophagosomes with endosomes. Traffic. 2006 Feb;7(2):129-45.

124. Andrade EC, Krueger DD, Nairn AC. Recent advances in neuroproteomics. Curr Opin Mol Ther. 2007 Jun;9(3):270-81.

125. Zhou Z, Jiang X, Liu D, et el. Autophagy is involved in influenza A virus replication. Autophagy. 2009 Apr;5(3):321-8.

126. Liu L, Feng D, Chen G, et el. Mitochondrial outer-membrane protein FUNDC1 mediates hypoxia-induced mitophagy in mammalian cells. Nat Cell Biol. 2012 Jan 22;14(2):177-85.

127. Cuervo AM, Dice JF. Unique properties of lamp2a compared to other lamp2 isoforms. J Cell Sci. 2000 Dec;113 Pt 24:4441-50.

128. Eskelinen EL, Illert AL, Tanaka Y, et el. Role of LAMP-2 in lysosome biogenesis and autophagy. Mol Biol Cell. 2002 Sep;13(9):3355-68.

129. Hayashi H, Sano H, Seo S, et el. The Foxc2 transcription factor regulates angiogenesis via induction of integrin beta3 expression. J Biol Chem. 2008 Aug 29;283(35):23791-800.

130. Bandyopadhyay U, Kaushik S, Varticovski L, et el. The chaperone-mediated autophagy receptor organizes in dynamic protein complexes at the lysosomal membrane. Mol Cell Biol. 2008 Sep;28(18):5747-63.

131. Li P, Ji M, Lu F, et el. Degradation of AF1Q by chaperone-mediated autophagy. Exp Cell Res. 2014 Sep 10;327(1):48-56.

132. Hubert V, Peschel A, Langer B, et el. LAMP-2 is required for incorporating syntaxin-17 into autophagosomes and for their fusion with lysosomes. Biol Open. 2016 Oct 15;5(10):1516-1529.

133. Parys JB, Decuypere JP, Bultynck G. Role of the inositol 1,4,5-trisphosphate receptor/Ca2+-release channel in autophagy. Cell Commun Signal. 2012 Jul 6;10(1):17.

134. Singh SB, Ornatowski W, Vergne I, et el. Human IRGM regulates autophagy and cell-autonomous immunity functions through mitochondria. Nat Cell Biol. 2010 Dec;12(12):1154-65.

135. Orenstein SJ, Cuervo AM. Chaperone-mediated autophagy: molecular mechanisms and physiological relevance. Semin Cell Dev Biol. 2010 Sep;21(7):719-26.

136. Macri C, Wang F, Tasset I, et el. Modulation of deregulated chaperone-mediated autophagy by a phosphopeptide. Autophagy. 2015;11(3):472-86.

137. Zhang H, Bosch-Marce M, Shimoda LA, et el. Mitochondrial autophagy is an HIF-1-dependent adaptive metabolic response to hypoxia. J Biol Chem. 2008 Apr 18;283(16):10892-903.

138. Bellot G, Garcia-Medina R, Gounon P, et el. Hypoxia-induced autophagy is mediated through hypoxia-inducible factor induction of BNIP3 and BNIP3L via their BH3 domains. Mol Cell Biol. 2009 May;29(10):2570-81.

139. Li X, Fan Z. The epidermal growth factor receptor antibody cetuximab induces autophagy in cancer cells by downregulating HIF-1alpha and Bcl-2 and activating the beclin 1/hVps34 complex. Cancer Res. 2010 Jul 15;70(14):5942-52.

140. Iwata A, Riley BE, Johnston JA, et el. HDAC6 and microtubules are required for autophagic degradation of aggregated huntingtin. J Biol Chem. 2005 Dec 2;280(48):40282-92.

141. Lee JY, Koga H, Kawaguchi Y, et el. HDAC6 controls autophagosome maturation essential for ubiquitin-selective quality-control autophagy. EMBO J. 2010 Mar 3;29(5):969-80.

142. Lopes da Fonseca T, Outeiro TF. ATP13A2 and Alpha-synuclein: a Metal Taste in Autophagy. Exp Neurobiol. 2014 Dec;23(4):314-23.

143. Yue Z, Horton A, Bravin M, et el. A novel protein complex linking the delta 2 glutamate receptor and autophagy: implications for neurodegeneration in lurcher mice. Neuron. 2002 Aug 29;35(5):921-33.

144. Hassel B, Schreff M, Stube EM, et el. CALEB/NGC interacts with the Golgi-associated protein PIST. J Biol Chem. 2003 Oct 10;278(41):40136-43.

145. Nakajima H, Itakura M, Kubo T, et el. Glyceraldehyde-3-phosphate Dehydrogenase (GAPDH) Aggregation Causes Mitochondrial Dysfunction during Oxidative Stress-induced Cell Death. J Biol Chem. 2017 Mar 17;292(11):4727-4742.

146. Weidberg H, Shvets E, Shpilka T, et el. LC3 and GATE-16/GABARAP subfamilies are both essential yet act differently in autophagosome biogenesis. EMBO J. 2010 Jun 2;29(11):1792-802.

147. Ding WX, Ni HM, Gao W, et el. Linking of autophagy to ubiquitin-proteasome system is important for the regulation of endoplasmic reticulum stress and cell viability. Am J Pathol. 2007 Aug;171(2):513-24.

148. Cremer TJ, Amer A, Tridandapani S, et el. Francisella tularensis regulates autophagy-related host cell signaling pathways. Autophagy. 2009 Jan;5(1):125-8.

149. Mammucari C, Milan G, Romanello V, et el. FoxO3 controls autophagy in skeletal muscle in vivo. Cell Metab. 2007 Dec;6(6):458-71.

150. Balakumaran BS, Porrello A, Hsu DS, et el. MYC activity mitigates response to rapamycin in prostate cancer through eukaryotic initiation factor 4E-binding protein 1-mediated inhibition of autophagy. Cancer Res. 2009 Oct 1;69(19):7803-10.

151. Tallóczy Z, Jiang W, Virgin HW 4th, et el. Regulation of starvation- and virus-induced autophagy by the eIF2alpha kinase signaling pathway. Proc Natl Acad Sci U S A. 2002 Jan 8;99(1):190-5.

152. Park MA, Zhang G, Martin AP, et el. Vorinostat and sorafenib increase ER stress, autophagy and apoptosis via ceramide-dependent CD95 and PERK activation. Cancer Biol Ther. 2008 Oct;7(10):1648-62.

153. Avivar-Valderas A, Salas E, Bobrovnikova-Marjon E, et el. PERK integrates autophagy and oxidative stress responses to promote survival during extracellular matrix detachment. Mol Cell Biol. 2011 Sep;31(17):3616-29.

154. Siddiqui MA, Malathi K. RNase L induces autophagy via c-Jun N-terminal kinase and double-stranded RNA-dependent protein kinase signaling pathways. J Biol Chem. 2012 Dec 21;287(52):43651-64.

155. Yang C, Shogren KL, Goyal R, et el. RNA-dependent protein kinase is essential for 2-methoxyestradiol-induced autophagy in osteosarcoma cells. PLoS One. 2013;8(3):e59406.

156. Tan X, Thapa N, Sun Y, et el. A kinase-independent role for EGF receptor in autophagy initiation. Cell. 2015 Jan 15;160(1-2):145-60.

157. Qiu J, Li X, He Y, et el. Distinct subgroup of the Ras family member 3 (DIRAS3) expression impairs metastasis and induces autophagy of gastric cancer cells in mice. J Cancer Res Clin Oncol. 2018 Oct;144(10):1869-1886.

158. Mahmood DF, Jguirim-Souissi I, Khadija el-H, et el. Peroxisome proliferator-activated receptor gamma induces apoptosis and inhibits autophagy of human monocyte-derived macrophages via induction of cathepsin L: potential role in atherosclerosis. J Biol Chem. 2011 Aug 19;286(33):28858-66.

159. Jiang H, Martin V, Gomez-Manzano C, et el. The RB-E2F1 pathway regulates autophagy. Cancer Res. 2010 Oct 15;70(20):7882-93.

160. Komata T, Kanzawa T, Takeuchi H, et el. Antitumour effect of cyclin-dependent kinase inhibitors (p16(INK4A), p18(INK4C), p19(INK4D), p21(WAF1/CIP1) and p27(KIP1)) on malignant glioma cells. Br J Cancer. 2003 Apr 22;88(8):1277-80.

161. Sun X, Momen A, Wu J, et el. p27 protein protects metabolically stressed cardiomyocytes from apoptosis by promoting autophagy. J Biol Chem. 2014 Jun 13;289(24):16924-35.

162. Demarchi F, Bertoli C, Copetti T, et el. Calpain is required for macroautophagy in mammalian cells. J Cell Biol. 2006 Nov 20;175(4):595-605.

163. Smith MA, Covington MD, Schnellmann RG. Loss of calpain 10 causes mitochondrial dysfunction during chronic hyperglycemia. Arch Biochem Biophys. 2012 Jul

15;523(2):161-8.

164. Xia HG, Zhang L, Chen G, et el. Control of basal autophagy by calpain1 mediated cleavage of ATG5. Autophagy. 2010 Jan;6(1):61-6.

165. Lipinski MM, Zheng B, Lu T, et el. Genome-wide analysis reveals mechanisms modulating autophagy in normal brain aging and in Alzheimer's disease. Proc Natl Acad Sci U S A. 2010 Aug 10;107(32):14164-9.

166. Shi W, Xu D, Gu J, et el. Saikosaponin-d inhibits proliferation by up-regulating autophagy via the CaMKKβ-AMPK-mTOR pathway in ADPKD cells. Mol Cell Biochem. 2018 Dec;449(1-2):219-226.

167. Thurston TL, Ryzhakov G, Bloor S, et el. The TBK1 adaptor and autophagy receptor NDP52 restricts the proliferation of ubiquitin-coated bacteria. Nat Immunol. 2009 Nov;10(11):1215-21.

168. Proskuryakov SY, Konoplyannikov AG, Gabai VL. Necrosis: a specific form of programmed cell death. Exp Cell Res. 2003 Feb 1;283(1):1-16.

169. Daido S, Kanzawa T, Yamamoto A, et el. Pivotal role of the cell death factor BNIP3 in ceramide-induced autophagic cell death in malignant glioma cells. Cancer Res. 2004 Jun 15;64(12):4286-93.

170. Kanzawa T, Zhang L, Xiao L, et el. Arsenic trioxide induces autophagic cell death in malignant glioma cells by upregulation of mitochondrial cell death protein BNIP3. Oncogene. 2005 Feb 3;24(6):980-91.

171. Azad MB, Chen Y, Henson ES, et el. Hypoxia induces autophagic cell death in apoptosis-competent cells through a mechanism involving BNIP3. Autophagy. 2008 Feb;4(2):195-204.

172. Galluzzi L, Joza N, Tasdemir E, et el. No death without life: vital functions of apoptotic effectors. Cell Death Differ. 2008 Jul;15(7):1113-23.

173. Byun YJ, Kim SK, Kim YM, et el. Hydrogen peroxide induces autophagic cell death in C6 glioma cells via BNIP3-mediated suppression of the mTOR pathway. Neurosci Lett. 2009 Sep 18;461(2):131-5.

174. Quinsay MN, Thomas RL, Lee Y, et el. Bnip3-mediated mitochondrial autophagy is independent of the mitochondrial permeability transition pore. Autophagy. 2010 Oct;6(7):855-62.

175. Hanna RA, Quinsay MN, Orogo AM, et el. Microtubule-associated protein 1 light chain 3

(LC3) interacts with Bnip3 protein to selectively remove endoplasmic reticulum and mitochondria via autophagy. J Biol Chem. 2012 Jun 1;287(23):19094-104.

176. Liang XH, Yu J, Brown K, et el. Beclin 1 contains a leucine-rich nuclear export signal that is required for its autophagy and tumor suppressor function. Cancer Res. 2001 Apr 15;61(8):3443-9.

177. Qu X, Yu J, Bhagat G, et el. Promotion of tumorigenesis by heterozygous disruption of the beclin 1 autophagy gene. J Clin Invest. 2003 Dec;112(12):1809-20.

178. Yue Z, Jin S, Yang C, et el. Beclin 1, an autophagy gene essential for early embryonic development, is a haploinsufficient tumor suppressor. Proc Natl Acad Sci U S A. 2003 Dec 9;100(25):15077-82.

179. Zeng X, Overmeyer JH, Maltese WA. Functional specificity of the mammalian Beclin-Vps34 PI 3-kinase complex in macroautophagy versus endocytosis and lysosomal enzyme trafficking. J Cell Sci. 2006 Jan 15;119(Pt 2):259-70.

180. Valentim L, Laurence KM, Townsend PA, et el. Urocortin inhibits Beclin1-mediated autophagic cell death in cardiac myocytes exposed to ischaemia/reperfusion injury. J Mol Cell Cardiol. 2006 Jun;40(6):846-52.

181. Zhu JH, Horbinski C, Guo F, et el. Regulation of autophagy by extracellular signal-regulated protein kinases during 1-methyl-4-phenylpyridinium-induced cell death. Am J Pathol. 2007 Jan;170(1):75-86.

182. Nguyen TM, Subramanian IV, Kelekar A, et el. Kringle 5 of human plasminogen, an angiogenesis inhibitor, induces both autophagy and apoptotic death in endothelial cells. Blood. 2007 Jun 1;109(11):4793-802.

183. Matsui Y, Takagi H, Qu X, et el. Distinct roles of autophagy in the heart during ischemia and reperfusion: roles of AMP-activated protein kinase and Beclin 1 in mediating autophagy. Circ Res. 2007 Mar 30;100(6):914-22.

184. Pacheco CD, Kunkel R, Lieberman AP. Autophagy in Niemann-Pick C disease is dependent upon Beclin-1 and responsive to lipid trafficking defects. Hum Mol Genet. 2007 Jun 15;16(12):1495-503.

185. Fimia GM, Stoykova A, Romagnoli A, et el. Ambra1 regulates autophagy and development of the nervous system. Nature. 2007 Jun 28;447(7148):1121-5.

186. Park KJ, Lee SH, Kim TI, et el. A human scFv antibody against TRAIL receptor 2 induces autophagic cell death in both TRAIL-sensitive and TRAIL-resistant cancer cells.

Cancer Res. 2007 Aug 1;67(15):7327-34.

187. Fujiwara K, Daido S, Yamamoto A, et el. Pivotal role of the cyclin-dependent kinase inhibitor p21WAF1/CIP1 in apoptosis and autophagy. J Biol Chem. 2008 Jan 4;283(1):388-97.

188. Pickford F, Masliah E, Britschgi M, et el. The autophagy-related protein beclin 1 shows reduced expression in early Alzheimer disease and regulates amyloid beta accumulation in mice. J Clin Invest. 2008 Jun;118(6):2190-9.

189. Wang J, Lian H, Zhao Y, et el. Vitamin D3 induces autophagy of human myeloid leukemia cells. J Biol Chem. 2008 Sep 12;283(37):25596-605.

190. Shi CS, Kehrl JH. MyD88 and Trif target Beclin 1 to trigger autophagy in macrophages. J Biol Chem. 2008 Nov 28;283(48):33175-82.

191. Luo S, Rubinsztein DC. Apoptosis blocks Beclin 1-dependent autophagosome synthesis: an effect rescued by Bcl-xL. Cell Death Differ. 2010 Feb;17(2):268-77.

192. Yoo BH, Wu X, Li Y, et el. Oncogenic ras-induced down-regulation of autophagy mediator Beclin-1 is required for malignant transformation of intestinal epithelial cells. J Biol Chem. 2010 Feb 19;285(8):5438-49.

193. Chang NC, Nguyen M, Germain M, et el. Antagonism of Beclin 1-dependent autophagy by BCL-2 at the endoplasmic reticulum requires NAF-1. EMBO J. 2010 Feb 3;29(3):606-18.

194. Walls KC, Ghosh AP, Franklin AV, et el. Lysosome dysfunction triggers Atg7-dependent neural apoptosis. J Biol Chem. 2010 Apr 2;285(14):10497-507.

195. Gao P, Bauvy C, Souquère S, et el. The Bcl-2 homology domain 3 mimetic gossypol induces both Beclin 1-dependent and Beclin 1-independent cytoprotective autophagy in cancer cells. J Biol Chem. 2010 Aug 13;285(33):25570-81.

196. Oh S, Xiaofei E, Ni D, et el. Downregulation of autophagy by Bcl-2 promotes MCF7 breast cancer cell growth independent of its inhibition of apoptosis. Cell Death Differ. 2011 Mar;18(3):452-64.

197. Li H, Wang P, Sun Q, et el. Following cytochrome c release, autophagy is inhibited during chemotherapy-induced apoptosis by caspase 8-mediated cleavage of Beclin 1. Cancer Res. 2011 May 15;71(10):3625-34.

198. Campbell GR, Spector SA. Hormonally active vitamin D3 (1alpha,25-dihydroxycholecalciferol) triggers autophagy in human macrophages that

inhibits HIV-1 infection. J Biol Chem. 2011 May 27;286(21):18890-902.

199. Tu SP, Quante M, Bhagat G, et el. IFN-γ inhibits gastric carcinogenesis by inducing epithelial cell autophagy and T-cell apoptosis. Cancer Res. 2011 Jun 15;71(12):4247-59.

200. Luo S, Garcia-Arencibia M, Zhao R, et el. Bim inhibits autophagy by recruiting Beclin 1 to microtubules. Mol Cell. 2012 Aug 10;47(3):359-70.

201. Margariti A, Li H, Chen T, et el. XBP1 mRNA splicing triggers an autophagic response in endothelial cells through BECLIN-1 transcriptional activation. J Biol Chem. 2013 Jan 11;288(2):859-72.

202. Pan W, Zhong Y, Cheng C, et el. MiR-30-regulated autophagy mediates angiotensin II-induced myocardial hypertrophy. PLoS One. 2013;8(1):e53950.

203. Kim J, Kim YC, Fang C, et el. Differential regulation of distinct Vps34 complexes by AMPK in nutrient stress and autophagy. Cell. 2013 Jan 17;152(1-2):290-303.

204. Shoji-Kawata S, Sumpter R, Leveno M, et el. Identification of a candidate therapeutic autophagy-inducing peptide. Nature. 2013 Feb 14;494(7436):201-6.

205. He C, Wei Y, Sun K, et el. Beclin 2 functions in autophagy, degradation of G protein-coupled receptors, and metabolism. Cell. 2013 Aug 29;154(5):1085-1099.

206. Su M, Mei Y, Sanishvili R, et el. Targeting γ-herpesvirus 68 Bcl-2-mediated down-regulation of autophagy. J Biol Chem. 2014 Mar 21;289(12):8029-40.

207. Ginet V, Spiehlmann A, Rummel C, et el. Involvement of autophagy in hypoxic-excitotoxic neuronal death. Autophagy. 2014 May;10(5):846-60.

208. Qi Y, Zhang M, Li H, et el. Autophagy inhibition by sustained overproduction of IL6 contributes to arsenic carcinogenesis. Cancer Res. 2014 Jul 15;74(14):3740-52.

209. Ye LX, Yu J, Liang YX, et el. Beclin 1 knockdown retards re-endothelialization and exacerbates neointimal formation via a crosstalk between autophagy and apoptosis. Atherosclerosis. 2014 Nov;237(1):146-54.

210. He M, Ding Y, Chu C, et el. Autophagy induction stabilizes microtubules and promotes axon regeneration after spinal cord injury. Proc Natl Acad Sci U S A. 2016 Oct 4;113(40):11324-11329.

211. Priault M, Hue E, Marhuenda F, et el. Differential dependence on Beclin 1 for the regulation of pro-survival autophagy by Bcl-2 and Bcl-xL in HCT116 colorectal cancer cells. PLoS One. 2010 Jan 18;5(1):e8755.

212. Bhutia SK, Dash R, Das SK, et el. Mechanism of autophagy to apoptosis switch triggered in prostate cancer cells by antitumor cytokine melanoma differentiation-associated gene 7/interleukin-24. Cancer Res. 2010 May 1;70(9):3667-76.

213. Zhu Y, Massen S, Terenzio M, et el. Modulation of serines 17 and 24 in the LC3-interacting region of Bnip3 determines pro-survival mitophagy versus apoptosis. J Biol Chem. 2013 Jan 11;288(2):1099-113.

214. Hollville E, Carroll RG, Cullen SP, et el. Bcl-2 family proteins participate in mitochondrial quality control by regulating Parkin/PINK1-dependent mitophagy. Mol Cell. 2014 Aug 7;55(3):451-66.

215. Wu H, Xue D, Chen G, et el. The BCL2L1 and PGAM5 axis defines hypoxia-induced receptor-mediated mitophagy. Autophagy. 2014 Oct 1;10(10):1712-25.

216. Casalino-Matsuda SM, Nair A, Beitel GJ, et el. Hypercapnia Inhibits Autophagy and Bacterial Killing in Human Macrophages by Increasing Expression of Bcl-2 and Bcl-xL. J Immunol. 2015 Jun 1;194(11):5388-96.

217. He C, Bassik MC, Moresi V, et el. Exercise-induced BCL2-regulated autophagy is required for muscle glucose homeostasis. Nature. 2012 Jan 18;481(7382):511-5.

218. Ulbricht A, Eppler FJ, Tapia VE, et el. Cellular mechanotransduction relies on tension-induced and chaperone-assisted autophagy. Curr Biol. 2013 Mar 4;23(5):430-5.

219. Yamada T, Carson AR, Caniggia I, et el. Endothelial nitric-oxide synthase antisense (NOS3AS) gene encodes an autophagy-related protein (APG9-like2) highly expressed in trophoblast. J Biol Chem. 2005 May 6;280(18):18283-90.

220. Saitoh T, Fujita N, Hayashi T, et el. Atg9a controls dsDNA-driven dynamic translocation of STING and the innate immune response. Proc Natl Acad Sci U S A. 2009 Dec 8;106(49):20842-6.

221. Komatsu M, Waguri S, Ueno T, et el. Impairment of starvation-induced and constitutive autophagy in Atg7-deficient mice. J Cell Biol. 2005 May 9;169(3):425-34.

222. Lavieu G, Scarlatti F, Sala G, et el. Regulation of autophagy by sphingosine kinase 1 and its role in cell survival during nutrient starvation. J Biol Chem. 2006 Mar 31;281(13):8518-27.

223. Wang QJ, Ding Y, Kohtz DS, et el. Induction of autophagy in axonal dystrophy and degeneration. J Neurosci. 2006 Aug 2;26(31):8057-68.

224. Nakai A, Yamaguchi O, Takeda T, et el. The role of autophagy in cardiomyocytes in the

basal state and in response to hemodynamic stress. Nat Med. 2007 May;13(5):619-24.

225. Komatsu M, Wang QJ, Holstein GR, et el. Essential role for autophagy protein Atg7 in the maintenance of axonal homeostasis and the prevention of axonal degeneration. Proc Natl Acad Sci U S A. 2007 Sep 4;104(36):14489-94.

226. Wan G, Zhaorigetu S, Liu Z, et el. Apolipoprotein L1, a novel Bcl-2 homology domain 3-only lipid-binding protein, induces autophagic cell death. J Biol Chem. 2008 Aug 1;283(31):21540-9.

227. Zhang Y, Goldman S, Baerga R, et el. Adipose-specific deletion of autophagy-related gene 7 (atg7) in mice reveals a role in adipogenesis. Proc Natl Acad Sci U S A. 2009 Nov 24;106(47):19860-5.

228. Cooney R, Baker J, Brain O, et el. NOD2 stimulation induces autophagy in dendritic cells influencing bacterial handling and antigen presentation. Nat Med. 2010 Jan;16(1):90-7.

229. Mortensen M, Ferguson DJ, Edelmann M, et el. Loss of autophagy in erythroid cells leads to defective removal of mitochondria and severe anemia in vivo. Proc Natl Acad Sci U S A. 2010 Jan 12;107(2):832-7.

230. Zhao Y, Yang J, Liao W, et el. Cytosolic FoxO1 is essential for the induction of autophagy and tumour suppressor activity. Nat Cell Biol. 2010 Jul;12(7):665-75.

231. Mariño G, Fernández AF, Cabrera S, et el. Autophagy is essential for mouse sense of balance. J Clin Invest. 2010 Jul;120(7):2331-44.

232. Lock R, Roy S, Kenific CM, et el. Autophagy facilitates glycolysis during Ras-mediated oncogenic transformation. Mol Biol Cell. 2011 Jan 15;22(2):165-78.

233. Shen S, Kepp O, Michaud M, et el. Association and dissociation of autophagy, apoptosis and necrosis by systematic chemical study. Oncogene. 2011 Nov 10;30(45):4544-56.

234. Cristancho AG, Lazar MA. Forming functional fat: a growing understanding of adipocyte differentiation. Nat Rev Mol Cell Biol. 2011 Sep 28;12(11):722-34.

235. Bejarano E, Girao H, Yuste A, et el. Autophagy modulates dynamics of connexins at the plasma membrane in a ubiquitin-dependent manner. Mol Biol Cell. 2012 Jun;23(11):2156-69.

236. Shehata M, Matsumura H, Okubo-Suzuki R, et el. Neuronal stimulation induces autophagy in hippocampal neurons that is involved in AMPA receptor degradation after chemical long-term depression. J Neurosci. 2012 Jul 25;32(30):10413-22.

237. Kikuchi M, Kuroki S, Kayama M, et el. Protease activity of procaspase-8 is essential for cell survival by inhibiting both apoptotic and nonapoptotic cell death dependent on receptor-interacting protein kinase 1 (RIP1) and RIP3. J Biol Chem. 2012 Nov 30;287(49):41165-73.

238. Onal M, Piemontese M, Xiong J, et el. Suppression of autophagy in osteocytes mimics skeletal aging. J Biol Chem. 2013 Jun 14;288(24):17432-40.

239. Belaid A, Cerezo M, Chargui A, et el. Autophagy plays a critical role in the degradation of active RHOA, the control of cell cytokinesis, and genomic stability. Cancer Res. 2013 Jul 15;73(14):4311-22.

240. Bartolomé A, López-Herradón A, Portal-Núñez S, et el. Autophagy impairment aggravates the inhibitory effects of high glucose on osteoblast viability and function. Biochem J. 2013 Nov 1;455(3):329-37.

241. Li S, Du L, Zhang L, et el. Cathepsin B contributes to autophagy-related 7 (Atg7)-induced nod-like receptor 3 (NLRP3)-dependent proinflammatory response and aggravates lipotoxicity in rat insulinoma cell line. J Biol Chem. 2013 Oct 18;288(42):30094-104.

242. Bonilla DL, Bhattacharya A, Sha Y, et el. Autophagy regulates phagocytosis by modulating the expression of scavenger receptors. Immunity. 2013 Sep 19;39(3):537-47.

243. Gonzalez Y, Aryal B, Chehab L, et el. Atg7- and Keap1-dependent autophagy protects breast cancer cell lines against mitoquinone-induced oxidative stress. Oncotarget. 2014 Mar 30;5(6):1526-37.

244. Teng YC, Jeng CJ, Huang HJ, et el. Role of autophagy in arsenite-induced neurotoxicity: the involvement of α-synuclein. Toxicol Lett. 2015 Mar 18;233(3):239-45.

245. Cao Y, Zhang S, Yuan N, et el. Hierarchal Autophagic Divergence of Hematopoietic System. J Biol Chem. 2015 Sep 18;290(38):23050-63.

246. Eng CH, Wang Z, Tkach D, et el. Macroautophagy is dispensable for growth of KRAS mutant tumors and chloroquine efficacy. Proc Natl Acad Sci U S A. 2016 Jan 5;113(1):182-7.

247. Mizushima N, Yamamoto A, Hatano M, et el. Dissection of autophagosome formation using Apg5-deficient mouse embryonic stem cells. J Cell Biol. 2001 Feb 19;152(4):657-68.

248. Pyo JO, Jang MH, Kwon YK, et el. Essential roles of Atg5 and FADD in autophagic cell

death: dissection of autophagic cell death into vacuole formation and cell death. J Biol Chem. 2005 May 27;280(21):20722-9.

249. Wang Y, Singh R, Massey AC, et el. Loss of macroautophagy promotes or prevents fibroblast apoptosis depending on the death stimulus. J Biol Chem. 2008 Feb 22;283(8):4766-77.

250. Kong DK, Georgescu SP, Cano C, et el. Deficiency of the transcriptional regulator p8 results in increased autophagy and apoptosis, and causes impaired heart function. Mol Biol Cell. 2010 Apr 15;21(8):1335-49.

251. Chang YP, Tsai CC, Huang WC, et el. Autophagy facilitates IFN-gamma-induced Jak2-STAT1 activation and cellular inflammation. J Biol Chem. 2010 Sep 10;285(37):28715-22.

252. Yang PM, Liu YL, Lin YC, et el. Inhibition of autophagy enhances anticancer effects of atorvastatin in digestive malignancies. Cancer Res. 2010 Oct 1;70(19):7699-709.

253. Qi HY, Daniels MP, Liu Y, et el. A cytosolic phospholipase A2-initiated lipid mediator pathway induces autophagy in macrophages. J Immunol. 2011 Nov 15;187(10):5286-92.

254. Lu Y, Hao BX, Graeff R, et el. Two pore channel 2 (TPC2) inhibits autophagosomal-lysosomal fusion by alkalinizing lysosomal pH. J Biol Chem. 2013 Aug 16;288(33):24247-63.

255. Shi CS, Qi HY, Boularan C, et el. SARS-coronavirus open reading frame-9b suppresses innate immunity by targeting mitochondria and the MAVS/TRAF3/TRAF6 signalosome. J Immunol. 2014 Sep 15;193(6):3080-9.

256. Mariño G, Uría JA, Puente XS, et el. Human autophagins, a family of cysteine proteinases potentially implicated in cell degradation by autophagy. J Biol Chem. 2003 Feb 7;278(6):3671-8.

257. Mariño G, Salvador-Montoliu N, Fueyo A, et el. Tissue-specific autophagy alterations and increased tumorigenesis in mice deficient in Atg4C/autophagin-3. J Biol Chem. 2007 Jun 22;282(25):18573-83.

258. Ketteler R, Sun Z, Kovacs KF, et el. A pathway sensor for genome-wide screens of intracellular proteolytic cleavage. Genome Biol. 2008 Apr 3;9(4):R64.

259. Sou YS, Waguri S, Iwata J, et el. The Atg8 conjugation system is indispensable for proper development of autophagic isolation membranes in mice. Mol Biol Cell. 2008 Nov;19(11):4762-75.

260. Radoshevich L, Murrow L, Chen N, et el. ATG12 conjugation to ATG3 regulates mitochondrial homeostasis and cell death. Cell. 2010 Aug 20;142(4):590-600.

261. Velikkakath AK, Nishimura T, Oita E, et el. Mammalian Atg2 proteins are essential for autophagosome formation and important for regulation of size and distribution of lipid droplets. Mol Biol Cell. 2012 Mar;23(5):896-909.

262. Saitoh T, Fujita N, Jang MH, et el. Loss of the autophagy protein Atg16L1 enhances endotoxin-induced IL-1beta production. Nature. 2008 Nov 13;456(7219):264-8.

263. Zhong Y, Wang QJ, Li X, et el. Distinct regulation of autophagic activity by Atg14L and Rubicon associated with Beclin 1-phosphatidylinositol-3-kinase complex. Nat Cell Biol. 2009 Apr;11(4):468-76.

264. Matsunaga K, Morita E, Saitoh T, et el. Autophagy requires endoplasmic reticulum targeting of the PI3-kinase complex via Atg14L. J Cell Biol. 2010 Aug 23;190(4):511-21.

265. Fogel AI, Dlouhy BJ, Wang C, et el. Role of membrane association and Atg14-dependent phosphorylation in beclin-1-mediated autophagy. Mol Cell Biol. 2013 Sep;33(18):3675-88.

266. Tanida I, Tanida-Miyake E, Nishitani T, et el. Murine Apg12p has a substrate preference for murine Apg7p over three Apg8p homologs. Biochem Biophys Res Commun. 2002 Mar 22;292(1):256-62.

267. Liu CL, Chen S, Dietrich D, et el. Changes in autophagy after traumatic brain injury. J Cereb Blood Flow Metab. 2008 Apr;28(4):674-83.

268. Boya P, González-Polo RA, Casares N, et el. Inhibition of macroautophagy triggers apoptosis. Mol Cell Biol. 2005 Feb;25(3):1025-40.

269. Cho YY, Kim DJ, Lee HS, et el. Autophagy and cellular senescence mediated by Sox2 suppress malignancy of cancer cells. PLoS One. 2013;8(2):e57172.

270. Shi C, Wu J, Fu M, et el. Ambra1 modulates starvation-induced autophagy through AMPK signaling pathway in cardiomyocytes. Biochem Biophys Res Commun. 2014 Sep 26;452(3):308-14.

271. Jiang P, Nishimura T, Sakamaki Y, et el. The HOPS complex mediates autophagosome-lysosome fusion through interaction with syntaxin 17. Mol Biol Cell. 2014 Apr;25(8):1327-37.

272. Zhen Y, Li W. Impairment of autophagosome-lysosome fusion in the buff mutant mice with the VPS33A(D251E) mutation. Autophagy. 2015;11(9):1608-22.

273. Ozturk DG, Kocak M, Akcay A, et el. MITF-MIR211 axis is a novel autophagy amplifier system during cellular stress. Autophagy. 2018 Oct 5:1-16.

274. Wartosch L, Günesdogan U, Graham SC, et el. Recruitment of VPS33A to HOPS by VPS16 Is Required for Lysosome Fusion with Endosomes and Autophagosomes. Traffic. 2015 Jul;16(7):727-42.

275. Fu R, Deng Q, Zhang H, et el. A novel autophagy inhibitor berbamine blocks SNARE-mediated autophagosome-lysosome fusion through upregulation of BNIP3. Cell Death Dis. 2018 Feb 14;9(2):243.

276. Haddad DM, Vilain S, Vos M, et el. Mutations in the intellectual disability gene Ube2a cause neuronal dysfunction and impair parkin-dependent mitophagy. Mol Cell. 2013 Jun 27;50(6):831-43.

277. Zong M, Wu XG, Chan CW, et el. The adaptor function of TRAPPC2 in mammalian TRAPPs explains TRAPPC2-associated SEDT and TRAPPC9-associated congenital intellectual disability. PLoS One. 2011;6(8):e23350.

278. Min Y, Kim MJ, Lee S, et el. Inhibition of TRAF6 ubiquitin-ligase activity by PRDX1 leads to inhibition of NFKB activation and autophagy activation. Autophagy. 2018;14(8):1347-1358.

279. Pu J, Wu S, Xie H, et el. miR-146a Inhibits dengue-virus-induced autophagy by targeting TRAF6. Arch Virol. 2017 Dec;162(12):3645-3659.

280. Chauhan S, Goodwin JG, Chauhan S, et el. ZKSCAN3 is a master transcriptional repressor of autophagy. Mol Cell. 2013 Apr 11;50(1):16-28.

281. Ogawa M, Yoshikawa Y, Kobayashi T, et el. A Tecpr1-dependent selective autophagy pathway targets bacterial pathogens. Cell Host Microbe. 2011 May 19;9(5):376-89.

282. Chen D, Fan W, Lu Y, et el. A mammalian autophagosome maturation mechanism mediated by TECPR1 and the Atg12-Atg5 conjugate. Mol Cell. 2012 Mar 9;45(5):629-41.

283. Popovic D, Akutsu M, Novak I, et el. Rab GTPase-activating proteins in autophagy: regulation of endocytic and autophagy pathways by direct binding to human ATG8 modifiers. Mol Cell Biol. 2012 May;32(9):1733-44.

284. Popovic D, Dikic I. TBC1D5 and the AP2 complex regulate ATG9 trafficking and initiation of autophagy. EMBO Rep. 2014 Apr;15(4):392-401.

285. Hirano S, Uemura T, Annoh H, et el. Differing susceptibility to autophagic degradation of

two LC3-binding proteins: SQSTM1/p62 and TBC1D25/OATL1. Autophagy. 2016;12(2):312-26.

286. Longatti A, Lamb CA, Razi M, et el. TBC1D14 regulates autophagosome formation via Rab11- and ULK1-positive recycling endosomes. J Cell Biol. 2012 May 28;197(5):659-75.

287. Itakura E, Kishi-Itakura C, Mizushima N. The hairpin-type tail-anchored SNARE syntaxin 17 targets to autophagosomes for fusion with endosomes/lysosomes. Cell. 2012 Dec 7;151(6):1256-69.

288. Kim EH, Sohn S, Kwon HJ, et el. Sodium selenite induces superoxide-mediated mitochondrial damage and subsequent autophagic cell death in malignant glioma cells. Cancer Res. 2007 Jul 1;67(13):6314-24.

289. Dodson M, Liu P, Jiang T, et el. Increased O-GlcNAcylation of SNAP29 drives arsenic-induced autophagic dysfunction. Mol Cell Biol. 2018 Mar 5.

290. Taniguchi M, Kitatani K, Kondo T, et el. Regulation of autophagy and its associated cell death by "sphingolipid rheostat": reciprocal role of ceramide and sphingosine 1-phosphate in the mammalian target of rapamycin pathway. J Biol Chem. 2012 Nov 16;287(47):39898-910.

291. Nicklin P, Bergman P, Zhang B, et el. Bidirectional transport of amino acids regulates mTOR and autophagy. Cell. 2009 Feb 6;136(3):521-34.

292. Bowman CJ, Ayer DE, Dynlacht BD. Foxk proteins repress the initiation of starvation-induced atrophy and autophagy programs. Nat Cell Biol. 2014 Dec;16(12):1202-14.

293. Zheng Z, Yang J, Zhao D, et el. Downregulated adaptor protein p66(Shc) mitigates autophagy process by low nutrient and enhances apoptotic resistance in human lung adenocarcinoma A549 cells. FEBS J. 2013 Sep;280(18):4522-30.

294. Polak R, Bierings MB, van der Leije CS, et el. Autophagy inhibition as a potential future targeted therapy for ETV6-RUNX1 driven B-cell precursor acute lymphoblastic leukemia. Haematologica. 2018 Oct 31.

295. Durán RV, Oppliger W, Robitaille AM, et el. Glutaminolysis activates Rag-mTORC1 signaling. Mol Cell. 2012 Aug 10;47(3):349-58.

296. Xu Y, Jagannath C, Liu XD, et el. Toll-like receptor 4 is a sensor for autophagy associated with innate immunity. Immunity. 2007 Jul;27(1):135-44.

297. Duan H, Li Y, Yan L, et el. Rcan1-1L overexpression induces mitochondrial autophagy and improves cell survival in angiotensin II-exposed cardiomyocytes. Exp Cell Res. 2015 Jul 1;335(1):99-106.

298. Simicek M, Lievens S, Laga M, et el. The deubiquitylase USP33 discriminates between RALB functions in autophagy and innate immune response. Nat Cell Biol. 2013 Oct;15(10):1220-30.

299. Patergnani S, Marchi S, Rimessi A, et el. PRKCB/protein kinase C, beta and the mitochondrial axis as key regulators of autophagy. Autophagy. 2013 Sep;9(9):1367-85.

300. Tan SH, Shui G, Zhou J, et el. Induction of autophagy by palmitic acid via protein kinase C-mediated signaling pathway independent of mTOR (mammalian target of rapamycin). J Biol Chem. 2012 Apr 27;287(18):14364-76.

301. Grotemeier A, Alers S, Pfisterer SG, et el. AMPK-independent induction of autophagy by cytosolic Ca2+ increase. Cell Signal. 2010 Jun;22(6):914-25.

302. Qiu SL, Xiao ZC, Piao CM, et el. AMP-activated protein kinase α2 protects against liver injury from metastasized tumors via reduced glucose deprivation-induced oxidative stress. J Biol Chem. 2014 Mar 28;289(13):9449-59.

303. Dunlop EA, Seifan S, Claessens T, et el. FLCN, a novel autophagy component, interacts with GABARAP and is regulated by ULK1 phosphorylation. Autophagy. 2014 Oct 1;10(10):1749-60.

304. Herrero-Martín G, Høyer-Hansen M, García-García C, et el. TAK1 activates AMPK-dependent cytoprotective autophagy in TRAIL-treated epithelial cells. EMBO J. 2009 Mar 18;28(6):677-85.

305. Gómez-Suaga P, Luzón-Toro B, Churamani D, et el. Leucine-rich repeat kinase 2 regulates autophagy through a calcium-dependent pathway involving NAADP. Hum Mol Genet. 2012 Feb 1;21(3):511-25.

306. Sinha RA, Singh BK, Zhou J, et el. Thyroid hormone induction of mitochondrial activity is coupled to mitophagy via ROS-AMPK-ULK1 signaling. Autophagy. 2015;11(8):1341-57.

307. Wong PM, Feng Y, Wang J, et el. Regulation of autophagy by coordinated action of mTORC1 and protein phosphatase 2A. Nat Commun. 2015 Aug 27;6:8048.

308. Fang K, Li HF, Hsieh CH, et el. Differential autophagic cell death under stress with ectopic cytoplasmic and mitochondrial-specific PPP2R2B in human neuroblastoma cells.

Apoptosis. 2013 May;18(5):627-38.

309. Song H, Pu J, Wang L, et el. ATG16L1 phosphorylation is oppositely regulated by CSNK2/casein kinase 2 and PPP1/protein phosphatase 1 which determines the fate of cardiomyocytes during hypoxia/reoxygenation. Autophagy. 2015;11(8):1308-25.

310. Dall'Armi C, Hurtado-Lorenzo A, Tian H, et el. The phospholipase D1 pathway modulates macroautophagy. Nat Commun. 2010;1:142.

311. Noda S, Asano Y, Takahashi T, et el. Decreased cathepsin V expression due to Fli1 deficiency contributes to the development of dermal fibrosis and proliferative vasculopathy in systemic sclerosis. Rheumatology (Oxford). 2013 May;52(5):790-9.

312. Cao Y, Wang Y, Abi Saab WF, et el. NRBF2 regulates macroautophagy as a component of Vps34 Complex I. Biochem J. 2014 Jul 15;461(2):315-22.

313. Lu J, He L, Behrends C, et el. NRBF2 regulates autophagy and prevents liver injury by modulating Atg14L-linked phosphatidylinositol-3 kinase III activity. Nat Commun. 2014 May 22;5:3920.

314. Lahiri A, Hedl M, Abraham C. MTMR3 risk allele enhances innate receptor-induced signaling and cytokines by decreasing autophagy and increasing caspase-1 activation. Proc Natl Acad Sci U S A. 2015 Aug 18;112(33):10461-6.

315. Travassos LH, Carneiro LA, Ramjeet M, et el. Nod1 and Nod2 direct autophagy by recruiting ATG16L1 to the plasma membrane at the site of bacterial entry. Nat Immunol. 2010 Jan;11(1):55-62.

316. Goodwin JM, Dowdle WE, DeJesus R, et el. Autophagy-Independent Lysosomal Targeting Regulated by ULK1/2-FIP200 and ATG9. Cell Rep. 2017 Sep 5;20(10):2341-2356.

317. Hailey DW, Rambold AS, Satpute-Krishnan P, et el. Mitochondria supply membranes for autophagosome biogenesis during starvation. Cell. 2010 May 14;141(4):656-67.

318. Zhao T, Huang X, Han L, et el. Central role of mitofusin 2 in autophagosome-lysosome fusion in cardiomyocytes. J Biol Chem. 2012 Jul 6;287(28):23615-25.

319. Muñoz JP, Ivanova S, Sánchez-Wandelmer J, et el. Mfn2 modulates the UPR and mitochondrial function via repression of PERK. EMBO J. 2013 Aug 28;32(17):2348-61.

320. Goussetis DJ, Altman JK, Glaser H, et el. Autophagy is a critical mechanism for the induction of the antileukemic effects of arsenic trioxide. J Biol Chem. 2010 Sep 24;285(39):29989-97.

321. Kang R, Loux T, Tang D, et el. The expression of the receptor for advanced glycation endproducts (RAGE) is permissive for early pancreatic neoplasia. Proc Natl Acad Sci U S A. 2012 May 1;109(18):7031-6.

322. Dodson M, Redmann M, Rajasekaran NS, et el. KEAP1-NRF2 signalling and autophagy in protection against oxidative and reductive proteotoxicity. Biochem J. 2015 Aug 1;469(3):347-55.

323. Sparks JD, Magra AL, Chamberlain JM, et el. Insulin dependent apolipoprotein B degradation and phosphatidylinositide 3-kinase activation with microsomal translocation are restored in McArdle RH7777 cells following serum deprivation. Biochem Biophys Res Commun. 2016 Jan 8;469(2):326-31.

324. Tomasetti M, Monaco F, Manzella N, et el. MicroRNA-126 induces autophagy by altering cell metabolism in malignant mesothelioma. Oncotarget. 2016 Jun 14;7(24):36338-36352.

325. Liu Y, Shi QF, Ye YC, et el. Activated O2(•−) and H2O2 mediated cell survival in SU11274-treated non-small-cell lung cancer A549 cells via c-Met-PI3K-Akt and c-Met-Grb2/SOS-Ras-p38 pathways. J Pharmacol Sci. 2012;119(2):150-9.

326. Ciechomska IA, Przanowski P, Jackl J, et el. BIX01294, an inhibitor of histone methyltransferase, induces autophagy-dependent differentiation of glioma stem-like cells. Sci Rep. 2016 Dec 9;6:38723.

327. Bodemann BO, Orvedahl A, Cheng T, et el. RalB and the exocyst mediate the cellular starvation response by direct activation of autophagosome assembly. Cell. 2011 Jan 21;144(2):253-67.

328. Zhao H, Zhao YG, Wang X, et el. Mice deficient in Epg5 exhibit selective neuronal vulnerability to degeneration. J Cell Biol. 2013 Mar 18;200(6):731-41.

329. Zhao YG, Zhao H, Sun H, et el. Role of Epg5 in selective neurodegeneration and Vici syndrome. Autophagy. 2013 Aug;9(8):1258-62.

330. Wengrod J, Wang D, Weiss S, et el. Phosphorylation of eIF2α triggered by mTORC1 inhibition and PP6C activation is required for autophagy and is aberrant in PP6C-mutated melanoma. Sci Signal. 2015 Mar 10;8(367):ra27.

331. Wang B, Ling S, Lin WC. 14-3-3Tau regulates Beclin 1 and is required for autophagy. PLoS One. 2010 Apr 29;5(4):e10409.

332. Park SM, Kim K, Lee EJ, et el. Reduced expression of DRAM2/TMEM77 in tumor cells interferes with cell death. Biochem Biophys Res Commun. 2009 Dec 25;390(4):1340-4.

333. Liu X, Rothe K, Yen R, et el. A novel AHI-1-BCR-ABL-DNM2 complex regulates leukemic properties of primitive CML cells through enhanced cellular endocytosis and ROS-mediated autophagy. Leukemia. 2017 Nov;31(11):2376-2387.

334. Kageyama Y, Zhang Z, Roda R, et el. Mitochondrial division ensures the survival of postmitotic neurons by suppressing oxidative damage. J Cell Biol. 2012 May 14;197(4):535-51.

335. Cahill TJ, Leo V, Kelly M, et el. Resistance of Dynamin-related Protein 1 Oligomers to Disassembly Impairs Mitophagy, Resulting in Myocardial Inflammation and Heart Failure. J Biol Chem. 2015 Oct 23;290(43):25907-19.

336. Zhao Y, Xiong X, Sun Y. DEPTOR, an mTOR inhibitor, is a physiological substrate of SCF(βTrCP) E3 ubiquitin ligase and regulates survival and autophagy. Mol Cell. 2011 Oct 21;44(2):304-16.

337. Molitoris JK, McColl KS, Swerdlow S, et el. Glucocorticoid elevation of dexamethasone-induced gene 2 (Dig2/RTP801/REDD1) protein mediates autophagy in lymphocytes. J Biol Chem. 2011 Aug 26;286(34):30181-9.

338. Ravikumar B, Moreau K, Jahreiss L, et el. Plasma membrane contributes to the formation of pre-autophagosomal structures. Nat Cell Biol. 2010 Aug;12(8):747-57.

339. Criollo A, Senovilla L, Authier H, et el. The IKK complex contributes to the induction of autophagy. EMBO J. 2010 Feb 3;29(3):619-31.

340. Menzies FM, Garcia-Arencibia M, Imarisio S, et el. Calpain inhibition mediates autophagy-dependent protection against polyglutamine toxicity. Cell Death Differ. 2015 Mar;22(3):433-44.

341. Nüchel J, Ghatak S, Zuk AV, et el. TGFB1 is secreted through an unconventional pathway dependent on the autophagic machinery and cytoskeletal regulators. Autophagy. 2018;14(3):465-486.

342. Moreau K, Ravikumar B, Puri C, et el. Arf6 promotes autophagosome formation via effects on phosphatidylinositol 4,5-bisphosphate and phospholipase D. J Cell Biol. 2012 Feb 20;196(4):483-96.

343. Keller CW, Loi M, Ewert S, et el. The autophagy machinery restrains iNKT cell activation through CD1D1 internalization. Autophagy. 2017 Jun 3;13(6):1025-1036.

344. Jia S, Wang Y, You Z, et el. Mammalian Atg9 contributes to the post-Golgi transport of lysosomal hydrolases by interacting with adaptor protein-1. FEBS Lett. 2017

Dec;591(24):4027-4038.

345. Takeuchi H, Kondo Y, Fujiwara K, et el. Synergistic augmentation of rapamycin-induced autophagy in malignant glioma cells by phosphatidylinositol 3-kinase/protein kinase B inhibitors. Cancer Res. 2005 Apr 15;65(8):3336-46.

346. Degtyarev M, De Mazière A, Orr C, et el. Akt inhibition promotes autophagy and sensitizes PTEN-null tumors to lysosomotropic agents. J Cell Biol. 2008 Oct 6;183(1):101-16.

347. Cheng Y, Ren X, Zhang Y, et el. eEF-2 kinase dictates cross-talk between autophagy and apoptosis induced by Akt Inhibition, thereby modulating cytotoxicity of novel Akt inhibitor MK-2206. Cancer Res. 2011 Apr 1;71(7):2654-63.

348. Cheng HC, Kim SR, Oo TF, et el. Akt suppresses retrograde degeneration of dopaminergic axons by inhibition of macroautophagy. J Neurosci. 2011 Feb 9;31(6):2125-35.

349. Nazarko TY, Ozeki K, Till A, et el. Peroxisomal Atg37 binds Atg30 or palmitoyl-CoA to regulate phagophore formation during pexophagy. J Cell Biol. 2014 Feb 17;204(4):541-57.

350. Colicelli J. ABL tyrosine kinases: evolution of function, regulation, and specificity. Sci Signal. 2010 Sep 14;3(139):re6.

351. Vicinanza M, Korolchuk VI, Ashkenazi A, et el. PI(5)P regulates autophagosome biogenesis. Mol Cell. 2015 Jan 22;57(2):219-34.

352. Chhipa RR, Wu Y, Ip C. AMPK-mediated autophagy is a survival mechanism in androgen-dependent prostate cancer cells subjected to androgen deprivation and hypoxia. Cell Signal. 2011 Sep;23(9):1466-72.

353. Gu Y, Li P, Peng F, et el. Autophagy-related prognostic signature for breast cancer. Mol Carcinog. 2016 Mar;55(3):292-9.

354. Koike M, Nakanishi H, Saftig P, et el. Cathepsin D deficiency induces lysosomal storage with ceroid lipofuscin in mouse CNS neurons. J Neurosci. 2000 Sep 15;20(18):6898-906.

355. Yin L, Liu J, Dong H, et el. Autophagy-related gene16L2, a potential serum biomarker of multiple sclerosis evaluated by bead-based proteomic technology. Neurosci Lett. 2014 Mar 6;562:34-8.

356. Qiao L, Guo B, Zhang H, et el. The clock gene, brain and muscle Arnt-like 1, regulates autophagy in high glucose-induced cardiomyocyte injury. Oncotarget. 2017 Sep

11;8(46):80612-80624.

357. Nozawa T, Aikawa C, Goda A, et el. The small GTPases Rab9A and Rab23 function at distinct steps in autophagy during Group A Streptococcus infection. Cell Microbiol. 2012 Aug;14(8):1149-65.

358. Bakula D, Müller AJ, Zuleger T, et el. WIPI3 and WIPI4 β-propellers are scaffolds for LKB1-AMPK-TSC signalling circuits in the control of autophagy. Nat Commun. 2017 May 31;8:15637.

359. Saitsu H, Nishimura T, Muramatsu K, et el. De novo mutations in the autophagy gene WDR45 cause static encephalopathy of childhood with neurodegeneration in adulthood. Nat Genet. 2013 Apr;45(4):445-9, 449e1.

360. Crighton D, O'Prey J, Bell HS, et el. p73 regulates DRAM-independent autophagy that does not contribute to programmed cell death. Cell Death Differ. 2007 Jun;14(6):1071-9.

361. Feng Z, Zhang H, Levine AJ, et el. The coordinate regulation of the p53 and mTOR pathways in cells. Proc Natl Acad Sci U S A. 2005 Jun 7;102(23):8204-9.

362. Pimkina J, Humbey O, Zilfou JT, et el. ARF induces autophagy by virtue of interaction with Bcl-xl. J Biol Chem. 2009 Jan 30;284(5):2803-10.

363. Livesey KM, Kang R, Vernon P, et el. p53/HMGB1 complexes regulate autophagy and apoptosis. Cancer Res. 2012 Apr 15;72(8):1996-2005.

364. Kenzelmann Broz D, Spano Mello S, Bieging KT, et el. Global genomic profiling reveals an extensive p53-regulated autophagy program contributing to key p53 responses. Genes Dev. 2013 May 1;27(9):1016-31.

365. Laforge M, Limou S, Harper F, et el. DRAM triggers lysosomal membrane permeabilization and cell death in CD4(+) T cells infected with HIV. PLoS Pathog. 2013;9(5):e1003328.

366. Gomes LR, Vessoni AT, Menck CF. Three-dimensional microenvironment confers enhanced sensitivity to doxorubicin by reducing p53-dependent induction of autophagy. Oncogene. 2015 Oct 16;34(42):5329-40.

367. Mills KR, Reginato M, Debnath J, et el. Tumor necrosis factor-related apoptosis-inducing ligand (TRAIL) is required for induction of autophagy during lumen formation in vitro. Proc Natl Acad Sci U S A. 2004 Mar 9;101(10):3438-43.

368. Thorburn J, Moore F, Rao A, et el. Selective inactivation of a Fas-associated death domain protein (FADD)-dependent apoptosis and autophagy pathway in immortal

epithelial cells. Mol Biol Cell. 2005 Mar;16(3):1189-99.

369. Yanagisawa H, Miyashita T, Nakano Y, et el. HSpin1, a transmembrane protein interacting with Bcl-2/Bcl-xL, induces a caspase-independent autophagic cell death. Cell Death Differ. 2003 Jul;10(7):798-807.

370. Kamimoto T, Shoji S, Hidvegi T, et el. Intracellular inclusions containing mutant alpha1-antitrypsin Z are propagated in the absence of autophagic activity. J Biol Chem. 2006 Feb 17;281(7):4467-76.

371. Smith SE, Granell S, Salcedo-Sicilia L, et el. Activating transcription factor 6 limits intracellular accumulation of mutant α(1)-antitrypsin Z and mitochondrial damage in hepatoma cells. J Biol Chem. 2011 Dec 2;286(48):41563-77.

372. Copetti T, Bertoli C, Dalla E, et el. p65/RelA modulates BECN1 transcription and autophagy. Mol Cell Biol. 2009 May;29(10):2594-608.

373. Msaki A, Sánchez AM, Koh LF, et el. The role of RelA (p65) threonine 505 phosphorylation in the regulation of cell growth, survival, and migration. Mol Biol Cell. 2011 Sep;22(17):3032-40.

374. Ciavarra G, Zacksenhaus E. Rescue of myogenic defects in Rb-deficient cells by inhibition of autophagy or by hypoxia-induced glycolytic shift. J Cell Biol. 2010 Oct 18;191(2):291-301.

375. Walmsley B, McCormack L. Moderate dementia: relational social engagement (RSE) during family visits. Aging Ment Health. 2018 Aug;22(8):954-963.

376. Guixé-Muntet S, de Mesquita FC, Vila S, et el. Cross-talk between autophagy and KLF2 determines endothelial cell phenotype and microvascular function in acute liver injury. J Hepatol. 2017 Jan;66(1):86-94.

377. Winslow AR, Chen CW, Corrochano S, et el. α-Synuclein impairs macroautophagy: implications for Parkinson's disease. J Cell Biol. 2010 Sep 20;190(6):1023-37.

378. Dong N, Zhu Y, Lu Q, et el. Structurally distinct bacterial TBC-like GAPs link Arf GTPase to Rab1 inactivation to counteract host defenses. Cell. 2012 Aug 31;150(5):1029-41.

379. Beitz JM. Fecal incontinence in acutely and critically ill patients: options in management. Ostomy Wound Manage. 2006 Dec;52(12):56-8, 60, 62-6.

380. Mukai R, Ohshima T. HTLV-1 HBZ positively regulates the mTOR signaling pathway via inhibition of GADD34 activity in the cytoplasm. Oncogene. 2014 May 1;33(18):2317-28.

381. Nascimbeni AC, Fanin M, Angelini C, et el. Autophagy dysregulation in Danon disease. Cell Death Dis. 2017 Jan 19;8(1):e2565.

382. Su WC, Chao TC, Huang YL, et el. Rab5 and class III phosphoinositide 3-kinase Vps34 are involved in hepatitis C virus NS4B-induced autophagy. J Virol. 2011 Oct;85(20):10561-71.

383. Willinger T, Flavell RA. Canonical autophagy dependent on the class III phosphoinositide-3 kinase Vps34 is required for naive T-cell homeostasis. Proc Natl Acad Sci U S A. 2012 May 29;109(22):8670-5.

384. Guo L, Stripay JL, Zhang X, et el. CaMKIα regulates AMP kinase-dependent, TORC-1-independent autophagy during lipopolysaccharide-induced acute lung neutrophilic inflammation. J Immunol. 2013 Apr 1;190(7):3620-8.

385. Yamashita S, Abe K, Tatemichi Y, et el. The membrane peroxin PEX3 induces peroxisome-ubiquitination-linked pexophagy. Autophagy. 2014 Sep;10(9):1549-64.

386. Suzuki T, Franchi L, Toma C, et el. Differential regulation of caspase-1 activation, pyroptosis, and autophagy via Ipaf and ASC in Shigella-infected macrophages. PLoS Pathog. 2007 Aug 10;3(8):e111.

387. Hsu CP, Oka S, Shao D, et el. Nicotinamide phosphoribosyltransferase regulates cell survival through NAD+ synthesis in cardiac myocytes. Circ Res. 2009 Aug 28;105(5):481-91.

388. Conacci-Sorrell M, Ngouenet C, Anderson S, et el. Stress-induced cleavage of Myc promotes cancer cell survival. Genes Dev. 2014 Apr 1;28(7):689-707.

389. Raciti M, Lotti LV, Valia S, et el. JNK2 is activated during ER stress and promotes cell survival. Cell Death Dis. 2012 Nov 22;3:e429.

390. Escobar ML, Echeverría OM, Ortíz R, et el. Combined apoptosis and autophagy, the process that eliminates the oocytes of atretic follicles in immature rats. Apoptosis. 2008 Oct;13(10):1253-66.

391. Cardoso CM, Groth-Pedersen L, Høyer-Hansen M, et el. Depletion of kinesin 5B affects lysosomal distribution and stability and induces peri-nuclear accumulation of autophagosomes in cancer cells. PLoS One. 2009;4(2):e4424.

392. He Q, Huang B, Zhao J, et el. Knockdown of integrin beta4-induced autophagic cell death associated with P53 in A549 lung adenocarcinoma cells. FEBS J. 2008 Nov;275(22):5725-32.

393. Li H, Huang S, Wang S, et el. Relationship between annexin A7 and integrin β4 in autophagy. Int J Biochem Cell Biol. 2013 Nov;45(11):2605-11.

394. Yacoub A, Hamed HA, Allegood J, et el. PERK-dependent regulation of ceramide synthase 6 and thioredoxin play a key role in mda-7/IL-24-induced killing of primary human glioblastoma multiforme cells. Cancer Res. 2010 Feb 1;70(3):1120-9.

395. Inbal B, Bialik S, Sabanay I, et el. DAP kinase and DRP-1 mediate membrane blebbing and the formation of autophagic vesicles during programmed cell death. J Cell Biol. 2002 Apr 29;157(3):455-68.

396. Matsuzawa T, Kim BH, Shenoy AR, et el. IFN-γ elicits macrophage autophagy via the p38 MAPK signaling pathway. J Immunol. 2012 Jul 15;189(2):813-8.

397. Kimura T, Jain A, Choi SW, et el. TRIM-mediated precision autophagy targets cytoplasmic regulators of innate immunity. J Cell Biol. 2015 Sep 14;210(6):973-89.

398. Wu Y, Ma C, Zhao H, et el. Alleviation of endoplasmic reticulum stress protects against cisplatin-induced ovarian damage. Reprod Biol Endocrinol. 2018 Sep 3;16(1):85.

399. Moresi V, Carrer M, Grueter CE, et el. Histone deacetylases 1 and 2 regulate autophagy flux and skeletal muscle homeostasis in mice. Proc Natl Acad Sci U S A. 2012 Jan 31;109(5):1649-54.

400. Shimada Y, Kobayashi H, Kawagoe S, et el. Endoplasmic reticulum stress induces autophagy through activation of p38 MAPK in fibroblasts from Pompe disease patients carrying c.546G>T mutation. Mol Genet Metab. 2011 Dec;104(4):566-73.

401. Hariharan N, Ikeda Y, Hong C, et el. Autophagy plays an essential role in mediating regression of hypertrophy during unloading of the heart. PLoS One. 2013;8(1):e51632.

402. Park SM, Schickel R, Peter ME. Nonapoptotic functions of FADD-binding death receptors and their signaling molecules. Curr Opin Cell Biol. 2005 Dec;17(6):610-6.

403. Wang BJ, Her GM, Hu MK, et el. ErbB2 regulates autophagic flux to modulate the proteostasis of APP-CTFs in Alzheimer's disease. Proc Natl Acad Sci U S A. 2017 Apr 11;114(15):E3129-E3138.

404. Ramírez-Valle F, Braunstein S, Zavadil J, et el. eIF4GI links nutrient sensing by mTOR to cell proliferation and inhibition of autophagy. J Cell Biol. 2008 Apr 21;181(2):293-307.

405. Badura M, Braunstein S, Zavadil J, et el. DNA damage and eIF4G1 in breast cancer cells reprogram translation for survival and DNA repair mRNAs. Proc Natl Acad Sci U S A. 2012 Nov 13;109(46):18767-72.

406. Crighton D, Wilkinson S, Ryan KM. DRAM links autophagy to p53 and programmed cell death. Autophagy. 2007 Jan-Feb;3(1):72-4.

407. Wu HT, Xie CR, Lv J, et el. The tumor suppressor DLC1 inhibits cancer progression and oncogenic autophagy in hepatocellular carcinoma. Lab Invest. 2018 Aug;98(8):1014-1024.

408. Bialik S, Kimchi A. The death-associated protein kinases: structure, function, and beyond. Annu Rev Biochem. 2006;75:189-210.

409. Kiyono K, Suzuki HI, Matsuyama H, et el. Autophagy is activated by TGF-beta and potentiates TGF-beta-mediated growth inhibition in human hepatocellular carcinoma cells. Cancer Res. 2009 Dec 1;69(23):8844-52.

410. Eisenberg-Lerner A, Kimchi A. PKD is a kinase of Vps34 that mediates ROS-induced autophagy downstream of DAPk. Cell Death Differ. 2012 May;19(5):788-97.

411. Lee JA, Beigneux A, Ahmad ST, et el. ESCRT-III dysfunction causes autophagosome accumulation and neurodegeneration. Curr Biol. 2007 Sep 18;17(18):1561-7.

412. Hurley JH, Hanson PI. Membrane budding and scission by the ESCRT machinery: it's all in the neck. Nat Rev Mol Cell Biol. 2010 Aug;11(8):556-66.

413. Metcalf D, Isaacs AM. The role of ESCRT proteins in fusion events involving lysosomes, endosomes and autophagosomes. Biochem Soc Trans. 2010 Dec;38(6):1469-73.

414. Lu Y, Zhang Z, Sun D, et el. Syntaxin 13, a genetic modifier of mutant CHMP2B in frontotemporal dementia, is required for autophagosome maturation. Mol Cell. 2013 Oct 24;52(2):264-71.

415. Tang SJ, Luo S, Ho JX, et el. Characterization of the Regulation of CD46 RNA Alternative Splicing. J Biol Chem. 2016 Jul 1;291(27):14311-23.

416. Roca H, Varsos Z, Pienta KJ. CCL2 protects prostate cancer PC3 cells from autophagic death via phosphatidylinositol 3-kinase/AKT-dependent survivin up-regulation. J Biol Chem. 2008 Sep 5;283(36):25057-73.

417. Wlodarska M, Thaiss CA, Nowarski R, et el. NLRP6 inflammasome orchestrates the colonic host-microbial interface by regulating goblet cell mucus secretion. Cell. 2014 Feb 27;156(5):1045-59.

418. Low CG, Luk IS, Lin D, et el. BIRC6 protein, an inhibitor of apoptosis: role in survival of human prostate cancer cells. PLoS One. 2013;8(2):e55837.

419. Shi K, An J, Shan L, et el. Survivin-2B promotes autophagy by accumulating IKK alpha in the nucleus of selenite-treated NB4 cells. Cell Death Dis. 2014 Feb 20;5:e1071.

420. Yeganeh B, Ghavami S, Rahim MN, et el. Autophagy activation is required for influenza A virus-induced apoptosis and replication. Biochim Biophys Acta Mol Cell Res. 2018 Feb;1865(2):364-378.

421. Pedro JM, Wei Y, Sica V, et el. BAX and BAK1 are dispensable for ABT-737-induced dissociation of the BCL2-BECN1 complex and autophagy. Autophagy. 2015;11(3):452-9.

422. Li X, Chen D, Hua Q, et el. Induction of Autophagy interferes the tachyzoite to bradyzoite transformation of Toxoplasma gondii. Parasitology. 2016 Apr;143(5):639-45.

423. Sharma M, Bhattacharyya S, Sharma KB, et el. Japanese encephalitis virus activates autophagy through XBP1 and ATF6 ER stress sensors in neuronal cells. J Gen Virol. 2017 May;98(5):1027-1039.

424. Fiesel FC, Moussaud-Lamodière EL, Ando M, et el. A specific subset of E2 ubiquitin-conjugating enzymes regulate Parkin activation and mitophagy differently. J Cell Sci. 2014 Aug 15;127(Pt 16):3488-504.

425. Yuan J, Zhang Y, Sheng Y, et el. MYBL2 guides autophagy suppressor VDAC2 in the developing ovary to inhibit autophagy through a complex of VDAC2-BECN1-BCL2L1 in mammals. Autophagy. 2015;11(7):1081-98.

426. Sánchez-Martín P, Romá-Mateo C, Viana R, et el. Ubiquitin conjugating enzyme E2-N and sequestosome-1 (p62) are components of the ubiquitination process mediated by the malin-laforin E3-ubiquitin ligase complex. Int J Biochem Cell Biol. 2015 Dec;69:204-14.

427. Ambivero CT, Cilenti L, Main S, et el. Mulan E3 ubiquitin ligase interacts with multiple E2 conjugating enzymes and participates in mitophagy by recruiting GABARAP. Cell Signal. 2014 Dec;26(12):2921-9.

428. Peng H, Yang J, Li G, et el. Ubiquitylation of p62/sequestosome1 activates its autophagy receptor function and controls selective autophagy upon ubiquitin stress. Cell Res. 2017 May;27(5):657-674.

429. Liu KK, Qiu WR, Naveen Raj E, et el. Ubiquitin-coated nanodiamonds bind to autophagy receptors for entry into the selective autophagy pathway. Autophagy. 2017 Jan 2;13(1):187-200.

430. Lynch-Day MA, Bhandari D, Menon S, et el. Trs85 directs a Ypt1 GEF, TRAPPIII, to the phagophore to promote autophagy. Proc Natl Acad Sci U S A. 2010 Apr

27;107(17):7811-6.

431. Imai K, Hao F, Fujita N, et el. Atg9A trafficking through the recycling endosomes is required for autophagosome formation. J Cell Sci. 2016 Oct 15;129(20):3781-3791.

432. Lamb CA, Nühlen S, Judith D, et el. TBC1D14 regulates autophagy via the TRAPP complex and ATG9 traffic. EMBO J. 2016 Feb 1;35(3):281-301.

433. Bachetti T, Chiesa S, Castagnola P, et el. Autophagy contributes to inflammation in patients with TNFR-associated periodic syndrome (TRAPS). Ann Rheum Dis. 2013 Jun;72(6):1044-52.

434. Salaheen S, Peng M, Joo J, et el. Eradication and Sensitization of Methicillin Resistant Staphylococcus aureus to Methicillin with Bioactive Extracts of Berry Pomace. Front Microbiol. 2017 Feb 21;8:253.

435. Hasson SA, Kane LA, Yamano K, et el. High-content genome-wide RNAi screens identify regulators of parkin upstream of mitophagy. Nature. 2013 Dec 12;504(7479):291-5.

436. Rasmussen SB, Horan KA, Holm CK, et el. Activation of autophagy by α-herpesviruses in myeloid cells is mediated by cytoplasmic viral DNA through a mechanism dependent on stimulator of IFN genes. J Immunol. 2011 Nov 15;187(10):5268-76.

437. Brown MC, Gromeier M. MNK Controls mTORC1:Substrate Association through Regulation of TELO2 Binding with mTORC1. Cell Rep. 2017 Feb 7;18(6):1444-1457.

438. Pirooz SD, He S, Zhang T, et el. UVRAG is required for virus entry through combinatorial interaction with the class C-Vps complex and SNAREs. Proc Natl Acad Sci U S A. 2014 Feb 18;111(7):2716-21.

439. Wang L, Kim JY, Liu HM, et el. HCV-induced autophagosomes are generated via homotypic fusion of phagophores that mediate HCV RNA replication. PLoS Pathog. 2017 Sep 19;13(9):e1006609.

440. Campos-Parra AD, Padua-Bracho A, Pedroza-Torres A, et el. Comprehensive transcriptome analysis identifies pathways with therapeutic potential in locally advanced cervical cancer. Gynecol Oncol. 2016 Nov;143(2):406-413.

441. He B, Zhang N, Zhao R. Dexamethasone Downregulates SLC7A5 Expression and Promotes Cell Cycle Arrest, Autophagy and Apoptosis in BeWo Cells. J Cell Physiol. 2016 Jan;231(1):233-42.

442. Tattoli I, Philpott DJ, Girardin SE. The bacterial and cellular determinants controlling the

recruitment of mTOR to the Salmonella-containing vacuole. Biol Open. 2012 Dec 15;1(12):1215-25.

443. Aizawa S, Fujiwara Y, Contu VR, et el. Lysosomal putative RNA transporter SIDT2 mediates direct uptake of RNA by lysosomes. Autophagy. 2016;12(3):565-78.

444. Pujals A, Favre L, Pioche-Durieu C, et el. Constitutive autophagy contributes to resistance to TP53-mediated apoptosis in Epstein-Barr virus-positive latency III B-cell lymphoproliferations. Autophagy. 2015;11(12):2275-87.

445. Choi H, Son JB, Kang J, et el. Leucine-induced localization of Leucyl-tRNA synthetase in lysosome membrane. Biochem Biophys Res Commun. 2017 Nov 18;493(2):1129-1135.

446. Efeyan A, Zoncu R, Chang S, et el. Regulation of mTORC1 by the Rag GTPases is necessary for neonatal autophagy and survival. Nature. 2013 Jan 31;493(7434):679-83.

447. Deng L, Jiang C, Chen L, et el. The ubiquitination of rag A GTPase by RNF152 negatively regulates mTORC1 activation. Mol Cell. 2015 Jun 4;58(5):804-18.

448. Martin TD, Chen XW, Kaplan RE, et el. Ral and Rheb GTPase activating proteins integrate mTOR and GTPase signaling in aging, autophagy, and tumor cell invasion. Mol Cell. 2014 Jan 23;53(2):209-20.

449. Yeasmin AM, Waliullah TM, Kondo A, et el. Orchestrated Action of PP2A Antagonizes Atg13 Phosphorylation and Promotes Autophagy after the Inactivation of TORC1. PLoS One. 2016 Dec 14;11(12):e0166636.

450. Yamamoto K, Seki T, Adachi N, et el. Mutant protein kinase C gamma that causes spinocerebellar ataxia type 14 (SCA14) is selectively degraded by autophagy. Genes Cells. 2010 May;15(5):425-38.

451. Li YB, Pei XY, Wang D, et el. The steroid hormone 20-hydroxyecdysone upregulates calcium release-activated calcium channel modulator 1 expression to induce apoptosis in the midgut of Helicoverpa armigera. Cell Calcium. 2017 Dec;68:24-33.

452. Eun SY, Lee JN, Nam IK, et el. PEX5 regulates autophagy via the mTORC1-TFEB axis during starvation. Exp Mol Med. 2018 Apr 6;50(4):4.

453. Gómez-Suárez M, Gutiérrez-Martínez IZ, Hernández-Trejo JA, et el. 14-3-3 Proteins regulate Akt Thr308 phosphorylation in intestinal epithelial cells. Cell Death Differ. 2016 Jun;23(6):1060-72.

454. Wang K, Gao W, Dou Q, et el. Ivermectin induces PAK1-mediated cytostatic autophagy

in breast cancer. Autophagy. 2016 Dec;12(12):2498-2499.

455. Wu F, Li S, Zhang N, et el. Hispidulin alleviates high-glucose-induced podocyte injury by regulating protective autophagy. Biomed Pharmacother. 2018 Aug;104:307-314.

456. Leonardi M, Perna E, Tronnolone S, et el. Activated kinase screening identifies the IKBKE oncogene as a positive regulator of autophagy. Autophagy. 2018 Oct 5:1-15.

457. Wang GQ, Tang T, Wang ZS, et el. Overexpression of Hypo-Phosphorylated IκBβ at Ser313 Protects the Heart against Sepsis. PLoS One. 2016 Aug 10;11(8):e0160860.

458. Huang Q, Zhan L, Cao H, et el. Increased mitochondrial fission promotes autophagy and hepatocellular carcinoma cell survival through the ROS-modulated coordinated regulation of the NFKB and TP53 pathways. Autophagy. 2016 Jun 2;12(6):999-1014.

459. Zhang M, Zhang J, Lu L, et el. Enhancement of chondrocyte autophagy is an early response in the degenerative cartilage of the temporomandibular joint to biomechanical dental stimulation. Apoptosis. 2013 Apr;18(4):423-34.

460. Qing G, Yan P, Qu Z, et el. Hsp90 regulates processing of NF-kappa B2 p100 involving protection of NF-kappa B-inducing kinase (NIK) from autophagy-mediated degradation. Cell Res. 2007 Jun;17(6):520-30.

461. Wang Z, Hu J, Li G, et el. PHF23 (plant homeodomain finger protein 23) negatively regulates cell autophagy by promoting ubiquitination and degradation of E3 ligase LRSAM1. Autophagy. 2014;10(12):2158-70.

462. Kania E, Roest G, Vervliet T, et el. IP(3) Receptor-Mediated Calcium Signaling and Its Role in Autophagy in Cancer. Front Oncol. 2017 Jul 5;7:140.

463. Bugnicourt A, Mari M, Reggiori F, et el. Irs4p and Tax4p: two redundant EH domain proteins involved in autophagy. Traffic. 2008 May;9(5):755-69.

464. O'Neill BT, Lee KY, Klaus K, et el. Insulin and IGF-1 receptors regulate FoxO-mediated signaling in muscle proteostasis. J Clin Invest. 2016 Sep 1;126(9):3433-46.

465. Naito T, Kuma A, Mizushima N. Differential contribution of insulin and amino acids to the mTORC1-autophagy pathway in the liver and muscle. J Biol Chem. 2013 Jul 19;288(29):21074-81.

466. Zientara-Rytter K, Sirko A. To deliver or to degrade - an interplay of the ubiquitin-proteasome system, autophagy and vesicular transport in plants. FEBS J. 2016 Oct;283(19):3534-3555.

467. Li Y, Zhao Y, Su M, et el. Structural insights into the interaction of the conserved mammalian proteins GAPR-1 and Beclin 1, a key autophagy protein. Acta Crystallogr D Struct Biol. 2017 Sep 1;73(Pt 9):775-792.

468. Bhujabal Z, Birgisdottir ÅB, Sjøttem E, et el. FKBP8 recruits LC3A to mediate Parkin-independent mitophagy. EMBO Rep. 2017 Jun;18(6):947-961.

469. Tanaka T, Goto K, Iino M. Diverse Functions and Signal Transduction of the Exocyst Complex in Tumor Cells. J Cell Physiol. 2017 May;232(5):939-957.

470. Yip PY. Phosphatidylinositol 3-kinase-AKT-mammalian target of rapamycin (PI3K-Akt-mTOR) signaling pathway in non-small cell lung cancer. Transl Lung Cancer Res. 2015 Apr;4(2):165-76.

471. Blandino-Rosano M, Barbaresso R, Jimenez-Palomares M, et el. Loss of mTORC1 signalling impairs β-cell homeostasis and insulin processing. Nat Commun. 2017 Jul 12;8:16014.

472. Lee HJ, Venkatarame Gowda Saralamma V, Kim SM, et el. Pectolinarigenin Induced Cell Cycle Arrest, Autophagy, and Apoptosis in Gastric Cancer Cell via PI3K/AKT/mTOR Signaling Pathway. Nutrients. 2018 Aug 8;10(8).

473. Niso-Santano M, Shen S, Adjemian S, et el. Direct interaction between STAT3 and EIF2AK2 controls fatty acid-induced autophagy. Autophagy. 2013 Mar;9(3):415-7.

474. Byun HO, Han NK, Lee HJ, et el. Cathepsin D and eukaryotic translation elongation factor 1 as promising markers of cellular senescence. Cancer Res. 2009 Jun 1;69(11):4638-47.

475. Durieux AC, Vassilopoulos S, Lainé J, et el. A centronuclear myopathy--dynamin 2 mutation impairs autophagy in mice. Traffic. 2012 Jun;13(6):869-79.

476. Nieto-Jacobo F, Pasch D, Basse CW. The mitochondrial Dnm1-like fission component is required for lgA2-induced mitophagy but dispensable for starvation-induced mitophagy in Ustilago maydis. Eukaryot Cell. 2012 Sep;11(9):1154-66.

477. Kravic B, Harbauer AB, Romanello V, et el. In mammalian skeletal muscle, phosphorylation of TOMM22 by protein kinase CSNK2/CK2 controls mitophagy. Autophagy. 2018;14(2):311-335.

478. Enomoto S, Shimizu K, Nibuya M, et el. Increased expression of endocytosis-Related proteins in rat hippocampus following 10-day electroconvulsive seizure treatment. Neurosci Lett. 2016 Jun 15;624:85-91.

479. Demarchi F, Schneider C. The calpain system as a modulator of stress/damage response. Cell Cycle. 2007 Jan 15;6(2):136-8.

480. Smith ED, Prieto GA, Tong L, et el. Rapamycin and interleukin-1β impair brain-derived neurotrophic factor-dependent neuron survival by modulating autophagy. J Biol Chem. 2014 Jul 25;289(30):20615-29.

481. Sheng Z, Ma L, Sun JE, et el. BCR-ABL suppresses autophagy through ATF5-mediated regulation of mTOR transcription. Blood. 2011 Sep 8;118(10):2840-8.

482. Prigione A, Cortopassi G. Mitochondrial DNA deletions induce the adenosine monophosphate-activated protein kinase energy stress pathway and result in decreased secretion of some proteins. Aging Cell. 2007 Oct;6(5):619-30.

483. Tian Y, Chang JC, Fan EY, et el. Adaptor complex AP2/PICALM, through interaction with LC3, targets Alzheimer's APP-CTF for terminal degradation via autophagy. Proc Natl Acad Sci U S A. 2013 Oct 15;110(42):17071-6.

484. Mahil SK, Twelves S, Farkas K, et el. AP1S3 Mutations Cause Skin Autoinflammation by Disrupting Keratinocyte Autophagy and Up-Regulating IL-36 Production. J Invest Dermatol. 2016 Nov;136(11):2251-2259.

485. Contu VR, Hase K, Kozuka-Hata H, et el. Lysosomal targeting of SIDT2 via multiple YxxΦ motifs is required for SIDT2 function in the process of RNautophagy. J Cell Sci. 2017 Sep 1;130(17):2843-2853.

486. Guo Y, Chang C, Huang R, et el. AP1 is essential for generation of autophagosomes from the trans-Golgi network. J Cell Sci. 2012 Apr 1;125(Pt 7):1706-15.

487. Tchetina EV, Maslova KA, Krylov MY, et el. Association of bone loss with the upregulation of survival-related genes and concomitant downregulation of Mammalian target of rapamycin and osteoblast differentiation-related genes in the peripheral blood of late postmenopausal osteoporotic women. J Osteoporos. 2015;2015:802694.

488. He CL, Bian YY, Xue Y, et el. Pyruvate Kinase M2 Activates mTORC1 by Phosphorylating AKT1S1. Sci Rep. 2016 Feb 15;6:21524.

489. Moreau K, Rubinsztein DC. The plasma membrane as a control center for autophagy. Autophagy. 2012 May 1;8(5):861-3.

490. Younce C, Kolattukudy P. MCP-1 induced protein promotes adipogenesis via oxidative stress, endoplasmic reticulum stress and autophagy. Cell Physiol Biochem. 2012;30(2):307-20.

491. Corum DG, Tsichlis PN, Muise-Helmericks RC. AKT3 controls mitochondrial biogenesis and autophagy via regulation of the major nuclear export protein CRM-1. FASEB J. 2014 Jan;28(1):395-407.

492. Zhao Y, Li X, Cai MY, et el. XBP-1u suppresses autophagy by promoting the degradation of FoxO1 in cancer cells. Cell Res. 2013 Apr;23(4):491-507.

493. Valdés P, Mercado G, Vidal RL, et el. Control of dopaminergic neuron survival by the unfolded protein response transcription factor XBP1. Proc Natl Acad Sci U S A. 2014 May 6;111(18):6804-9.

494. Xia P, Wang S, Du Y, et el. WASH inhibits autophagy through suppression of Beclin 1 ubiquitination. EMBO J. 2013 Oct 16;32(20):2685-96.

495. Peng C, Ye J, Yan S, et el. Ablation of vacuole protein sorting 18 (Vps18) gene leads to neurodegeneration and impaired neuronal migration by disrupting multiple vesicle transport pathways to lysosomes. J Biol Chem. 2012 Sep 21;287(39):32861-73.

496. Dando I, Fiorini C, Pozza ED, et el. UCP2 inhibition triggers ROS-dependent nuclear translocation of GAPDH and autophagic cell death in pancreatic adenocarcinoma cells. Biochim Biophys Acta. 2013 Mar;1833(3):672-9.

497. Tasaki T, Kim ST, Zakrzewska A, et el. UBR box N-recognin-4 (UBR4), an N-recognin of the N-end rule pathway, and its role in yolk sac vascular development and autophagy. Proc Natl Acad Sci U S A. 2013 Mar 5;110(10):3800-5.

498. Rothenberg C, Srinivasan D, Mah L, et el. Ubiquilin functions in autophagy and is degraded by chaperone-mediated autophagy. Hum Mol Genet. 2010 Aug 15;19(16):3219-32.

499. Mandell MA, Jain A, Arko-Mensah J, et el. TRIM proteins regulate autophagy and can target autophagic substrates by direct recognition. Dev Cell. 2014 Aug 25;30(4):394-409.

500. Tomar D, Singh R, Singh AK, et el. TRIM13 regulates ER stress induced autophagy and clonogenic ability of the cells. Biochim Biophys Acta. 2012 Feb;1823(2):316-26.

501. Seillier M, Peuget S, Gayet O, et el. TP53INP1, a tumor suppressor, interacts with LC3 and ATG8-family proteins through the LC3-interacting region (LIR) and promotes autophagy-dependent cell death. Cell Death Differ. 2012 Sep;19(9):1525-35.

502. Sancho A, Duran J, García-España A, et el. DOR/Tp53inp2 and Tp53inp1 constitute a metazoan gene family encoding dual regulators of autophagy and transcription. PLoS One. 2012;7(3):e34034.

503. Colleran A, Ryan A, O'Gorman A, et el. Autophagosomal IkappaB alpha degradation plays a role in the long term control of tumor necrosis factor-alpha-induced nuclear factor-kappaB (NF-kappaB) activity. J Biol Chem. 2011 Jul 1;286(26):22886-93.

504. Boada-Romero E, Letek M, Fleischer A, et el. TMEM59 defines a novel ATG16L1-binding motif that promotes local activation of LC3. EMBO J. 2013 Feb 20;32(4):566-82.

505. Castillo K, Rojas-Rivera D, Lisbona F, et el. BAX inhibitor-1 regulates autophagy by controlling the IRE1α branch of the unfolded protein response. EMBO J. 2011 Sep 16;30(21):4465-78.

506. Bertin S, Samson M, Pons C, et el. Comparative proteomics study reveals that bacterial CpG motifs induce tumor cell autophagy in vitro and in vivo. Mol Cell Proteomics. 2008 Dec;7(12):2311-22.

507. Doyle A, Zhang G, Abdel Fattah EA, et el. Toll-like receptor 4 mediates lipopolysaccharide-induced muscle catabolism via coordinate activation of ubiquitin-proteasome and autophagy-lysosome pathways. FASEB J. 2011 Jan;25(1):99-110.

508. Neal MD, Sodhi CP, Dyer M, et el. A critical role for TLR4 induction of autophagy in the regulation of enterocyte migration and the pathogenesis of necrotizing enterocolitis. J Immunol. 2013 Apr 1;190(7):3541-51.

509. Dibble CC, Elis W, Menon S, et el. TBC1D7 is a third subunit of the TSC1-TSC2 complex upstream of mTORC1. Mol Cell. 2012 Aug 24;47(4):535-46.

510. Wauson EM, Zaganjor E, Lee AY, et el. The G protein-coupled taste receptor T1R1/T1R3 regulates mTORC1 and autophagy. Mol Cell. 2012 Sep 28;47(6):851-62.

511. Takaesu G, Kobayashi T, Yoshimura A. TGFβ-activated kinase 1 (TAK1)-binding proteins (TAB) 2 and 3 negatively regulate autophagy. J Biochem. 2012 Feb;151(2):157-66.

512. Wang Y, Ballar P, Zhong Y, et el. SVIP induces localization of p97/VCP to the plasma and lysosomal membranes and regulates autophagy. PLoS One. 2011;6(8):e24478.

513. King KY, Baldridge MT, Weksberg DC, et el. Irgm1 protects hematopoietic stem cells by negative regulation of IFN signaling. Blood. 2011 Aug 11;118(6):1525-33.

514. Fukui K, Ferris HA, Kahn CR. Effect of cholesterol reduction on receptor signaling in neurons. J Biol Chem. 2015 Oct 30;290(44):26383-92.

515. Zheng YH, Tian C, Meng Y, et el. Osteopontin stimulates autophagy via integrin/CD44 and p38 MAPK signaling pathways in vascular smooth muscle cells. J Cell Physiol. 2012 Jan;227(1):127-35.

516. Stefanis L, Larsen KE, Rideout HJ, et el. Expression of A53T mutant but not wild-type alpha-synuclein in PC12 cells induces alterations of the ubiquitin-dependent degradation system, loss of dopamine release, and autophagic cell death. J Neurosci. 2001 Dec 15;21(24):9549-60.

517. Calì T, Ottolini D, Negro A, et el. α-Synuclein controls mitochondrial calcium homeostasis by enhancing endoplasmic reticulum-mitochondria interactions. J Biol Chem. 2012 May 25;287(22):17914-29.

518. Lin X, Parisiadou L, Sgobio C, et el. Conditional expression of Parkinson's disease-related mutant α-synuclein in the midbrain dopaminergic neurons causes progressive neurodegeneration and degradation of transcription factor nuclear receptor related 1. J Neurosci. 2012 Jul 4;32(27):9248-64.

519. Steneberg P, Bernardo L, Edfalk S, et el. The type 2 diabetes-associated gene ide is required for insulin secretion and suppression of α-synuclein levels in β-cells. Diabetes. 2013 Jun;62(6):2004-14.

520. Chen L, Xie Z, Turkson S, et el. A53T human α-synuclein overexpression in transgenic mice induces pervasive mitochondria macroautophagy defects preceding dopamine neuron degeneration. J Neurosci. 2015 Jan 21;35(3):890-905.

521. Pan CC, Kumar S, Shah N, et el. Endoglin Regulation of Smad2 Function Mediates Beclin1 Expression and Endothelial Autophagy. J Biol Chem. 2015 Jun 12;290(24):14884-92.

522. Peng Y, Li M, Clarkson BD, et el. Deficient import of acetyl-CoA into the ER lumen causes neurodegeneration and propensity to infections, inflammation, and cancer. J Neurosci. 2014 May 14;34(20):6772-89.

523. Takasaka N, Araya J, Hara H, et el. Autophagy induction by SIRT6 through attenuation of insulin-like growth factor signaling is involved in the regulation of human bronchial epithelial cell senescence. J Immunol. 2014 Feb 1;192(3):958-68.

524. Yang FC, Tan BC, Chen WH, et el. Reversible acetylation regulates salt-inducible kinase (SIK2) and its function in autophagy. J Biol Chem. 2013 Mar 1;288(9):6227-37.

525. Ogasawara Y, Itakura E, Kono N, et el. Stearoyl-CoA desaturase 1 activity is required for autophagosome formation. J Biol Chem. 2014 Aug 22;289(34):23938-50.

526. Ghavami S, Eshragi M, Ande SR, et el. S100A8/A9 induces autophagy and apoptosis via ROS-mediated cross-talk between mitochondria and lysosomes that involves BNIP3. Cell Res. 2010 Mar;20(3):314-31.

527. Bround MJ, Wambolt R, Luciani DS, et el. Cardiomyocyte ATP production, metabolic flexibility, and survival require calcium flux through cardiac ryanodine receptors in vivo. J Biol Chem. 2013 Jun 28;288(26):18975-86.

528. Bauer PO, Wong HK, Oyama F, et el. Inhibition of Rho kinases enhances the degradation of mutant huntingtin. J Biol Chem. 2009 May 8;284(19):13153-64.

529. Gurkar AU, Chu K, Raj L, et el. Identification of ROCK1 kinase as a critical regulator of Beclin1-mediated autophagy during metabolic stress. Nat Commun. 2013;4:2189.

530. Kuang E, Okumura CY, Sheffy-Levin S, et el. Regulation of ATG4B stability by RNF5 limits basal levels of autophagy and influences susceptibility to bacterial infection. PLoS Genet. 2012;8(10):e1003007.

531. Mealer RG, Murray AJ, Shahani N, et el. Rhes, a striatal-selective protein implicated in Huntington disease, binds beclin-1 and activates autophagy. J Biol Chem. 2014 Feb 7;289(6):3547-54.

532. Zhu WL, Hossain MS, Guo DY, et el. A role for Rac3 GTPase in the regulation of autophagy. J Biol Chem. 2011 Oct 7;286(40):35291-8.

533. Poillet L, Pernodet N, Boyer-Guittaut M, et el. QSOX1 inhibits autophagic flux in breast cancer cells. PLoS One. 2014 Jan 24;9(1):e86641.

534. Spalinger MR, Lang S, Vavricka SR, et el. Protein tyrosine phosphatase non-receptor type 22 modulates NOD2-induced cytokine release and autophagy. PLoS One. 2013 Aug 26;8(8):e72384.

535. Wilson CA, Murphy DD, Giasson BI, et el. Degradative organelles containing mislocalized alpha-and beta-synuclein proliferate in presenilin-1 null neurons. J Cell Biol. 2004 May 10;165(3):335-46.

536. Tatti M, Motta M, Di Bartolomeo S, et el. Reduced cathepsins B and D cause impaired autophagic degradation that can be almost completely restored by overexpression of these two proteases in Sap C-deficient fibroblasts. Hum Mol Genet. 2012 Dec 1;21(23):5159-73.

537. Pandhare J, Dash S, Jones B, et el. A Novel Role of Proline Oxidase in HIV-1 Envelope Glycoprotein-induced Neuronal Autophagy. J Biol Chem. 2015 Oct 16;290(42):25439-51.

538. Zhou M, Ottenberg G, Sferrazza GF, et el. Highly neurotoxic monomeric α-helical prion protein. Proc Natl Acad Sci U S A. 2012 Feb 21;109(8):3113-8.

539. Daido S, Yamamoto A, Fujiwara K, et el. Inhibition of the DNA-dependent protein kinase catalytic subunit radiosensitizes malignant glioma cells by inducing autophagy. Cancer Res. 2005 May 15;65(10):4368-75.

540. Jiang M, Fernandez S, Jerome WG, et el. Disruption of PPARgamma signaling results in mouse prostatic intraepithelial neoplasia involving active autophagy. Cell Death Differ. 2010 Mar;17(3):469-81.

541. Lin WJ, Yang CY, Li LL, et el. Lysosomal targeting of phafin1 mediated by Rab7 induces autophagosome formation. Biochem Biophys Res Commun. 2012 Jan 6;417(1):35-42.

542. Chiavarina B, Whitaker-Menezes D, Martinez-Outschoorn UE, et el. Pyruvate kinase expression (PKM1 and PKM2) in cancer-associated fibroblasts drives stromal nutrient production and tumor growth. Cancer Biol Ther. 2011 Dec 15;12(12):1101-13.

543. Bohensky J, Shapiro IM, Leshinsky S, et el. PIM-2 is an independent regulator of chondrocyte survival and autophagy in the epiphyseal growth plate. J Cell Physiol. 2007 Oct;213(1):246-51.

544. Aki T, Yamaguchi K, Fujimiya T, et el. Phosphoinositide 3-kinase accelerates autophagic cell death during glucose deprivation in the rat cardiomyocyte-derived cell line H9c2. Oncogene. 2003 Nov 20;22(52):8529-35.

545. Dbouk HA, Backer JM. A beta version of life: p110β takes center stage. Oncotarget. 2010 Dec;1(8):729-33.

546. Wojtalla A, Fischer B, Kotelevets N, et el. Targeting the phosphoinositide 3-kinase p110-α isoform impairs cell proliferation, survival, and tumor growth in small cell lung cancer. Clin Cancer Res. 2013 Jan 1;19(1):96-105.

547. Devereaux K, Dall'Armi C, Alcazar-Roman A, et el. Regulation of mammalian autophagy by class II and III PI 3-kinases through PI3P synthesis. PLoS One. 2013 Oct 3;8(10):e76405.

548. Shen X, Ying H, Qiu Y, et el. Processing of optineurin in neuronal cells. J Biol Chem. 2011 Feb 4;286(5):3618-29.

549. Wild P, Farhan H, McEwan DG, et el. Phosphorylation of the autophagy receptor optineurin restricts Salmonella growth. Science. 2011 Jul 8;333(6039):228-33.

550. Sarkar S, Korolchuk VI, Renna M, et el. Complex inhibitory effects of nitric oxide on

autophagy. Mol Cell. 2011 Jul 8;43(1):19-32.

551. Jounai N, Kobiyama K, Shiina M, et el. NLRP4 negatively regulates autophagic processes through an association with beclin1. J Immunol. 2011 Feb 1;186(3):1646-55.

552. Tringali C, Lupo B, Silvestri I, et el. The plasma membrane sialidase NEU3 regulates the malignancy of renal carcinoma cells by controlling β1 integrin internalization and recycling. J Biol Chem. 2012 Dec 14;287(51):42835-45.

553. Sahni S, Bae DH, Lane DJ, et el. The metastasis suppressor, N-myc downstream-regulated gene 1 (NDRG1), inhibits stress-induced autophagy in cancer cells. J Biol Chem. 2014 Apr 4;289(14):9692-709.

554. Zhang Y, Cheng Y, Ren X, et el. NAC1 modulates sensitivity of ovarian cancer cells to cisplatin by altering the HMGB1-mediated autophagic response. Oncogene. 2012 Feb 23;31(8):1055-64.

555. Taguchi-Atarashi N, Hamasaki M, Matsunaga K, et el. Modulation of local PtdIns3P levels by the PI phosphatase MTMR3 regulates constitutive autophagy. Traffic. 2010 Apr;11(4):468-78.

556. Bhutia SK, Kegelman TP, Das SK, et el. Astrocyte elevated gene-1 induces protective autophagy. Proc Natl Acad Sci U S A. 2010 Dec 21;107(51):22243-8.

557. Tesfay L, Schulz VV, Frank SB, et el. Receptor tyrosine kinase Met promotes cell survival via kinase-independent maintenance of integrin α3β1. Mol Biol Cell. 2016 Aug 1;27(15):2493-504.

558. Martina JA, Lelouvier B, Puertollano R. The calcium channel mucolipin-3 is a novel regulator of trafficking along the endosomal pathway. Traffic. 2009 Aug;10(8):1143-56.

559. Zeevi DA, Lev S, Frumkin A, et el. Heteromultimeric TRPML channel assemblies play a crucial role in the regulation of cell viability models and starvation-induced autophagy. J Cell Sci. 2010 Sep 15;123(Pt 18):3112-24.

560. Thomas RL, Roberts DJ, Kubli DA, et el. Loss of MCL-1 leads to impaired autophagy and rapid development of heart failure. Genes Dev. 2013 Jun 15;27(12):1365-77.

561. Kalkat M, Garcia J, Ebrahimi J, et el. Placental autophagy regulation by the BOK-MCL1 rheostat. Autophagy. 2013 Dec;9(12):2140-53.

562. Lindqvist LM, Heinlein M, Huang DC, et el. Prosurvival Bcl-2 family members affect autophagy only indirectly, by inhibiting Bax and Bak. Proc Natl Acad Sci U S A. 2014 Jun 10;111(23):8512-7.

563. Pacheco CD, Elrick MJ, Lieberman AP. Tau deletion exacerbates the phenotype of Niemann-Pick type C mice and implicates autophagy in pathogenesis. Hum Mol Genet. 2009 Mar 1;18(5):956-65.

564. Xie R, Nguyen S, McKeehan K, et el. Microtubule-associated protein 1S (MAP1S) bridges autophagic components with microtubules and mitochondria to affect autophagosomal biogenesis and degradation. J Biol Chem. 2011 Mar 25;286(12):10367-77.

565. Alegre-Abarrategui J, Christian H, Lufino MM, et el. LRRK2 regulates autophagic activity and localizes to specific membrane microdomains in a novel human genomic reporter cellular model. Hum Mol Genet. 2009 Nov 1;18(21):4022-34.

566. Tong Y, Yamaguchi H, Giaime E, et el. Loss of leucine-rich repeat kinase 2 causes impairment of protein degradation pathways, accumulation of alpha-synuclein, and apoptotic cell death in aged mice. Proc Natl Acad Sci U S A. 2010 May 25;107(21):9879-84.

567. Manzoni C, Mamais A, Dihanich S, et el. Pathogenic Parkinson's disease mutations across the functional domains of LRRK2 alter the autophagic/lysosomal response to starvation. Biochem Biophys Res Commun. 2013 Nov 29;441(4):862-6.

568. Doonan PJ, Chandramoorthy HC, Hoffman NE, et el. LETM1-dependent mitochondrial Ca2+ flux modulates cellular bioenergetics and proliferation. FASEB J. 2014 Nov;28(11):4936-49.

569. Cassano S, Pucino V, La Rocca C, et el. Leptin modulates autophagy in human CD4+CD25- conventional T cells. Metabolism. 2014 Oct;63(10):1272-9.

570. Dominguez-Bautista JA, Klinkenberg M, Brehm N, et el. Loss of lysosome-associated membrane protein 3 (LAMP3) enhances cellular vulnerability against proteasomal inhibition. Eur J Cell Biol. 2015 Mar-Apr;94(3-4):148-61.

571. Buraschi S, Neill T, Goyal A, et el. Decorin causes autophagy in endothelial cells via Peg3. Proc Natl Acad Sci U S A. 2013 Jul 9;110(28):E2582-91.

572. Füllgrabe J, Lynch-Day MA, Heldring N, et el. The histone H4 lysine 16 acetyltransferase hMOF regulates the outcome of autophagy. Nature. 2013 Aug 22;500(7463):468-71.

573. Li XZ, Sui CY, Chen Q, et el. Promotion of autophagy at the maturation step by IL-6 is associated with the sustained mitogen-activated protein kinase/extracellular signal-regulated kinase activity. Mol Cell Biochem. 2013 Aug;380(1-2):219-27.

574. Liang X, De Vera ME, Buchser WJ, et el. Inhibiting systemic autophagy during interleukin 2 immunotherapy promotes long-term tumor regression. Cancer Res. 2012 Jun 1;72(11):2791-801.

575. English L, Chemali M, Duron J, et el. Autophagy enhances the presentation of endogenous viral antigens on MHC class I molecules during HSV-1 infection. Nat Immunol. 2009 May;10(5):480-7.

576. Mi S, Li Z, Yang HZ, et el. Blocking IL-17A promotes the resolution of pulmonary inflammation and fibrosis via TGF-beta1-dependent and -independent mechanisms. J Immunol. 2011 Sep 15;187(6):3003-14.

577. Gontier G, George C, Chaker Z, et el. Blocking IGF Signaling in Adult Neurons Alleviates Alzheimer's Disease Pathology through Amyloid-β Clearance. J Neurosci. 2015 Aug 19;35(33):11500-13.

578. Poluzzi C, Casulli J, Goyal A, et el. Endorepellin evokes autophagy in endothelial cells. J Biol Chem. 2014 Jun 6;289(23):16114-28.

579. Desai S, Liu Z, Yao J, et el. Heat shock factor 1 (HSF1) controls chemoresistance and autophagy through transcriptional regulation of autophagy-related protein 7 (ATG7). J Biol Chem. 2013 Mar 29;288(13):9165-76.

580. Bolisetty S, Traylor AM, Kim J, et el. Heme oxygenase-1 inhibits renal tubular macroautophagy in acute kidney injury. J Am Soc Nephrol. 2010 Oct;21(10):1702-12.

581. Tang D, Kang R, Livesey KM, et el. Endogenous HMGB1 regulates autophagy. J Cell Biol. 2010 Sep 6;190(5):881-92.

582. Pérez-Carrión MD, Ceña V. Knocking down HMGB1 using dendrimer-delivered siRNA unveils its key role in NMDA-induced autophagy in rat cortical neurons. Pharm Res. 2013 Oct;30(10):2584-95.

583. Oehme I, Linke JP, Böck BC, et el. Histone deacetylase 10 promotes autophagy-mediated cell survival. Proc Natl Acad Sci U S A. 2013 Jul 9;110(28):E2592-601.

584. Sarkar S, Krishna G, Imarisio S, et el. A rational mechanism for combination treatment of Huntington's disease using lithium and rapamycin. Hum Mol Genet. 2008 Jan 15;17(2):170-8.

585. Marazziti D, Di Pietro C, Golini E, et el. Induction of macroautophagy by overexpression of the Parkinson's disease-associated GPR37 receptor. FASEB J. 2009 Jun;23(6):1978-87.

586. Chang SH, Hong SH, Jiang HL, et el. GOLGA2/GM130, cis-Golgi matrix protein, is a

novel target of anticancer gene therapy. Mol Ther. 2012 Nov;20(11):2052-63.

587. Kobayashi S, Volden P, Timm D, et el. Transcription factor GATA4 inhibits doxorubicin-induced autophagy and cardiomyocyte death. J Biol Chem. 2010 Jan 1;285(1):793-804.

588. Carito V, Bonuccelli G, Martinez-Outschoorn UE, et el. Metabolic remodeling of the tumor microenvironment: migration stimulating factor (MSF) reprograms myofibroblasts toward lactate production, fueling anabolic tumor growth. Cell Cycle. 2012 Sep 15;11(18):3403-14.

589. Kuchay S, Duan S, Schenkein E, et el. FBXL2- and PTPL1-mediated degradation of p110-free p85β regulatory subunit controls the PI(3)K signaling cascade. Nat Cell Biol. 2013 May;15(5):472-80.

590. Kandouz M, Haidara K, Zhao J, et el. The EphB2 tumor suppressor induces autophagic cell death via concomitant activation of the ERK1/2 and PI3K pathways. Cell Cycle. 2010 Jan 15;9(2):398-407.

591. Nakagawa Y, Kuwahara K, Takemura G, et el. p300 plays a critical role in maintaining cardiac mitochondrial function and cell survival in postnatal hearts. Circ Res. 2009 Oct 9;105(8):746-54.

592. Mariño G, Pietrocola F, Eisenberg T, et el. Regulation of autophagy by cytosolic acetyl-coenzyme A. Mol Cell. 2014 Mar 6;53(5):710-25.

593. Sebti S, Prébois C, Pérez-Gracia E, et el. BAG6/BAT3 modulates autophagy by affecting EP300/p300 intracellular localization. Autophagy. 2014 Jul;10(7):1341-2.

594. Choi JC, Wu W, Muchir A, et el. Dual specificity phosphatase 4 mediates cardiomyopathy caused by lamin A/C (LMNA) gene mutation. J Biol Chem. 2012 Nov 23;287(48):40513-24.

595. Kovaleva V, Mora R, Park YJ, et el. miRNA-130a targets ATG2B and DICER1 to inhibit autophagy and trigger killing of chronic lymphocytic leukemia cells. Cancer Res. 2012 Apr 1;72(7):1763-72.

596. Lu C, Chen J, Xu HG, et el. MIR106B and MIR93 prevent removal of bacteria from epithelial cells by disrupting ATG16L1-mediated autophagy. Gastroenterology. 2014 Jan;146(1):188-99.

597. Neill T, Torres A, Buraschi S, et el. Decorin induces mitophagy in breast carcinoma cells via peroxisome proliferator-activated receptor γ coactivator-1α (PGC-1α) and mitostatin.

J Biol Chem. 2014 Feb 21;289(8):4952-68.

598. Koren I, Reem E, Kimchi A. DAP1, a novel substrate of mTOR, negatively regulates autophagy. Curr Biol. 2010 Jun 22;20(12):1093-8.

599. Jaishy B, Zhang Q, Chung HS, et el. Lipid-induced NOX2 activation inhibits autophagic flux by impairing lysosomal enzyme activity. J Lipid Res. 2015 Mar;56(3):546-61.

600. Balan M, Pal S. A novel CXCR3-B chemokine receptor-induced growth-inhibitory signal in cancer cells is mediated through the regulation of Bach-1 protein and Nrf2 protein nuclear translocation. J Biol Chem. 2014 Feb 7;289(6):3126-37.

601. Petherick KJ, Williams AC, Lane JD, et el. Autolysosomal β-catenin degradation regulates Wnt-autophagy-p62 crosstalk. EMBO J. 2013 Jul 3;32(13):1903-16.

602. Shtutman M, Baig M, Levina E, et el. Tumor-specific silencing of COPZ2 gene encoding coatomer protein complex subunit ζ 2 renders tumor cells dependent on its paralogous gene COPZ1. Proc Natl Acad Sci U S A. 2011 Jul 26;108(30):12449-54.

603. Chen YF, Kao CH, Chen YT, et el. Cisd2 deficiency drives premature aging and causes mitochondria-mediated defects in mice. Genes Dev. 2009 May 15;23(10):1183-94.

604. Sohn YS, Tamir S, Song L, et el. NAF-1 and mitoNEET are central to human breast cancer proliferation by maintaining mitochondrial homeostasis and promoting tumor growth. Proc Natl Acad Sci U S A. 2013 Sep 3;110(36):14676-81.

605. Puustinen P, Rytter A, Mortensen M, et el. CIP2A oncoprotein controls cell growth and autophagy through mTORC1 activation. J Cell Biol. 2014 Mar 3;204(5):713-27.

606. Abreu MM, Sealy L. The C/EBPbeta isoform, liver-inhibitory protein (LIP), induces autophagy in breast cancer cell lines. Exp Cell Res. 2010 Nov 15;316(19):3227-38.

607. Tavera-Mendoza L, Wang TT, Lallemant B, et el. Convergence of vitamin D and retinoic acid signalling at a common hormone response element. EMBO Rep. 2006 Feb;7(2):180-5.

608. Zhang Y, Xu M, Xia M, et el. Defective autophagosome trafficking contributes to impaired autophagic flux in coronary arterial myocytes lacking CD38 gene. Cardiovasc Res. 2014 Apr 1;102(1):68-78.

609. Brown NE, Jeselsohn R, Bihani T, et el. Cyclin D1 activity regulates autophagy and senescence in the mammary epithelium. Cancer Res. 2012 Dec 15;72(24):6477-89.

610. Han J, Hou W, Goldstein LA, et el. A Complex between Atg7 and Caspase-9: A NOVEL

MECHANISM OF CROSS-REGULATION BETWEEN AUTOPHAGY AND APOPTOSIS. J Biol Chem. 2014 Mar 7;289(10):6485-97.

611. Tiwari M, Lopez-Cruzan M, Morgan WW, et el. Loss of caspase-2-dependent apoptosis induces autophagy after mitochondrial oxidative stress in primary cultures of young adult cortical neurons. J Biol Chem. 2011 Mar 11;286(10):8493-506.

612. Farg MA, Sundaramoorthy V, Sultana JM, et el. C9ORF72, implicated in amytrophic lateral sclerosis and frontotemporal dementia, regulates endosomal trafficking. Hum Mol Genet. 2014 Jul 1;23(13):3579-95.

613. Zou J, Li W, Misra A, et el. The viral restriction factor tetherin prevents leucine-rich pentatricopeptide repeat-containing protein (LRPPRC) from association with beclin 1 and B-cell CLL/lymphoma 2 (Bcl-2) and enhances autophagy and mitophagy. J Biol Chem. 2015 Mar 13;290(11):7269-79.

614. Fan S, Meng Q, Saha T, et el. Low concentrations of diindolylmethane, a metabolite of indole-3-carbinol, protect against oxidative stress in a BRCA1-dependent manner. Cancer Res. 2009 Aug 1;69(15):6083-91.

615. Esteve JM, Armengod ME, Knecht E. BRCA1 negatively regulates formation of autophagic vacuoles in MCF-7 breast cancer cells. Exp Cell Res. 2010 Oct 1;316(16):2618-29.

616. Contreras AU, Mebratu Y, Delgado M, et el. Deacetylation of p53 induces autophagy by suppressing Bmf expression. J Cell Biol. 2013 Apr 29;201(3):427-37.

617. Meng N, Wu L, Gao J, et el. Lipopolysaccharide induces autophagy through BIRC2 in human umbilical vein endothelial cells. J Cell Physiol. 2010 Oct;225(1):174-9.

618. Chen S, Zhang Y, Zhou L, et el. A Bim-targeting strategy overcomes adaptive bortezomib resistance in myeloma through a novel link between autophagy and apoptosis. Blood. 2014 Oct 23;124(17):2687-97.

619. Yee KS, Wilkinson S, James J, et el. PUMA- and Bax-induced autophagy contributes to apoptosis. Cell Death Differ. 2009 Aug;16(8):1135-45.

620. Hamidi T, Cano CE, Grasso D, et el. Nupr1-aurora kinase A pathway provides protection against metabolic stress-mediated autophagic-associated cell death. Clin Cancer Res. 2012 Oct 1;18(19):5234-46.

621. Kett LR, Stiller B, Bernath MM, et el. α-Synuclein-independent histopathological and motor deficits in mice lacking the endolysosomal Parkinsonism protein Atp13a2. J

Neurosci. 2015 Apr 8;35(14):5724-42.

622. Rajadas J, Sun W, Li H, et el. Enhanced Aβ(1-40) production in endothelial cells stimulated with fibrillar Aβ(1-42). PLoS One. 2013;8(3):e58194.

623. Schnöder L, Hao W, Qin Y, et el. Deficiency of Neuronal p38α MAPK Attenuates Amyloid Pathology in Alzheimer Disease Mouse and Cell Models through Facilitating Lysosomal Degradation of BACE1. J Biol Chem. 2016 Jan 29;291(5):2067-79.

624. Zhaorigetu S, Yang Z, Toma I, et el. Apolipoprotein L6, induced in atherosclerotic lesions, promotes apoptosis and blocks Beclin 1-dependent autophagy in atherosclerotic cells. J Biol Chem. 2011 Aug 5;286(31):27389-98.

625. Fu M, St-Pierre P, Shankar J, et el. Regulation of mitophagy by the Gp78 E3 ubiquitin ligase. Mol Biol Cell. 2013 Apr;24(8):1153-62.

626. Song YM, Song SO, You YH, et el. Glycated albumin causes pancreatic β-cells dysfunction through autophagy dysfunction. Endocrinology. 2013 Aug;154(8):2626-39.

627. Porrello ER, D'Amore A, Curl CL, et el. Angiotensin II type 2 receptor antagonizes angiotensin II type 1 receptor-mediated cardiomyocyte autophagy. Hypertension. 2009 Jun;53(6):1032-40.

628. Essick EE, Wilson RM, Pimentel DR, et el. Adiponectin modulates oxidative stress-induced autophagy in cardiomyocytes. PLoS One. 2013 Jul 19;8(7):e68697.

629. Kang R, Tang D, Schapiro NE, et el. The receptor for advanced glycation end products (RAGE) sustains autophagy and limits apoptosis, promoting pancreatic tumor cell survival. Cell Death Differ. 2010 Apr;17(4):666-76.

630. Hu P, Lai D, Lu P, et el. ERK and Akt signaling pathways are involved in advanced glycation end product-induced autophagy in rat vascular smooth muscle cells. Int J Mol Med. 2012 Apr;29(4):613-8.

631. Zhang J, Xu D, Nie J, et el. Comparative gene identification-58 (CGI-58) promotes autophagy as a putative lysophosphatidylglycerol acyltransferase. J Biol Chem. 2014 Nov 21;289(47):33044-53.

632. Atlashkin V, Kreykenbohm V, Eskelinen EL, et el. Deletion of the SNARE vti1b in mice results in the loss of a single SNARE partner, syntaxin 8. Mol Cell Biol. 2003 Aug;23(15):5198-207.

633. Tresse E, Salomons FA, Vesa J, et el. VCP/p97 is essential for maturation of ubiquitin-containing autophagosomes and this function is impaired by mutations that

cause IBMPFD. Autophagy. 2010 Feb;6(2):217-27.

634. Gonzalez MA, Feely SM, Speziani F, et el. A novel mutation in VCP causes Charcot-Marie-Tooth Type 2 disease. Brain. 2014 Nov;137(Pt 11):2897-902.

635. de la Cruz-Morcillo MA, Valero ML, Callejas-Valera JL, et el. P38MAPK is a major determinant of the balance between apoptosis and autophagy triggered by 5-fluorouracil: implication in resistance. Oncogene. 2012 Mar 1;31(9):1073-85.

636. Mei S, Gu H, Ward A, et el. p38 mitogen-activated protein kinase (MAPK) promotes cholesterol ester accumulation in macrophages through inhibition of macroautophagy. J Biol Chem. 2012 Apr 6;287(15):11761-8.

637. Gomes LC, Scorrano L. High levels of Fis1, a pro-fission mitochondrial protein, trigger autophagy. Biochim Biophys Acta. 2008 Jul-Aug;1777(7-8):860-6.

638. Zhao YG, Zhao H, Miao L, et el. The p53-induced gene Ei24 is an essential component of the basal autophagy pathway. J Biol Chem. 2012 Dec 7;287(50):42053-63.

639. Liu J, Xia H, Kim M, et el. Beclin1 controls the levels of p53 by regulating the deubiquitination activity of USP10 and USP13. Cell. 2011 Sep 30;147(1):223-34.

640. Amaya C, Militello RD, Calligaris SD, et el. Rab24 interacts with the Rab7/Rab interacting lysosomal protein complex to regulate endosomal degradation. Traffic. 2016 Nov;17(11):1181-1196.

641. Harvey AJ, Pennington CJ, Porter S, et el. Brk protects breast cancer cells from autophagic cell death induced by loss of anchorage. Am J Pathol. 2009 Sep;175(3):1226-34.

642. Yu Z, Ma J, Li X, et el. Autophagy defects and related genetic variations in renal cell carcinoma with eosinophilic cytoplasmic inclusions. Sci Rep. 2018 Jul 2;8(1):9972.

643. Ohshiro K, Rayala SK, El-Naggar AK, et el. Delivery of cytoplasmic proteins to autophagosomes. Autophagy. 2008 Jan;4(1):104-6.

644. Al-Maskari M, Care MA, Robinson E, et el. Site-1 protease function is essential for the generation of antibody secreting cells and reprogramming for secretory activity. Sci Rep. 2018 Sep 25;8(1):14338.

645. Wu H, Zhu H, Liu DX, et el. Silencing of elongation factor-2 kinase potentiates the effect of 2-deoxy-D-glucose against human glioma cells through blunting of autophagy. Cancer Res. 2009 Mar 15;69(6):2453-60.

646. Li C, Imai M, Hasegawa S, et el. Growth Inhibition of Refractory Human Gallbladder Cancer Cells by Retinol, and Its Mechanism of Action. Biol Pharm Bull. 2017 Apr 1;40(4):495-503.

647. Khan SY, Vasanth S, Kabir F, et el. FOXE3 contributes to Peters anomaly through transcriptional regulation of an autophagy-associated protein termed DNAJB1. Nat Commun. 2016 Apr 6;7:10953.

648. He HY, Ren L, Guo T, et el. Neuronal autophagy aggravates microglial inflammatory injury by downregulating CX3CL1/fractalkine after ischemic stroke. Neural Regen Res. 2019 Feb;14(2):280-288.

649. Liu J, Copland DA, Theodoropoulou S, et el. Impairing autophagy in retinal pigment epithelium leads to inflammasome activation and enhanced macrophage-mediated angiogenesis. Sci Rep. 2016 Feb 5;6:20639.

650. Cho B, Choi SY, Park OH, et el. Differential expression of BNIP family members of BH3-only proteins during the development and after axotomy in the rat. Mol Cells. 2012 Jun;33(6):605-10.

651. Chen HE, Lin JF, Tsai TF, et el. Allyl Isothiocyanate Induces Autophagy through the Up-Regulation of Beclin-1 in Human Prostate Cancer Cells. Am J Chin Med. 2018 Oct 4:1-19.

652. Sanghavi P, Koenig H. Autophagy-related changes of arylsulphatases A and B in rat liver lysosomes. Biochem J. 1976 Jun 1;155(3):725-8.

653. Vantaggiato C, Clementi E, Bassi MT. ZFYVE26/SPASTIZIN: a close link between complicated hereditary spastic paraparesis and autophagy. Autophagy. 2014 Feb;10(2):374-5.

654. Noiret M, Hardy S, Audic Y. zfp36 expression delineates both myeloid cells and cells localized to the fusing neural folds in Xenopus tropicalis. Int J Dev Biol. 2014;58(10-12):751-5.

655. Feng L, Ma Y, Sun J, et el. YY1-MIR372-SQSTM1 regulatory axis in autophagy. Autophagy. 2014 Aug;10(8):1442-53.

656. Guo F, Jiao D, Sui GQ, et el. Anticancer effect of YWHAZ silencing via inducing apoptosis and autophagy in gastric cancer cells. Neoplasma. 2018 Sep 19;65(5):693-700.

657. Miyamoto M, Takano M, Aoyama T, et el. Phenoxodiol Increases Cisplatin Sensitivity in Ovarian Clear Cancer Cells Through XIAP Down-regulation and Autophagy Inhibition.

Anticancer Res. 2018 Jan;38(1):301-306.

658. Feidantsis K, Pörtner HO, Vlachonikola E, et el. Seasonal Changes in Metabolism and Cellular Stress Phenomena in the Gilthead Sea Bream (Sparus aurata). Physiol Biochem Zool. 2018 May/Jun;91(3):878-895.

659. Sun PH, Zhu LM, Qiao MM, et el. The XAF1 tumor suppressor induces autophagic cell death via upregulation of Beclin-1 and inhibition of Akt pathway. Cancer Lett. 2011 Nov 28;310(2):170-80.

660. Tsai CW, Lai FJ, Sheu HM, et el. WWOX suppresses autophagy for inducing apoptosis in methotrexate-treated human squamous cell carcinoma. Cell Death Dis. 2013 Sep 5;4:e792.

661. Maity J, Bohr VA, Laskar A, et el. Transient overexpression of Werner protein rescues starvation induced autophagy in Werner syndrome cells. Biochim Biophys Acta. 2014 Dec;1842(12 Pt A):2387-94.

662. Lock R, Kenific CM, Leidal AM, et el. Autophagy-dependent production of secreted factors facilitates oncogenic RAS-driven invasion. Cancer Discov. 2014 Apr;4(4):466-79.

663. Wu X, Zhang J, Ma C, et el. A role for Wnt/β-catenin signalling in suppressing Bacillus Calmette-Guerin-induced macrophage autophagy. Microb Pathog. 2018 Dec 11;127:277-287.

664. Ortiz-Masiá D, Cosín-Roger J, Calatayud S, et el. Hypoxic macrophages impair autophagy in epithelial cells through Wnt1: relevance in IBD. Mucosal Immunol. 2014 Jul;7(4):929-38.

665. Willén K, Edgar JR, Hasegawa T, et el. Aβ accumulation causes MVB enlargement and is modelled by dominant negative VPS4A. Mol Neurodegener. 2017 Aug 23;12(1):61.

666. Zavodszky E, Seaman MN, Rubinsztein DC. VPS35 Parkinson mutation impairs autophagy via WASH. Cell Cycle. 2014;13(14):2155-6.

667. Munteanu I, Kalimo H, Saraste A, et el. Cardiac autophagic vacuolation in severe X-linked myopathy with excessive autophagy. Neuromuscul Disord. 2017 Feb;27(2):185-187.

668. Mikhaylova O, Stratton Y, Hall D, et el. VHL-regulated MiR-204 suppresses tumor growth through inhibition of LC3B-mediated autophagy in renal clear cell carcinoma. Cancer Cell. 2012 Apr 17;21(4):532-46.

669. Stanton MJ, Dutta S, Zhang H, et el. Autophagy control by the VEGF-C/NRP-2 axis in

cancer and its implication for treatment resistance. Cancer Res. 2013 Jan 1;73(1):160-71.

670. Joachim J, Wirth M, McKnight NC, et el. Coiling up with SCOC and WAC: two new regulators of starvation-induced autophagy. Autophagy. 2012 Sep;8(9):1397-400.

671. Xu Y, Her C. VBP1 facilitates proteasome and autophagy-mediated degradation of MutS homologue hMSH4. FASEB J. 2013 Dec;27(12):4799-810.

672. Moreau K, Fleming A, Imarisio S, et el. PICALM modulates autophagy activity and tau accumulation. Nat Commun. 2014 Sep 22;5:4998.

673. Schulze U, Vollenbröker B, Braun DA, et el. The Vac14-interaction network is linked to regulators of the endolysosomal and autophagic pathway. Mol Cell Proteomics. 2014 Jun;13(6):1397-411.

674. Hasnat M, Yuan Z, Naveed M, et el. Drp1-associated mitochondrial dysfunction and mitochondrial autophagy: a novel mechanism in triptolide-induced hepatotoxicity. Cell Biol Toxicol. 2018 Dec 13.

675. Sun J, Hu Q, Peng H, et el. The ubiquitin-specific protease USP8 deubiquitinates and stabilizes Cx43. J Biol Chem. 2018 May 25;293(21):8275-8284.

676. Taillebourg E, Gregoire I, Viargues P, et el. The deubiquitinating enzyme USP36 controls selective autophagy activation by ubiquitinated proteins. Autophagy. 2012 May 1;8(5):767-79.

677. Liang JX, Ning Z, Gao W, et el. Ubiquitin-specific protease 22-induced autophagy is correlated with poor prognosis of pancreatic cancer. Oncol Rep. 2014 Dec;32(6):2726-34.

678. Cornelissen T, Haddad D, Wauters F, et el. The deubiquitinase USP15 antagonizes Parkin-mediated mitochondrial ubiquitination and mitophagy. Hum Mol Genet. 2014 Oct 1;23(19):5227-42.

679. Giussani P, Bassi R, Anelli V, et el. Glucosylceramide synthase protects glioblastoma cells against autophagic and apoptotic death induced by temozolomide and Paclitaxel. Cancer Invest. 2012 Jan;30(1):27-37.

680. Watanabe H, Bohensky J, Freeman T, et el. Hypoxic induction of UCP3 in the growth plate: UCP3 suppresses chondrocyte autophagy. J Cell Physiol. 2008 Aug;216(2):419-25.

681. Lu X, Altshuler-Keylin S, Wang Q, et el. Mitophagy controls beige adipocyte maintenance through a Parkin-dependent and UCP1-independent mechanism. Sci Signal. 2018 Apr 24;11(527).

682. Zhang D, Han S, Wang S, et el. cPKCγ-mediated down-regulation of UCHL1 alleviates ischaemic neuronal injuries by decreasing autophagy via ERK-mTOR pathway. J Cell Mol Med. 2017 Dec;21(12):3641-3657.

683. Lee DY, Arnott D, Brown EJ. Ubiquilin4 is an adaptor protein that recruits Ubiquilin1 to the autophagy machinery. EMBO Rep. 2013 Apr;14(4):373-81.

684. Wu Q, Liu M, Huang C, et el. Pathogenic Ubqln2 gains toxic properties to induce neuron death. Acta Neuropathol. 2015 Mar;129(3):417-28.

685. Chen S, Wang C, Sun L, et el. RAD6 promotes homologous recombination repair by activating the autophagy-mediated degradation of heterochromatin protein HP1. Mol Cell Biol. 2015 Jan;35(2):406-16.

686. Cao H, Xie J, Guo L, et el. All-trans retinoic acid induces autophagic degradation of ubiquitin-like modifier activating enzyme 3 in acute promyelocytic leukemia cells. Leuk Lymphoma. 2018 May;59(5):1222-1230.

687. Chang TK, Shravage BV, Hayes SD, et el. Uba1 functions in Atg7- and Atg3-independent autophagy. Nat Cell Biol. 2013 Sep;15(9):1067-78.

688. Wang J, Wang J, Wang JJ, et el. [Role of autophagy in TXNIP overexpression-induced apoptosis of INS-1 islet cells]. Sheng Li Xue Bao. 2017 Aug 25;69(4):445-451.

689. Zhang SF, Wang XY, Fu ZQ, et el. TXNDC17 promotes paclitaxel resistance via inducing autophagy in ovarian cancer. Autophagy. 2015;11(2):225-38.

690. Zhang Z, Wang A, Li H, et el. STAT3-dependent TXNDC17 expression mediates Taxol resistance through inducing autophagy in human colorectal cancer cells. Gene. 2016 Jun 10;584(1):75-82.

691. Lei H, Wang G, Zhang J, et el. Inhibiting TrxR suppresses liver cancer by inducing apoptosis and eliciting potent antitumor immunity. Oncol Rep. 2018 Dec;40(6):3447-3457.

692. Li H, Zhang Y, Cao L, et el. Curcumin could reduce the monomer of TTR with Tyr114Cys mutation via autophagy in cell model of familial amyloid polyneuropathy. Drug Des Devel Ther. 2014 Oct 31;8:2121-8.

693. Zhang F, Hu C, Dong Y, et el. The impact of V30A mutation on transthyretin protein structural stability and cytotoxicity against neuroblastoma cells. Arch Biochem Biophys. 2013 Jul 15;535(2):120-7.

694. Kaizuka T, Hara T, Oshiro N, et el. Tti1 and Tel2 are critical factors in mammalian target

of rapamycin complex assembly. J Biol Chem. 2010 Jun 25;285(26):20109-16.

695. Amantini C, Farfariello V, Cardinali C, et el. The TRPV1 ion channel regulates thymocyte differentiation by modulating autophagy and proteasome activity. Oncotarget. 2017 Oct 11;8(53):90766-90780.

696. Kondratskyi A, Yassine M, Kondratska K, et el. Calcium-permeable ion channels in control of autophagy and cancer. Front Physiol. 2013 Oct 2;4:272.

697. Cost NG, Czyzyk-Krzeska MF. Regulation of autophagy by two products of one gene: TRPM3 and miR-204. Mol Cell Oncol. 2015 Jan 23;2(4):e1002712.

698. Jiang Q, Gao Y, Wang C, et el. Nitration of TRPM2 as a Molecular Switch Induces Autophagy During Brain Pericyte Injury. Antioxid Redox Signal. 2017 Dec 1;27(16):1297-1316.

699. Zhang L, Dai F, Cui L, et el. Novel role for TRPC4 in regulation of macroautophagy by a small molecule in vascular endothelial cells. Biochim Biophys Acta. 2015 Feb;1853(2):377-87.

700. Du GJ, Li JH, Liu WJ, et el. The combination of TRPM8 and TRPA1 expression causes an invasive phenotype in lung cancer. Tumour Biol. 2014 Feb;35(2):1251-61.

701. Li J, Wang P, Xie Z, et el. Elevated TRAF4 expression impaired LPS-induced autophagy in mesenchymal stem cells from ankylosing spondylitis patients. Exp Mol Med. 2017 Jun 9;49(6):e343.

702. Bae SY, Byun S, Bae SH, et el. TPT1 (tumor protein, translationally-controlled 1) negatively regulates autophagy through the BECN1 interactome and an MTORC1-mediated pathway. Autophagy. 2017 May 4;13(5):820-833.

703. Vidal-Donet JM, Cárcel-Trullols J, Casanova B, et el. Alterations in ROS activity and lysosomal pH account for distinct patterns of macroautophagy in LINCL and JNCL fibroblasts. PLoS One. 2013;8(2):e55526.

704. Ibar C, Cataldo VF, Vásquez-Doorman C, et el. Drosophila p53-related protein kinase is required for PI3K/TOR pathway-dependent growth. Development. 2013 Mar;140(6):1282-91.

705. Lin M, Chang Y, Xie F, et el. ASPP2 Inhibits the Profibrotic Effects of Transforming Growth Factor-β1 in Hepatic Stellate Cells by Reducing Autophagy. Dig Dis Sci. 2018 Jan;63(1):146-154.

706. Gou WF, Shen DF, Yang XF, et el. ING5 suppresses proliferation, apoptosis, migration

and invasion, and induces autophagy and differentiation of gastric cancer cells: a good marker for carcinogenesis and subsequent progression. Oncotarget. 2015 Aug 14;6(23):19552-79.

707. Bertolin G, Ferrando-Miguel R, Jacoupy M, et el. The TOMM machinery is a molecular switch in PINK1 and PARK2/PARKIN-dependent mitochondrial clearance. Autophagy. 2013 Nov 1;9(11):1801-17.

708. Tumbarello DA, Waxse BJ, Arden SD, et el. Autophagy receptors link myosin VI to autophagosomes to mediate Tom1-dependent autophagosome maturation and fusion with the lysosome. Nat Cell Biol. 2012 Oct;14(10):1024-35.

709. Jones S, Cunningham DL, Rappoport JZ, et el. The non-receptor tyrosine kinase Ack1 regulates the fate of activated EGFR by inducing trafficking to the p62/NBR1 pre-autophagosome. J Cell Sci. 2014 Mar 1;127(Pt 5):994-1006.

710. Bhatnagar S, Mittal A, Gupta SK, et el. TWEAK causes myotube atrophy through coordinated activation of ubiquitin-proteasome system, autophagy, and caspases. J Cell Physiol. 2012 Mar;227(3):1042-51.

711. Chang LC, Hsieh MT, Yang JS, et el. Effect of bis(hydroxymethyl) alkanoate curcuminoid derivative MTH-3 on cell cycle arrest, apoptotic and autophagic pathway in triple-negative breast adenocarcinoma MDA-MB-231 cells: An in vitro study. Int J Oncol. 2018 Jan;52(1):67-76.

712. Wang C, Ma Y, Hu Q, et el. Bifidobacterial recombinant thymidine kinase-ganciclovir gene therapy system induces FasL and TNFR2 mediated antitumor apoptosis in solid tumors. BMC Cancer. 2016 Jul 27;16:545.

713. Azevedo FV, Lopes DS, Cirilo Gimenes SN, et el. Human breast cancer cell death induced by BnSP-6, a Lys-49 PLA2 homologue from Bothrops pauloensis venom. Int J Biol Macromol. 2016 Jan;82:671-7.

714. Chen K, Dai H, Yuan J, et el. Optineurin-mediated mitophagy protects renal tubular epithelial cells against accelerated senescence in diabetic nephropathy. Cell Death Dis. 2018 Jan 24;9(2):105.

715. Lim SC, Jeon HJ, Kee KH, et el. Involvement of DR4/JNK pathway-mediated autophagy in acquired TRAIL resistance in HepG2 cells. Int J Oncol. 2016 Nov;49(5):1983-1990.

716. Niture S, Ramalinga M, Kedir H, et el. TNFAIP8 promotes prostate cancer cell survival by inducing autophagy. Oncotarget. 2018 Jun 1;9(42):26884-26899.

717. Matsuzawa Y, Oshima S, Takahara M, et el. TNFAIP3 promotes survival of CD4 T cells by restricting MTOR and promoting autophagy. Autophagy. 2015;11(7):1052-62.

718. Zhao Y, Hu J, Miao G, et el. Transmembrane protein 208: a novel ER-localized protein that regulates autophagy and ER stress. PLoS One. 2013 May 14;8(5):e64228.

719. Shyu RY, Wang CH, Wu CC, et el. Tazarotene-Induced Gene 1 Enhanced Cervical Cell Autophagy through Transmembrane Protein 192. Mol Cells. 2016 Dec;39(12):877-887.

720. Campbell GR, Rawat P, Bruckman RS, et el. Human Immunodeficiency Virus Type 1 Nef Inhibits Autophagy through Transcription Factor EB Sequestration. PLoS Pathog. 2015 Jun 26;11(6):e1005018.

721. Chuang SY, Yang CH, Chou CC, et el. TLR-induced PAI-2 expression suppresses IL-1β processing via increasing autophagy and NLRP3 degradation. Proc Natl Acad Sci U S A. 2013 Oct 1;110(40):16079-84.

722. Fiorentino L, Cavalera M, Menini S, et el. Loss of TIMP3 underlies diabetic nephropathy via FoxO1/STAT1 interplay. EMBO Mol Med. 2013 Mar;5(3):441-55.

723. Feng J, Chen K, Xia Y, et el. Salidroside ameliorates autophagy and activation of hepatic stellate cells in mice via NF-κB and TGF-β1/Smad3 pathways. Drug Des Devel Ther. 2018 Jun 22;12:1837-1853.

724. Carrascoso I, Alcalde J, Tabas-Madrid D, et el. Transcriptome-wide analysis links the short-term expression of the b isoforms of TIA proteins to protective proteostasis-mediated cell quiescence response. PLoS One. 2018 Dec 11;13(12):e0208526.

725. Yeo SY, Itahana Y, Guo AK, et el. Transglutaminase 2 contributes to a TP53-induced autophagy program to prevent oncogenic transformation. Elife. 2016 Mar 9;5:e07101.

726. Ahmed M, Lai TH, Zada S, et el. Functional Linkage of RKIP to the Epithelial to Mesenchymal Transition and Autophagy during the Development of Prostate Cancer. Cancers (Basel). 2018 Aug 16;10(8).

727. Choi SI, Maeng YS, Kim KS, et el. Autophagy is induced by raptor degradation via the ubiquitin/proteasome system in granular corneal dystrophy type 2. Biochem Biophys Res Commun. 2014 Aug 8;450(4):1505-11.

728. Zhang Y, Tang H, Yuan X, et el. TGF-β3 Promotes MUC5AC Hyper-Expression by Modulating Autophagy Pathway in Airway Epithelium. EBioMedicine. 2018 Jul;33:242-252.

729. Ding LY, Chu M, Jiao YS, et el. TFDP3 regulates the apoptosis and autophagy in breast cancer cell line MDA-MB-231. PLoS One. 2018 Sep 20;13(9):e0203833.

730. Yang Q, Li X, Li R, et el. Low Shear Stress Inhibited Endothelial Cell Autophagy Through TET2 Downregulation. Ann Biomed Eng. 2016 Jul;44(7):2218-27.

731. Covone AE, Fiorillo C, Acquaviva M, et el. WES in a family trio suggests involvement of TECPR2 in a complex form of progressive motor neuron disease. Clin Genet. 2016 Aug;90(2):182-5.

732. Darvekar SR, Elvenes J, Brenne HB, et el. SPBP is a sulforaphane induced transcriptional coactivator of NRF2 regulating expression of the autophagy receptor p62/SQSTM1. PLoS One. 2014 Jan 9;9(1):e85262.

733. Toyofuku T, Morimoto K, Sasawatari S, et el. Leucine-Rich Repeat Kinase 1 Regulates Autophagy through Turning On TBC1D2-Dependent Rab7 Inactivation. Mol Cell Biol. 2015 Sep 1;35(17):3044-58.

734. Yamano K, Fogel AI, Wang C, et el. Mitochondrial Rab GAPs govern autophagosome biogenesis during mitophagy. Elife. 2014 Feb 25;3:e01612.

735. Tumbarello DA, Manna PT, Allen M, et el. The Autophagy Receptor TAX1BP1 and the Molecular Motor Myosin VI Are Required for Clearance of Salmonella Typhimurium by Autophagy. PLoS Pathog. 2015 Oct 9;11(10):e1005174.

736. Feng L, Zhang J, Zhu N, et el. Ubiquitin ligase SYVN1/HRD1 facilitates degradation of the SERPINA1 Z variant/α-1-antitrypsin Z variant via SQSTM1/p62-dependent selective autophagy. Autophagy. 2017 Apr 3;13(4):686-702.

737. Cho SJ, Yun SM, Jo C, et el. SUMO1 promotes Aβ production via the modulation of autophagy. Autophagy. 2015;11(1):100-12.

738. Rivera-Monroy J, Musiol L, Unthan-Fechner K, et el. Mice lacking WRB reveal differential biogenesis requirements of tail-anchored proteins in vivo. Sci Rep. 2016 Dec 21;6:39464.

739. Hu J, Wang S, Xiong Z, et el. Exosomal Mst1 transfer from cardiac microvascular endothelial cells to cardiomyocytes deteriorates diabetic cardiomyopathy. Biochim Biophys Acta Mol Basis Dis. 2018 Nov;1864(11):3639-3649.

740. Wilkinson DS, Jariwala JS, Anderson E, et el. Phosphorylation of LC3 by the Hippo kinases STK3/STK4 is essential for autophagy. Mol Cell. 2015 Jan 8;57(1):55-68.

741. Lake AD, Novak P, Hardwick RN, et el. The adaptive endoplasmic reticulum stress

response to lipotoxicity in progressive human nonalcoholic fatty liver disease. Toxicol Sci. 2014 Jan;137(1):26-35.

742. Demetriadou A, Morales-Sanfrutos J, Nearchou M, et el. Mouse Stbd1 is N-myristoylated and affects ER-mitochondria association and mitochondrial morphology. J Cell Sci. 2017 Mar 1;130(5):903-915.

743. Qin C, Liu Q, Hu ZW, et el. Microglial TLR4-dependent autophagy induces ischemic white matter damage via STAT1/6 pathway. Theranostics. 2018 Oct 29;8(19):5434-5451.

744. Kowalski E, Geng S, Rathes A, et el. Toll-interacting protein differentially modulates HIF1α and STAT5-mediated genes in fibroblasts. J Biol Chem. 2018 Aug 3;293(31):12239-12247.

745. Watanabe Y, Tanaka M. p62/SQSTM1 in autophagic clearance of a non-ubiquitylated substrate. J Cell Sci. 2011 Aug 15;124(Pt 16):2692-701.

746. Yuan X, Du J, Hua S, et el. Suppression of autophagy augments the radiosensitizing effects of STAT3 inhibition on human glioma cells. Exp Cell Res. 2015 Jan 15;330(2):267-76.

747. Matarrese P, Garofalo T, Manganelli V, et el. Evidence for the involvement of GD3 ganglioside in autophagosome formation and maturation. Autophagy. 2014 May;10(5):750-65.

748. Moon SY, Kim HS, Nho KW, et el. Endoplasmic reticulum stress induces epithelial-mesenchymal transition through autophagy via activation of c-Src kinase. Nephron Exp Nephrol. 2014;126(3):127-40.

749. Xiong Y, Lee HJ, Mariko B, et el. Sphingosine kinases are not required for inflammatory responses in macrophages. J Biol Chem. 2013 Nov 8;288(45):32563-73.

750. Vantaggiato C, Panzeri E, Castelli M, et el. ZFYVE26/SPASTIZIN and SPG11/SPATACSIN mutations in hereditary spastic paraplegia types AR-SPG15 and AR-SPG11 have different effects on autophagy and endocytosis. Autophagy. 2019 Jan;15(1):34-57.

751. Peixoto E, Atorrasagasti C, Aquino JB, et el. SPARC (secreted protein acidic and rich in cysteine) knockdown protects mice from acute liver injury by reducing vascular endothelial cell damage. Gene Ther. 2015 Jan;22(1):9-19.

752. Li N, Li X, Li S, et el. Cisplatin-induced downregulation of SOX1 increases drug resistance by activating autophagy in non-small cell lung cancer cell. Biochem Biophys

Res Commun. 2013 Sep 20;439(2):187-90.

753. Amengual J, Guo L, Strong A, et el. Autophagy Is Required for Sortilin-Mediated Degradation of Apolipoprotein B100. Circ Res. 2018 Feb 16;122(4):568-582.

754. Knævelsrud H, Carlsson SR, Simonsen A. SNX18 tubulates recycling endosomes for autophagosome biogenesis. Autophagy. 2013 Oct;9(10):1639-41.

755. Poehler AM, Xiang W, Spitzer P, et el. Autophagy modulates SNCA/α-synuclein release, thereby generating a hostile microenvironment. Autophagy. 2014;10(12):2171-92.

756. Fan JD, Lei PJ, Zheng JY, et el. The selective activation of p53 target genes regulated by SMYD2 in BIX-01294 induced autophagy-related cell death. PLoS One. 2015 Jan 6;10(1):e0116782.

757. Jimenez-Sanchez M, Menzies FM, Chang YY, et el. The Hedgehog signalling pathway regulates autophagy. Nat Commun. 2012;3:1200.

758. Bultman SJ, Holley DW, G de Ridder G, et el. BRG1 and BRM SWI/SNF ATPases redundantly maintain cardiomyocyte homeostasis by regulating cardiomyocyte mitophagy and mitochondrial dynamics in vivo. Cardiovasc Pathol. 2016 May-Jun;25(3):258-269.

759. Gozes I. Sexual divergence in activity-dependent neuroprotective protein impacting autism, schizophrenia, and Alzheimer's disease. J Neurosci Res. 2017 Jan 2;95(1-2):652-660.

760. Lien SC, Chang SF, Lee PL, et el. Mechanical regulation of cancer cell apoptosis and autophagy: roles of bone morphogenetic protein receptor, Smad1/5, and p38 MAPK. Biochim Biophys Acta. 2013 Dec;1833(12):3124-3133.

761. Yang N, Dang S, Shi J, et el. Caffeic acid phenethyl ester attenuates liver fibrosis via inhibition of TGF-β1/Smad3 pathway and induction of autophagy pathway. Biochem Biophys Res Commun. 2017 Apr 22;486(1):22-28.

762. Cao J, Xie H, Sun Y, et el. Sevoflurane post-conditioning reduces rat myocardial ischemia reperfusion injury through an increase in NOS and a decrease in phopshorylated NHE1 levels. Int J Mol Med. 2015 Dec;36(6):1529-37.

763. Kang R, Tang D. Autophagy and Ferroptosis - What's the Connection. Curr Pathobiol Rep. 2017 Jun;5(2):153-159.

764. Karunakaran S, Ramachandran S, Coothankandaswamy V, et el. SLC6A14 (ATB0,+) protein, a highly concentrative and broad specific amino acid transporter, is a novel and

effective drug target for treatment of estrogen receptor-positive breast cancer. J Biol Chem. 2011 Sep 9;286(36):31830-8.

765. Ahn HH, Oh Y, Lee H, et el. Identification of glucose-6-phosphate transporter as a key regulator functioning at the autophagy initiation step. FEBS Lett. 2015 Jul 22;589(16):2100-9.

766. Park S, Choi SG, Yoo SM, et el. Choline dehydrogenase interacts with SQSTM1/p62 to recruit LC3 and stimulate mitophagy. Autophagy. 2014;10(11):1906-20.

767. Orlotti NI, Cimino-Reale G, Borghini E, et el. Autophagy acts as a safeguard mechanism against G-quadruplex ligand-mediated DNA damage. Autophagy. 2012 Aug;8(8):1185-96.

768. Lee I, Lee SJ, Kang WK, et el. Inhibition of monocarboxylate transporter 2 induces senescence-associated mitochondrial dysfunction and suppresses progression of colorectal malignancies in vivo. Mol Cancer Ther. 2012 Nov;11(11):2342-51.

769. Long Y, Gao Z, Hu X, et el. Downregulation of MCT4 for lactate exchange promotes the cytotoxicity of NK cells in breast carcinoma. Cancer Med. 2018 Sep;7(9):4690-4700.

770. Geillinger KE, Kipp AP, Schink K, et el. Nrf2 regulates the expression of the peptide transporter PEPT1 in the human colon carcinoma cell line Caco-2. Biochim Biophys Acta. 2014 Jun;1840(6):1747-54.

771. Wei X, Li X, Yan W, et el. SKP2 Promotes Hepatocellular Carcinoma Progression Through Nuclear AMPK-SKP2-CARM1 Signaling Transcriptionally Regulating Nutrient-Deprived Autophagy Induction. Cell Physiol Biochem. 2018;47(6):2484-2497.

772. Shumin C, Wei X, Yunfeng L, et el. Genipin alleviates vascular hyperpermeability following hemorrhagic shock by up-regulation of SIRT3/autophagy. Cell Death Discov. 2018 May 9;4:52.

773. MacVicar TD, Mannack LV, Lees RM, et el. Targeted siRNA Screens Identify ER-to-Mitochondrial Calcium Exchange in Autophagy and Mitophagy Responses in RPE1 Cells. Int J Mol Sci. 2015 Jun 11;16(6):13356-80.

774. Petralia RS, Schwartz CM, Wang YX, et el. Sonic hedgehog promotes autophagy in hippocampal neurons. Biol Open. 2013 Apr 8;2(5):499-504.

775. Lépine S, Allegood JC, Park M, et el. Sphingosine-1-phosphate phosphohydrolase-1 regulates ER stress-induced autophagy. Cell Death Differ. 2011 Feb;18(2):350-61.

776. Mitroi DN, Karunakaran I, Gräler M, et el. SGPL1 (sphingosine phosphate lyase 1) modulates neuronal autophagy via phosphatidylethanolamine production. Autophagy.

2017 May 4;13(5):885-899.

777. Liu W, Wang X, Wang Y, et el. SGK1 inhibition-induced autophagy impairs prostate cancer metastasis by reversing EMT. J Exp Clin Cancer Res. 2018 Apr 2;37(1):73.

778. Yi X, Xiang L, Huang Y, et el. Apoptosis and pro-death autophagy induced by a spirostanol saponin isolated from Rohdea chinensis (Baker) N. Tanaka (synonym Tupistra chinensis Baker) on HL-60 cells. Phytomedicine. 2018 Mar 15;42:83-89.

779. Hawkins A, Guttentag SH, Deterding R, et el. A non-BRICHOS SFTPC mutant (SP-CI73T) linked to interstitial lung disease promotes a late block in macroautophagy disrupting cellular proteostasis and mitophagy. Am J Physiol Lung Cell Mol Physiol. 2015 Jan 1;308(1):L33-47.

780. Tsukahara T, Matsuda Y, Haniu H. PSF knockdown enhances apoptosis via downregulation of LC3B in human colon cancer cells. Biomed Res Int. 2013;2013:204973.

781. Gekonge B, Raymond AD, Yin X, et el. Retinoblastoma protein induction by HIV viremia or CCR5 in monocytes exposed to HIV-1 mediates protection from activation-induced apoptosis: ex vivo and in vitro study. J Leukoc Biol. 2012 Aug;92(2):397-405.

782. Kimura T, Jia J, Claude-Taupin A, et el. Cellular and molecular mechanism for secretory autophagy. Autophagy. 2017 Jun 3;13(6):1084-1085.

783. Gan-Or Z, Dion PA, Rouleau GA. Genetic perspective on the role of the autophagy-lysosome pathway in Parkinson disease. Autophagy. 2015;11(9):1443-57.

784. Dong W, Zhang P, Fu Y, et el. Roles of SATB2 in site-specific stemness, autophagy and senescence of bone marrow mesenchymal stem cells. J Cell Physiol. 2015 Mar;230(3):680-90.

785. Xiao Q, Yang Y, Qin Y, et el. AMP-activated protein kinase-dependent autophagy mediated the protective effect of sonic hedgehog pathway on oxygen glucose deprivation-induced injury of cardiomyocytes. Biochem Biophys Res Commun. 2015 Feb 13;457(3):419-25.

786. Huang YL, Chang CL, Tang CH, et el. Extrinsic sphingosine 1-phosphate activates S1P5 and induces autophagy through generating endoplasmic reticulum stress in human prostate cancer PC-3 cells. Cell Signal. 2014 Mar;26(3):611-8.

787. Chen YZ, Wang F, Wang HJ, et el. Sphingosine 1 phosphate receptor-1 (S1PR1) signaling protects cardiac function by inhibiting cardiomyocyte autophagy. J Geriatr Cardiol. 2018

May;15(5):334-345.

788. Sun W, Zheng Y, Lu Z, et el. Overexpression of S100A7 protects LPS-induced mitochondrial dysfunction and stimulates IL-6 and IL-8 in HaCaT cells. PLoS One. 2014 Mar 26;9(3):e92927.

789. Xu Q, Meng S, Liu B, et el. MicroRNA-130a regulates autophagy of endothelial progenitor cells through Runx3. Clin Exp Pharmacol Physiol. 2014 May;41(5):351-7.

790. Chen R, Jin R, Wu L, et el. Reticulon 3 attenuates the clearance of cytosolic prion aggregates via inhibiting autophagy. Autophagy. 2011 Feb;7(2):205-16.

791. Chen P, Wu JN, Shu Y, et el. Gemcitabine resistance mediated by ribonucleotide reductase M2 in lung squamous cell carcinoma is reversed by GW8510 through autophagy induction. Clin Sci (Lond). 2018 Jul 9;132(13):1417-1433.

792. Won SJ, Yen CH, Liu HS, et el. Justicidin A-induced autophagy flux enhances apoptosis of human colorectal cancer cells via class III PI3K and Atg5 pathway. J Cell Physiol. 2015 Apr;230(4):930-46.

793. Zhang F, Yan T, Guo W, et el. Novel oncogene COPS3 interacts with Beclin1 and Raf-1 to regulate metastasis of osteosarcoma through autophagy. J Exp Clin Cancer Res. 2018 Jul 3;37(1):135.

794. Heijnen HF, van Wijk R, Pereboom TC, et el. Ribosomal protein mutations induce autophagy through S6 kinase inhibition of the insulin pathway. PLoS Genet. 2014 May 29;10(5):e1004371.

795. Tang F, Wang B, Li N, et el. RNF185, a novel mitochondrial ubiquitin E3 ligase, regulates autophagy through interaction with BNIP1. PLoS One. 2011;6(9):e24367.

796. Carroll B, Mohd-Naim N, Maximiano F, et el. The TBC/RabGAP Armus coordinates Rac1 and Rab7 functions during autophagy. Dev Cell. 2013 Apr 15;25(1):15-28.

797. Lin SY, Hsieh SY, Fan YT, et el. Necroptosis promotes autophagy-dependent upregulation of DAMP and results in immunosurveillance. Autophagy. 2018;14(5):778-795.

798. Zhang L, Qin Y, Chen M. Viral strategies for triggering and manipulating mitophagy. Autophagy. 2018;14(10):1665-1673.

799. Grigaravicius P, von Deimling A, Frappart PO. RINT1 functions as a multitasking protein at the crossroads between genomic stability, ER homeostasis, and autophagy. Autophagy. 2016 Aug 2;12(8):1413-5.

800. Wang X. Destructive cellular paths underlying familial and sporadic Parkinson disease converge on mitophagy. Autophagy. 2017;13(11):1998-1999.

801. Poillet-Perez L, Xie X, Zhan L, et el. Autophagy maintains tumour growth through circulating arginine. Nature. 2018 Nov;563(7732):569-573.

802. Rosenthal AK, Gohr CM, Mitton-Fitzgerald E, et el. Autophagy modulates articular cartilage vesicle formation in primary articular chondrocytes. J Biol Chem. 2015 May 22;290(21):13028-38.

803. Yan Z, Zou H, Tian F, et el. Human rhomboid family-1 gene silencing causes apoptosis or autophagy to epithelial cancer cells and inhibits xenograft tumor growth. Mol Cancer Ther. 2008 Jun;7(6):1355-64.

804. Su JC, Tseng PH, Hsu CY, et el. RFX1-dependent activation of SHP-1 induces autophagy by a novel obatoclax derivative in hepatocellular carcinoma cells. Oncotarget. 2014 Jul 15;5(13):4909-19.

805. Yang D, Zhao Y, Liu J, et el. Protective autophagy induced by RBX1/ROC1 knockdown or CRL inactivation via modulating the DEPTOR-MTOR axis. Autophagy. 2012 Dec;8(12):1856-8.

806. Eapen VV, Haber JE. DNA damage signaling triggers the cytoplasm-to-vacuole pathway of autophagy to regulate cell cycle progression. Autophagy. 2013 Mar;9(3):440-1.

807. Di J, Tang J, Qian H, et el. p53 upregulates PLCε-IP3-Ca(2+) pathway and inhibits autophagy through its target gene Rap2B. Oncotarget. 2017 May 23;8(39):64657-64669.

808. Zhu X, Pan Q, Huang N, et el. RAD51 regulates CHK1 stability via autophagy to promote cell growth in esophageal squamous carcinoma cells. Tumour Biol. 2016 Oct 14.

809. Relic B, Charlier E, Deroyer C, et el. Serum starvation raises turnover of phosphorylated p62/SQSTM1 (Serine 349), reveals expression of proteasome and N-glycanase1 interactive protein RAD23B and sensitizes human synovial fibroblasts to BAY 11-7085-induced cell death. Oncotarget. 2018 Nov 9;9(88):35830-35843.

810. Masaracchia C, Hnida M, Gerhardt E, et el. Membrane binding, internalization, and sorting of alpha-synuclein in the cell. Acta Neuropathol Commun. 2018 Aug 14;6(1):79.

811. Guo W, Chen Z, Chen Z, et el. Promotion of Cell Proliferation through Inhibition of Cell Autophagy Signalling Pathway by Rab3IP is Restrained by MicroRNA-532-3p in Gastric Cancer. J Cancer. 2018 Oct 22;9(23):4363-4373.

812. Spang N, Feldmann A, Huesmann H, et el. RAB3GAP1 and RAB3GAP2 modulate basal

and rapamycin-induced autophagy. Autophagy. 2014;10(12):2297-309.

813. Liu Y, Tao X, Jia L, et el. Knockdown of RAB25 promotes autophagy and inhibits cell growth in ovarian cancer cells. Mol Med Rep. 2012 Nov;6(5):1006-12.

814. Haobam B, Nozawa T, Minowa-Nozawa A, et el. Rab17-mediated recycling endosomes contribute to autophagosome formation in response to Group A Streptococcus invasion. Cell Microbiol. 2014 Dec;16(12):1806-21.

815. Wang M, An S, Wang D, et el. Quantitative Proteomics Identify the Possible Tumor Suppressive Role of Protease-Activated Receptor-4 in Esophageal Squamous Cell Carcinoma Cells. Pathol Oncol Res. 2018 Mar 4.

816. Kosumi K, Masugi Y, Yang J, et el. Tumor SQSTM1 (p62) expression and T cells in colorectal cancer. Oncoimmunology. 2017 Jan 31;6(3):e1284720.

817. You L, Wang Z, Li H, et el. The role of STAT3 in autophagy. Autophagy. 2015;11(5):729-39.

818. Mocholi E, Dowling SD, Botbol Y, et el. Autophagy Is a Tolerance-Avoidance Mechanism that Modulates TCR-Mediated Signaling and Cell Metabolism to Prevent Induction of T Cell Anergy. Cell Rep. 2018 Jul 31;24(5):1136-1150.

819. Huang YH, Al-Aidaroos AQ, Yuen HF, et el. A role of autophagy in PTP4A3-driven cancer progression. Autophagy. 2014 Oct 1;10(10):1787-800.

820. Wang Z, Li L, Wang Y. Effects of Per2 overexpression on growth inhibition and metastasis, and on MTA1, nm23-H1 and the autophagy-associated PI3K/PKB signaling pathway in nude mice xenograft models of ovarian cancer. Mol Med Rep. 2016 Jun;13(6):4561-8.

821. Tuloup-Minguez V, Greffard A, Codogno P, et el. Regulation of autophagy by extracellular matrix glycoproteins in HeLa cells. Autophagy. 2011 Jan;7(1):27-39.

822. Zheng L, Zhu K, Jiao H, et el. PTHrP expression in human MDA-MB-231 breast cancer cells is critical for tumor growth and survival and osteoblast inhibition. Int J Biol Sci. 2013 Aug 21;9(8):830-41.

823. Choi J, Jo M, Lee E, et el. ERK1/2 is involved in luteal cell autophagy regulation during corpus luteum regression via an mTOR-independent pathway. Mol Hum Reprod. 2014 Oct;20(10):972-80.

824. Amantini C, Morelli MB, Nabissi M, et el. Capsaicin triggers autophagic cell survival which drives epithelial mesenchymal transition and chemoresistance in bladder cancer

cells in an Hedgehog-dependent manner. Oncotarget. 2016 Aug 2;7(31):50180-50194.

825. Chen X, Morales-Alcala CC, Riobo-Del Galdo NA. Autophagic Flux Is Regulated by Interaction Between the C-terminal Domain of PATCHED1 and ATG101. Mol Cancer Res. 2018 May;16(5):909-919.

826. Dong S, Jia C, Zhang S, et el. The REGγ proteasome regulates hepatic lipid metabolism through inhibition of autophagy. Cell Metab. 2013 Sep 3;18(3):380-91.

827. Zhang X, Garbett K, Veeraraghavalu K, et el. A role for presenilins in autophagy revisited: normal acidification of lysosomes in cells lacking PSEN1 and PSEN2. J Neurosci. 2012 Jun 20;32(25):8633-48.

828. Rockenfeller P, Koska M, Pietrocola F, et el. Phosphatidylethanolamine positively regulates autophagy and longevity. Cell Death Differ. 2015 Mar;22(3):499-508.

829. Xu G, Li T, Chen J, et el. Autosomal dominant retinitis pigmentosa-associated gene PRPF8 is essential for hypoxia-induced mitophagy through regulating ULK1 mRNA splicing. Autophagy. 2018;14(10):1818-1830.

830. Ragusa S, Cheng J, Ivanov KI, et el. PROX1 promotes metabolic adaptation and fuels outgrowth of Wnt(high) metastatic colon cancer cells. Cell Rep. 2014 Sep 25;8(6):1957-1973.

831. Chen H, Luo Z, Dong L, et el. CD133/prominin-1-mediated autophagy and glucose uptake beneficial for hepatoma cell survival. PLoS One. 2013;8(2):e56878.

832. Yu J, Lou Y, He K, et el. Goose broodiness is involved in granulosa cell autophagy and homeostatic imbalance of follicular hormones. Poult Sci. 2016 May;95(5):1156-64.

833. Huck B, Duss S, Hausser A, et el. Elevated protein kinase D3 (PKD3) expression supports proliferation of triple-negative breast cancer cells and contributes to mTORC1-S6K1 pathway activation. J Biol Chem. 2014 Feb 7;289(6):3138-47.

834. Khaodee W, Inboot N, Udomsom S, et el. Glucosidase II beta subunit (GluIIβ) plays a role in autophagy and apoptosis regulation in lung carcinoma cells in a p53-dependent manner. Cell Oncol (Dordr). 2017 Dec;40(6):579-591.

835. Qu L, Li G, Xia D, et el. PRKCI negatively regulates autophagy via PIK3CA/AKT-MTOR signaling. Biochem Biophys Res Commun. 2016 Feb 5;470(2):306-312.

836. Shoji S, Titani K, Demaille JG, et el. Sequence of two phosphorylated sites in the catalytic subunit of bovine cardiac muscle adenosine 3':5'-monophosphate-dependent

protein kinase. J Biol Chem. 1979 Jul 25;254(14):6211-4.

837. Russo D, Ottaggio L, Foggetti G, et el. PRIMA-1 induces autophagy in cancer cells carrying mutant or wild type p53. Biochim Biophys Acta. 2013 Aug;1833(8):1904-13.

838. Duan L, Motchoulski N, Danzer B, et el. Prolylcarboxypeptidase regulates proliferation, autophagy, and resistance to 4-hydroxytamoxifen-induced cytotoxicity in estrogen receptor-positive breast cancer cells. J Biol Chem. 2011 Jan 28;286(4):2864-76.

839. Chikh A, Sanzà P, Raimondi C, et el. iASPP is a novel autophagy inhibitor in keratinocytes. J Cell Sci. 2014 Jul 15;127(Pt 14):3079-93.

840. Torii S, Yoshida T, Arakawa S, et el. Identification of PPM1D as an essential Ulk1 phosphatase for genotoxic stress-induced autophagy. EMBO Rep. 2016 Nov;17(11):1552-1564.

841. Carreira RS, Lee Y, Ghochani M, et el. Cyclophilin D is required for mitochondrial removal by autophagy in cardiac cells. Autophagy. 2010 May;6(4):462-72.

842. Mao M, Yu X, Ge X, et el. Acetylated cyclophilin A is a major mediator in hypoxia-induced autophagy and pulmonary vascular angiogenesis. J Hypertens. 2017 Apr;35(4):798-809.

843. Singh BK, Sinha RA, Tripathi M, et el. Thyroid hormone receptor and ERRα coordinately regulate mitochondrial fission, mitophagy, biogenesis, and function. Sci Signal. 2018 Jun 26;11(536).

844. Choy KW, Mustafa MR, Lau YS, et el. Paeonol protects against endoplasmic reticulum stress-induced endothelial dysfunction via AMPK/PPARδ signaling pathway. Biochem Pharmacol. 2016 Sep 15;116:51-62.

845. Sharif T, Martell E, Dai C, et el. HDAC6 differentially regulates autophagy in stem-like versus differentiated cancer cells. Autophagy. 2018 Nov 16.

846. Wang J, Cui D, Gu S, et el. Autophagy regulates apoptosis by targeting NOXA for degradation. Biochim Biophys Acta Mol Cell Res. 2018 Aug;1865(8):1105-1113.

847. Wang H, Tian C, Sun J, et el. Overexpression of PLK3 Mediates the Degradation of Abnormal Prion Proteins Dependent on Chaperone-Mediated Autophagy. Mol Neurobiol. 2017 Aug;54(6):4401-4413.

848. Chen LL, Wang YB, Song JX, et el. Phosphoproteome-based kinase activity profiling reveals the critical role of MAP2K2 and PLK1 in neuronal autophagy. Autophagy. 2017;13(11):1969-1980.

849. Tsai TH, Chen E, Li L, et el. The constitutive lipid droplet protein PLIN2 regulates autophagy in liver. Autophagy. 2017 Jul 3;13(7):1130-1144.

850. Shimabukuro MK, Langhi LG, Cordeiro I, et el. Lipid-laden cells differentially distributed in the aging brain are functionally active and correspond to distinct phenotypes. Sci Rep. 2016 Mar 31;6:23795.

851. McEwan DG, Popovic D, Gubas A, et el. PLEKHM1 regulates autophagosome-lysosome fusion through HOPS complex and LC3/GABARAP proteins. Mol Cell. 2015 Jan 8;57(1):39-54.

852. Matsuda-Lennikov M, Suizu F, Hirata N, et el. Lysosomal interaction of Akt with Phafin2: a critical step in the induction of autophagy. PLoS One. 2014 Jan 8;9(1):e79795.

853. Liu ST, Chang YL, Wang WM, et el. A non-covalent interaction between small ubiquitin-like modifier-1 and Zac1 regulates Zac1 cellular functions. Int J Biochem Cell Biol. 2012 Mar;44(3):547-55.

854. Segawa S, Kondo Y, Nakai Y, et el. Placenta Specific 8 Suppresses IL-18 Production through Regulation of Autophagy and Is Associated with Adult Still Disease. J Immunol. 2018 Dec 15;201(12):3534-3545.

855. Claerhout S, Lim JY, Choi W, et el. Gene expression signature analysis identifies vorinostat as a candidate therapy for gastric cancer. PLoS One. 2011;6(9):e24662.

856. Cebotaru V, Cebotaru L, Kim H, et el. Polycystin-1 negatively regulates Polycystin-2 expression via the aggresome/autophagosome pathway. J Biol Chem. 2014 Mar 7;289(10):6404-14.

857. Hessvik NP, Øverbye A, Brech A, et el. PIKfyve inhibition increases exosome release and induces secretory autophagy. Cell Mol Life Sci. 2016 Dec;73(24):4717-4737.

858. Feng FB, Qiu HY. Effects of Artesunate on chondrocyte proliferation, apoptosis and autophagy through the PI3K/AKT/mTOR signaling pathway in rat models with rheumatoid arthritis. Biomed Pharmacother. 2018 Jun;102:1209-1220.

859. Hu G, McQuiston T, Bernard A, et el. TOR-dependent post-transcriptional regulation of autophagy. Autophagy. 2015;11(12):2390-2.

860. Ando K, Tomimura K, Sazdovitch V, et el. Level of PICALM, a key component of clathrin-mediated endocytosis, is correlated with levels of phosphotau and autophagy-related proteins and is associated with tau inclusions in AD, PSP and Pick disease. Neurobiol Dis. 2016 Oct;94:32-43.

861. Naidu SR, Lakhter AJ, Androphy EJ. PIASy-mediated Tip60 sumoylation regulates p53-induced autophagy. Cell Cycle. 2012 Jul 15;11(14):2717-28.

862. Durbas M, Pabisz P, Wawak K, et el. GD2 ganglioside-binding antibody 14G2a and specific aurora A kinase inhibitor MK-5108 induce autophagy in IMR-32 neuroblastoma cells. Apoptosis. 2018 Oct;23(9-10):492-511.

863. Kathiria AS, Butcher LD, Feagins LA, et el. Prohibitin 1 modulates mitochondrial stress-related autophagy in human colonic epithelial cells. PLoS One. 2012;7(2):e31231.

864. Zhu X, Ji M, Han Y, et el. PGRMC1-dependent autophagy by hyperoside induces apoptosis and sensitizes ovarian cancer cells to cisplatin treatment. Int J Oncol. 2017 Mar;50(3):835-846.

865. De Amicis F, Guido C, Santoro M, et el. A novel functional interplay between Progesterone Receptor-B and PTEN, via AKT, modulates autophagy in breast cancer cells. J Cell Mol Med. 2014 Nov;18(11):2252-65.

866. Ghavami S, Mutawe MM, Schaafsma D, et el. Geranylgeranyl transferase 1 modulates autophagy and apoptosis in human airway smooth muscle. Am J Physiol Lung Cell Mol Physiol. 2012 Feb 15;302(4):L420-8.

867. Lu Y, Wang Y, Xu H, et el. Profilin 1 induces drug resistance through Beclin1 complex-mediated autophagy in multiple myeloma. Cancer Sci. 2018 Sep;109(9):2706-2716.

868. Wang C, Qu J, Yan S, et el. PFK15, a PFKFB3 antagonist, inhibits autophagy and proliferation in rhabdomyosarcoma cells. Int J Mol Med. 2018 Jul;42(1):359-367.

869. Rami A, Fekadu J, Rawashdeh O. The Hippocampal Autophagic Machinery is Depressed in the Absence of the Circadian Clock Protein PER1 that may Lead to Vulnerability During Cerebral Ischemia. Curr Neurovasc Res. 2017;14(3):207-214.

870. Neill T, Sharpe C, Owens RT, et el. Decorin-evoked paternally expressed gene 3 (PEG3) is an upstream regulator of the transcription factor EB (TFEB) in endothelial cell autophagy. J Biol Chem. 2017 Sep 29;292(39):16211-16220.

871. Noh HS, Hah YS, Zada S, et el. PEBP1, a RAF kinase inhibitory protein, negatively regulates starvation-induced autophagy by direct interaction with LC3. Autophagy. 2016 Nov;12(11):2183-2196.

872. Gupta A, Roy S, Lazar AJ, et el. Autophagy inhibition and antimalarials promote cell death in gastrointestinal stromal tumor (GIST). Proc Natl Acad Sci U S A. 2010 Aug

10;107(32):14333-8.

873. Booth L, Roberts JL, Rais R, et el. Palbociclib augments Neratinib killing of tumor cells that is further enhanced by HDAC inhibition. Cancer Biol Ther. 2018 Sep 5:1-12.

874. Fernández-Araujo A, Alfonso A, Vieytes MR, et el. Key role of phosphodiesterase 4A (PDE4A) in autophagy triggered by yessotoxin. Toxicology. 2015 Mar 2;329:60-72.

875. Zhang S, Li G, Fu X, et el. PDCD5 protects against cardiac remodeling by regulating autophagy and apoptosis. Biochem Biophys Res Commun. 2015 May 29;461(2):321-8.

876. Li Y, Wang X, Wang X, et el. PDCD4 suppresses proliferation, migration, and invasion of endometrial cells by inhibiting autophagy and NF-κB/MMP2/MMP9 signal pathway. Biol Reprod. 2018 Aug 1;99(2):360-372.

877. Ding Z, Wang X, Liu S, et el. PCSK9 expression in the ischaemic heart and its relationship to infarct size, cardiac function, and development of autophagy. Cardiovasc Res. 2018 Nov 1;114(13):1738-1751.

878. Oh S, Shin JH, Jang EJ, et el. Anti-inflammatory activity of chloroquine and amodiaquine through p21-mediated suppression of T cell proliferation and Th1 cell differentiation. Biochem Biophys Res Commun. 2016 May 27;474(2):345-350.

879. Lu H, Han M, Yuan X, et el. Role of IL-6-mediated expression of NS5ATP9 in autophagy of liver cancer cells. J Cell Physiol. 2018 Dec;233(12):9312-9319.

880. Hu X, Sui X, Li L, et el. Protocadherin 17 acts as a tumour suppressor inducing tumour cell apoptosis and autophagy, and is frequently methylated in gastric and colorectal cancers. J Pathol. 2013 Jan;229(1):62-73.

881. Rah B, ur Rasool R, Nayak D, et el. PAWR-mediated suppression of BCL2 promotes switching of 3-azido withaferin A (3-AWA)-induced autophagy to apoptosis in prostate cancer cells. Autophagy. 2015;11(2):314-31.

882. Lee DH, Kim D, Kim ST, et el. PARK7 modulates autophagic proteolysis through binding to the N-terminally arginylated form of the molecular chaperone HSPA5. Autophagy. 2018;14(11):1870-1885.

883. Fan T, Zhang C, Zong M, et el. Peptidylarginine deiminase IV promotes the development of chemoresistance through inducing autophagy in hepatocellular carcinoma. Cell Biosci. 2014 Aug 26;4:49.

884. Taylor JM, Brody KM, Lockhart PJ. Parkin co-regulated gene is involved in aggresome formation and autophagy in response to proteasomal impairment. Exp Cell Res. 2012 Oct

1;318(16):2059-70.

885. Chatterjee C, Sparks DL. Hepatic lipase release is inhibited by a purinergic induction of autophagy. Cell Physiol Biochem. 2014;33(4):883-94.

886. Mawatwal S, Behura A, Ghosh A, et el. Calcimycin mediates mycobacterial killing by inducing intracellular calcium-regulated autophagy in a P2RX7 dependent manner. Biochim Biophys Acta Gen Subj. 2017 Dec;1861(12):3190-3200.

887. Li W, Li X, Wang W, et el. Tumor suppressor gene Oxidored-nitro domain-containing protein 1 regulates nasopharyngeal cancer cell autophagy, metabolism, and apoptosis in vitro. Int J Biochem Cell Biol. 2013 Sep;45(9):2016-26.

888. Ding Z, Liu S, Wang X, et el. LOX-1, oxidant stress, mtDNA damage, autophagy, and immune response in atherosclerosis. Can J Physiol Pharmacol. 2014 Jul;92(7):524-30.

889. Chen L, Li H, Liu W, et el. Olfactomedin 4 suppresses prostate cancer cell growth and metastasis via negative interaction with cathepsin D and SDF-1. Carcinogenesis. 2011 Jul;32(7):986-94.

890. Zhou F, Yang X, Zhao H, et el. Down-regulation of OGT promotes cisplatin resistance by inducing autophagy in ovarian cancer. Theranostics. 2018 Oct 6;8(19):5200-5212.

891. Vanrell MC, Cueto JA, Barclay JJ, et el. Polyamine depletion inhibits the autophagic response modulating Trypanosoma cruzi infectivity. Autophagy. 2013 Jul;9(7):1080-93.

892. Jawhari S, Bessette B, Hombourger S, et el. Autophagy and TrkC/NT-3 signaling joined forces boost the hypoxic glioblastoma cell survival. Carcinogenesis. 2017 Jun 1;38(6):592-603.

893. Franco ML, Melero C, Sarasola E, et el. Mutations in TrkA Causing Congenital Insensitivity to Pain with Anhidrosis (CIPA) Induce Misfolding, Aggregation, and Mutation-dependent Neurodegeneration by Dysfunction of the Autophagic Flux. J Biol Chem. 2016 Oct 7;291(41):21363-21374.

894. Bouhidel JO, Wang P, Siu KL, et el. Netrin-1 improves post-injury cardiac function in vivo via DCC/NO-dependent preservation of mitochondrial integrity, while attenuating autophagy. Biochim Biophys Acta. 2015 Feb;1852(2):277-89.

895. Bouzas-Rodríguez J, Zárraga-Granados G, Sánchez-Carbente Mdel R, et el. The nuclear receptor NR4A1 induces a form of cell death dependent on autophagy in mammalian cells. PLoS One. 2012;7(10):e46422.

896. Silvente-Poirot S, Segala G, Poirot MC, et el. Ligand-dependent transcriptional induction

of lethal autophagy: A new perspective for cancer treatment. Autophagy. 2018;14(3):555-557.

897. Kruppa AJ, Ott S, Chandraratna DS, et el. Suppression of Aβ toxicity by puromycin-sensitive aminopeptidase is independent of its proteolytic activity. Biochim Biophys Acta. 2013 Dec;1832(12):2115-26.

898. Yamamura T, Ohsaki Y, Suzuki M, et el. Inhibition of Niemann-Pick-type C1-like1 by ezetimibe activates autophagy in human hepatocytes and reduces mutant α1-antitrypsin Z deposition. Hepatology. 2014 Apr;59(4):1591-9.

899. Sciarretta S, Volpe M, Sadoshima J. NOX4 regulates autophagy during energy deprivation. Autophagy. 2014 Apr;10(4):699-701.

900. Rouaud F, Boucher JL, Slama-Schwok A, et el. Mechanism of melanoma cells selective apoptosis induced by a photoactive NADPH analogue. Oncotarget. 2016 Dec 13;7(50):82804-82819.

901. Kim SM, Kim YG, Kim DJ, et el. Inflammasome-Independent Role of NLRP3 Mediates Mitochondrial Regulation in Renal Injury. Front Immunol. 2018 Nov 12;9:2563.

902. Vizza D, Perri A, Toteda G, et el. Rapamycin-induced autophagy protects proximal tubular renal cells against proteinuric damage through the transcriptional activation of the nerve growth factor receptor NGFR. Autophagy. 2018;14(6):1028-1042.

903. Wang W, Liu M, Guan Y, et el. Hypoxia-Responsive Mir-301a and Mir-301b Promote Radioresistance of Prostate Cancer Cells via Downregulating NDRG2. Med Sci Monit. 2016 Jun 21;22:2126-32.

904. Sinha RA, Singh BK, Zhou J, et el. Loss of ULK1 increases RPS6KB1-NCOR1 repression of NR1H/LXR-mediated Scd1 transcription and augments lipotoxicity in hepatic cells. Autophagy. 2017 Jan 2;13(1):169-186.

905. Wu MY, Fu J, Xu J, et el. Steroid receptor coactivator 3 regulates autophagy in breast cancer cells through macrophage migration inhibitory factor. Cell Res. 2012 Jun;22(6):1003-21.

906. Cook AJ, Gurard-Levin ZA, Vassias I, et el. A specific function for the histone chaperone NASP to fine-tune a reservoir of soluble H3-H4 in the histone supply chain. Mol Cell. 2011 Dec 23;44(6):918-27.

907. Augustin S, Berard M, Kellaf S, et el. Matrix metalloproteinases are involved in both type I (apoptosis) and type II (autophagy) cell death induced by sodium phenylacetate in

MDA-MB-231 breast tumour cells. Anticancer Res. 2009 Apr;29(4):1335-43.

908. Liu S, Wan J, Kong Y, et el. Inhibition of CRL-NEDD8 pathway as a new approach to enhance ATRA-induced differentiation of acute promyelocytic leukemia cells. Int J Med Sci. 2018 Apr 3;15(7):674-681.

909. Yasuda K, Ohyama K, Onga K, et el. Mdm20 stimulates polyQ aggregation via inhibiting autophagy through Akt-Ser473 phosphorylation. PLoS One. 2013 Dec 16;8(12):e82523.

910. Qian X, Li X, Lu Z. Protein kinase activity of the glycolytic enzyme PGK1 regulates autophagy to promote tumorigenesis. Autophagy. 2017 Jul 3;13(7):1246-1247.

911. Groth-Pedersen L, Aits S, Corcelle-Termeau E, et el. Identification of cytoskeleton-associated proteins essential for lysosomal stability and survival of human cancer cells. PLoS One. 2012;7(10):e45381.

912. Brandstaetter H, Kishi-Itakura C, Tumbarello DA, et el. Loss of functional MYO1C/myosin 1c, a motor protein involved in lipid raft trafficking, disrupts autophagosome-lysosome fusion. Autophagy. 2014;10(12):2310-23.

913. Llewellyn KJ, Nalbandian A, Weiss LN, et el. Myogenic differentiation of VCP disease-induced pluripotent stem cells: A novel platform for drug discovery. PLoS One. 2017 Jun 2;12(6):e0176919.

914. Kumar V, Aneesh KA, Kshemada K, et el. Amalaki rasayana, a traditional Indian drug enhances cardiac mitochondrial and contractile functions and improves cardiac function in rats with hypertrophy. Sci Rep. 2017 Aug 17;7(1):8588.

915. Wang J, Huang S, Tian R, et el. The protective autophagy activated by GANT-61 in MYCN amplified neuroblastoma cells is mediated by PERK. Oncotarget. 2018 Jan 13;9(18):14413-14427.

916. Singh SR, Zech ATL, Geertz B, et el. Activation of Autophagy Ameliorates Cardiomyopathy in Mybpc3-Targeted Knockin Mice. Circ Heart Fail. 2017 Oct;10(10).

917. Garbar C, Mascaux C, Giustiniani J, et el. Autophagy is decreased in triple-negative breast carcinoma involving likely the MUC1-EGFR-NEU1 signalling pathway. Int J Clin Exp Pathol. 2015 May 1;8(5):4344-55.

918. Zou J, Zhang C, Marjanovic J, et el. Myotubularin-related protein (MTMR) 9 determines the enzymatic activity, substrate specificity, and role in autophagy of MTMR8. Proc Natl Acad Sci U S A. 2012 Jun 12;109(24):9539-44.

919. Wang DT, Yang YJ, Huang RH, et el. Myostatin Activates the Ubiquitin-Proteasome and

Autophagy-Lysosome Systems Contributing to Muscle Wasting in Chronic Kidney Disease. Oxid Med Cell Longev. 2015;2015:684965.

920. Zhu Q, Wang H, Wang H, et el. Protective effects of ethyl pyruvate on lipopolysaccharide-induced acute lung injury through inhibition of autophagy in neutrophils. Mol Med Rep. 2017 Mar;15(3):1272-1278.

921. Abnave P, Mottola G, Gimenez G, et el. Screening in planarians identifies MORN2 as a key component in LC3-associated phagocytosis and resistance to bacterial infection. Cell Host Microbe. 2014 Sep 10;16(3):338-50.

922. Li WD, Hu N, Lei FR, et el. Autophagy inhibits endothelial progenitor cells migration via the regulation of MMP2, MMP9 and uPA under normoxia condition. Biochem Biophys Res Commun. 2015 Oct 23;466(3):376-80.

923. Zhan M, Usman IM, Sun L, et el. Disruption of renal tubular mitochondrial quality control by Myo-inositol oxygenase in diabetic kidney disease. J Am Soc Nephrol. 2015 Jun;26(6):1304-21.

924. Chen HR, Chuang YC, Chao CH, et el. Macrophage migration inhibitory factor induces vascular leakage via autophagy. Biol Open. 2015 Jan 23;4(2):244-52.

925. Zhang Y, Zhang Y, Tang J, et el. Oxymatrine Inhibits Homocysteine-Mediated Autophagy via MIF/mTOR Signaling in Human Umbilical Vein Endothelial Cells. Cell Physiol Biochem. 2018;45(5):1893-1903.

926. Majumder P, Chakrabarti O. Mahogunin regulates fusion between amphisomes/MVBs and lysosomes via ubiquitination of TSG101. Cell Death Dis. 2015 Nov 5;6:e1970.

927. Zhang W, Hou J, Wang X, et el. PTPRO-mediated autophagy prevents hepatosteatosis and tumorigenesis. Oncotarget. 2015 Apr 20;6(11):9420-33.

928. Lorente M, Torres S, Salazar M, et el. Stimulation of the midkine/ALK axis renders glioma cells resistant to cannabinoid antitumoral action. Cell Death Differ. 2011 Jun;18(6):959-73.

929. Tomar D, Dong Z, Shanmughapriya S, et el. MCUR1 Is a Scaffold Factor for the MCU Complex Function and Promotes Mitochondrial Bioenergetics. Cell Rep. 2016 May 24;15(8):1673-85.

930. Kim Y, Kim C, Son SM, et el. The novel RAGE interactor PRAK is associated with autophagy signaling in Alzheimer's disease pathogenesis. Mol Neurodegener. 2016 Jan 12;11:4.

931. Saleiro D, Blyth GT, Kosciuczuk EM, et el. IFN-γ-inducible antiviral responses require ULK1-mediated activation of MLK3 and ERK5. Sci Signal. 2018 Nov 20;11(557).

932. Colecchia D, Dapporto F, Tronnolone S, et el. MAPK15 is part of the ULK complex and controls its activity to regulate early phases of the autophagic process. J Biol Chem. 2018 Oct 12;293(41):15962-15976.

933. Zhong W, Zhu H, Sheng F, et el. Activation of the MAPK11/12/13/14 (p38 MAPK) pathway regulates the transcription of autophagy genes in response to oxidative stress induced by a novel copper complex in HeLa cells. Autophagy. 2014 Jul;10(7):1285-300.

934. Mahoney E, Byrd JC, Johnson AJ. Autophagy and ER stress play an essential role in the mechanism of action and drug resistance of the cyclin-dependent kinase inhibitor flavopiridol. Autophagy. 2013 Mar;9(3):434-5.

935. Linares JF, Duran A, Reina-Campos M, et el. Amino Acid Activation of mTORC1 by a PB1-Domain-Driven Kinase Complex Cascade. Cell Rep. 2015 Aug 25;12(8):1339-52.

936. Ahn JS, Ann EJ, Kim MY, et el. Autophagy negatively regulates tumor cell proliferation through phosphorylation dependent degradation of the Notch1 intracellular domain. Oncotarget. 2016 Nov 29;7(48):79047-79063.

937. Zhou Y, Cao ZQ, Wang HY, et el. The anti-inflammatory effects of Morin hydrate in atherosclerosis is associated with autophagy induction through cAMP signaling. Mol Nutr Food Res. 2017 Sep;61(9).

938. Harrison B, Kraus M, Burch L, et el. DAPK-1 binding to a linear peptide motif in MAP1B stimulates autophagy and membrane blebbing. J Biol Chem. 2008 Apr 11;283(15):9999-10014.

939. Ugun-Klusek A, Theodosi TS, Fitzgerald JC, et el. Monoamine oxidase-A promotes protective autophagy in human SH-SY5Y neuroblastoma cells through Bcl-2 phosphorylation. Redox Biol. 2018 Oct 9;20:167-181.

940. Sohda M, Misumi Y, Ogata S, et el. Trans-Golgi protein p230/golgin-245 is involved in phagophore formation. Biochem Biophys Res Commun. 2015 Jan 2;456(1):275-81.

941. Wu J, Zhang D, Li J, et el. MACC1 induces autophagy to regulate proliferation, apoptosis, migration and invasion of squamous cell carcinoma. Oncol Rep. 2017 Oct;38(4):2369-2377.

942. Li X, He S, Zhou X, et el. Lyn Delivers Bacteria to Lysosomes for Eradication through TLR2-Initiated Autophagy Related Phagocytosis. PLoS Pathog. 2016 Jan

6;12(1):e1005363.

943. Yahiro K, Satoh M, Nakano M, et el. Low-density lipoprotein receptor-related protein-1 (LRP1) mediates autophagy and apoptosis caused by Helicobacter pylori VacA. J Biol Chem. 2012 Sep 7;287(37):31104-15.

944. Alkhairy OK, Abolhassani H, Rezaei N, et el. Spectrum of Phenotypes Associated with Mutations in LRBA. J Clin Immunol. 2016 Jan;36(1):33-45.

945. Xie CM, Liu XY, Sham KW, et el. Silencing of EEF2K (eukaryotic elongation factor-2 kinase) reveals AMPK-ULK1-dependent autophagy in colon cancer cells. Autophagy. 2014 Sep;10(9):1495-508.

946. Bertolo C, Roa S, Sagardoy A, et el. LITAF, a BCL6 target gene, regulates autophagy in mature B-cell lymphomas. Br J Haematol. 2013 Sep;162(5):621-30.

947. Akbari M, Keijzers G, Maynard S, et el. Overexpression of DNA ligase III in mitochondria protects cells against oxidative stress and improves mitochondrial DNA base excision repair. DNA Repair (Amst). 2014 Apr;16:44-53.

948. Montespan C, Marvin SA, Austin S, et el. Multi-layered control of Galectin-8 mediated autophagy during adenovirus cell entry through a conserved PPxY motif in the viral capsid. PLoS Pathog. 2017 Feb 13;13(2):e1006217.

949. Chan YK, Sung HK, Jahng JW, et el. Lipocalin-2 inhibits autophagy and induces insulin resistance in H9c2 cells. Mol Cell Endocrinol. 2016 Jul 15;430:68-76.

950. Wang L, Liu X, Nie J, et el. ALCAT1 controls mitochondrial etiology of fatty liver diseases, linking defective mitophagy to steatosis. Hepatology. 2015 Feb;61(2):486-96.

951. Segev N, Hay N. Hijacking leucyl-tRNA synthetase for amino acid-dependent regulation of TORC1. Mol Cell. 2012 Apr 13;46(1):4-6.

952. Liu B, Wang T, Wang H, et el. Oncoprotein HBXIP enhances HOXB13 acetylation and co-activates HOXB13 to confer tamoxifen resistance in breast cancer. J Hematol Oncol. 2018 Feb 23;11(1):26.

953. Lin CY, Nozawa T, Minowa-Nozawa A, et el. LAMTOR2/LAMTOR1 complex is required for TAX1BP1-mediated xenophagy. Cell Microbiol. 2018 Nov 14:e12981.

954. Baek A, Yoon S, Kim J, et el. Autophagy and KRT8/keratin 8 protect degeneration of retinal pigment epithelium under oxidative stress. Autophagy. 2017 Feb;13(2):248-263.

955. Laurila E, Vuorinen E, Savinainen K, et el. KPNA7, a nuclear transport receptor,

promotes malignant properties of pancreatic cancer cells in vitro. Exp Cell Res. 2014 Mar 10;322(1):159-67.

956. Xie B, Zhou J, Shu G, et el. Restoration of klotho gene expression induces apoptosis and autophagy in gastric cancer cells: tumor suppressive role of klotho in gastric cancer. Cancer Cell Int. 2013 Feb 21;13(1):18.

957. Deng L, Feng J, Broaddus RR. The novel estrogen-induced gene EIG121 regulates autophagy and promotes cell survival under stress. Cell Death Dis. 2010;1:e32.

958. Epple UD, Eskelinen EL, Thumm M. Intravacuolar membrane lysis in Saccharomyces cerevisiae. Does vacuolar targeting of Cvt17/Aut5p affect its function. J Biol Chem. 2003 Mar 7;278(10):7810-21.

959. Yi C, Yu L. How does acetylation regulate autophagy. Autophagy. 2012 Oct;8(10):1529-30.

960. Yogev O, Goldberg R, Anzi S, et el. Jun proteins are starvation-regulated inhibitors of autophagy. Cancer Res. 2010 Mar 15;70(6):2318-27.

961. Pratt J, Annabi B. Induction of autophagy biomarker BNIP3 requires a JAK2/STAT3 and MT1-MMP signaling interplay in Concanavalin-A-activated U87 glioblastoma cells. Cell Signal. 2014 May;26(5):917-24.

962. Yeo SK, Guan JL. Hierarchical heterogeneity in mammary tumors and its regulation by autophagy. Autophagy. 2016 Oct 2;12(10):1960-1961.

963. Chhangani D, Upadhyay A, Amanullah A, et el. Ubiquitin ligase ITCH recruitment suppresses the aggregation and cellular toxicity of cytoplasmic misfolded proteins. Sci Rep. 2014 May 28;4:5077.

964. Desai SD, Reed RE, Babu S, et el. ISG15 deregulates autophagy in genotoxin-treated ataxia telangiectasia cells. J Biol Chem. 2013 Jan 25;288(4):2388-402.

965. Ratovitski EA. ΔNp63α/IRF6 interplay activates NOS2 transcription and induces autophagy upon tobacco exposure. Arch Biochem Biophys. 2011 Feb 15;506(2):208-15.

966. Morelli E, Leone E, Cantafio ME, et el. Selective targeting of IRF4 by synthetic microRNA-125b-5p mimics induces anti-multiple myeloma activity in vitro and in vivo. Leukemia. 2015 Nov;29(11):2173-83.

967. Liang J, Piao Y, Henry V, et el. Interferon-regulatory factor-1 (IRF1) regulates bevacizumab induced autophagy. Oncotarget. 2015 Oct 13;6(31):31479-92.

968. Wu Q, van Dyk LF, Jiang D, et el. Interleukin-1 receptor-associated kinase M (IRAK-M) promotes human rhinovirus infection in lung epithelial cells via the autophagic pathway. Virology. 2013 Nov;446(1-2):199-206.

969. Zheng HC, Zhao S, Song Y, et el. The roles of ING5 expression in ovarian carcinogenesis and subsequent progression: a target of gene therapy. Oncotarget. 2017 Oct 19;8(61):103449-103464.

970. Gong A, Ye S, Xiong E, et el. Autophagy contributes to ING4-induced glioma cell death. Exp Cell Res. 2013 Jul 15;319(12):1714-23.

971. Jiao H, Su GQ, Dong W, et el. Chaperone-like protein p32 regulates ULK1 stability and autophagy. Cell Death Differ. 2015 Nov;22(11):1812-23.

972. Wang X, Shen C, Liu Z, et el. Nitazoxanide, an antiprotozoal drug, inhibits late-stage autophagy and promotes ING1-induced cell cycle arrest in glioblastoma. Cell Death Dis. 2018 Oct 9;9(10):1032.

973. Sosa P, Alcalde-Estevez E, Plaza P, et el. Hyperphosphatemia Promotes Senescence of Myoblasts by Impairing Autophagy Through Ilk Overexpression, A Possible Mechanism Involved in Sarcopenia. Aging Dis. 2018 Oct 1;9(5):769-784.

974. Xia F, Deng C, Jiang Y, et el. IL4 (interleukin 4) induces autophagy in B cells leading to exacerbated asthma. Autophagy. 2018;14(3):450-464.

975. Sharma G, Dutta RK, Khan MA, et el. IL-27 inhibits IFN-γ induced autophagy by concomitant induction of JAK/PI3 K/Akt/mTOR cascade and up-regulation of Mcl-1 in Mycobacterium tuberculosis H37Rv infected macrophages. Int J Biochem Cell Biol. 2014 Oct;55:335-47.

976. Zheng T, Xu C, Mao C, et el. Increased Interleukin-23 in Hashimoto's Thyroiditis Disease Induces Autophagy Suppression and Reactive Oxygen Species Accumulation. Front Immunol. 2018 Jan 29;9:96.

977. Lodder J, Denaës T, Chobert MN, et el. Macrophage autophagy protects against liver fibrosis in mice. Autophagy. 2015;11(8):1280-92.

978. Shi J, Wang H, Guan H, et el. IL10 inhibits starvation-induced autophagy in hypertrophic scar fibroblasts via cross talk between the IL10-IL10R-STAT3 and IL10-AKT-mTOR pathways. Cell Death Dis. 2016 Mar 10;7:e2133.

979. Zarzyńska J, Gajkowska B, Wojewódzka U, et el. Apoptosis and autophagy in involuting bovine mammary gland is accompanied by up-regulation of TGF-beta1 and suppression

of somatotropic pathway. Pol J Vet Sci. 2007;10(1):1-9.

980. Chen G, Zhou X, Xu Z. Effects of IGFBP3 gene silencing mediated inhibition of ERK/MAPK signaling pathway on proliferation, apoptosis, autophagy, and cell senescence in rats nucleus pulposus cells. J Cell Physiol. 2018 Oct 28.

981. Ramakrishnan R, Gabrilovich DI. The role of mannose-6-phosphate receptor and autophagy in influencing the outcome of combination therapy. Autophagy. 2013 Apr;9(4):615-6.

982. Yu W, Cui X, Wan Z, et el. Silencing forkhead box M1 promotes apoptosis and autophagy through SIRT7/mTOR/IGF2 pathway in gastric cancer cells. J Cell Biochem. 2018 Nov;119(11):9090-9098.

983. Chrysanthopoulou A, Kambas K, Stakos D, et el. Interferon lambda1/IL-29 and inorganic polyphosphate are novel regulators of neutrophil-driven thromboinflammation. J Pathol. 2017 Sep;243(1):111-122.

984. Ambjørn M, Ejlerskov P, Liu Y, et el. IFNB1/interferon-β-induced autophagy in MCF-7 breast cancer cells counteracts its proapoptotic function. Autophagy. 2013 Mar;9(3):287-302.

985. Kurt R, Chandra PK, Aboulnasr F, et el. Chaperone-Mediated Autophagy Targets IFNAR1 for Lysosomal Degradation in Free Fatty Acid Treated HCV Cell Culture. PLoS One. 2015 May 11;10(5):e0125962.

986. Dong G, You M, Fan H, et el. STS-1 promotes IFN-α induced autophagy by activating the JAK1-STAT1 signaling pathway in B cells. Eur J Immunol. 2015 Aug;45(8):2377-88.

987. Yount JS, Karssemeijer RA, Hang HC. S-palmitoylation and ubiquitination differentially regulate interferon-induced transmembrane protein 3 (IFITM3)-mediated resistance to influenza virus. J Biol Chem. 2012 Jun 1;287(23):19631-41.

988. Huang J, Li Y, Qi Y, et el. Coordinated regulation of autophagy and apoptosis determines endothelial cell fate during Dengue virus type 2 infection. Mol Cell Biochem. 2014 Dec;397(1-2):157-65.

989. Shi X, Liu J, Liu Q, et el. IFI16 mis-localization can be a contributing factor to hepatocellular carcinoma progression. Med Hypotheses. 2014 Mar;82(3):398-400.

990. Gilbert MR, Liu Y, Neltner J, et el. Autophagy and oxidative stress in gliomas with IDH1 mutations. Acta Neuropathol. 2014 Feb;127(2):221-33.

991. Rivera JF, Costes S, Gurlo T, et el. Autophagy defends pancreatic β cells from human islet

amyloid polypeptide-induced toxicity. J Clin Invest. 2014 Aug;124(8):3489-500.

992. Darreh-Shori T, Rezaeianyazdi S, Lana E, et el. Increased Active OMI/HTRA2 Serine Protease Displays a Positive Correlation with Cholinergic Alterations in the Alzheimer's Disease Brain. Mol Neurobiol. 2018 Oct 25.

993. Lian WS, Ko JY, Chen YS, et el. Chaperonin 60 sustains osteoblast autophagy and counteracts glucocorticoid aggravation of osteoporosis by chaperoning RPTOR. Cell Death Dis. 2018 Sep 17;9(10):938.

994. Matsumoto T, Urushido M, Ide H, et el. Small Heat Shock Protein Beta-1 (HSPB1) Is Upregulated and Regulates Autophagy and Apoptosis of Renal Tubular Cells in Acute Kidney Injury. PLoS One. 2015 May 11;10(5):e0126229.

995. Iannaccone A, Giorgianni F, New DD, et el. Circulating Autoantibodies in Age-Related Macular Degeneration Recognize Human Macular Tissue Antigens Implicated in Autophagy, Immunomodulation, and Protection from Oxidative Stress and Apoptosis. PLoS One. 2015 Dec 30;10(12):e0145323.

996. Young CN, Sinadinos A, Lefebvre A, et el. A novel mechanism of autophagic cell death in dystrophic muscle regulated by P2RX7 receptor large-pore formation and HSP90. Autophagy. 2015;11(1):113-30.

997. Webber DL, Choo A, Hewson LJ, et el. Neuronal-specific impairment of heparan sulfate degradation in Drosophila reveals pathogenic mechanisms for Mucopolysaccharidosis type IIIA. Exp Neurol. 2018 May;303:38-47.

998. Kang MH, Das J, Gurunathan S, et el. The cytotoxic effects of dimethyl sulfoxide in mouse preimplantation embryos: a mechanistic study. Theranostics. 2017 Oct 17;7(19):4735-4752.

999. Pike LR, Phadwal K, Simon AK, et el. ATF4 orchestrates a program of BH3-only protein expression in severe hypoxia. Mol Biol Rep. 2012 Dec;39(12):10811-22.

1000. Banreti A, Hudry B, Sass M, et el. Hox proteins mediate developmental and environmental control of autophagy. Dev Cell. 2014 Jan 13;28(1):56-69.

1001. Meng Y, Gao R, Ma J, et el. MicroRNA-140-5p regulates osteosarcoma chemoresistance by targeting HMGN5 and autophagy. Sci Rep. 2017 Mar 24;7(1):416.

1002. Jiao L, Zhang HL, Li DD, et el. Regulation of glycolytic metabolism by autophagy in liver cancer involves selective autophagic degradation of HK2 (hexokinase 2). Autophagy. 2018;14(4):671-684.

1003. Quiroga C, Gatica D, Paredes F, et el. Herp depletion protects from protein aggregation by up-regulating autophagy. Biochim Biophys Acta. 2013 Dec;1833(12):3295-3305.

1004. Shi W, Wei X, Wang Z, et el. HDAC9 exacerbates endothelial injury in cerebral ischaemia/reperfusion injury. J Cell Mol Med. 2016 Jun;20(6):1139-49.

1005. Roccaro AM, Sacco A, Jia X, et el. microRNA-dependent modulation of histone acetylation in Waldenstrom macroglobulinemia. Blood. 2010 Sep 2;116(9):1506-14.

1006. Yang D, Xiao C, Long F, et el. HDAC4 regulates vascular inflammation via activation of autophagy. Cardiovasc Res. 2018 Jun 1;114(7):1016-1028.

1007. Sun XY, Qu Y, Ni AR, et el. Novel histone deacetylase inhibitor N25 exerts anti-tumor effects and induces autophagy in human glioma cells by inhibiting HDAC3. Oncotarget. 2017 Sep 8;8(43):75232-75242.

1008. Chae YB, Kim MM. Activation of p53 by spermine mediates induction of autophagy in HT1080 cells. Int J Biol Macromol. 2014 Feb;63:56-63.

1009. Wong YC, Holzbaur EL. The regulation of autophagosome dynamics by huntingtin and HAP1 is disrupted by expression of mutant huntingtin, leading to defective cargo degradation. J Neurosci. 2014 Jan 22;34(4):1293-305.

1010. Liu Z, Chen P, Gao H, et el. Ubiquitylation of autophagy receptor Optineurin by HACE1 activates selective autophagy for tumor suppression. Cancer Cell. 2014 Jul 14;26(1):106-20.

1011. Huenchuguala S, Muñoz P, Zavala P, et el. Glutathione transferase mu 2 protects glioblastoma cells against aminochrome toxicity by preventing autophagy and lysosome dysfunction. Autophagy. 2014 Apr;10(4):618-30.

1012. Zhou J, Freeman TA, Ahmad F, et el. GSK-3α is a central regulator of age-related pathologies in mice. J Clin Invest. 2013 Apr;123(4):1821-32.

1013. Holler CJ, Taylor G, McEachin ZT, et el. Trehalose upregulates progranulin expression in human and mouse models of GRN haploinsufficiency: a novel therapeutic lead to treat frontotemporal dementia. Mol Neurodegener. 2016 Jun 24;11(1):46.

1014. Weng TP, Fu TC, Wang CH, et el. Activation of lymphocyte autophagy/apoptosis reflects haemodynamic inefficiency and functional aerobic impairment in patients with heart failure. Clin Sci (Lond). 2014 Nov;127(10):589-602.

1015. Garcia-Marcos M, Ear J, Farquhar MG, et el. A GDI (AGS3) and a GEF (GIV) regulate autophagy by balancing G protein activity and growth factor signals. Mol Biol Cell. 2011

Mar 1;22(5):673-86.

1016. Søreng K, Knævelsrud H, Holland P, et el. HS1BP3 inhibits autophagy by regulation of PLD1. Autophagy. 2017 May 4;13(5):985-986.

1017. Zhou YY, Jiang JC, You J, et el. Effect of Golgi phosphoprotein 2 (GOLPH2/GP73) on autophagy in human hepatocellular carcinoma HepG2 cells. Tumour Biol. 2015 May;36(5):3399-406.

1018. Ambrosini G, Musi E, Ho AL, et el. Inhibition of mutant GNAQ signaling in uveal melanoma induces AMPK-dependent autophagic cell death. Mol Cancer Ther. 2013 May;12(5):768-76.

1019. Fakharnia F, Khodagholi F, Dargahi L, et el. Prevention of Cyclophilin D-Mediated mPTP Opening Using Cyclosporine-A Alleviates the Elevation of Necroptosis, Autophagy and Apoptosis-Related Markers Following Global Cerebral Ischemia-Reperfusion. J Mol Neurosci. 2017 Jan;61(1):52-60.

1020. Cai X, Li J, Wang M, et el. GLP-1 Treatment Improves Diabetic Retinopathy by Alleviating Autophagy through GLP-1R-ERK1/2-HDAC6 Signaling Pathway. Int J Med Sci. 2017 Sep 19;14(12):1203-1212.

1021. Hsiao CJ, Chang CH, Ibrahim RB, et el. Gli2 modulates cell cycle re-entry through autophagy-mediated regulation of the length of primary cilia. J Cell Sci. 2018 Dec 17;131(24).

1022. Sun Y, Guo W, Ren T, et el. Gli1 inhibition suppressed cell growth and cell cycle progression and induced apoptosis as well as autophagy depending on ERK1/2 activity in human chondrosarcoma cells. Cell Death Dis. 2014 Jan 2;5:e979.

1023. Martins-Marques T, Catarino S, Zuzarte M, et el. Ischaemia-induced autophagy leads to degradation of gap junction protein connexin43 in cardiomyocytes. Biochem J. 2015 Apr 15;467(2):231-45.

1024. Bhattacharya S, Pal K, Sharma AK, et el. GAIP interacting protein C-terminus regulates autophagy and exosome biogenesis of pancreatic cancer through metabolic pathways. PLoS One. 2014 Dec 3;9(12):e114409.

1025. Jiang P, Mukthavaram R, Chao Y, et el. In vitro and in vivo anticancer effects of mevalonate pathway modulation on human cancer cells. Br J Cancer. 2014 Oct 14;111(8):1562-71.

1026. Kim MJ, Choi OK, Chae KS, et el. Autophagy deficiency in β cells blunts

incretin-induced suppression of glucagon release from α cells. Islets. 2015;7(5):e1129096.

1027. Piras A, Gianetto D, Conte D, et el. Activation of autophagy in a rat model of retinal ischemia following high intraocular pressure. PLoS One. 2011;6(7):e22514.

1028. Du TT, Wang L, Duan CL, et el. GBA deficiency promotes SNCA/α-synuclein accumulation through autophagic inhibition by inactivated PPP2A. Autophagy. 2015;11(10):1803-20.

1029. Nabatov AA, Hatzis P, Rouschop KM, et el. Hypoxia inducible NOD2 interacts with 3-O-sulfogalactoceramide and regulates vesicular homeostasis. Biochim Biophys Acta. 2013 Nov;1830(11):5277-86.

1030. He G, Xu W, Tong L, et el. Gadd45b prevents autophagy and apoptosis against rat cerebral neuron oxygen-glucose deprivation/reperfusion injury. Apoptosis. 2016 Apr;21(4):390-403.

1031. Zhang D, Zhang W, Li D, et el. GADD45A inhibits autophagy by regulating the interaction between BECN1 and PIK3C3. Autophagy. 2015;11(12):2247-58.

1032. Zhu W, Swaminathan G, Plowey ED. GA binding protein augments autophagy via transcriptional activation of BECN1-PIK3C3 complex genes. Autophagy. 2014 Sep;10(9):1622-36.

1033. Kang YK, Putluri N, Maity S, et el. CAPER is vital for energy and redox homeostasis by integrating glucose-induced mitochondrial functions via ERR-α-Gabpa and stress-induced adaptive responses via NF-κB-cMYC. PLoS Genet. 2015 Apr 1;11(4):e1005116.

1034. Yamada E, Okada S, Bastie CC, et el. Fyn phosphorylates AMPK to inhibit AMPK activity and AMP-dependent activation of autophagy. Oncotarget. 2016 Nov 15;7(46):74612-74629.

1035. Pankiv S, Johansen T. FYCO1: linking autophagosomes to microtubule plus end-directing molecular motors. Autophagy. 2010 May;6(4):550-2.

1036. Nabhan JF, Gooch RL, Piatnitski Chekler EL, et el. Perturbation of cellular proteostasis networks identifies pathways that modulate precursor and intermediate but not mature levels of frataxin. Sci Rep. 2015 Dec 16;5:18251.

1037. Matsumura F, Yamakita Y, Starovoytov V, et el. Fascin confers resistance to Listeria infection in dendritic cells. J Immunol. 2013 Dec 15;191(12):6156-64.

1038. Lin X, Zhang Y, Liu L, et el. FRS2α is essential for the fibroblast growth factor to regulate the mTOR pathway and autophagy in mouse embryonic fibroblasts. Int J Biol Sci.

2011;7(8):1114-21.

1039. Lin X, Liu X, Ma Y, et el. Coherent apoptotic and autophagic activities involved in regression of chicken postovulatory follicles. Aging (Albany NY). 2018 Apr 29;10(4):819-832.

1040. Bareford MD, Hamed HA, Tang Y, et el. Sorafenib enhances pemetrexed cytotoxicity through an autophagy-dependent mechanism in cancer cells. Autophagy. 2011 Oct;7(10):1261-2.

1041. Huett A, Ng A, Cao Z, et el. A novel hybrid yeast-human network analysis reveals an essential role for FNBP1L in antibacterial autophagy. J Immunol. 2009 Apr 15;182(8):4917-30.

1042. Dany M, Gencer S, Nganga R, et el. Targeting FLT3-ITD signaling mediates ceramide-dependent mitophagy and attenuates drug resistance in AML. Blood. 2016 Oct 13;128(15):1944-1958.

1043. Svobodova M, Raudenska M, Gumulec J, et el. Establishment of oral squamous cell carcinoma cell line and magnetic bead-based isolation and characterization of its CD90/CD44 subpopulations. Oncotarget. 2017 Aug 3;8(39):66254-66269.

1044. Gassen NC, Hartmann J, Schmidt MV, et el. FKBP5/FKBP51 enhances autophagy to synergize with antidepressant action. Autophagy. 2015;11(3):578-80.

1045. Belleudi F, Purpura V, Caputo S, et el. FGF7/KGF regulates autophagy in keratinocytes: A novel dual role in the induction of both assembly and turnover of autophagosomes. Autophagy. 2014 May;10(5):803-21.

1046. Eom YW, Oh JE, Lee JI, et el. The role of growth factors in maintenance of stemness in bone marrow-derived mesenchymal stem cells. Biochem Biophys Res Commun. 2014 Feb 28;445(1):16-22.

1047. Tang C, Shan Y, Hu Y, et el. FGF2 Attenuates Neural Cell Death via Suppressing Autophagy after Rat Mild Traumatic Brain Injury. Stem Cells Int. 2017;2017:2923182.

1048. Yu JJ, Sun HT, Zhang ZF, et el. IL15 promotes growth and invasion of endometrial stromal cells and inhibits killing activity of NK cells in endometriosis. Reproduction. 2016 Aug;152(2):151-60.

1049. Murdoch JD, Rostosky CM, Gowrisankaran S, et el. Endophilin-A Deficiency Induces the Foxo3a-Fbxo32 Network in the Brain and Causes Dysregulation of Autophagy and the Ubiquitin-Proteasome System. Cell Rep. 2016 Oct 18;17(4):1071-1086.

1050. Chen KD, Wang CC, Tsai MC, et el. Interconnections between autophagy and the coagulation cascade in hepatocellular carcinoma. Cell Death Dis. 2014 May 22;5:e1244.

1051. Du C, Zhang T, Xiao X, et el. Protease-activated receptor-2 promotes kidney tubular epithelial inflammation by inhibiting autophagy via the PI3K/Akt/mTOR signalling pathway. Biochem J. 2017 Aug 2;474(16):2733-2747.

1052. Lamattina AM, Taveira-Dasilva A, Goldberg HJ, et el. Circulating Biomarkers From the Phase 1 Trial of Sirolimus and Autophagy Inhibition for Patients With Lymphangioleiomyomatosis. Chest. 2018 Nov;154(5):1070-1082.

1053. Wei FZ, Cao Z, Wang X, et el. Epigenetic regulation of autophagy by the methyltransferase EZH2 through an MTOR-dependent pathway. Autophagy. 2015;11(12):2309-22.

1054. Zheng K, Li Y, Wang S, et el. Inhibition of autophagosome-lysosome fusion by ginsenoside Ro via the ESR2-NCF1-ROS pathway sensitizes esophageal cancer cells to 5-fluorouracil-induced cell death via the CHEK1-mediated DNA damage checkpoint. Autophagy. 2016 Sep;12(9):1593-613.

1055. Felzen V, Hiebel C, Koziollek-Drechsler I, et el. Estrogen receptor α regulates non-canonical autophagy that provides stress resistance to neuroblastoma and breast cancer cells and involves BAG3 function. Cell Death Dis. 2015 Jul 9;6:e1812.

1056. Scheibye-Knudsen M, Ramamoorthy M, Sykora P, et el. Cockayne syndrome group B protein prevents the accumulation of damaged mitochondria by promoting mitochondrial autophagy. J Exp Med. 2012 Apr 9;209(4):855-69.

1057. Booth L, Albers T, Roberts JL, et el. Multi-kinase inhibitors interact with sildenafil and ERBB1/2/4 inhibitors to kill tumor cells in vitro and in vivo. Oncotarget. 2016 Jun 28;7(26):40398-40417.

1058. Xie X, Le L, Fan Y, et el. Autophagy is induced through the ROS-TP53-DRAM1 pathway in response to mitochondrial protein synthesis inhibition. Autophagy. 2012 Jul 1;8(7):1071-84.

1059. Welsch T, Younsi A, Disanza A, et el. Eps8 is recruited to lysosomes and subjected to chaperone-mediated autophagy in cancer cells. Exp Cell Res. 2010 Jul 15;316(12):1914-24.

1060. Zhao Y, Li H, Wu R, et el. Antitumor Effects of Oncolytic Adenovirus-Carrying siRNA Targeting Potential Oncogene EphA3. PLoS One. 2015 May 15;10(5):e0126726.

1061. Tanabe H, Kuribayashi K, Tsuji N, et el. Sesamin induces autophagy in colon cancer cells by reducing tyrosine phosphorylation of EphA1 and EphB2. Int J Oncol. 2011 Jul;39(1):33-40.

1062. Cruzeiro GAV, Dos Reis MB, Silveira VS, et el. HIF1A is Overexpressed in Medulloblastoma and its Inhibition Reduces Proliferation and Increases EPAS1 and ATG16L1 Methylation. Curr Cancer Drug Targets. 2018;18(3):287-294.

1063. Zadran S, Amighi A, Otiniano E, et el. ENTPD5-mediated modulation of ATP results in altered metabolism and decreased survival in gliomablastoma multiforme. Tumour Biol. 2012 Dec;33(6):2411-21.

1064. Biederbick A, Rose S, Elsässer HP. A human intracellular apyrase-like protein, LALP70, localizes to lysosomal/autophagic vacuoles. J Cell Sci. 1999 Aug;112 (Pt 15):2473-84.

1065. Ren SX, Shen J, Cheng AS, et el. FK-16 derived from the anticancer peptide LL-37 induces caspase-independent apoptosis and autophagic cell death in colon cancer cells. PLoS One. 2013 May 20;8(5):e63641.

1066. Deroyer C, Rénert AF, Merville MP, et el. New role for EMD (emerin), a key inner nuclear membrane protein, as an enhancer of autophagosome formation in the C16-ceramide autophagy pathway. Autophagy. 2014 Jul;10(7):1229-40.

1067. Shen X, Kan S, Hu J, et el. EMC6/TMEM93 suppresses glioblastoma proliferation by modulating autophagy. Cell Death Dis. 2016 Jan 14;7:e2043.

1068. Wen X, Klionsky DJ. BRD4 is a newly characterized transcriptional regulator that represses autophagy and lysosomal function. Autophagy. 2017;13(11):1801-1803.

1069. He J, Yu JJ, Xu Q, et el. Downregulation of ATG14 by EGR1-MIR152 sensitizes ovarian cancer cells to cisplatin-induced apoptosis by inhibiting cyto-protective autophagy. Autophagy. 2015;11(2):373-84.

1070. Rantanen K, Pursiheimo JP, Högel H, et el. p62/SQSTM1 regulates cellular oxygen sensing by attenuating PHD3 activity through aggregate sequestration and enhanced degradation. J Cell Sci. 2013 Mar 1;126(Pt 5):1144-54.

1071. Maynard AA, Dvorak K, Khailova L, et el. Epidermal growth factor reduces autophagy in intestinal epithelium and in the rat model of necrotizing enterocolitis. Am J Physiol Gastrointest Liver Physiol. 2010 Sep;299(3):G614-22.

1072. Saito K, Iizuka Y, Ohta S, et el. Functional analysis of a novel glioma antigen, EFTUD1. Neuro Oncol. 2014 Dec;16(12):1618-29.

1073. Guo F, Li X, Peng J, et el. Autophagy regulates vascular endothelial cell eNOS and ET-1 expression induced by laminar shear stress in an ex vivo perfused system. Ann Biomed Eng. 2014 Sep;42(9):1978-88.

1074. Gorbea C, Rechsteiner M, Vallejo JG, et el. Depletion of the 26S proteasome adaptor Ecm29 increases Toll-like receptor 3 signaling. Sci Signal. 2013 Oct 1;6(295):ra86.

1075. Dai Y, Cros MP, Pontoizeau C, et el. Downregulation of transcription factor E4F1 in hepatocarcinoma cells: HBV-dependent effects on autophagy, proliferation and metabolism. Carcinogenesis. 2014 Mar;35(3):635-50.

1076. Wang CY, Xu ZB, Wang JP, et el. Rb deficiency accelerates progression of carcinoma of the urinary bladder in vivo and in vitro through inhibiting autophagy and apoptosis. Int J Oncol. 2017 Apr;50(4):1221-1232.

1077. Chen ZH, Kim HP, Sciurba FC, et el. Egr-1 regulates autophagy in cigarette smoke-induced chronic obstructive pulmonary disease. PLoS One. 2008 Oct 2;3(10):e3316.

1078. Delgado M, Tesfaigzi Y. Is BMF central for anoikis and autophagy. Autophagy. 2014 Jan;10(1):168-9.

1079. Luo S, Rubinsztein DC. BCL2L11/BIM: a novel molecular link between autophagy and apoptosis. Autophagy. 2013 Jan;9(1):104-5.

1080. Lee YH, Morrison BL, Bottaro DP. Synergistic signaling of tumor cell invasiveness by hepatocyte growth factor and hypoxia. J Biol Chem. 2014 Jul 25;289(30):20448-61.

1081. Wang J, Zhou JY, Kho D, et el. Role for DUSP1 (dual-specificity protein phosphatase 1) in the regulation of autophagy. Autophagy. 2016 Oct 2;12(10):1791-1803.

1082. Sobhakumari A, Schickling BM, Love-Homan L, et el. NOX4 mediates cytoprotective autophagy induced by the EGFR inhibitor erlotinib in head and neck cancer cells. Toxicol Appl Pharmacol. 2013 Nov 1;272(3):736-45.

1083. Wang JD, Cao YL, Li Q, et el. A pivotal role of FOS-mediated BECN1/Beclin 1 upregulation in dopamine D2 and D3 receptor agonist-induced autophagy activation. Autophagy. 2015 Nov 2;11(11):2057-2073.

1084. Brot S, Auger C, Bentata R, et el. Collapsin response mediator protein 5 (CRMP5) induces mitophagy, thereby regulating mitochondrion numbers in dendrites. J Biol Chem. 2014 Jan 24;289(4):2261-76.

1085. Bourdeau V, Ferbeyre G. CDK4-CDK6 inhibitors induce autophagy-mediated

degradation of DNMT1 and facilitate the senescence antitumor response. Autophagy. 2016 Oct 2;12(10):1965-1966.

1086. Fischer H, Fumicz J, Rossiter H, et el. Holocrine Secretion of Sebum Is a Unique DNase2-Dependent Mode of Programmed Cell Death. J Invest Dermatol. 2017 Mar;137(3):587-594.

1087. Zhang G, Luo Y, Li G, et el. DHRSX, a novel non-classical secretory protein associated with starvation induced autophagy. Int J Med Sci. 2014 Jul 10;11(9):962-70.

1088. Salcher S, Hermann M, Kiechl-Kohlendorfer U, et el. C10ORF10/DEPP-mediated ROS accumulation is a critical modulator of FOXO3-induced autophagy. Mol Cancer. 2017 May 25;16(1):95.

1089. Lv Q, Hua F, Hu ZW. DEDD, a novel tumor repressor, reverses epithelial-mesenchymal transition by activating selective autophagy. Autophagy. 2012 Nov;8(11):1675-6.

1090. Xia P, Wang S, Huang G, et el. RNF2 is recruited by WASH to ubiquitinate AMBRA1 leading to downregulation of autophagy. Cell Res. 2014 Aug;24(8):943-58.

1091. Evans CS, Holzbaur ELF. Autophagy and mitophagy in ALS. Neurobiol Dis. 2018 Jul 5.

1092. Paul P, Murphy T, Oseni Z, et el. Pathogenic effects of amyotrophic lateral sclerosis-linked mutation in D-amino acid oxidase are mediated by D-serine. Neurobiol Aging. 2014 Apr;35(4):876-85.

1093. Hu C, Yang J, He Q, et el. CysLTR1 Blockage Ameliorates Liver Injury Caused by Aluminum-Overload via PI3K/AKT/mTOR-Mediated Autophagy Activation in Vivo and in Vitro. Mol Pharm. 2018 May 7;15(5):1996-2006.

1094. Lu Y, Cederbaum AI. Autophagy Protects against CYP2E1/Chronic Ethanol-Induced Hepatotoxicity. Biomolecules. 2015 Oct 16;5(4):2659-74.

1095. Campbell GR, Spector SA. Toll-like receptor 8 ligands activate a vitamin D mediated autophagic response that inhibits human immunodeficiency virus type 1. PLoS Pathog. 2012;8(11):e1003017.

1096. Ohtani S, Iwamaru A, Deng W, et el. Tumor suppressor 101F6 and ascorbate synergistically and selectively inhibit non-small cell lung cancer growth by caspase-independent apoptosis and autophagy. Cancer Res. 2007 Jul 1;67(13):6293-303.

1097. Chen ZH, Wu YF, Wang PL, et el. Autophagy is essential for ultrafine particle-induced inflammation and mucus hyperproduction in airway epithelium. Autophagy. 2016;12(2):297-311.

1098. Antonioli M, Albiero F, Nazio F, et el. AMBRA1 interplay with cullin E3 ubiquitin ligases regulates autophagy dynamics. Dev Cell. 2014 Dec 22;31(6):734-46.

1099. Chen KL, Chang WS, Cheung CH, et el. Targeting cathepsin S induces tumor cell autophagy via the EGFR-ERK signaling pathway. Cancer Lett. 2012 Apr 1;317(1):89-98.

1100. Park SH, Chung YJ, Song JY, et el. Müllerian inhibiting substance inhibits an ovarian cancer cell line via β-catenin interacting protein deregulation of the Wnt signal pathway. Int J Oncol. 2017 Mar;50(3):1022-1028.

1101. Capparelli C, Whitaker-Menezes D, Guido C, et el. CTGF drives autophagy, glycolysis and senescence in cancer-associated fibroblasts via HIF1 activation, metabolically promoting tumor growth. Cell Cycle. 2012 Jun 15;11(12):2272-84.

1102. Eaves-Pyles T, Patel J, Arigi E, et el. Immunomodulatory and antibacterial effects of cystatin 9 against Francisella tularensis. Mol Med. 2013 Aug 28;19:263-75.

1103. Zou J, Chen Z, Wei X, et el. Cystatin C as a potential therapeutic mediator against Parkinson's disease via VEGF-induced angiogenesis and enhanced neuronal autophagy in neurovascular units. Cell Death Dis. 2017 Jun 1;8(6):e2854.

1104. Lee SW, Song YS, Lee SY, et el. Downregulation of protein kinase CK2 activity facilitates tumor necrosis factor-α-mediated chondrocyte death through apoptosis and autophagy. PLoS One. 2011 Apr 29;6(4):e19163.

1105. Cheong JK, Zhang F, Chua PJ, et el. Casein kinase 1α-dependent feedback loop controls autophagy in RAS-driven cancers. J Clin Invest. 2015 Apr;125(4):1401-18.

1106. Obba S, Hizir Z, Boyer L, et el. The PRKAA1/AMPKα1 pathway triggers autophagy during CSF1-induced human monocyte differentiation and is a potential target in CMML. Autophagy. 2015;11(7):1114-29.

1107. Escobar KA, Cole NH, Mermier CM, et el. Autophagy and aging: Maintaining the proteome through exercise and caloric restriction. Aging Cell. 2018 Nov 15:e12876.

1108. Dessarthe B, Thedrez A, Latouche JB, et el. CRTAM receptor engagement by Necl-2 on tumor cells triggers cell death of activated Vγ9Vδ2 T cells. J Immunol. 2013 May 1;190(9):4868-76.

1109. Chen F, Wen X, Lin P, et el. Activation of CREBZF Increases Cell Apoptosis in Mouse Ovarian Granulosa Cells by Regulating the ERK1/2 and mTOR Signaling Pathways. Int J Mol Sci. 2018 Nov 8;19(11).

1110. Lee JW, Park HS, Park SA, et el. A Novel Small-Molecule Inhibitor Targeting

CREB-CBP Complex Possesses Anti-Cancer Effects along with Cell Cycle Regulation, Autophagy Suppression and Endoplasmic Reticulum Stress. PLoS One. 2015 Apr 21;10(4):e0122628.

1111. Seto S, Tsujimura K, Koide Y. Coronin-1a inhibits autophagosome formation around Mycobacterium tuberculosis-containing phagosomes and assists mycobacterial survival in macrophages. Cell Microbiol. 2012 May;14(5):710-27.

1112. Christodoulou EG, Yang H, Lademann F, et el. Detection of COPB2 as a KRAS synthetic lethal partner through integration of functional genomics screens. Oncotarget. 2017 May 23;8(21):34283-34297.

1113. Razi M, Chan EY, Tooze SA. Early endosomes and endosomal coatomer are required for autophagy. J Cell Biol. 2009 Apr 20;185(2):305-21.

1114. Dey A, Mustafi SB, Saha S, et el. Inhibition of BMI1 induces autophagy-mediated necroptosis. Autophagy. 2016;12(4):659-70.

1115. Lee AJ, Roylance R, Sander J, et el. CERT depletion predicts chemotherapy benefit and mediates cytotoxic and polyploid-specific cancer cell death through autophagy induction. J Pathol. 2012 Feb;226(3):482-94.

1116. Hiebel C, Kromm T, Stark M, et el. Cannabinoid receptor 1 modulates the autophagic flux independent of mTOR- and BECLIN1-complex. J Neurochem. 2014 Nov;131(4):484-97.

1117. Xie Q, Deng Y, Huang C, et el. Chemerin-induced mitochondrial dysfunction in skeletal muscle. J Cell Mol Med. 2015 May;19(5):986-95.

1118. Alnasser HA, Guan Q, Zhang F, et el. Requirement of clusterin expression for prosurvival autophagy in hypoxic kidney tubular epithelial cells. Am J Physiol Renal Physiol. 2016 Jan 15;310(2):F160-73.

1119. Zhong J, Kong X, Zhang H, et el. Inhibition of CLIC4 enhances autophagy and triggers mitochondrial and ER stress-induced apoptosis in human glioma U251 cells under starvation. PLoS One. 2012;7(6):e39378.

1120. Su J, Xu Y, Zhou L, et el. Suppression of chloride channel 3 expression facilitates sensitivity of human glioma U251 cells to cisplatin through concomitant inhibition of Akt and autophagy. Anat Rec (Hoboken). 2013 Apr;296(4):595-603.

1121. Liang Q, Seo GJ, Choi YJ, et el. Autophagy side of MB21D1/cGAS DNA sensor. Autophagy. 2014 Jun;10(6):1146-7.

1122. Tazi MF, Dakhlallah DA, Caution K, et el. Elevated Mirc1/Mir17-92 cluster expression

negatively regulates autophagy and CFTR (cystic fibrosis transmembrane conductance regulator) function in CF macrophages. Autophagy. 2016 Nov;12(11):2026-2037.

1123. Li S, Wu Y, Ding Y, et el. CerS6 regulates cisplatin resistance in oral squamous cell carcinoma by altering mitochondrial fission and autophagy. J Cell Physiol. 2018 Dec;233(12):9416-9425.

1124. Hu X, Lu Z, Yu S, et el. CERKL regulates autophagy via the NAD-dependent deacetylase SIRT1. Autophagy. 2018 Sep 25:1-13.

1125. Kuo TC, Chen CT, Baron D, et el. Midbody accumulation through evasion of autophagy contributes to cellular reprogramming and tumorigenicity. Nat Cell Biol. 2011 Sep 11;13(10):1214-23.

1126. Lu GD, Ang YH, Zhou J, et el. CCAAT/enhancer binding protein α predicts poorer prognosis and prevents energy starvation-induced cell death in hepatocellular carcinoma. Hepatology. 2015 Mar;61(3):965-78.

1127. Su LY, Li H, Lv L, et el. Melatonin attenuates MPTP-induced neurotoxicity via preventing CDK5-mediated autophagy and SNCA/α-synuclein aggregation. Autophagy. 2015;11(10):1745-59.

1128. Su M, Wang J, Wang C, et el. MicroRNA-221 inhibits autophagy and promotes heart failure by modulating the p27/CDK2/mTOR axis. Cell Death Differ. 2015 Jun;22(6):986-99.

1129. Wilkinson S, Croft DR, O'Prey J, et el. The cyclin-dependent kinase PITSLRE/CDK11 is required for successful autophagy. Autophagy. 2011 Nov;7(11):1295-301.

1130. Gong FR, Wu MY, Shen M, et el. PP2A inhibitors arrest G2/M transition through JNK/Sp1- dependent down-regulation of CDK1 and autophagy-dependent up-regulation of p21. Oncotarget. 2015 Jul 30;6(21):18469-83.

1131. Uekita T, Fujii S, Miyazawa Y, et el. Suppression of autophagy by CUB domain-containing protein 1 signaling is essential for anchorage-independent survival of lung cancer cells. Cancer Sci. 2013 Jul;104(7):865-70.

1132. Chung YH, Yoon SY, Choi B, et el. Microtubule-associated protein light chain 3 regulates Cdc42-dependent actin ring formation in osteoclast. Int J Biochem Cell Biol. 2012 Jun;44(6):989-97.

1133. Jinwal UK, Abisambra JF, Zhang J, et el. Cdc37/Hsp90 protein complex disruption triggers an autophagic clearance cascade for TDP-43 protein. J Biol Chem. 2012 Jul

13;287(29):24814-20.

1134. Wu CY, Yan J, Yang YF, et el. Overexpression of KAI1 induces autophagy and increases MiaPaCa-2 cell survival through the phosphorylation of extracellular signal-regulated kinases. Biochem Biophys Res Commun. 2011 Jan 21;404(3):802-8.

1135. Alinari L, Baiocchi RA, Praetorius-Ibba M. FTY720-induced blockage of autophagy enhances anticancer efficacy of milatuzumab in mantle cell lymphoma: is FTY720 the next autophagy-blocking agent in lymphoma treatment. Autophagy. 2012 Mar;8(3):416-7.

1136. Zhang X, Fan J, Wang S, et el. Targeting CD47 and Autophagy Elicited Enhanced Antitumor Effects in Non-Small Cell Lung Cancer. Cancer Immunol Res. 2017 May;5(5):363-375.

1137. Naik PP, Mukhopadhyay S, Panda PK, et el. Autophagy regulates cisplatin-induced stemness and chemoresistance via the upregulation of CD44, ABCB1 and ADAM17 in oral squamous cell carcinoma. Cell Prolif. 2018 Feb;51(1).

1138. Klug-Micu GM, Stenger S, Sommer A, et el. CD40 ligand and interferon-γ induce an antimicrobial response against Mycobacterium tuberculosis in human monocytes. Immunology. 2013 May;139(1):121-8.

1139. Portillo JA, Okenka G, Reed E, et el. The CD40-autophagy pathway is needed for host protection despite IFN-Γ-dependent immunity and CD40 induces autophagy via control of P21 levels. PLoS One. 2010 Dec 31;5(12):e14472.

1140. Sanjurjo L, Amézaga N, Aran G, et el. The human CD5L/AIM-CD36 axis: A novel autophagy inducer in macrophages that modulates inflammatory responses. Autophagy. 2015;11(3):487-502.

1141. Shin DM, Yuk JM, Lee HM, et el. Mycobacterial lipoprotein activates autophagy via TLR2/1/CD14 and a functional vitamin D receptor signalling. Cell Microbiol. 2010 Nov;12(11):1648-65.

1142. Ge JN, Huang D, Xiao T, et el. [Effect of starvation-induced autophagy on cell cycle of tumor cells]. Ai Zheng. 2008 Aug;27(8):788-94.

1143. Weng L, Han YP, Enomoto A, et el. Negative regulation of amino acid signaling by MAPK-regulated 4F2hc/Girdin complex. PLoS Biol. 2018 Mar 14;16(3):e2005090.

1144. Peuget S, Bonacci T, Soubeyran P, et el. Oxidative stress-induced p53 activity is enhanced by a redox-sensitive TP53INP1 SUMOylation. Cell Death Differ. 2014 Jul;21(7):1107-18.

1145. Zhang G, Liu J, Zhang Y, et el. Cbl-b-dependent degradation of FLIP(L) is involved in

ATO-induced autophagy in leukemic K562 and gastric cancer cells. FEBS Lett. 2012 Sep 21;586(19):3104-10.

1146. Greer AH, Yong T, Fennell K, et el. Knockdown of core binding factorβ alters sphingolipid metabolism. J Cell Physiol. 2013 Dec;228(12):2350-64.

1147. Zhang J, Ma K, Qi T, et el. P62 regulates resveratrol-mediated Fas/Cav-1 complex formation and transition from autophagy to apoptosis. Oncotarget. 2015 Jan 20;6(2):789-801.

1148. Dias MV, Teixeira BL, Rodrigues BR, et el. PRNP/prion protein regulates the secretion of exosomes modulating CAV1/caveolin-1-suppressed autophagy. Autophagy. 2016 Nov;12(11):2113-2128.

1149. Yang PM, Tseng HH, Peng CW, et el. Dietary flavonoid fisetin targets caspase-3-deficient human breast cancer MCF-7 cells by induction of caspase-7-associated apoptosis and inhibition of autophagy. Int J Oncol. 2012 Feb;40(2):469-78.

1150. Ye YC, Wang HJ, Chen L, et el. Negatively-regulated necroptosis by autophagy required caspase-6 activation in TNFα-treated murine fibrosarcoma L929 cells. Int Immunopharmacol. 2013 Nov;17(3):548-55.

1151. Guo W, Dong A, Pan X, et el. Role of caspase-10 in the death of acute leukemia cells. Oncol Lett. 2016 Aug;12(2):1623-1629.

1152. Proikas-Cezanne T, Takacs Z, Dönnes P, et el. WIPI proteins: essential PtdIns3P effectors at the nascent autophagosome. J Cell Sci. 2015 Jan 15;128(2):207-17.

1153. Das A, Pushparaj C, Herreros J, et el. T-type calcium channel blockers inhibit autophagy and promote apoptosis of malignant melanoma cells. Pigment Cell Melanoma Res. 2013 Nov;26(6):874-85.

1154. Wang TK, Cheng CK, Chi TH, et el. Effects of carbonic anhydrase-related protein VIII on human cells harbouring an A8344G mitochondrial DNA mutation. Biochem J. 2014 Apr 1;459(1):149-60.

1155. Jiao H, You H. p32: A new player in autophagy. Mol Cell Oncol. 2015 Jul 6;3(3):e1061097.

1156. Gao D, Inuzuka H, Tan MK, et el. mTOR drives its own activation via SCF(βTrCP)-dependent degradation of the mTOR inhibitor DEPTOR. Mol Cell. 2011 Oct 21;44(2):290-303.

1157. Gou WF, Yang XF, Shen DF, et el. The roles of BTG3 expression in gastric cancer: a

potential marker for carcinogenesis and a target molecule for gene therapy. Oncotarget. 2015 Aug 14;6(23):19841-67.

1158. Hu Z, Cai M, Deng L, et el. The fucosylated CD147 enhances the autophagy in epithelial ovarian cancer cells. Oncotarget. 2016 Dec 13;7(50):82921-82932.

1159. Goulielmaki M, Koustas E, Moysidou E, et el. BRAF associated autophagy exploitation: BRAF and autophagy inhibitors synergise to efficiently overcome resistance of BRAF mutant colorectal cancer cells. Oncotarget. 2016 Feb 23;7(8):9188-221.

1160. Delorme-Axford E, Morosky S, Bomberger J, et el. BPIFB3 regulates autophagy and coxsackievirus B replication through a noncanonical pathway independent of the core initiation machinery. MBio. 2014 Dec 9;5(6):e02147.

1161. Jiao G, Guo W, Ren T, et el. BMPR2 inhibition induced apoptosis and autophagy via destabilization of XIAP in human chondrosarcoma cells. Cell Death Dis. 2014 Dec 11;5:e1571.

1162. Song TF, Huang LW, Yuan Y, et el. LncRNA MALAT1 regulates smooth muscle cell phenotype switch via activation of autophagy. Oncotarget. 2017 Dec 14;9(4):4411-4426.

1163. Deng G, Zeng S, Qu Y, et el. BMP4 promotes hepatocellular carcinoma proliferation by autophagy activation through JNK1-mediated Bcl-2 phosphorylation. J Exp Clin Cancer Res. 2018 Jul 16;37(1):156.

1164. Yao L, Wang J, Tian BY, et el. Activation of the Nrf2-ARE Signaling Pathway Prevents Hyperphosphatemia-Induced Vascular Calcification by Inducing Autophagy in Renal Vascular Smooth Muscle Cells. J Cell Biochem. 2017 Dec;118(12):4708-4715.

1165. Scott I, Webster BR, Chan CK, et el. GCN5-like protein 1 (GCN5L1) controls mitochondrial content through coordinated regulation of mitochondrial biogenesis and mitophagy. J Biol Chem. 2014 Jan 31;289(5):2864-72.

1166. Hay-Koren A, Bialik S, Levin-Salomon V, et el. Changes in cIAP2, survivin and BimEL expression characterize the switch from autophagy to apoptosis in prolonged starvation. J Intern Med. 2017 May;281(5):458-470.

1167. Sun Y, Li Q, Zhang J, et el. Autophagy regulatory molecule, TMEM74, interacts with BIK and inhibits BIK-induced apoptosis. Cell Signal. 2017 Aug;36:34-41.

1168. Lamy L, Ngo VN, Emre NC, et el. Control of autophagic cell death by caspase-10 in multiple myeloma. Cancer Cell. 2013 Apr 15;23(4):435-49.

1169. Crawford AC, Riggins RB, Shajahan AN, et el. Co-inhibition of BCL-W and BCL2

restores antiestrogen sensitivity through BECN1 and promotes an autophagy-associated necrosis. PLoS One. 2010 Jan 6;5(1):e8604.

1170. Liu X, Hu X, Kuang Y, et el. BCLB, methylated in hepatocellular carcinoma, is a starvation stress sensor that induces apoptosis and autophagy through the AMPK-mTOR signaling cascade. Cancer Lett. 2017 Jun 1;395:63-71.

1171. Jones SE, Fleuren EDG, Frankum J, et el. ATR Is a Therapeutic Target in Synovial Sarcoma. Cancer Res. 2017 Dec 15;77(24):7014-7026.

1172. Nick AM, Stone RL, Armaiz-Pena G, et el. Silencing of p130cas in ovarian carcinoma: a novel mechanism for tumor cell death. J Natl Cancer Inst. 2011 Nov 2;103(21):1596-612.

1173. Yu X, Muñoz-Alarcón A, Ajayi A, et el. Inhibition of autophagy via p53-mediated disruption of ULK1 in a SCA7 polyglutamine disease model. J Mol Neurosci. 2013 Jul;50(3):586-99.

1174. Ou Z, Luo M, Niu X, et el. Autophagy Promoted the Degradation of Mutant ATXN3 in Neurally Differentiated Spinocerebellar Ataxia-3 Human Induced Pluripotent Stem Cells. Biomed Res Int. 2016;2016:6701793.

1175. Xu X, Wang H, Liu S, et el. TP53-dependent autophagy links the ATR-CHEK1 axis activation to proinflammatory VEGFA production in human bronchial epithelial cells exposed to fine particulate matter (PM2.5). Autophagy. 2016 Oct 2;12(10):1832-1848.

1176. Mangieri LR, Mader BJ, Thomas CE, et el. ATP6V0C knockdown in neuroblastoma cells alters autophagy-lysosome pathway function and metabolism of proteins that accumulate in neurodegenerative disease. PLoS One. 2014 Apr 2;9(4):e93257.

1177. Hsin IL, Sheu GT, Jan MS, et el. Inhibition of lysosome degradation on autophagosome formation and responses to GMI, an immunomodulatory protein from Ganoderma microsporum. Br J Pharmacol. 2012 Nov;167(6):1287-300.

1178. Cannata Serio M, Rujano MA, Simons M. Mutations in ATP6AP2 cause autophagic liver disease in humans. Autophagy. 2018;14(6):1088-1089.

1179. Lefebvre V, Du Q, Baird S, et el. Genome-wide RNAi screen identifies ATPase inhibitory factor 1 (ATPIF1) as essential for PARK2 recruitment and mitophagy. Autophagy. 2013 Nov 1;9(11):1770-9.

1180. Zhou H, Zhu J, Yue S, et el. The Dichotomy of Endoplasmic Reticulum Stress Response in Liver Ischemia-Reperfusion Injury. Transplantation. 2016 Feb;100(2):365-72.

1181. Sood V, Sharma KB, Gupta V, et el. ATF3 negatively regulates cellular antiviral signaling

and autophagy in the absence of type I interferons. Sci Rep. 2017 Aug 18;7(1):8789.

1182. Chen TC, Hung YC, Lin TY, et el. Human papillomavirus infection and expression of ATPase family AAA domain containing 3A, a novel anti-autophagy factor, in uterine cervical cancer. Int J Mol Med. 2011 Nov;28(5):689-96.

1183. Qiu F, Chen YR, Liu X, et el. Arginine starvation impairs mitochondrial respiratory function in ASS1-deficient breast cancer cells. Sci Signal. 2014 Apr 1;7(319):ra31.

1184. Huang HL, Chen WC, Hsu HP, et el. Argininosuccinate lyase is a potential therapeutic target in breast cancer. Oncol Rep. 2015 Dec;34(6):3131-9.

1185. Melland-Smith M, Ermini L, Chauvin S, et el. Disruption of sphingolipid metabolism augments ceramide-induced autophagy in preeclampsia. Autophagy. 2015 Apr 3;11(4):653-69.

1186. Xiong Y, Yepuri G, Forbiteh M, et el. ARG2 impairs endothelial autophagy through regulation of MTOR and PRKAA/AMPK signaling in advanced atherosclerosis. Autophagy. 2014;10(12):2223-38.

1187. Liu K, Zhao E, Ilyas G, et el. Impaired macrophage autophagy increases the immune response in obese mice by promoting proinflammatory macrophage polarization. Autophagy. 2015;11(2):271-84.

1188. Blessing AM, Rajapakshe K, Reddy Bollu L, et el. Transcriptional regulation of core autophagy and lysosomal genes by the androgen receptor promotes prostate cancer progression. Autophagy. 2017 Mar 4;13(3):506-521.

1189. Paulo JA, Gygi SP. Nicotine-induced protein expression profiling reveals mutually altered proteins across four human cell lines. Proteomics. 2017 Jan;17(1-2).

1190. Yang L, Su T, Lv D, et el. ERK1/2 mediates lung adenocarcinoma cell proliferation and autophagy induced by apelin-13. Acta Biochim Biophys Sin (Shanghai). 2014 Feb;46(2):100-11.

1191. Crauwels P, Bohn R, Thomas M, et el. Apoptotic-like Leishmania exploit the host's autophagy machinery to reduce T-cell-mediated parasite elimination. Autophagy. 2015;11(2):285-97.

1192. Zhu JF, Huang W, Yi HM, et el. Annexin A1-suppressed autophagy promotes nasopharyngeal carcinoma cell invasion and metastasis by PI3K/AKT signaling activation. Cell Death Dis. 2018 Nov 20;9(12):1154.

1193. Xia Y, Chen K, Zhang MH, et el. MicroRNA-124 involves in ankylosing spondylitis by

targeting ANTXR2. Mod Rheumatol. 2015 Sep;25(5):784-9.

1194. Zhang Y, Guo X, Yan W, et el. ANGPTL8 negatively regulates NF-κB activation by facilitating selective autophagic degradation of IKKγ. Nat Commun. 2017 Dec 18;8(1):2164.

1195. Sonigo C, Beau I, Grynberg M, et el. AMH prevents primordial ovarian follicle loss and fertility alteration in cyclophosphamide-treated mice. FASEB J. 2018 Aug 16:fj201801089R.

1196. Otomo A, Kunita R, Suzuki-Utsunomiya K, et el. Defective relocalization of ALS2/alsin missense mutants to Rac1-induced macropinosomes accounts for loss of their cellular function and leads to disturbed amphisome formation. FEBS Lett. 2011 Mar 9;585(5):730-6.

1197. Morgan AH, Hammond VJ, Sakoh-Nakatogawa M, et el. A novel role for 12/15-lipoxygenase in regulating autophagy. Redox Biol. 2015;4:40-7.

1198. Lorente M, Torres S, Salazar M, et el. Stimulation of ALK by the growth factor midkine renders glioma cells resistant to autophagy-mediated cell death. Autophagy. 2011 Sep;7(9):1071-3.

1199. Liu M, Lu S, He W, et el. ULK1-regulated autophagy: A mechanism in cellular protection for ALDH2 against hyperglycemia. Toxicol Lett. 2018 Feb;283:106-115.

1200. Eby KG, Rosenbluth JM, Mays DJ, et el. ISG20L1 is a p53 family target gene that modulates genotoxic stress-induced autophagy. Mol Cancer. 2010 Apr 29;9:95.

1201. Demishtein A, Fraiberg M, Berko D, et el. SQSTM1/p62-mediated autophagy compensates for loss of proteasome polyubiquitin recruiting capacity. Autophagy. 2017 Oct 3;13(10):1697-1708.

1202. Wu FQ, Fang T, Yu LX, et el. ADRB2 signaling promotes HCC progression and sorafenib resistance by inhibiting autophagic degradation of HIF1α. J Hepatol. 2016 Aug;65(2):314-24.

1203. Chen ML, Yi L, Jin X, et el. Resveratrol attenuates vascular endothelial inflammation by inducing autophagy through the cAMP signaling pathway. Autophagy. 2013 Dec;9(12):2033-45.

1204. Li X, Yu W, Qian X, et el. Nucleus-Translocated ACSS2 Promotes Gene Transcription for Lysosomal Biogenesis and Autophagy. Mol Cell. 2017 Jun 1;66(5):684-697.e9.

1205. Deng M, Huang L, Ning B, et el. β-asarone improves learning and memory and reduces

Acetyl Cholinesterase and Beta-amyloid 42 levels in APP/PS1 transgenic mice by regulating Beclin-1-dependent autophagy. Brain Res. 2016 Dec 1;1652:188-194.

1206. Desideri E, Filomeni G, Ciriolo MR. Glutathione participates in the modulation of starvation-induced autophagy in carcinoma cells. Autophagy. 2012 Dec;8(12):1769-81.

1207. Funasaka T, Tsuka E, Wong RW. Regulation of autophagy by nucleoporin Tpr. Sci Rep. 2012;2:878.

1208. Baldwin A, Grueneberg DA, Hellner K, et el. Kinase requirements in human cells: V. Synthetic lethal interactions between p53 and the protein kinases SGK2 and PAK3. Proc Natl Acad Sci U S A. 2010 Jul 13;107(28):12463-8.

1209. Gan W, Zhang C, Siu KY, et el. ULK1 phosphorylates Sec23A and mediates autophagy-induced inhibition of ER-to-Golgi traffic. BMC Cell Biol. 2017 May 10;18(1):22.

1210. Cao L, Zhang L, Zhao X, et el. A Hybrid Chalcone Combining the Trimethoxyphenyl and Isatinyl Groups Targets Multiple Oncogenic Proteins and Pathways in Hepatocellular Carcinoma Cells. PLoS One. 2016 Aug 15;11(8):e0161025.

1211. Lin SY, Li TY, Liu Q, et el. GSK3-TIP60-ULK1 signaling pathway links growth factor deprivation to autophagy. Science. 2012 Apr 27;336(6080):477-81.

1212. Wang J, Davis S, Menon S, et el. Ypt1/Rab1 regulates Hrr25/CK1δ kinase activity in ER-Golgi traffic and macroautophagy. J Cell Biol. 2015 Jul 20;210(2):273-85.

1213. Tam RC, Li MW, Gao YP, et el. Human CLEC16A regulates autophagy through modulating mTOR activity. Exp Cell Res. 2017 Mar 15;352(2):304-312.

1214. Cai W, Wei Y, Jarnik M, et el. The GATOR2 Component Wdr24 Regulates TORC1 Activity and Lysosome Function. PLoS Genet. 2016 May 11;12(5):e1006036.

1215. Pérez-Victoria FJ, Schindler C, Magadán JG, et el. Ang2/fat-free is a conserved subunit of the Golgi-associated retrograde protein complex. Mol Biol Cell. 2010 Oct 1;21(19):3386-95.

1216. Muñoz-Braceras S, Calvo R, Escalante R. TipC and the chorea-acanthocytosis protein VPS13A regulate autophagy in Dictyostelium and human HeLa cells. Autophagy. 2015;11(6):918-27.

1217. Warr MR, Binnewies M, Flach J, et el. FOXO3A directs a protective autophagy program in haematopoietic stem cells. Nature. 2013 Feb 21;494(7437):323-7.

1218. Joffre C, Dupont N, Hoa L, et el. The Pro-apoptotic STK38 Kinase Is a New Beclin1 Partner Positively Regulating Autophagy. Curr Biol. 2015 Oct 5;25(19):2479-92.

1219. Alexaki A, Gupta SD, Majumder S, et el. Autophagy regulates sphingolipid levels in the liver. J Lipid Res. 2014 Dec;55(12):2521-31.

1220. Cowherd RB, Asmar MM, Alderman JM, et el. Adiponectin lowers glucose production by increasing SOGA. Am J Pathol. 2010 Oct;177(4):1936-45.

1221. Quidville V, Alsafadi S, Goubar A, et el. Targeting the deregulated spliceosome core machinery in cancer cells triggers mTOR blockade and autophagy. Cancer Res. 2013 Apr 1;73(7):2247-58.

1222. Akalay I, Janji B, Hasmim M, et el. Epithelial-to-mesenchymal transition and autophagy induction in breast carcinoma promote escape from T-cell-mediated lysis. Cancer Res. 2013 Apr 15;73(8):2418-27.

1223. Li-Harms X, Milasta S, Lynch J, et el. Mito-protective autophagy is impaired in erythroid cells of aged mtDNA-mutator mice. Blood. 2015 Jan 1;125(1):162-74.

1224. Oueslati A, Schneider BL, Aebischer P, et el. Polo-like kinase 2 regulates selective autophagic α-synuclein clearance and suppresses its toxicity in vivo. Proc Natl Acad Sci U S A. 2013 Oct 8;110(41):E3945-54.

1225. Héraud C, Griffiths A, Pandruvada SN, et el. Severe neurodegeneration with impaired autophagy mechanism triggered by ostm1 deficiency. J Biol Chem. 2014 May 16;289(20):13912-25.

1226. De Leo MG, Staiano L, Vicinanza M, et el. Autophagosome-lysosome fusion triggers a lysosomal response mediated by TLR9 and controlled by OCRL. Nat Cell Biol. 2016 Aug;18(8):839-850.

1227. Huang J, Wang T, Wright AC, et el. Myocardin is required for maintenance of vascular and visceral smooth muscle homeostasis during postnatal development. Proc Natl Acad Sci U S A. 2015 Apr 7;112(14):4447-52.

1228. Lee SJ, Park MH, Kim HJ, et el. Metallothionein-3 regulates lysosomal function in cultured astrocytes under both normal and oxidative conditions. Glia. 2010 Aug;58(10):1186-96.

1229. Brandenstein L, Schweizer M, Sedlacik J, et el. Lysosomal dysfunction and impaired autophagy in a novel mouse model deficient for the lysosomal membrane protein Cln7. Hum Mol Genet. 2016 Feb 15;25(4):777-91.

1230. Bonhoure N, Byrnes A, Moir RD, et el. Loss of the RNA polymerase III repressor MAF1 confers obesity resistance. Genes Dev. 2015 May 1;29(9):934-47.

1231. Laguna A, Schintu N, Nobre A, et el. Dopaminergic control of autophagic-lysosomal function implicates Lmx1b in Parkinson's disease. Nat Neurosci. 2015 Jun;18(6):826-35.

1232. Wang N, Zimmerman K, Raab RW, et el. Lacritin rescues stressed epithelia via rapid forkhead box O3 (FOXO3)-associated autophagy that restores metabolism. J Biol Chem. 2013 Jun 21;288(25):18146-61.

1233. Bernard A, Jin M, González-Rodríguez P, et el. Rph1/KDM4 mediates nutrient-limitation signaling that leads to the transcriptional induction of autophagy. Curr Biol. 2015 Mar 2;25(5):546-55.

1234. Wu J, Carlock C, Zhou C, et el. IL-33 is required for disposal of unnecessary cells during ovarian atresia through regulation of autophagy and macrophage migration. J Immunol. 2015 Mar 1;194(5):2140-7.

1235. Jrad-Lamine A, Henry-Berger J, Gourbeyre P, et el. Deficient tryptophan catabolism along the kynurenine pathway reveals that the epididymis is in a unique tolerogenic state. J Biol Chem. 2011 Mar 11;286(10):8030-42.

1236. Mashimo T, Hadjebi O, Amair-Pinedo F, et el. Progressive Purkinje cell degeneration in tambaleante mutant mice is a consequence of a missense mutation in HERC1 E3 ubiquitin ligase. PLoS Genet. 2009 Dec;5(12):e1000784.

1237. Kim KW, Paul P, Qiao J, et el. Autophagy mediates paracrine regulation of vascular endothelial cells. Lab Invest. 2013 Jun;93(6):639-45.

1238. Todd LR, Damin MN, Gomathinayagam R, et el. Growth factor erv1-like modulates Drp1 to preserve mitochondrial dynamics and function in mouse embryonic stem cells. Mol Biol Cell. 2010 Apr 1;21(7):1225-36.

1239. Niu G, Zhang H, Liu D, et el. Tid1, the Mammalian Homologue of Drosophila Tumor Suppressor Tid56, Mediates Macroautophagy by Interacting with Beclin1-containing Autophagy Protein Complex. J Biol Chem. 2015 Jul 17;290(29):18102-10.

1240. Matsui T, Noguchi K, Fukuda M. Dennd3 functions as a guanine nucleotide exchange factor for small GTPase Rab12 in mouse embryonic fibroblasts. J Biol Chem. 2014 May 16;289(20):13986-95.

1241. Xiao L, Xian H, Lee KY, et el. Death-associated Protein 3 Regulates Mitochondrial-encoded Protein Synthesis and Mitochondrial Dynamics. J Biol Chem.

2015 Oct 9;290(41):24961-74.

1242. Zhang Y, Fang F, Goldstein JL, et el. Reduced autophagy in livers of fasted, fat-depleted, ghrelin-deficient mice: reversal by growth hormone. Proc Natl Acad Sci U S A. 2015 Jan 27;112(4):1226-31.

1243. Begun J, Lassen KG, Jijon HB, et el. Integrated Genomics of Crohn's Disease Risk Variant Identifies a Role for CLEC12A in Antibacterial Autophagy. Cell Rep. 2015 Jun 30;11(12):1905-18.

1244. Ren J, Hascall VC, Wang A. Cyclin D3 mediates synthesis of a hyaluronan matrix that is adhesive for monocytes in mesangial cells stimulated to divide in hyperglycemic medium. J Biol Chem. 2009 Jun 12;284(24):16621-32.

1245. Yuk JM, Shin DM, Lee HM, et el. Vitamin D3 induces autophagy in human monocytes/macrophages via cathelicidin. Cell Host Microbe. 2009 Sep 17;6(3):231-43.

1246. Venco P, Bonora M, Giorgi C, et el. Mutations of C19orf12, coding for a transmembrane glycine zipper containing mitochondrial protein, cause mis-localization of the protein, inability to respond to oxidative stress and increased mitochondrial Ca2+. Front Genet. 2015 May 19;6:185.

1247. Sebti S, Prébois C, Pérez-Gracia E, et el. BAT3 modulates p300-dependent acetylation of p53 and autophagy-related protein 7 (ATG7) during autophagy. Proc Natl Acad Sci U S A. 2014 Mar 18;111(11):4115-20.

1248. Oppenheim RW, Blomgren K, Ethell DW, et el. Developing postmitotic mammalian neurons in vivo lacking Apaf-1 undergo programmed cell death by a caspase-independent, nonapoptotic pathway involving autophagy. J Neurosci. 2008 Feb 6;28(6):1490-7.

1249. Grevengoed TJ, Cooper DE, Young PA, et el. Loss of long-chain acyl-CoA synthetase isoform 1 impairs cardiac autophagy and mitochondrial structure through mechanistic target of rapamycin complex 1 activation. FASEB J. 2015 Nov;29(11):4641-53.

1250. Lei Y, Wen H, Yu Y, et el. The mitochondrial proteins NLRX1 and TUFM form a complex that regulates type I interferon and autophagy. Immunity. 2012 Jun 29;36(6):933-46.

1251. Douarre C, Sourbier C, Dalla Rosa I, et el. Mitochondrial topoisomerase I is critical for mitochondrial integrity and cellular energy metabolism. PLoS One. 2012;7(7):e41094.

1252. Xu Y, Liu XD, Gong X, et el. Signaling pathway of autophagy associated with innate immunity. Autophagy. 2008 Jan;4(1):110-2.

1253. Shibuya Y, Chang CC, Huang LH, et el. Inhibiting ACAT1/SOAT1 in microglia

stimulates autophagy-mediated lysosomal proteolysis and increases Aβ1-42 clearance. J Neurosci. 2014 Oct 22;34(43):14484-501.

1254. Kim YM, Stone M, Hwang TH, et el. SH3BP4 is a negative regulator of amino acid-Rag GTPase-mTORC1 signaling. Mol Cell. 2012 Jun 29;46(6):833-46.

1255. Birukawa NK, Murase K, Sato Y, et el. Activated hepatic stellate cells are dependent on self-collagen, cleaved by membrane type 1 matrix metalloproteinase for their growth. J Biol Chem. 2014 Jul 18;289(29):20209-21.

1256. Brown DI, Lassègue B, Lee M, et el. Poldip2 knockout results in perinatal lethality, reduced cellular growth and increased autophagy of mouse embryonic fibroblasts. PLoS One. 2014 May 5;9(5):e96657.

1257. Woldt E, Sebti Y, Solt LA, et el. Rev-erb-α modulates skeletal muscle oxidative capacity by regulating mitochondrial biogenesis and autophagy. Nat Med. 2013 Aug;19(8):1039-46.

1258. Criado O, Aguado C, Gayarre J, et el. Lafora bodies and neurological defects in malin-deficient mice correlate with impaired autophagy. Hum Mol Genet. 2012 Apr 1;21(7):1521-33.

1259. Thoreen CC, Sabatini DM. Huntingtin aggregates ask to be eaten. Nat Genet. 2004 Jun;36(6):553-4.

1260. Ryu H, Rosas HD, Hersch SM, et el. The therapeutic role of creatine in Huntington's disease. Pharmacol Ther. 2005 Nov;108(2):193-207.

1261. Soll C, Jang JH, Riener MO, et el. Serotonin promotes tumor growth in human hepatocellular cancer. Hepatology. 2010 Apr;51(4):1244-54.

1262. Galvez AS, Diwan A, Odley AM, et el. Cardiomyocyte degeneration with calpain deficiency reveals a critical role in protein homeostasis. Circ Res. 2007 Apr 13;100(7):1071-8.

1263. Sirohi K, Chalasani ML, Sudhakar C, et el. M98K-OPTN induces transferrin receptor degradation and RAB12-mediated autophagic death in retinal ganglion cells. Autophagy. 2013 Apr;9(4):510-27.
