# Supplementary material for: Deciphering the autophagy regulatory network via single-cell transcriptome analysis reveals a requirement for autophagy homeostasis in spermatogenesis
Source: Theranostics. 2021 Mar 5;11(10):5010–27. doi: 10.7150/thno.55645 (PMC7978313; doi:10.7150/thno.55645)
Supplement: Supplementary file 3 — Supplementary references 2. [file thnov11p5010s3.pdf]

Supplemental references

1. Sun WL, Wang L, Luo J, et al. Ambra1 modulates the sensitivity of breast cancer cells to epirubicin by regulating autophagy via ATG12. Cancer Sci. 2018 Oct;109(10):3129-3138.
2. Schoenherr C, Byron A, Sandilands E, et al. Ambra1 spatially regulates Src activity and Src/FAK-mediated cancer cell invasion via trafficking networks. Elife. 2017 Mar 31;6.
3. Martinez J, Malireddi RK, Lu Q, et al. Molecular characterization of LC3-associated phagocytosis reveals distinct roles for Rubicon, NOX2 and autophagy proteins. Nat Cell Biol. 2015 Jul;17(7):893-906.
4. Cianfanelli V, Fuoco C, Lorente M, et al. AMBRA1 links autophagy to cell proliferation and tumorigenesis by promoting c-Myc dephosphorylation and degradation. Nat Cell Biol. 2015 Jan;17(1):20-30.
5. Yazdankhah M, Farioli-Vecchioli S, Tonchev AB, et al. The autophagy regulators Ambra1 and Beclin 1 are required for adult neurogenesis in the brain subventricular zone. Cell Death Dis. 2014 Sep 4;5:e1403.
6. Strappazzon F, Nazio F, Corrado M, et al. AMBRA1 is able to induce mitophagy via LC3 binding, regardless of PARKIN and p62/SQSTM1. Cell Death Differ. 2015 Mar;22(3):419-32.
7. Sanchez CG, Penfornis P, Oskowitz AZ, et al. Activation of autophagy in mesenchymal stem cells provides tumor stromal support. Carcinogenesis. 2011 Jul;32(7):964-72.
8. Cho YY, Kim DJ, Lee HS, et al. Autophagy and cellular senescence mediated by Sox2 suppress malignancy of cancer cells. PLoS One. 2013;8(2):e57172.
9. Song C, Mitter SK, Qi X, et al. Oxidative stress-mediated NFkappaB phosphorylation upregulates p62/SQSTM1 and promotes retinal pigmented epithelial cell survival through increased autophagy. PLoS One. 2017;12(2):e0171940.
10. Wang S, Ji LY, Li L, et al. Oxidative stress, autophagy and pyroptosis in the neovascularization of oxygeninduced retinopathy in mice. Mol Med Rep. 2019 Feb;19(2):927-934.
11. Yang K, Yu B, Cheng C, et al. Mir505-3p regulates axonal development via inhibiting the autophagy pathway by targeting Atg12. Autophagy. 2017 Oct 3;13(10):1679-1696.
12. Liu H, Zhang X, Zhang S, et al. Oxidative Stress Mediates Microcystin-LR-Induced Endoplasmic Reticulum Stress and Autophagy in KK-1 Cells and C57BL/6 Mice Ovaries. Front Physiol. 2018;9:1058.
13. Wold MS, Lim J, Lachance V, et al. ULK1-mediated phosphorylation of ATG14 promotes autophagy and is impaired in Huntington's disease models. Mol Neurodegener. 2016 Dec 9;11(1):76.
14. Li B, Wu X, Chen H, et al. miR199a-5p inhibits hepatic insulin sensitivity via suppression of ATG14-mediated autophagy. Cell Death Dis. 2018 Mar 14;9(3):405.
15. Xiong X, Tao R, DePinho RA, et al. The autophagy-related gene 14 (Atg14) is regulated by forkhead box O transcription factors and circadian rhythms and plays a critical role in hepatic autophagy and lipid metabolism. J Biol Chem. 2012 Nov 9;287(46):39107-14.
16. Conway KL, Kuballa P, Song JH, et al. Atg16l1 is required for autophagy in intestinal epithelial cells and protection of mice from Salmonella infection. Gastroenterology. 2013 Dec;145(6):1347-57.
17. Matsuzawa-Ishimoto Y, Shono Y, Gomez LE, et al. Autophagy protein ATG16L1 prevents

necroptosis in the intestinal epithelium. J Exp Med. 2017 Dec 4;214(12):3687-3705.
18. Li Y, Zhou D, Ren Y, et al. Mir223 restrains autophagy and promotes CNS inflammation by targeting ATG16L1. Autophagy. 2019 Mar;15(3):478-492.
19. Litwinoff EMS, Gold MY, Singh K, et al. Myeloid ATG16L1 does not affect adipose tissue inflammation or body mass in mice fed high fat diet. Obes Res Clin Pract. 2018 Mar - Apr;12(2):174-186.
20. Kabat AM, Harrison OJ, Riffelmacher T, et al. The autophagy gene Atg16l1 differentially regulates Treg and TH2 cells to control intestinal inflammation. Elife. 2016 Feb 24;5:e12444.
21. Li N, Wu X, Holzer RG, et al. Loss of acinar cell IKKalpha triggers spontaneous pancreatitis in mice. J Clin Invest. 2013 May;123(5):2231-43.
22. Ishibashi K, Fujita N, Kanno E, et al. Atg16L2, a novel isoform of mammalian Atg16L that is not essential for canonical autophagy despite forming an Atg12-5-16L2 complex. Autophagy. 2011 Dec;7(12):1500-13.
23. Frudd K, Burgoyne T, Burgoyne JR. Oxidation of Atg3 and Atg7 mediates inhibition of autophagy. Nat Commun. 2018 Jan 8;9(1):95.
24. Xiu YL, Sun KX, Chen X, et al. Upregulation of the lncRNA Meg3 induces autophagy to inhibit tumorigenesis and progression of epithelial ovarian carcinoma by regulating activity of ATG3. Oncotarget. 2017 May 9;8(19):31714-31725.
25. Ma K, Fu W, Tang M, et al. PTK2-mediated degradation of ATG3 impedes cancer cells susceptible to DNA damage treatment. Autophagy. 2017 Mar 4;13(3):579-591.
26. Liu K, Zhao Q, Liu P, et al. ATG3-dependent autophagy mediates mitochondrial homeostasis in pluripotency acquirement and maintenance. Autophagy. 2016 Nov;12(11):2000-2008.
27. Li Y, Zhang Y, Wang L, et al. Autophagy impairment mediated by S-nitrosation of ATG4B leads to neurotoxicity in response to hyperglycemia. Autophagy. 2017 Jul 3;13(7):1145-1160.
28. Yang SW, Ping YF, Jiang YX, et al. ATG4A promotes tumor metastasis by inducing the epithelial-mesenchymal transition and stem-like properties in gastric cells. Oncotarget. 2016 Jun 28;7(26):39279-39292.
29. Wolf J, Dewi DL, Fredebohm J, et al. A mammosphere formation RNAi screen reveals that ATG4A promotes a breast cancer stem-like phenotype. Breast Cancer Res. 2013 Nov 14;15(6):R109.
30. Yang Z, Wilkie-Grantham RP, Yanagi T, et al. ATG4B (Autophagin-1) phosphorylation modulates autophagy. J Biol Chem. 2015 Oct 30;290(44):26549-61.
31. Cabrera S, Maciel M, Herrera I, et al. Essential role for the ATG4B protease and autophagy in bleomycin-induced pulmonary fibrosis. Autophagy. 2015 Apr 3;11(4):670-84.
32. Akin D, Wang SK, Habibzadegah-Tari P, et al. A novel ATG4B antagonist inhibits autophagy and has a negative impact on osteosarcoma tumors. Autophagy. 2014;10(11):2021-35.
33. Marino G, Salvador-Montoliu N, Fueyo A, et al. Tissue-specific autophagy alterations and increased tumorigenesis in mice deficient in Atg4C/autophagin-3. J Biol Chem. 2007 Jun 22;282(25):18573-83.

34. Ndoye A, Budina-Kolomets A, Kugel CH, 3rd, et al. ATG5 Mediates a Positive Feedback Loop between Wnt Signaling and Autophagy in Melanoma. Cancer Res. 2017 Nov 1;77(21):5873-5885.
35. Pyo JO, Yoo SM, Ahn HH, et al. Overexpression of Atg5 in mice activates autophagy and extends lifespan. Nat Commun. 2013;4:2300.
36. Peng X, Wang Y, Li H, et al. ATG5-mediated autophagy suppresses NF-kappaB signaling to limit epithelial inflammatory response to kidney injury. Cell Death Dis. 2019 Mar 15;10(4):253.
37. Cassidy LD, Young AR, Perez-Mancera PA, et al. A novel Atg5-shRNA mouse model enables temporal control of Autophagy in vivo. Autophagy. 2018;14(7):1256-1266.
38. Su LY, Luo R, Liu Q, et al. Atg5- and Atg7-dependent autophagy in dopaminergic neurons regulates cellular and behavioral responses to morphine. Autophagy. 2017 Sep 2;13(9):1496-1511.
39. Arakawa S, Tsujioka M, Yoshida T, et al. Role of Atg5-dependent cell death in the embryonic development of Bax/Bak double-knockout mice. Cell Death Differ. 2017 Sep;24(9):1598-1608.
40. Zhang Y, Cross SD, Stanton JB, et al. Early AMD-like defects in the RPE and retinal degeneration in aged mice with RPE-specific deletion of Atg5 or Atg7. Mol Vis. 2017;23:228-241.
41. Imagawa Y, Saitoh T, Tsujimoto Y. Vital staining for cell death identifies Atg9a-dependent necrosis in developmental bone formation in mouse. Nat Commun. 2016 Nov 4;7:13391.
42. Lee JC, Choe SY, Cha CI. Region-specific changes in the immunoreactivity of Atg9A in the central nervous system of SOD1(G93A) transgenic mice. Anat Cell Biol. 2014 Jun;47(2):101-10.
43. Komatsu M, Waguri S, Ueno T, et al. Impairment of starvation-induced and constitutive autophagy in Atg7-deficient mice. J Cell Biol. 2005 May 9;169(3):425-34.
44. Kim WY, Nam SA, Choi A, et al. Atg7-dependent canonical autophagy regulates the degradation of aquaporin 2 in prolonged hypokalemia. Sci Rep. 2019 Feb 28;9(1):3021.
45. Sukseree S, Laszlo L, Gruber F, et al. Filamentous Aggregation of Sequestosome-1/p62 in Brain Neurons and Neuroepithelial Cells upon Tyr-Cre-Mediated Deletion of the Autophagy Gene Atg7. Mol Neurobiol. 2018 Nov;55(11):8425-8437.
46. Jiang CY, Yang BY, Zhao S, et al. Deregulation of ATG9A by impaired AR signaling induces autophagy in prostate stromal fibroblasts and promotes BPH progression. Cell Death Dis. 2018 Apr 1;9(4):431.
47. De Pace R, Skirzewski M, Damme M, et al. Altered distribution of ATG9A and accumulation of axonal aggregates in neurons from a mouse model of AP-4 deficiency syndrome. PLoS Genet. 2018 Apr;14(4):e1007363.
48. Pang J, Xiong H, Lin P, et al. Activation of miR-34a impairs autophagic flux and promotes cochlear cell death via repressing ATG9A: implications for age-related hearing loss. Cell Death Dis. 2017 Oct 5;8(10):e3079.
49. Abdul Rahim SA, Dirkse A, Oudin A, et al. Regulation of hypoxia-induced autophagy in glioblastoma involves ATG9A. Br J Cancer. 2017 Sep 5;117(6):813-825.
50. Yamaguchi J, Suzuki C, Nanao T, et al. Atg9a deficiency causes axon-specific lesions including neuronal circuit dysgenesis. Autophagy. 2018;14(5):764-777.

51. Tamura H, Shibata M, Koike M, et al. Atg9A protein, an autophagy-related membrane protein, is localized in the neurons of mouse brains. J Histochem Cytochem. 2010 May;58(5):443-53.
52. Chen JF, Wu P, Xia R, et al. STAT3-induced lncRNA HAGLROS overexpression contributes to the malignant progression of gastric cancer cells via mTOR signal-mediated inhibition of autophagy. Mol Cancer. 2018 Jan 12;17(1):6.
53. Wang N, Tan HY, Li S, et al. Atg9b Deficiency Suppresses Autophagy and Potentiates Endoplasmic Reticulum Stress-Associated Hepatocyte Apoptosis in Hepatocarcinogenesis. Theranostics. 2017;7(8):2325-2338.
54. Nassif M, Valenzuela V, Rojas-Rivera D, et al. Pathogenic role of BECN1/Beclin 1 in the development of amyotrophic lateral sclerosis. Autophagy. 2014 Jul;10(7):1256-71.
55. Pedro JM, Wei Y, Sica V, et al. BAX and BAK1 are dispensable for ABT-737-induced dissociation of the BCL2-BECN1 complex and autophagy. Autophagy. 2015;11(3):452-9.
56. Zhao SJ, Kong FQ, Cai W, et al. GIT1 contributes to autophagy in osteoclast through disruption of the binding of Beclin1 and Bcl2 under starvation condition. Cell Death Dis. 2018 Dec 13;9(12):1195.
57. Upadhyay R, Sanchez-Hidalgo A, Wilusz CJ, et al. Host Directed Therapy for Chronic Tuberculosis via Intrapulmonary Delivery of Aerosolized Peptide Inhibitors Targeting the IL-10-STAT3 Pathway. Sci Rep. 2018 Nov 9;8(1):16610.
58. Liu J, Liu W, Lu Y, et al. Piperlongumine restores the balance of autophagy and apoptosis by increasing BCL2 phosphorylation in rotenone-induced Parkinson disease models. Autophagy. 2018;14(5):845-861.
59. Piras A, Schiaffino L, Boido M, et al. Inhibition of autophagy delays motoneuron degeneration and extends lifespan in a mouse model of spinal muscular atrophy. Cell Death Dis. 2017 Dec 20;8(12):3223.
60. Liu GS, Zhu H, Cai WF, et al. Regulation of BECN1-mediated autophagy by HSPB6: Insights from a human HSPB6(S10F) mutant. Autophagy. 2018;14(1):80-97.
61. Rocchi A, Yamamoto S, Ting T, et al. A Becn1 mutation mediates hyperactive autophagic sequestration of amyloid oligomers and improved cognition in Alzheimer's disease. PLoS Genet. 2017 Aug;13(8):e1006962.
62. Peng Y, Miao H, Wu S, et al. ABHD5 interacts with BECN1 to regulate autophagy and tumorigenesis of colon cancer independent of PNPLA2. Autophagy. 2016 Nov;12(11):2167-2182.
63. Cicchini M, Chakrabarti R, Kongara S, et al. Autophagy regulator BECN1 suppresses mammary tumorigenesis driven by WNT1 activation and following parity. Autophagy. 2014;10(11):2036-52.
64. Liu J, Wang H, Gu J, et al. BECN1-dependent CASP2 incomplete autophagy induction by binding to rabies virus phosphoprotein. Autophagy. 2017 Apr 3;13(4):739-753.
65. Wang X, Tao Y, Huang Y, et al. Catalase ameliorates diabetes-induced cardiac injury through reduced p65/RelA- mediated transcription of BECN1. J Cell Mol Med. 2017 Dec;21(12):3420-3434.
66. Chen C, Yang S, Li H, et al. Mir30c Is Involved in Diabetic Cardiomyopathy through Regulation of Cardiac Autophagy via BECN1. Mol Ther Nucleic Acids. 2017 Jun 16;7:127-139.

67. Bae SY, Byun S, Bae SH, et al. TPT1 (tumor protein, translationally-controlled 1) negatively regulates autophagy through the BECN1 interactome and an MTORC1-mediated pathway. Autophagy. 2017 May 4;13(5):820-833.
68. Wei ZB, Yuan YF, Jaouen F, et al. SLC35D3 increases autophagic activity in midbrain dopaminergic neurons by enhancing BECN1-ATG14-PIK3C3 complex formation. Autophagy. 2016 Jul 2;12(7):1168-79.
69. Kuramoto K, Wang N, Fan Y, et al. Autophagy activation by novel inducers prevents BECN2-mediated drug tolerance to cannabinoids. Autophagy. 2016 Sep;12(9):1460-71.
70. Zhang W, He C. Regulation of plasma membrane receptors by a new autophagy-related BECN/Beclin family member. Autophagy. 2014 Aug;10(8):1472-3.
71. Sung YH, Jin Y, Kang Y, et al. Ei24, a novel E2F target gene, affects p53-independent cell death upon ultraviolet C irradiation. J Biol Chem. 2013 Oct 25;288(43):31261-7.
72. Zhao YG, Zhao H, Miao L, et al. The p53-induced gene Ei24 is an essential component of the basal autophagy pathway. J Biol Chem. 2012 Dec 7;287(50):42053-63.
73. Salah FS, Ebbinghaus M, Muley VY, et al. Tumor suppression in mice lacking GABARAP, an Atg8/LC3 family member implicated in autophagy, is associated with alterations in cytokine secretion and cell death. Cell Death Dis. 2016 Apr 28;7:e2205.
74. Dunlop EA, Seifan S, Claessens T, et al. FLCN, a novel autophagy component, interacts with GABARAP and is regulated by ULK1 phosphorylation. Autophagy. 2014 Oct 1;10(10):1749-60.
75. Nath S, Dancourt J, Shteyn V, et al. Lipidation of the LC3/GABARAP family of autophagy proteins relies on a membrane-curvature-sensing domain in Atg3. Nat Cell Biol. 2014 May;16(5):415-24.
76. Le Grand JN, Bon K, Fraichard A, et al. Specific distribution of the autophagic protein GABARAPL1/GEC1 in the developing and adult mouse brain and identification of neuronal populations expressing GABARAPL1/GEC1. PLoS One. 2013;8(5):e63133.
77. Pascall JC, Rotondo S, Mukadam AS, et al. The immune system GTPase GIMAP6 interacts with the Atg8 homologue GABARAPL2 and is recruited to autophagosomes. PLoS One. 2013;8(10):e77782.
78. Hui KK, Takashima N, Watanabe A, et al. GABARAPs dysfunction by autophagy deficiency in adolescent brain impairs GABAA receptor trafficking and social behavior. Sci Adv. 2019 Apr;5(4):eaau8237.
79. Zhang P, Verity MA, Reue K. Lipin-1 regulates autophagy clearance and intersects with statin drug effects in skeletal muscle. Cell Metab. 2014 Aug 5;20(2):267-79.
80. Heras-Sandoval D, Perez-Rojas JM, Hernandez-Damian J, et al. The role of PI3K/AKT/mTOR pathway in the modulation of autophagy and the clearance of protein aggregates in neurodegeneration. Cell Signal. 2014 Dec;26(12):2694-701.
81. Xu J, Kozlov G, McPherson PS, et al. A PH-like domain of the Rab12 guanine nucleotide exchange factor DENND3 binds actin and is required for autophagy. J Biol Chem. 2018 Mar 23;293(12):4566-4574.
82. Wang B, Iyengar R, Li-Harms X, et al. The autophagy-inducing kinases, ULK1 and ULK2, regulate axon guidance in the developing mouse forebrain via a noncanonical pathway. Autophagy. 2018;14(5):796-811.
83. Touyama K, Khan M, Aoki K, et al. Bif-1/Endophilin B1/SH3GLB1 regulates bone

homeostasis. J Cell Biochem. 2019 Nov;120(11):18793-18804.
84. Zachari M, Ganley IG. The mammalian ULK1 complex and autophagy initiation. Essays Biochem. 2017 Dec 12;61(6):585-596.
85. Afzal S, Hao Z, Itsumi M, et al. Autophagy-independent functions of UVRAG are essential for peripheral naive T-cell homeostasis. Proc Natl Acad Sci U S A. 2015 Jan 27;112(4):1119-24.
86. Bakula D, Muller AJ, Zuleger T, et al. WIPI3 and WIPI4 beta-propellers are scaffolds for LKB1-AMPK-TSC signalling circuits in the control of autophagy. Nat Commun. 2017 May 31;8:15637.
87. Bansal M, Moharir SC, Sailasree SP, et al. Optineurin promotes autophagosome formation by recruiting the autophagy-related Atg12-5-16L1 complex to phagophores containing the Wipi2 protein. J Biol Chem. 2018 Jan 5;293(1):132-147.
88. Tamura N, Nishimura T, Sakamaki Y, et al. Differential requirement for ATG2A domains for localization to autophagic membranes and lipid droplets. FEBS Lett. 2017 Dec;591(23):3819-3830.
89. Zhou J, Wu S, Chen Y, et al. microRNA-143 is associated with the survival of ALDH1+CD133+ osteosarcoma cells and the chemoresistance of osteosarcoma. Exp Biol Med (Maywood). 2015 Jul;240(7):867-75.
90. Wang H, Wan H, Li X, et al. Atg7 is required for acrosome biogenesis during spermatogenesis in mice. Cell Res. 2014 Jul;24(7):852-69.
91. Haddad DM, Vilain S, Vos M, et al. Mutations in the intellectual disability gene Ube2a cause neuronal dysfunction and impair parkin-dependent mitophagy. Mol Cell. 2013 Jun 27;50(6):831-43.
92. Koyama-Honda I, Itakura E, Fujiwara TK, et al. Temporal analysis of recruitment of mammalian ATG proteins to the autophagosome formation site. Autophagy. 2013 Oct;9(10):1491-9.
93. Kaur G, Tan LX, Rathnasamy G, et al. Aberrant early endosome biogenesis mediates complement activation in the retinal pigment epithelium in models of macular degeneration. Proc Natl Acad Sci U S A. 2018 Sep 4;115(36):9014-9019.
94. Hebron ML, Lonskaya I, Moussa CE. Tyrosine kinase inhibition facilitates autophagic SNCA/alpha-synuclein clearance. Autophagy. 2013 Aug;9(8):1249-50.
95. Li X, Yu W, Qian X, et al. Nucleus-Translocated ACSS2 Promotes Gene Transcription for Lysosomal Biogenesis and Autophagy. Mol Cell. 2017 Jun 1;66(5):684-697.e9.
96. Magga J, Vainio L, Kilpio T, et al. Systemic Blockade of ACVR2B Ligands Protects Myocardium from Acute Ischemia-Reperfusion Injury. Mol Ther. 2019 Mar 6;27(3):600-610.
97. Hentila J, Nissinen TA, Korkmaz A, et al. Activin Receptor Ligand Blocking and Cancer Have Distinct Effects on Protein and Redox Homeostasis in Skeletal Muscle and Liver. Front Physiol. 2018;9:1917.
98. Du WW, Yang W, Fang L, et al. miR-17 extends mouse lifespan by inhibiting senescence signaling mediated by MKP7. Cell Death Dis. 2014 Jul 31;5:e1355.
99. Chung SJ, Nagaraju GP, Nagalingam A, et al. ADIPOQ/adiponectin induces cytotoxic autophagy in breast cancer cells through STK11/LKB1-mediated activation of the AMPK-ULK1 axis. Autophagy. 2017 Aug 3;13(8):1386-1403.

100. Kim SN, Kwon HJ, Im SW, et al. Connexin 43 is required for the maintenance of mitochondrial integrity in brown adipose tissue. Sci Rep. 2017 Aug 2;7(1):7159.
101. Xu A, Sweeney G. Emerging role of autophagy in mediating widespread actions of ADIPOQ/adiponectin. Autophagy. 2015 Apr 3;11(4):723-4.
102. Chou IP, Chiu YP, Ding ST, et al. Adiponectin receptor 1 overexpression reduces lipid accumulation and hypertrophy in the heart of diet-induced obese mice--possible involvement of oxidative stress and autophagy. Endocr Res. 2014;39(4):173-9.
103. Kang R, Tang D, Lotze MT, et al. AGER/RAGE-mediated autophagy promotes pancreatic tumorigenesis and bioenergetics through the IL6-pSTAT3 pathway. Autophagy. 2012 Jun;8(6):989-91.
104. Chakrabarti L, Eng J, Ivanov N, et al. Autophagy activation and enhanced mitophagy characterize the Purkinje cells of pcd mice prior to neuronal death. Mol Brain. 2009 Jul 29;2:24.
105. Berezniuk I, Sironi J, Callaway MB, et al. CCP1/Nna1 functions in protein turnover in mouse brain: Implications for cell death in Purkinje cell degeneration mice. Faseb j. 2010 Jun;24(6):1813-23.
106. Tong J, Lai Y, Yao YA, et al. Qiliqiangxin Rescues Mouse Cardiac Function by Regulating AGTR1/TRPV1-Mediated Autophagy in STZ-Induced Diabetes Mellitus. Cell Physiol Biochem. 2018;47(4):1365-1376.
107. Porrello ER, D'Amore A, Curl CL, et al. Angiotensin II type 2 receptor antagonizes angiotensin II type 1 receptor-mediated cardiomyocyte autophagy. Hypertension. 2009 Jun;53(6):1032-40.
108. Shi CS, Shenderov K, Huang NN, et al. Activation of autophagy by inflammatory signals limits IL-1beta production by targeting ubiquitinated inflammasomes for destruction. Nat Immunol. 2012 Jan 29;13(3):255-63.
109. Sun Q, Loughran P, Shapiro R, et al. Redox-dependent regulation of hepatocyte absent in melanoma 2 inflammasome activation in sterile liver injury in mice. Hepatology. 2017 Jan;65(1):253-268.
110. Kurdi A, De Doncker M, Leloup A, et al. Continuous administration of the mTORC1 inhibitor everolimus induces tolerance and decreases autophagy in mice. Br J Pharmacol. 2016 Dec;173(23):3359-3371.
111. Furlong HC, Stampfli MR, Gannon AM, et al. Cigarette smoke exposure triggers the autophagic cascade via activation of the AMPK pathway in mice. Biol Reprod. 2015 Oct;93(4):93.
112. Li R, Tan S, Yu M, et al. Annexin A2 Regulates Autophagy in Pseudomonas aeruginosa Infection through the Akt1-mTOR-ULK1/2 Signaling Pathway. J Immunol. 2015 Oct 15;195(8):3901-11.
113. Sutton MN, Yang H, Huang GY, et al. RAS-related GTPases DIRAS1 and DIRAS2 induce autophagic cancer cell death and are required for autophagy in murine ovarian cancer cells. Autophagy. 2018;14(4):637-653.
114. Wang Q, Ren J. mTOR-Independent autophagy inducer trehalose rescues against insulin resistance-induced myocardial contractile anomalies: Role of p38 MAPK and Foxo1. Pharmacol Res. 2016 Sep;111:357-373.
115. Zhang Y, Han X, Hu N, et al. Akt2 knockout alleviates prolonged caloric

restriction-induced change in cardiac contractile function through regulation of autophagy. J Mol Cell Cardiol. 2014 Jun;71:81-91.
116. Corum DG, Tsichlis PN, Muise-Helmericks RC. AKT3 controls mitochondrial biogenesis and autophagy via regulation of the major nuclear export protein CRM-1. Faseb j. 2014 Jan;28(1):395-407.
117. Shah M, Edman MC, Reddy Janga S, et al. Rapamycin Eye Drops Suppress Lacrimal Gland Inflammation In a Murine Model of Sjogren's Syndrome. Invest Ophthalmol Vis Sci. 2017 Jan 1;58(1):372-385.
118. Wani A, Gupta M, Ahmad M, et al. Alborixin clears amyloid-beta by inducing autophagy through PTEN-mediated inhibition of the AKT pathway. Autophagy. 2019 Oct;15(10):1810-1828.
119. Zhong S, Li L, Zhang YL, et al. Acetaldehyde dehydrogenase 2 interactions with LDLR and AMPK regulate foam cell formation. J Clin Invest. 2019 Jan 2;129(1):252-267.
120. Li M, Xu M, Li J, et al. Alda-1 Ameliorates Liver Ischemia-Reperfusion Injury by Activating Aldehyde Dehydrogenase 2 and Enhancing Autophagy in Mice. J Immunol Res. 2018;2018:9807139.
121. Chen L, Lang AL, Poff GD, et al. Vinyl chloride-induced interaction of nonalcoholic and toxicant-associated steatohepatitis: Protection by the ALDH2 activator Alda-1. Redox Biol. 2019 Jun;24:101205.
122. Tanaka K, Whelan KA, Chandramouleeswaran PM, et al. ALDH2 modulates autophagy flux to regulate acetaldehyde-mediated toxicity thresholds. Am J Cancer Res. 2016;6(4):781-96.
123. Wu B, Yu L, Wang Y, et al. Aldehyde dehydrogenase 2 activation in aged heart improves the autophagy by reducing the carbonyl modification on SIRT1. Oncotarget. 2016 Jan 19;7(3):2175-88.
124. Vogel KR, Ainslie GR, Jansen EE, et al. Torin 1 partially corrects vigabatrin-induced mitochondrial increase in mouse. Ann Clin Transl Neurol. 2015 Jun;2(6):699-706.
125. Morgan AH, Hammond VJ, Sakoh-Nakatogawa M, et al. A novel role for 12/15-lipoxygenase in regulating autophagy. Redox Biol. 2015;4:40-7.
126. Di Meco A, Li JG, Blass BE, et al. 12/15-Lipoxygenase Inhibition Reverses Cognitive Impairment, Brain Amyloidosis, and Tau Pathology by Stimulating Autophagy in Aged Triple Transgenic Mice. Biol Psychiatry. 2017 Jan 15;81(2):92-100.
127. Hadano S, Otomo A, Kunita R, et al. Loss of ALS2/Alsin exacerbates motor dysfunction in a SOD1-expressing mouse ALS model by disturbing endolysosomal trafficking. PLoS One. 2010 Mar 22;5(3):e9805.
128. Hadano S, Mitsui S, Pan L, et al. Functional links between SQSTM1 and ALS2 in the pathogenesis of ALS: cumulative impact on the protection against mutant SOD1-mediated motor dysfunction in mice. Hum Mol Genet. 2016 Aug 1;25(15):3321-3340.
129. Zhu JF, Huang W, Yi HM, et al. Annexin A1-suppressed autophagy promotes nasopharyngeal carcinoma cell invasion and metastasis by PI3K/AKT signaling activation. Cell Death Dis. 2018 Nov 20;9(12):1154.
130. Liu L, An D, Xu J, et al. Ac2-26 Induces IKKbeta Degradation Through Chaperone-Mediated Autophagy Via HSPB1 in NCM-Treated Microglia. Front Mol

Neurosci. 2018;11:76.
131. Liu Y, Zhang J, Wang Y, et al. Apelin involved in progression of diabetic nephropathy by inhibiting autophagy in podocytes. Cell Death Dis. 2017 Aug 24;8(8):e3006.
132. Liang D, Han D, Fan W, et al. Therapeutic efficacy of apelin on transplanted mesenchymal stem cells in hindlimb ischemic mice via regulation of autophagy. Sci Rep. 2016 Feb 23;6:21914.
133. Zhang H, Gong Y, Wang Z, et al. Apelin inhibits the proliferation and migration of rat PASMCs via the activation of PI3K/Akt/mTOR signal and the inhibition of autophagy under hypoxia. J Cell Mol Med. 2014 Mar;18(3):542-53.
134. Goiran T, Duplan E, Chami M, et al. beta-Amyloid Precursor Protein Intracellular Domain Controls Mitochondrial Function by Modulating Phosphatase and Tensin Homolog-Induced Kinase 1 Transcription in Cells and in Alzheimer Mice Models. Biol Psychiatry. 2018 Mar 1;83(5):416-427.
135. Cortes CJ, Miranda HC, Frankowski H, et al. Polyglutamine-expanded androgen receptor interferes with TFEB to elicit autophagy defects in SBMA. Nat Neurosci. 2014 Sep;17(9):1180-9.
136. Nguyen HG, Yang JC, Kung HJ, et al. Targeting autophagy overcomes Enzalutamide resistance in castration-resistant prostate cancer cells and improves therapeutic response in a xenograft model. Oncogene. 2014 Sep 4;33(36):4521-30.
137. Rusmini P, Crippa V, Giorgetti E, et al. Clearance of the mutant androgen receptor in motoneuronal models of spinal and bulbar muscular atrophy. Neurobiol Aging. 2013 Nov;34(11):2585-603.
138. Bo L, Su-Ling D, Fang L, et al. Autophagic program is regulated by miR-325. Cell Death Differ. 2014 Jun;21(6):967-77.
139. Yang JS, Hsu JW, Park SY, et al. GAPDH inhibits intracellular pathways during starvation for cellular energy homeostasis. Nature. 2018 Sep;561(7722):263-267.
140. Xiong Y, Yepuri G, Forbiteh M, et al. ARG2 impairs endothelial autophagy through regulation of MTOR and PRKAA/AMPK signaling in advanced atherosclerosis. Autophagy. 2014;10(12):2223-38.
141. Liu J, Li QX, Wang XJ, et al. beta-Arrestins promote podocyte injury by inhibition of autophagy in diabetic nephropathy. Cell Death Dis. 2016 Apr 7;7:e2183.
142. Wang P, Xu TY, Wei K, et al. ARRB1/beta-arrestin-1 mediates neuroprotection through coordination of BECN1-dependent autophagy in cerebral ischemia. Autophagy. 2014 Sep;10(9):1535-48.
143. Huang HL, Hsu HP, Shieh SC, et al. Attenuation of argininosuccinate lyase inhibits cancer growth via cyclin A2 and nitric oxide. Mol Cancer Ther. 2013 Nov;12(11):2505-16.
144. Sood V, Sharma KB, Gupta V, et al. ATF3 negatively regulates cellular antiviral signaling and autophagy in the absence of type I interferons. Sci Rep. 2017 Aug 18;7(1):8789.
145. Aguirre A, Lopez-Alonso I, Gonzalez-Lopez A, et al. Defective autophagy impairs ATF3 activity and worsens lung injury during endotoxemia. J Mol Med (Berl). 2014 Jun;92(6):665-76.
146. Xiao Y, Deng Y, Yuan F, et al. An ATF4-ATG5 signaling in hypothalamic POMC neurons regulates obesity. Autophagy. 2017 Jun 3;13(6):1088-1089.

147. Luo B, Lin Y, Jiang S, et al. Endoplasmic reticulum stress eIF2alpha-ATF4 pathway-mediated cyclooxygenase-2 induction regulates cadmium-induced autophagy in kidney. Cell Death Dis. 2016 Jun 2;7(6):e2251.
148. Dey S, Sayers CM, Verginadis, II, et al. ATF4-dependent induction of heme oxygenase 1 prevents anoikis and promotes metastasis. J Clin Invest. 2015 Jul 1;125(7):2592-608.
149. Wang S, Chen XA, Hu J, et al. ATF4 Gene Network Mediates Cellular Response to the Anticancer PAD Inhibitor YW3-56 in Triple-Negative Breast Cancer Cells. Mol Cancer Ther. 2015 Apr;14(4):877-88.
150. Sheng Z, Ma L, Sun JE, et al. BCR-ABL suppresses autophagy through ATF5-mediated regulation of mTOR transcription. Blood. 2011 Sep 8;118(10):2840-8.
151. Yu Z, Sheng H, Liu S, et al. Activation of the ATF6 branch of the unfolded protein response in neurons improves stroke outcome. J Cereb Blood Flow Metab. 2017 Mar;37(3):1069-1079.
152. Gade P, Manjegowda SB, Nallar SC, et al. Regulation of the death-associated protein kinase 1 expression and autophagy via ATF6 requires apoptosis signal-regulating kinase 1. Mol Cell Biol. 2014 Nov;34(21):4033-48.
153. Kalvakolanu DV, Gade P. IFNG and autophagy: a critical role for the ER-stress mediator ATF6 in controlling bacterial infections. Autophagy. 2012 Nov;8(11):1673-4.
154. Kaizuka T, Mizushima N. Atg13 Is Essential for Autophagy and Cardiac Development in Mice. Mol Cell Biol. 2016 Feb 15;36(4):585-95.
155. Chen S, Wang C, Yeo S, et al. Distinct roles of autophagy-dependent and -independent functions of FIP200 revealed by generation and analysis of a mutant knock-in mouse model. Genes Dev. 2016 Apr 1;30(7):856-69.
156. Luo Z, Xu W, Ma S, et al. Moderate Autophagy Inhibits Vascular Smooth Muscle Cell Senescence to Stabilize Progressed Atherosclerotic Plaque via the mTORC1/ULK1/ATG13 Signal Pathway. Oxid Med Cell Longev. 2017;2017:3018190.
157. Peng N, Meng N, Wang S, et al. An activator of mTOR inhibits oxLDL-induced autophagy and apoptosis in vascular endothelial cells and restricts atherosclerosis in apolipoprotein E(-)/(-) mice. Sci Rep. 2014 Jul 1;4:5519.
158. Zhang J, Tripathi DN, Jing J, et al. ATM functions at the peroxisome to induce pexophagy in response to ROS. Nat Cell Biol. 2015 Oct;17(10):1259-1269.
159. Fang EF, Bohr VA. NAD(+): The convergence of DNA repair and mitophagy. Autophagy. 2017 Feb;13(2):442-443.
160. Kanner S, Goldin M, Galron R, et al. Astrocytes restore connectivity and synchronization in dysfunctional cerebellar networks. Proc Natl Acad Sci U S A. 2018 Jul 31;115(31):8025-8030.
161. Figueiredo N, Chora A, Raquel H, et al. Anthracyclines induce DNA damage response-mediated protection against severe sepsis. Immunity. 2013 Nov 14;39(5):874-84.
162. Valentin-Vega YA, Kastan MB. A new role for ATM: regulating mitochondrial function and mitophagy. Autophagy. 2012 May 1;8(5):840-1.
163. Alexander A, Cai SL, Kim J, et al. ATM signals to TSC2 in the cytoplasm to regulate mTORC1 in response to ROS. Proc Natl Acad Sci U S A. 2010 Mar 2;107(9):4153-8.
164. Ramonet D, Podhajska A, Stafa K, et al. PARK9-associated ATP13A2 localizes to

intracellular acidic vesicles and regulates cation homeostasis and neuronal integrity. Hum Mol Genet. 2012 Apr 15;21(8):1725-43.

165. Gusdon AM, Zhu J, Van Houten B, et al. ATP13A2 regulates mitochondrial bioenergetics through macroautophagy. Neurobiol Dis. 2012 Mar;45(3):962-72.

166. Yin JJ, Li YB, Wang Y, et al. The role of autophagy in endoplasmic reticulum stress-induced pancreatic beta cell death. Autophagy. 2012 Feb 1;8(2):158-64.

167. Rujano MA, Cannata Serio M, Panasyuk G, et al. Mutations in the X-linked ATP6AP2 cause a glycosylation disorder with autophagic defects. J Exp Med. 2017 Dec 4;214(12):3707-3729.

168. Dubos A, Castells-Nobau A, Meziane H, et al. Conditional depletion of intellectual disability and Parkinsonism candidate gene ATP6AP2 in fly and mouse induces cognitive impairment and neurodegeneration. Hum Mol Genet. 2015 Dec 1;24(23):6736-55.

169. Tripathi M, Zhang CW, Singh BK, et al. Hyperhomocysteinemia causes ER stress and impaired autophagy that is reversed by Vitamin B supplementation. Cell Death Dis. 2016 Dec 8;7(12):e2513.

170. Mookerjee S, Papanikolaou T, Guyenet SJ, et al. Posttranslational modification of ataxin-7 at lysine 257 prevents autophagy-mediated turnover of an N-terminal caspase-7 cleavage fragment. J Neurosci. 2009 Dec 2;29(48):15134-44.

171. Kim H, Vick P, Hedtke J, et al. Wnt Signaling Translocates Lys48-Linked Polyubiquitinated Proteins to the Lysosomal Pathway. Cell Rep. 2015 May 26;11(8):1151-9.

172. Maiuri MC, Le Toumelin G, Criollo A, et al. Functional and physical interaction between Bcl-X(L) and a BH3-like domain in Beclin-1. Embo j. 2007 May 16;26(10):2527-39.

173. Liu X, Yamashita T, Shang J, et al. Molecular switching from ubiquitin-proteasome to autophagy pathways in mice stroke model. J Cereb Blood Flow Metab. 2018 Oct 30:271678x18810617.

174. Sebastiani A, Golz C, Sebastiani PG, et al. Sequestosome 1 Deficiency Delays, but Does Not Prevent Brain Damage Formation Following Acute Brain Injury in Adult Mice. Front Neurosci. 2017;11:678.

175. Rusmini P, Cristofani R, Galbiati M, et al. The Role of the Heat Shock Protein B8 (HSPB8) in Motoneuron Diseases. Front Mol Neurosci. 2017;10:176.

176. Rusmini P, Polanco MJ, Cristofani R, et al. Aberrant Autophagic Response in The Muscle of A Knock-in Mouse Model of Spinal and Bulbar Muscular Atrophy. Sci Rep. 2015 Oct 22;5:15174.

177. Crippa V, Boncoraglio A, Galbiati M, et al. Differential autophagy power in the spinal cord and muscle of transgenic ALS mice. Front Cell Neurosci. 2013;7:234.

178. Gamerdinger M, Hajieva P, Kaya AM, et al. Protein quality control during aging involves recruitment of the macroautophagy pathway by BAG3. Embo j. 2009 Apr 8;28(7):889-901.

179. McClung JM, McCord TJ, Ryan TE, et al. BAG3 (Bcl-2-Associated Athanogene-3) Coding Variant in Mice Determines Susceptibility to Ischemic Limb Muscle Myopathy by Directing Autophagy. Circulation. 2017 Jul 18;136(3):281-296.

180. Gamerdinger M, Kaya AM, Wolfrum U, et al. BAG3 mediates chaperone-based aggresome-targeting and selective autophagy of misfolded proteins. EMBO Rep. 2011

Feb;12(2):149-56.
181. Sebti S, Prebois C, Perez-Gracia E, et al. BAG6/BAT3 modulates autophagy by affecting EP300/p300 intracellular localization. Autophagy. 2014 Jul;10(7):1341-2.
182. Vucicevic L, Misirkic-Marjanovic M, Paunovic V, et al. Autophagy inhibition uncovers the neurotoxic action of the antipsychotic drug olanzapine. Autophagy. 2014;10(12):2362-78.
183. Liu H, Cheng Y, Yang J, et al. BBC3 in macrophages promoted pulmonary fibrosis development through inducing autophagy during silicosis. Cell Death Dis. 2017 Mar 9;8(3):e2657.
184. Zhang Y, Shen K, Bai Y, et al. Mir143-BBC3 cascade reduces microglial survival via interplay between apoptosis and autophagy: Implications for methamphetamine-mediated neurotoxicity. Autophagy. 2016 Sep;12(9):1538-59.
185. Vogler M. BCL2A1: the underdog in the BCL2 family. Cell Death Differ. 2012 Jan;19(1):67-74.
186. Yuan J, Zhang Y, Sheng Y, et al. MYBL2 guides autophagy suppressor VDAC2 in the developing ovary to inhibit autophagy through a complex of VDAC2-BECN1-BCL2L1 in mammals. Autophagy. 2015;11(7):1081-98.
187. Luo S, Rubinsztein DC. BCL2L11/BIM: a novel molecular link between autophagy and apoptosis. Autophagy. 2013 Jan;9(1):104-5.
188. Stepp MA, Pal-Ghosh S, Tadvalkar G, et al. Reduced Corneal Innervation in the CD25 Null Model of Sjogren Syndrome. Int J Mol Sci. 2018 Nov 30;19(12).
189. Lee SB, Kim HT, Yang HO, et al. Anodal transcranial direct current stimulation prevents methyl-4-phenyl-1,2,3,6-tetrahydropyridine (MPTP)-induced neurotoxicity by modulating autophagy in an in vivo mouse model of Parkinson's disease. Sci Rep. 2018 Oct 11;8(1):15165.
190. Mazouffre C, Geyl S, Perraud A, et al. Dual inhibition of BDNF/TrkB and autophagy: a promising therapeutic approach for colorectal cancer. J Cell Mol Med. 2017 Oct;21(10):2610-2622.
191. Bak DH, Zhang E, Yi MH, et al. High omega3-polyunsaturated fatty acids in fat-1 mice prevent streptozotocin-induced Purkinje cell degeneration through BDNF-mediated autophagy. Sci Rep. 2015 Oct 27;5:15465.
192. Perucho J, Casarejos MJ, Gomez A, et al. Striatal infusion of glial conditioned medium diminishes huntingtin pathology in r6/1 mice. PLoS One. 2013;8(9):e73120.
193. Li H, Li D, Ma Z, et al. Defective autophagy in osteoblasts induces endoplasmic reticulum stress and causes remarkable bone loss. Autophagy. 2018;14(10):1726-1741.
194. Amir M, Zhao E, Fontana L, et al. Inhibition of hepatocyte autophagy increases tumor necrosis factor-dependent liver injury by promoting caspase-8 activation. Cell Death Differ. 2013 Jul;20(7):878-87.
195. Kim SI, Yeo SG, Gen Y, et al. Differences in autophagy-associated mRNAs in peritoneal fluid of patients with endometriosis and gynecologic cancers. Eur J Obstet Gynecol Reprod Biol X. 2019 Apr;2:100016.
196. Ikeda F. The anti-apoptotic ubiquitin conjugating enzyme BIRC6/BRUCE regulates autophagosome-lysosome fusion. Autophagy. 2018;14(7):1283-1284.
197. Zhang A, He X, Zhang L, et al. Biogenesis of lysosome-related organelles complex-1

subunit 1 (BLOS1) interacts with sorting nexin 2 and the endosomal sorting complex required for transport-I (ESCRT-I) component TSG101 to mediate the sorting of epidermal growth factor receptor into endosomal compartments. J Biol Chem. 2014 Oct 17;289(42):29180-94.
198. Contreras AU, Mebratu Y, Delgado M, et al. Deacetylation of p53 induces autophagy by suppressing Bmf expression. J Cell Biol. 2013 Apr 29;201(3):427-37.
199. Delgado M, Tesfaigzi Y. Is BMF central for anoikis and autophagy? Autophagy. 2014 Jan;10(1):168-9.
200. Cai B, Zheng Y, Yan J, et al. BMP2-mediated PTEN enhancement promotes differentiation of hair follicle stem cells by inducing autophagy. Exp Cell Res. 2019 Dec 15;385(2):111647.
201. Yasunaga M, Oumi N, Osaki M, et al. Establishment and characterization of a transgenic mouse model for in vivo imaging of Bmp4 expression in the pancreas. PLoS One. 2011;6(9):e24956.
202. Yan XT, Sun YS, Ren S, et al. Dietary alpha-Mangostin Provides Protective Effects against Acetaminophen-Induced Hepatotoxicity in Mice via Akt/mTOR-Mediated Inhibition of Autophagy and Apoptosis. Int J Mol Sci. 2018 May 1;19(5).
203. Li L, Zviti R, Ha C, et al. Forkhead box O3 (FoxO3) regulates kidney tubular autophagy following urinary tract obstruction. J Biol Chem. 2017 Aug 18;292(33):13774-13783.
204. Ren J, Yang L, Zhu L, et al. Akt2 ablation prolongs life span and improves myocardial contractile function with adaptive cardiac remodeling: role of Sirt1-mediated autophagy regulation. Aging Cell. 2017 Oct;16(5):976-987.
205. Su Z, Klein JD, Du J, et al. Chronic kidney disease induces autophagy leading to dysfunction of mitochondria in skeletal muscle. Am J Physiol Renal Physiol. 2017 Jun 1;312(6):F1128-f1140.
206. Chen K, Li JJ, Li SN, et al. 15-Deoxy-Delta(12,14)-prostaglandin J2 alleviates hepatic ischemia-reperfusion injury in mice via inducing antioxidant response and inhibiting apoptosis and autophagy. Acta Pharmacol Sin. 2017 May;38(5):672-687.
207. Southern WM, Nichenko AS, Shill DD, et al. Skeletal muscle metabolic adaptations to endurance exercise training are attainable in mice with simvastatin treatment. PLoS One. 2017;12(2):e0172551.
208. Sebastian D, Sorianello E, Segales J, et al. Mfn2 deficiency links age-related sarcopenia and impaired autophagy to activation of an adaptive mitophagy pathway. Embo j. 2016 Aug 1;35(15):1677-93.
209. Li S, Xia Y, Chen K, et al. Epigallocatechin-3-gallate attenuates apoptosis and autophagy in concanavalin A-induced hepatitis by inhibiting BNIP3. Drug Des Devel Ther. 2016;10:631-47.
210. Yuan Y, Zheng Y, Zhang X, et al. BNIP3L/NIX-mediated mitophagy protects against ischemic brain injury independent of PARK2. Autophagy. 2017 Oct 3;13(10):1754-1766.
211. Bellot G, Garcia-Medina R, Gounon P, et al. Hypoxia-induced autophagy is mediated through hypoxia-inducible factor induction of BNIP3 and BNIP3L via their BH3 domains. Mol Cell Biol. 2009 May;29(10):2570-81.
212. Ni HM, Bhakta A, Wang S, et al. Role of hypoxia inducing factor-1beta in alcohol-induced autophagy, steatosis and liver injury in mice. PLoS One.

2014;9(12):e115849.
213. Chiavarina B, Whitaker-Menezes D, Migneco G, et al. HIF1-alpha functions as a tumor promoter in cancer associated fibroblasts, and as a tumor suppressor in breast cancer cells: Autophagy drives compartment-specific oncogenesis. Cell Cycle. 2010 Sep 1;9(17):3534-51.
214. Martinez-Outschoorn UE, Trimmer C, Lin Z, et al. Autophagy in cancer associated fibroblasts promotes tumor cell survival: Role of hypoxia, HIF1 induction and NFkappaB activation in the tumor stromal microenvironment. Cell Cycle. 2010 Sep 1;9(17):3515-33.
215. Sandoval H, Thiagarajan P, Dasgupta SK, et al. Essential role for Nix in autophagic maturation of erythroid cells. Nature. 2008 Jul 10;454(7201):232-5.
216. Li S, Song Y, Quach C, et al. Transcriptional regulation of autophagy-lysosomal function in BRAF-driven melanoma progression and chemoresistance. Nat Commun. 2019 Apr 12;10(1):1693.
217. Strohecker AM, Guo JY, Karsli-Uzunbas G, et al. Autophagy sustains mitochondrial glutamine metabolism and growth of BrafV600E-driven lung tumors. Cancer Discov. 2013 Nov;3(11):1272-85.
218. Sumpter R, Jr., Sirasanagandla S, Fernandez AF, et al. Fanconi Anemia Proteins Function in Mitophagy and Immunity. Cell. 2016 May 5;165(4):867-81.
219. Whitehouse CA, Waters S, Marchbank K, et al. Neighbor of Brca1 gene (Nbr1) functions as a negative regulator of postnatal osteoblastic bone formation and p38 MAPK activity. Proc Natl Acad Sci U S A. 2010 Jul 20;107(29):12913-8.
220. Sakamaki JI, Wilkinson S, Hahn M, et al. Bromodomain Protein BRD4 Is a Transcriptional Repressor of Autophagy and Lysosomal Function. Mol Cell. 2017 May 18;66(4):517-532.e9.
221. Song N, Li P, Jiang Y, et al. C5a receptor1 inhibition alleviates influenza virus-induced acute lung injury. Int Immunopharmacol. 2018 Jun;59:12-20.
222. Pajares M, Jimenez-Moreno N, Garcia-Yague AJ, et al. Transcription factor NFE2L2/NRF2 is a regulator of macroautophagy genes. Autophagy. 2016 Oct 2;12(10):1902-1916.
223. Jo C, Gundemir S, Pritchard S, et al. Nrf2 reduces levels of phosphorylated tau protein by inducing autophagy adaptor protein NDP52. Nat Commun. 2014 Mar 25;5:3496.
224. Pfisterer SG, Mauthe M, Codogno P, et al. Ca2+/calmodulin-dependent kinase (CaMK) signaling via CaMKI and AMP-activated protein kinase contributes to the regulation of WIPI-1 at the onset of autophagy. Mol Pharmacol. 2011 Dec;80(6):1066-75.
225. Sinha RA, Singh BK, Zhou J, et al. Thyroid hormone induction of mitochondrial activity is coupled to mitophagy via ROS-AMPK-ULK1 signaling. Autophagy. 2015;11(8):1341-57.
226. Hall DP, Cost NG, Hegde S, et al. TRPM3 and miR-204 establish a regulatory circuit that controls oncogenic autophagy in clear cell renal cell carcinoma. Cancer Cell. 2014 Nov 10;26(5):738-53.
227. Saez ME, Grilo A, Moron FJ, et al. Interaction between Calpain 5, Peroxisome proliferator-activated receptor-gamma and Peroxisome proliferator-activated receptor-delta genes: a polygenic approach to obesity. Cardiovasc Diabetol. 2008 Jul 25;7:23.

228. Kim JS, Nitta T, Mohuczy D, et al. Impaired autophagy: A mechanism of mitochondrial dysfunction in anoxic rat hepatocytes. Hepatology. 2008 May;47(5):1725-36.
229. Demarchi F, Schneider C. The calpain system as a modulator of stress/damage response. Cell Cycle. 2007 Jan 15;6(2):136-8.
230. Wang D, Zhang J, Jiang W, et al. The role of NLRP3-CASP1 in inflammasome-mediated neuroinflammation and autophagy dysfunction in manganese-induced, hippocampal-dependent impairment of learning and memory ability. Autophagy. 2017 May 4;13(5):914-927.
231. Han J, Bae J, Choi CY, et al. Autophagy induced by AXL receptor tyrosine kinase alleviates acute liver injury via inhibition of NLRP3 inflammasome activation in mice. Autophagy. 2016 Dec;12(12):2326-2343.
232. Yang Q, Stevenson HL, Scott MJ, et al. Type I interferon contributes to noncanonical inflammasome activation, mediates immunopathology, and impairs protective immunity during fatal infection with lipopolysaccharide-negative ehrlichiae. Am J Pathol. 2015 Feb;185(2):446-61.
233. Tiwari M, Sharma LK, Vanegas D, et al. A nonapoptotic role for CASP2/caspase 2: modulation of autophagy. Autophagy. 2014 Jun;10(6):1054-70.
234. Man N, Tan Y, Sun XJ, et al. Caspase-3 controls AML1-ETO-driven leukemogenesis via autophagy modulation in a ULK1-dependent manner. Blood. 2017 May 18;129(20):2782-2792.
235. Wnuk A, Rzemieniec J, Lason W, et al. Benzophenone-3 Impairs Autophagy, Alters Epigenetic Status, and Disrupts Retinoid X Receptor Signaling in Apoptotic Neuronal Cells. Mol Neurobiol. 2018 Jun;55(6):5059-5074.
236. Porcu C, Sideri S, Martini M, et al. Oleuropein Induces AMPK-Dependent Autophagy in NAFLD Mice, Regardless of the Gender. Int J Mol Sci. 2018 Dec 8;19(12).
237. Krause K, Caution K, Badr A, et al. CASP4/caspase-11 promotes autophagosome formation in response to bacterial infection. Autophagy. 2018;14(11):1928-1942.
238. Wong BKY, Ehrnhoefer DE, Graham RK, et al. Partial rescue of some features of Huntington Disease in the genetic absence of caspase-6 in YAC128 mice. Neurobiol Dis. 2015 Apr;76:24-36.
239. Lassen KG, Xavier RJ. An alteration in ATG16L1 stability in Crohn disease. Autophagy. 2014 Oct 1;10(10):1858-60.
240. Guo R, Lin B, Pan JF, et al. Inhibition of caspase-9 aggravates acute liver injury through suppression of cytoprotective autophagy. Sci Rep. 2016 Sep 1;6:32447.
241. Menzies FM, Garcia-Arencibia M, Imarisio S, et al. Calpain inhibition mediates autophagy-dependent protection against polyglutamine toxicity. Cell Death Differ. 2015 Mar;22(3):433-44.
242. Cheng S, Wani WY, Hottman DA, et al. Haplodeficiency of Cathepsin D does not affect cerebral amyloidosis and autophagy in APP/PS1 transgenic mice. J Neurochem. 2017 Jul;142(2):297-304.
243. Yang M, Liu J, Shao J, et al. Cathepsin S-mediated autophagic flux in tumor-associated macrophages accelerate tumor development by promoting M2 polarization. Mol Cancer. 2014 Mar 2;13:43.
244. Pan L, Li Y, Jia L, et al. Cathepsin S deficiency results in abnormal accumulation of

autophagosomes in macrophages and enhances Ang II-induced cardiac inflammation. PLoS One. 2012;7(4):e35315.

245. Luo X, Dan W, Luo X, et al. Caveolin 1-related autophagy initiated by aldosterone-induced oxidation promotes liver sinusoidal endothelial cells defenestration. Redox Biol. 2017 Oct;13:508-521.

246. Nah J, Yoo SM, Jung S, et al. Phosphorylated CAV1 activates autophagy through an interaction with BECN1 under oxidative stress. Cell Death Dis. 2017 May 25;8(5):e2822.

247. Dias MV, Teixeira BL, Rodrigues BR, et al. PRNP/prion protein regulates the secretion of exosomes modulating CAV1/caveolin-1-suppressed autophagy. Autophagy. 2016 Nov;12(11):2113-2128.

248. Qin L, Tian Y, Yu Z, et al. Targeting PDK1 with dichloroacetophenone to inhibit acute myeloid leukemia (AML) cell growth. Oncotarget. 2016 Jan 12;7(2):1395-407.

249. Sandilands E, Serrels B, Wilkinson S, et al. Src-dependent autophagic degradation of Ret in FAK-signalling-defective cancer cells. EMBO Rep. 2012 Aug;13(8):733-40.

250. Syu JS, Baba T, Huang JY, et al. Lysosomal activity maintains glycolysis and cyclin E1 expression by mediating Ad4BP/SF-1 stability for proper steroidogenic cell growth. Sci Rep. 2017 Mar 21;7(1):240.

251. Rah SY, Lee YH, Kim UH. NAADP-mediated Ca(2+) signaling promotes autophagy and protects against LPS-induced liver injury. Faseb j. 2017 Jul;31(7):3126-3137.

252. Bao JX, Zhang QF, Wang M, et al. Implication of CD38 gene in autophagic degradation of collagen I in mouse coronary arterial myocytes. Front Biosci (Landmark Ed). 2017 Jan 1;22:558-569.

253. Zhang Y, Xu M, Xia M, et al. Defective autophagosome trafficking contributes to impaired autophagic flux in coronary arterial myocytes lacking CD38 gene. Cardiovasc Res. 2014 Apr 1;102(1):68-78.

254. Xiong J, Xia M, Xu M, et al. Autophagy maturation associated with CD38-mediated regulation of lysosome function in mouse glomerular podocytes. J Cell Mol Med. 2013 Dec;17(12):1598-607.

255. Portillo JA, Okenka G, Reed E, et al. The CD40-autophagy pathway is needed for host protection despite IFN-Gamma-dependent immunity and CD40 induces autophagy via control of P21 levels. PLoS One. 2010 Dec 31;5(12):e14472.

256. Naor D, Sionov RV, Ish-Shalom D. CD44: structure, function, and association with the malignant process. Adv Cancer Res. 1997;71:241-319.

257. Soto-Pantoja DR, Kaur S, Roberts DD. CD47 signaling pathways controlling cellular differentiation and responses to stress. Crit Rev Biochem Mol Biol. 2015;50(3):212-30.

258. Sharifi-Sanjani M, Shoushtari AH, Quiroz M, et al. Cardiac CD47 drives left ventricular heart failure through Ca2+-CaMKII-regulated induction of HDAC3. J Am Heart Assoc. 2014 Jun 10;3(3):e000670.

259. Soto-Pantoja DR, Ridnour LA, Wink DA, et al. Blockade of CD47 increases survival of mice exposed to lethal total body irradiation. Sci Rep. 2013;3:1038.

260. Soto-Pantoja DR, Miller TW, Pendrak ML, et al. CD47 deficiency confers cell and tissue radioprotection by activation of autophagy. Autophagy. 2012 Nov;8(11):1628-42.

261. Woodham EF, Paul NR, Tyrrell B, et al. Coordination by Cdc42 of Actin, Contractility, and Adhesion for Melanoblast Movement in Mouse Skin. Curr Biol. 2017 Mar

6;27(5):624-637.
262. Linares JF, Amanchy R, Greis K, et al. Phosphorylation of p62 by cdk1 controls the timely transit of cells through mitosis and tumor cell proliferation. Mol Cell Biol. 2011 Jan;31(1):105-17.
263. Su M, Wang J, Wang C, et al. MicroRNA-221 inhibits autophagy and promotes heart failure by modulating the p27/CDK2/mTOR axis. Cell Death Differ. 2015 Jun;22(6):986-99.
264. Chen YW, Chen YF, Chen YT, et al. The STIM1-Orai1 pathway of store-operated Ca2+ entry controls the checkpoint in cell cycle G1/S transition. Sci Rep. 2016 Feb 26;6:22142.
265. Su LY, Li H, Lv L, et al. Melatonin attenuates MPTP-induced neurotoxicity via preventing CDK5-mediated autophagy and SNCA/alpha-synuclein aggregation. Autophagy. 2015;11(10):1745-59.
266. Toshima T, Shirabe K, Fukuhara T, et al. Suppression of autophagy during liver regeneration impairs energy charge and hepatocyte senescence in mice. Hepatology. 2014 Jul;60(1):290-300.
267. Jia W, He MX, McLeod IX, et al. Autophagy regulates T lymphocyte proliferation through selective degradation of the cell-cycle inhibitor CDKN1B/p27Kip1. Autophagy. 2015;11(12):2335-45.
268. Nakashima A, Aoki A, Kusabiraki T, et al. Role of autophagy in oocytogenesis, embryogenesis, implantation, and pathophysiology of pre-eclampsia. J Obstet Gynaecol Res. 2017 Apr;43(4):633-643.
269. Budina-Kolomets A, Hontz RD, Pimkina J, et al. A conserved domain in exon 2 coding for the human and murine ARF tumor suppressor protein is required for autophagy induction. Autophagy. 2013 Oct;9(10):1553-65.
270. Huang G, Zhang F, Ye Q, et al. The circadian clock regulates autophagy directly through the nuclear hormone receptor Nr1d1/Rev-erbalpha and indirectly via Cebpb/(C/ebpbeta) in zebrafish. Autophagy. 2016 Aug 2;12(8):1292-309.
271. Barakat DJ, Mendonca J, Barberi T, et al. C/EBPbeta regulates sensitivity to bortezomib in prostate cancer cells by inducing REDD1 and autophagosome-lysosome fusion. Cancer Lett. 2016 May 28;375(1):152-161.
272. Yang CS, Kim JJ, Lee HM, et al. The AMPK-PPARGC1A pathway is required for antimicrobial host defense through activation of autophagy. Autophagy. 2014 May;10(5):785-802.
273. Tsai HH, Lai HY, Chen YC, et al. Metformin promotes apoptosis in hepatocellular carcinoma through the CEBPD-induced autophagy pathway. Oncotarget. 2017 Feb 21;8(8):13832-13845.
274. Fan S, Wang Y, Wang C, et al. Hepatocyte-specific deletion of LASS2 protects against diet-induced hepatic steatosis and insulin resistance. Free Radic Biol Med. 2018 May 20;120:330-341.
275. Gosejacob D, Jager PS, Vom Dorp K, et al. Ceramide Synthase 5 Is Essential to Maintain C16:0-Ceramide Pools and Contributes to the Development of Diet-induced Obesity. J Biol Chem. 2016 Mar 25;291(13):6989-7003.
276. Giampietri C, Petrungaro S, Conti S, et al. c-Flip KO fibroblasts display lipid accumulation associated with endoplasmic reticulum stress. Biochim Biophys Acta. 2015

Jul;1851(7):929-36.
277. Racanelli AC, Kikkers SA, Choi AMK, et al. Autophagy and inflammation in chronic respiratory disease. Autophagy. 2018;14(2):221-232.
278. Ferrari E, Monzani R, Villella VR, et al. Cysteamine re-establishes the clearance of Pseudomonas aeruginosa by macrophages bearing the cystic fibrosis-relevant F508del-CFTR mutation. Cell Death Dis. 2017 Jan 12;8(1):e2544.
279. Bodas M, Silverberg D, Walworth K, et al. Augmentation of S-Nitrosoglutathione Controls Cigarette Smoke-Induced Inflammatory-Oxidative Stress and Chronic Obstructive Pulmonary Disease-Emphysema Pathogenesis by Restoring Cystic Fibrosis Transmembrane Conductance Regulator Function. Antioxid Redox Signal. 2017 Sep 1;27(7):433-451.
280. Tazi MF, Dakhlallah DA, Caution K, et al. Elevated Mirc1/Mir17-92 cluster expression negatively regulates autophagy and CFTR (cystic fibrosis transmembrane conductance regulator) function in CF macrophages. Autophagy. 2016 Nov;12(11):2026-2037.
281. Xia D, Qu L, Li G, et al. MARCH2 regulates autophagy by promoting CFTR ubiquitination and degradation and PIK3CA-AKT-MTOR signaling. Autophagy. 2016 Sep;12(9):1614-30.
282. Tosco A, De Gregorio F, Esposito S, et al. A novel treatment of cystic fibrosis acting on-target: cysteamine plus epigallocatechin gallate for the autophagy-dependent rescue of class II-mutated CFTR. Cell Death Differ. 2016 Aug;23(8):1380-93.
283. De Stefano D, Villella VR, Esposito S, et al. Restoration of CFTR function in patients with cystic fibrosis carrying the F508del-CFTR mutation. Autophagy. 2014;10(11):2053-74.
284. Bodas M, Min T, Vij N. Critical role of CFTR-dependent lipid rafts in cigarette smoke-induced lung epithelial injury. Am J Physiol Lung Cell Mol Physiol. 2011 Jun;300(6):L811-20.
285. Park C, Suh Y, Cuervo AM. Regulated degradation of Chk1 by chaperone-mediated autophagy in response to DNA damage. Nat Commun. 2015 Apr 16;6:6823.
286. Zaglia T, Milan G, Ruhs A, et al. Atrogin-1 deficiency promotes cardiomyopathy and premature death via impaired autophagy. J Clin Invest. 2014 Jun;124(6):2410-24.
287. Lee JA, Gao FB. Inhibition of autophagy induction delays neuronal cell loss caused by dysfunctional ESCRT-III in frontotemporal dementia. J Neurosci. 2009 Jul 1;29(26):8506-11.
288. Sagona AP, Nezis IP, Stenmark H. Association of CHMP4B and autophagy with micronuclei: implications for cataract formation. Biomed Res Int. 2014;2014:974393.
289. Sun AG, Meng FG, Wang MG. CISD2 promotes the proliferation of glioma cells via suppressing beclin1mediated autophagy and is targeted by microRNA449a. Mol Med Rep. 2017 Dec;16(6):7939-7948.
290. Yan H, Zhang X, Hu W, et al. Histamine H3 receptors aggravate cerebral ischaemic injury by histamine-independent mechanisms. Nat Commun. 2014 Feb 25;5:3334.
291. Yan Q, Han C, Wang G, et al. Activation of AMPK/mTORC1-Mediated Autophagy by Metformin Reverses Clk1 Deficiency-Sensitized Dopaminergic Neuronal Death. Mol Pharmacol. 2017 Dec;92(6):640-652.
292. Wavre-Shapton ST, Calvi AA, Turmaine M, et al. Photoreceptor phagosome processing

defects and disturbed autophagy in retinal pigment epithelium of Cln3Deltaex1-6 mice modelling juvenile neuronal ceroid lipofuscinosis (Batten disease). Hum Mol Genet. 2015 Dec 15;24(24):7060-74.
293. Schultz ML, Tecedor L, Stein CS, et al. CLN3 deficient cells display defects in the ARF1-Cdc42 pathway and actin-dependent events. PLoS One. 2014;9(5):e96647.
294. Chang JW, Choi H, Cotman SL, et al. Lithium rescues the impaired autophagy process in CbCln3(Deltaex7/8/Deltaex7/8) cerebellar cells and reduces neuronal vulnerability to cell death via IMPase inhibition. J Neurochem. 2011 Feb;116(4):659-68.
295. Zhang F, Kumano M, Beraldi E, et al. Clusterin facilitates stress-induced lipidation of LC3 and autophagosome biogenesis to enhance cancer cell survival. Nat Commun. 2014 Dec 12;5:5775.
296. Denaes T, Lodder J, Chobert MN, et al. The Cannabinoid Receptor 2 Protects Against Alcoholic Liver Disease Via a Macrophage Autophagy-Dependent Pathway. Sci Rep. 2016 Jun 27;6:28806.
297. Cescon M, Chen P, Castagnaro S, et al. Lack of collagen VI promotes neurodegeneration by impairing autophagy and inducing apoptosis during aging. Aging (Albany NY). 2016 May;8(5):1083-101.
298. Scotton C, Bovolenta M, Schwartz E, et al. Deep RNA profiling identified CLOCK and molecular clock genes as pathophysiological signatures in collagen VI myopathy. J Cell Sci. 2016 Apr 15;129(8):1671-84.
299. Chrisam M, Pirozzi M, Castagnaro S, et al. Reactivation of autophagy by spermidine ameliorates the myopathic defects of collagen VI-null mice. Autophagy. 2015;11(12):2142-52.
300. Bernardi P, Bonaldo P. Mitochondrial dysfunction and defective autophagy in the pathogenesis of collagen VI muscular dystrophies. Cold Spring Harb Perspect Biol. 2013 May 1;5(5):a011387.
301. Grumati P, Coletto L, Schiavinato A, et al. Physical exercise stimulates autophagy in normal skeletal muscles but is detrimental for collagen VI-deficient muscles. Autophagy. 2011 Dec;7(12):1415-23.
302. Meng H, Matthaei M, Ramanan N, et al. L450W and Q455K Col8a2 knock-in mouse models of Fuchs endothelial corneal dystrophy show distinct phenotypes and evidence for altered autophagy. Invest Ophthalmol Vis Sci. 2013 Mar 28;54(3):1887-97.
303. Su H, Li J, Zhang H, et al. COP9 signalosome controls the degradation of cytosolic misfolded proteins and protects against cardiac proteotoxicity. Circ Res. 2015 Nov 6;117(11):956-66.
304. Chen L, Jia JM, Zhong W, et al. [P70S6K is involved in the inhibition of testosterone production in TM3 mouse Leydig cells overexpressing Cox7a2]. Zhonghua Nan Ke Xue. 2011 Apr;17(4):291-5.
305. Lu SZ, Guo YS, Liang PZ, et al. Suppression of astrocytic autophagy by alphaB-crystallin contributes to alpha-synuclein inclusion formation. Transl Neurodegener. 2019;8:3.
306. Pan B, Zhang H, Cui T, et al. TFEB activation protects against cardiac proteotoxicity via increasing autophagic flux. J Mol Cell Cardiol. 2017 Dec;113:51-62.
307. Gupta MK, McLendon PM, Gulick J, et al. UBC9-Mediated Sumoylation Favorably Impacts Cardiac Function in Compromised Hearts. Circ Res. 2016 Jun

10;118(12):1894-905.
308. McLendon PM, Ferguson BS, Osinska H, et al. Tubulin hyperacetylation is adaptive in cardiac proteotoxicity by promoting autophagy. Proc Natl Acad Sci U S A. 2014 Dec 2;111(48):E5178-86.
309. Bhuiyan MS, Pattison JS, Osinska H, et al. Enhanced autophagy ameliorates cardiac proteinopathy. J Clin Invest. 2013 Dec;123(12):5284-97.
310. Zheng Q, Su H, Ranek MJ, et al. Autophagy and p62 in cardiac proteinopathy. Circ Res. 2011 Jul 22;109(3):296-308.
311. Maloyan A, Sayegh J, Osinska H, et al. Manipulation of death pathways in desmin-related cardiomyopathy. Circ Res. 2010 May 14;106(9):1524-32.
312. Zou J, Chen Z, Wei X, et al. Cystatin C as a potential therapeutic mediator against Parkinson's disease via VEGF-induced angiogenesis and enhanced neuronal autophagy in neurovascular units. Cell Death Dis. 2017 Jun 1;8(6):e2854.
313. Capparelli C, Whitaker-Menezes D, Guido C, et al. CTGF drives autophagy, glycolysis and senescence in cancer-associated fibroblasts via HIF1 activation, metabolically promoting tumor growth. Cell Cycle. 2012 Jun 15;11(12):2272-84.
314. Festa BP, Chen Z, Berquez M, et al. Impaired autophagy bridges lysosomal storage disease and epithelial dysfunction in the kidney. Nat Commun. 2018 Jan 11;9(1):161.
315. Napolitano G, Johnson JL, He J, et al. Impairment of chaperone-mediated autophagy leads to selective lysosomal degradation defects in the lysosomal storage disease cystinosis. EMBO Mol Med. 2015 Feb;7(2):158-74.
316. Wu P, Yuan X, Li F, et al. Myocardial Upregulation of Cathepsin D by Ischemic Heart Disease Promotes Autophagic Flux and Protects Against Cardiac Remodeling and Heart Failure. Circ Heart Fail. 2017 Jul;10(7).
317. Sarkar C, Zhao Z, Aungst S, et al. Impaired autophagy flux is associated with neuronal cell death after traumatic brain injury. Autophagy. 2014;10(12):2208-22.
318. Valapala M, Wilson C, Hose S, et al. Lysosomal-mediated waste clearance in retinal pigment epithelial cells is regulated by CRYBA1/betaA3/A1-crystallin via V-ATPase-MTORC1 signaling. Autophagy. 2014 Mar;10(3):480-96.
319. Martinet W, Schrijvers DM, Timmermans JP, et al. Immunohistochemical analysis of macroautophagy: recommendations and limitations. Autophagy. 2013 Mar;9(3):386-402.
320. Mizunoe Y, Sudo Y, Okita N, et al. Involvement of lysosomal dysfunction in autophagosome accumulation and early pathologies in adipose tissue of obese mice. Autophagy. 2017 Apr 3;13(4):642-653.
321. Wang GQ, Tang T, Wang ZS, et al. Overexpression of Hypo-Phosphorylated IkappaBbeta at Ser313 Protects the Heart against Sepsis. PLoS One. 2016;11(8):e0160860.
322. Sun M, Ouzounian M, de Couto G, et al. Cathepsin-L ameliorates cardiac hypertrophy through activation of the autophagy-lysosomal dependent protein processing pathways. J Am Heart Assoc. 2013 Apr 22;2(2):e000191.
323. Garcia-Jaramillo M, Spooner MH, Lohr CV, et al. Lipidomic and transcriptomic analysis of western diet-induced nonalcoholic steatohepatitis (NASH) in female Ldlr -/- mice. PLoS One. 2019;14(4):e0214387.
324. Alimov A, Wang H, Liu M, et al. Expression of autophagy and UPR genes in the developing brain during ethanol-sensitive and resistant periods. Metab Brain Dis. 2013

Dec;28(4):667-76.
325. Tanaka Y, Matsuwaki T, Yamanouchi K, et al. Increased lysosomal biogenesis in activated microglia and exacerbated neuronal damage after traumatic brain injury in progranulin-deficient mice. Neuroscience. 2013 Oct 10;250:8-19.
326. Luciani A, Villella VR, Esposito S, et al. Targeting autophagy as a novel strategy for facilitating the therapeutic action of potentiators on DeltaF508 cystic fibrosis transmembrane conductance regulator. Autophagy. 2012 Nov;8(11):1657-72.
327. Zheng J, Li H, He L, et al. Preconditioning of umbilical cord-derived mesenchymal stem cells by rapamycin increases cell migration and ameliorates liver ischaemia/reperfusion injury in mice via the CXCR4/CXCL12 axis. Cell Prolif. 2019 Mar;52(2):e12546.
328. Zhao G, Wang S, Wang Z, et al. CXCR6 deficiency ameliorated myocardial ischemia/reperfusion injury by inhibiting infiltration of monocytes and IFN-gamma-dependent autophagy. Int J Cardiol. 2013 Sep 30;168(2):853-62.
329. Giovannetti E, Wang Q, Avan A, et al. Role of CYB5A in pancreatic cancer prognosis and autophagy modulation. J Natl Cancer Inst. 2014 Jan;106(1):djt346.
330. Whitehead NP. Enhanced autophagy as a potential mechanism for the improved physiological function by simvastatin in muscular dystrophy. Autophagy. 2016;12(4):705-6.
331. Lu Y, Cederbaum AI. Cytochrome P450s and Alcoholic Liver Disease. Curr Pharm Des. 2018;24(14):1502-1517.
332. Lu Y, Cederbaum AI. Autophagy Protects against CYP2E1/Chronic Ethanol-Induced Hepatotoxicity. Biomolecules. 2015 Oct 16;5(4):2659-74.
333. Wu D, Cederbaum AI. Inhibition of autophagy promotes CYP2E1-dependent toxicity in HepG2 cells via elevated oxidative stress, mitochondria dysfunction and activation of p38 and JNK MAPK. Redox Biol. 2013;1:552-65.
334. Das S, Seth RK, Kumar A, et al. Purinergic receptor X7 is a key modulator of metabolic oxidative stress-mediated autophagy and inflammation in experimental nonalcoholic steatohepatitis. Am J Physiol Gastrointest Liver Physiol. 2013 Dec;305(12):G950-63.
335. Wu D, Wang X, Zhou R, et al. Alcohol steatosis and cytotoxicity: the role of cytochrome P4502E1 and autophagy. Free Radic Biol Med. 2012 Sep 15;53(6):1346-57.
336. Yang L, Wu D, Wang X, et al. Cytochrome P4502E1, oxidative stress, JNK, and autophagy in acute alcohol-induced fatty liver. Free Radic Biol Med. 2012 Sep 1;53(5):1170-80.
337. Li RN, Liu B, Li XM, et al. DACT1 Overexpression in type I ovarian cancer inhibits malignant expansion and cis-platinum resistance by modulating canonical Wnt signalling and autophagy. Sci Rep. 2017 Aug 24;7(1):9285.
338. Kondori NR, Paul P, Robbins JP, et al. Focus on the Role of D-serine and D-amino Acid Oxidase in Amyotrophic Lateral Sclerosis/Motor Neuron Disease (ALS). Front Mol Biosci. 2018;5:8.
339. Kondori NR, Paul P, Robbins JP, et al. Characterisation of the pathogenic effects of the in vivo expression of an ALS-linked mutation in D-amino acid oxidase: Phenotype and loss of spinal cord motor neurons. PLoS One. 2017;12(12):e0188912.
340. Lopes F, Keita AV, Saxena A, et al. ER-stress mobilization of death-associated protein kinase-1-dependent xenophagy counteracts mitochondria stress-induced epithelial barrier

dysfunction. J Biol Chem. 2018 Mar 2;293(9):3073-3087.
341. Puto LA, Brognard J, Hunter T. Transcriptional Repressor DAXX Promotes Prostate Cancer Tumorigenicity via Suppression of Autophagy. J Biol Chem. 2015 Jun 19;290(25):15406-20.
342. Chi HC, Chen SL, Tsai CY, et al. Thyroid hormone suppresses hepatocarcinogenesis via DAPK2 and SQSTM1-dependent selective autophagy. Autophagy. 2016 Dec;12(12):2271-2285.
343. Xu M, Li XX, Chen Y, et al. Enhancement of dynein-mediated autophagosome trafficking and autophagy maturation by ROS in mouse coronary arterial myocytes. J Cell Mol Med. 2014 Nov;18(11):2165-75.
344. Xia P, Wang S, Huang G, et al. RNF2 is recruited by WASH to ubiquitinate AMBRA1 leading to downregulation of autophagy. Cell Res. 2014 Aug;24(8):943-58.
345. Zhu P, Xue J, Zhang ZJ, et al. Helicobacter pylori VacA induces autophagic cell death in gastric epithelial cells via the endoplasmic reticulum stress pathway. Cell Death Dis. 2017 Dec 13;8(12):3207.
346. Zecchini S, Giovarelli M, Perrotta C, et al. Autophagy controls neonatal myogenesis by regulating the GH-IGF1 system through a NFE2L2- and DDIT3-mediated mechanism. Autophagy. 2019 Jan;15(1):58-77.
347. Tang B, Li Q, Zhao XH, et al. Shiga toxins induce autophagic cell death in intestinal epithelial cells via the endoplasmic reticulum stress pathway. Autophagy. 2015;11(2):344-54.
348. Gordon BS, Liu C, Steiner JL, et al. Loss of REDD1 augments the rate of the overload-induced increase in muscle mass. Am J Physiol Regul Integr Comp Physiol. 2016 Sep 1;311(3):R545-57.
349. Cui J, Jin S, Wang RF. The BECN1-USP19 axis plays a role in the crosstalk between autophagy and antiviral immune responses. Autophagy. 2016 Jul 2;12(7):1210-1.
350. Hernandez-Tiedra S, Fabrias G, Davila D, et al. Dihydroceramide accumulation mediates cytotoxic autophagy of cancer cells via autolysosome destabilization. Autophagy. 2016 Nov;12(11):2213-2229.
351. Xu J, McPherson PS. Regulation of DENND3, the exchange factor for the small GTPase Rab12 through an intramolecular interaction. J Biol Chem. 2017 Apr 28;292(17):7274-7282.
352. Matsui T, Noguchi K, Fukuda M. Dennd3 functions as a guanine nucleotide exchange factor for small GTPase Rab12 in mouse embryonic fibroblasts. J Biol Chem. 2014 May 16;289(20):13986-95.
353. De Palma C, Morisi F, Cheli S, et al. Autophagy as a new therapeutic target in Duchenne muscular dystrophy. Cell Death Dis. 2012 Nov 15;3:e418.
354. Lan YY, Londono D, Bouley R, et al. Dnase2a deficiency uncovers lysosomal clearance of damaged nuclear DNA via autophagy. Cell Rep. 2014 Oct 9;9(1):180-192.
355. Wang Q, Wu S, Zhu H, et al. Deletion of PRKAA triggers mitochondrial fission by inhibiting the autophagy-dependent degradation of DNM1L. Autophagy. 2017 Feb;13(2):404-422.
356. Huang Q, Zhan L, Cao H, et al. Increased mitochondrial fission promotes autophagy and hepatocellular carcinoma cell survival through the ROS-modulated coordinated regulation

of the NFKB and TP53 pathways. Autophagy. 2016 Jun 2;12(6):999-1014.
357. Durieux AC, Vassilopoulos S, Laine J, et al. A centronuclear myopathy--dynamin 2 mutation impairs autophagy in mice. Traffic. 2012 Jun;13(6):869-79.
358. Yang A, Jiao Y, Yang S, et al. Homocysteine activates autophagy by inhibition of CFTR expression via interaction between DNA methylation and H3K27me3 in mouse liver. Cell Death Dis. 2018 Feb 7;9(2):169.
359. Sumis A, Cook KL, Andrade FO, et al. Social isolation induces autophagy in the mouse mammary gland: link to increased mammary cancer risk. Endocr Relat Cancer. 2016 Oct;23(10):839-56.
360. Di Fazio P, Waldegger P, Jabari S, et al. Autophagy-related cell death by pan-histone deacetylase inhibition in liver cancer. Oncotarget. 2016 May 17;7(20):28998-9010.
361. Perusek L, Sahu B, Parmar T, et al. Di-retinoid-pyridinium-ethanolamine (A2E) Accumulation and the Maintenance of the Visual Cycle Are Independent of Atg7-mediated Autophagy in the Retinal Pigmented Epithelium. J Biol Chem. 2015 Nov 27;290(48):29035-44.
362. Metge BJ, Mitra A, Chen D, et al. N-Myc and STAT Interactor regulates autophagy and chemosensitivity in breast cancer cells. Sci Rep. 2015 Jul 6;5:11995.
363. Yu M, Jiang Y, Feng Q, et al. DRAM1 protects neuroblastoma cells from oxygen-glucose deprivation/reperfusion-induced injury via autophagy. Int J Mol Sci. 2014 Oct 23;15(10):19253-64.
364. Li KC, Hua KT, Lin YS, et al. Inhibition of G9a induces DUSP4-dependent autophagic cell death in head and neck squamous cell carcinoma. Mol Cancer. 2014 Jul 15;13:172.
365. Choi JC, Wu W, Muchir A, et al. Dual specificity phosphatase 4 mediates cardiomyopathy caused by lamin A/C (LMNA) gene mutation. J Biol Chem. 2012 Nov 23;287(48):40513-24.
366. Sopariwala DH, Yadav V, Badin PM, et al. Long-term PGC1beta overexpression leads to apoptosis, autophagy and muscle wasting. Sci Rep. 2017 Aug 31;7(1):10237.
367. Wang P, Long M, Zhang S, et al. Hypoxia inducible factor-1alpha regulates autophagy via the p27-E2F1 signaling pathway. Mol Med Rep. 2017 Aug;16(2):2107-2112.
368. Chen ZH, Wang WT, Huang W, et al. The lncRNA HOTAIRM1 regulates the degradation of PML-RARA oncoprotein and myeloid cell differentiation by enhancing the autophagy pathway. Cell Death Differ. 2017 Feb;24(2):212-224.
369. Haim Y, Bluher M, Slutsky N, et al. Elevated autophagy gene expression in adipose tissue of obese humans: A potential non-cell-cycle-dependent function of E2F1. Autophagy. 2015 Nov 2;11(11):2074-2088.
370. Olivan S, Calvo AC, Gasco S, et al. Time-Point Dependent Activation of Autophagy and the UPS in SOD1G93A Mice Skeletal Muscle. PLoS One. 2015;10(8):e0134830.
371. Wang SN, Wang LT, Sun DP, et al. Intestine-specific homeobox (ISX) upregulates E2F1 expression and related oncogenic activities in HCC. Oncotarget. 2016 Jun 14;7(24):36924-36939.
372. Wang F, Cho WC, Chan LW, et al. Gene network exploration of crosstalk between apoptosis and autophagy in chronic myelogenous leukemia. Biomed Res Int. 2015;2015:459840.
373. Qiang L, Zhao B, Shah P, et al. Autophagy positively regulates DNA damage recognition

by nucleotide excision repair. Autophagy. 2016;12(2):357-68.

374. Hatchi E, Rodier G, Sardet C, et al. E4F1 dysfunction results in autophagic cell death in myeloid leukemic cells. Autophagy. 2011 Dec;7(12):1566-7.

375. Monastyrska I, Ulasli M, Rottier PJ, et al. An autophagy-independent role for LC3 in equine arteritis virus replication. Autophagy. 2013 Feb 1;9(2):164-74.

376. Schaaf MB, Houbaert D, Mece O, et al. Lysosomal Pathways and Autophagy Distinctively Control Endothelial Cell Behavior to Affect Tumor Vasculature. Front Oncol. 2019;9:171.

377. Ryu JW, Choe SS, Ryu SH, et al. Paradoxical induction of growth arrest and apoptosis by EGF via the up-regulation of PTEN by activating Redox factor-1/Egr-1 in human lung cancer cells. Oncotarget. 2017 Jan 17;8(3):4181-4195.

378. Shapiro IM, Layfield R, Lotz M, et al. Boning up on autophagy: the role of autophagy in skeletal biology. Autophagy. 2014 Jan;10(1):7-19.

379. Wu Y, Li D, Wang Y, et al. Beta-Defensin 2 and 3 Promote Bacterial Clearance of Pseudomonas aeruginosa by Inhibiting Macrophage Autophagy through Downregulation of Early Growth Response Gene-1 and c-FOS. Front Immunol. 2018;9:211.

380. He J, Yu JJ, Xu Q, et al. Downregulation of ATG14 by EGR1-MIR152 sensitizes ovarian cancer cells to cisplatin-induced apoptosis by inhibiting cyto-protective autophagy. Autophagy. 2015;11(2):373-84.

381. Artal-Martinez de Narvajas A, Gomez TS, Zhang JS, et al. Epigenetic regulation of autophagy by the methyltransferase G9a. Mol Cell Biol. 2013 Oct;33(20):3983-93.

382. Periyasamy P, Guo ML, Buch S. Cocaine induces astrocytosis through ER stress-mediated activation of autophagy. Autophagy. 2016 Aug 2;12(8):1310-29.

383. Guo ML, Liao K, Periyasamy P, et al. Cocaine-mediated microglial activation involves the ER stress-autophagy axis. Autophagy. 2015;11(7):995-1009.

384. Dey S, Tameire F, Koumenis C. PERK-ing up autophagy during MYC-induced tumorigenesis. Autophagy. 2013 Apr;9(4):612-4.

385. Bretin A, Lucas C, Larabi A, et al. AIEC infection triggers modification of gut microbiota composition in genetically predisposed mice, contributing to intestinal inflammation. Sci Rep. 2018 Aug 17;8(1):12301.

386. Bretin A, Carriere J, Dalmasso G, et al. Activation of the EIF2AK4-EIF2A/eIF2alpha-ATF4 pathway triggers autophagy response to Crohn disease-associated adherent-invasive Escherichia coli infection. Autophagy. 2016 May 3;12(5):770-83.

387. Kim C, Kim W, Lee H, et al. The RNA-binding protein HuD regulates autophagosome formation in pancreatic beta cells by promoting autophagy-related gene 5 expression. J Biol Chem. 2014 Jan 3;289(1):112-21.

388. Nordstroma U, Beauvais G, Ghosh A, et al. Progressive nigrostriatal terminal dysfunction and degeneration in the engrailed1 heterozygous mouse model of Parkinson's disease. Neurobiol Dis. 2015 Jan;73:70-82.

389. Schonenberger MJ, Krek W, Kovacs WJ. EPAS1/HIF-2alpha is a driver of mammalian pexophagy. Autophagy. 2015;11(6):967-9.

390. Lu Q, Yokoyama CC, Williams JW, et al. Homeostatic Control of Innate Lung Inflammation by Vici Syndrome Gene Epg5 and Additional Autophagy Genes Promotes

Influenza Pathogenesis. Cell Host Microbe. 2016 Jan 13;19(1):102-13.
391. Baron O, Boudi A, Dias C, et al. Stall in Canonical Autophagy-Lysosome Pathways Prompts Nucleophagy-Based Nuclear Breakdown in Neurodegeneration. Curr Biol. 2017 Dec 4;27(23):3626-3642.e6.
392. Miao G, Zhao YG, Zhao H, et al. Mice deficient in the Vici syndrome gene Epg5 exhibit features of retinitis pigmentosa. Autophagy. 2016 Dec;12(12):2263-2270.
393. Herpin A, Englberger E, Zehner M, et al. Defective autophagy through epg5 mutation results in failure to reduce germ plasm and mitochondria. Faseb j. 2015 Oct;29(10):4145-61.
394. Zhao YG, Zhao H, Sun H, et al. Role of Epg5 in selective neurodegeneration and Vici syndrome. Autophagy. 2013 Aug;9(8):1258-62.
395. Zhao H, Zhao YG, Wang X, et al. Mice deficient in Epg5 exhibit selective neuronal vulnerability to degeneration. J Cell Biol. 2013 Mar 18;200(6):731-41.
396. Garyali P, Segvich DM, DePaoli-Roach AA, et al. Protein degradation and quality control in cells from laforin and malin knockout mice. J Biol Chem. 2014 Jul 25;289(30):20606-14.
397. Wang BJ, Her GM, Hu MK, et al. ErbB2 regulates autophagic flux to modulate the proteostasis of APP-CTFs in Alzheimer's disease. Proc Natl Acad Sci U S A. 2017 Apr 11;114(15):E3129-e3138.
398. Lozy F, Cai-McRae X, Teplova I, et al. ERBB2 overexpression suppresses stress-induced autophagy and renders ERBB2-induced mammary tumorigenesis independent of monoallelic Becn1 loss. Autophagy. 2014 Apr;10(4):662-76.
399. Otaka N, Shibata R, Ohashi K, et al. Myonectin Is an Exercise-Induced Myokine That Protects the Heart From Ischemia-Reperfusion Injury. Circ Res. 2018 Dec 7;123(12):1326-1338.
400. Vidal RL, Hetz C. Crosstalk between the UPR and autophagy pathway contributes to handling cellular stress in neurodegenerative disease. Autophagy. 2012 Jun;8(6):970-2.
401. Bell BD, Leverrier S, Weist BM, et al. FADD and caspase-8 control the outcome of autophagic signaling in proliferating T cells. Proc Natl Acad Sci U S A. 2008 Oct 28;105(43):16677-82.
402. Datta K, Suman S, Fornace AJ, Jr. Radiation persistently promoted oxidative stress, activated mTOR via PI3K/Akt, and downregulated autophagy pathway in mouse intestine. Int J Biochem Cell Biol. 2014 Dec;57:167-76.
403. Murdoch JD, Rostosky CM, Gowrisankaran S, et al. Endophilin-A Deficiency Induces the Foxo3a-Fbxo32 Network in the Brain and Causes Dysregulation of Autophagy and the Ubiquitin-Proteasome System. Cell Rep. 2016 Oct 18;17(4):1071-1086.
404. Wakatsuki S, Tokunaga S, Shibata M, et al. GSK3B-mediated phosphorylation of MCL1 regulates axonal autophagy to promote Wallerian degeneration. J Cell Biol. 2017 Feb;216(2):477-493.
405. Livingston MJ, Ding HF, Huang S, et al. Persistent activation of autophagy in kidney tubular cells promotes renal interstitial fibrosis during unilateral ureteral obstruction. Autophagy. 2016 Jun 2;12(6):976-98.
406. Cinque L, Forrester A, Bartolomeo R, et al. FGF signalling regulates bone growth through autophagy. Nature. 2015 Dec 10;528(7581):272-5.

407. Vaccari I, Carbone A, Previtali SC, et al. Loss of Fig4 in both Schwann cells and motor neurons contributes to CMT4J neuropathy. Hum Mol Genet. 2015 Jan 15;24(2):383-96.
408. Campeau PM, Lenk GM, Lu JT, et al. Yunis-Varon syndrome is caused by mutations in FIG4, encoding a phosphoinositide phosphatase. Am J Hum Genet. 2013 May 2;92(5):781-91.
409. Ferguson CJ, Lenk GM, Jones JM, et al. Neuronal expression of Fig4 is both necessary and sufficient to prevent spongiform neurodegeneration. Hum Mol Genet. 2012 Aug 15;21(16):3525-34.
410. Ferguson CJ, Lenk GM, Meisler MH. Defective autophagy in neurons and astrocytes from mice deficient in PI(3,5)P2. Hum Mol Genet. 2009 Dec 15;18(24):4868-78.
411. Lenk GM, Ferguson CJ, Chow CY, et al. Pathogenic mechanism of the FIG4 mutation responsible for Charcot-Marie-Tooth disease CMT4J. PLoS Genet. 2011 Jun;7(6):e1002104.
412. Gomes LC, Scorrano L. High levels of Fis1, a pro-fission mitochondrial protein, trigger autophagy. Biochim Biophys Acta. 2008 Jul-Aug;1777(7-8):860-6.
413. Yuan Q, Chen Z, Santulli G, et al. Functional role of Calstabin2 in age-related cardiac alterations. Sci Rep. 2014 Dec 11;4:7425.
414. Gassen NC, Hartmann J, Schmidt MV, et al. FKBP5/FKBP51 enhances autophagy to synergize with antidepressant action. Autophagy. 2015;11(3):578-80.
415. Incio J, Tam J, Rahbari NN, et al. PlGF/VEGFR-1 Signaling Promotes Macrophage Polarization and Accelerated Tumor Progression in Obesity. Clin Cancer Res. 2016 Jun 15;22(12):2993-3004.
416. Siggs OM, Stockenhuber A, Deobagkar-Lele M, et al. Mutation of Fnip1 is associated with B-cell deficiency, cardiomyopathy, and elevated AMPK activity. Proc Natl Acad Sci U S A. 2016 Jun 28;113(26):E3706-15.
417. Bowman CJ, Ayer DE, Dynlacht BD. Foxk proteins repress the initiation of starvation-induced atrophy and autophagy programs. Nat Cell Biol. 2014 Dec;16(12):1202-14.
418. Li Y, Jiang J, Liu W, et al. microRNA-378 promotes autophagy and inhibits apoptosis in skeletal muscle. Proc Natl Acad Sci U S A. 2018 Nov 13;115(46):E10849-e10858.
419. Shen M, Cao Y, Jiang Y, et al. Melatonin protects mouse granulosa cells against oxidative damage by inhibiting FOXO1-mediated autophagy: Implication of an antioxidation-independent mechanism. Redox Biol. 2018 Sep;18:138-157.
420. Liu P, Liu K, Gu H, et al. High autophagic flux guards ESC identity through coordinating autophagy machinery gene program by FOXO1. Cell Death Differ. 2017 Oct;24(10):1672-1680.
421. Hong-Brown LQ, Brown CR, Navaratnarajah M, et al. FoxO1-AMPK-ULK1 Regulates Ethanol-Induced Autophagy in Muscle by Enhanced ATG14 Association with the BECN1-PIK3C3 Complex. Alcohol Clin Exp Res. 2017 May;41(5):895-910.
422. Li J, Chen T, Xiao M, et al. Mouse Sirt3 promotes autophagy in AngII-induced myocardial hypertrophy through the deacetylation of FoxO1. Oncotarget. 2016 Dec 27;7(52):86648-86659.
423. Liu L, Zheng LD, Zou P, et al. FoxO1 antagonist suppresses autophagy and lipid droplet growth in adipocytes. Cell Cycle. 2016 Aug 2;15(15):2033-41.

424. Wang B, Ding W, Zhang M, et al. Role of FOXO1 in aldosterone-induced autophagy: a compensatory protective mechanism related to podocyte injury. Oncotarget. 2016 Jul 19;7(29):45331-45351.
425. Audesse AJ, Dhakal S, Hassell LA, et al. FOXO3 directly regulates an autophagy network to functionally regulate proteostasis in adult neural stem cells. PLoS Genet. 2019 Apr;15(4):e1008097.
426. Liang R, Camprecios G, Kou Y, et al. A Systems Approach Identifies Essential FOXO3 Functions at Key Steps of Terminal Erythropoiesis. PLoS Genet. 2015 Oct;11(10):e1005526.
427. Cao DJ, Jiang N, Blagg A, et al. Mechanical unloading activates FoxO3 to trigger Bnip3-dependent cardiomyocyte atrophy. J Am Heart Assoc. 2013 Apr 8;2(2):e000016.
428. Wang X, Su H. FoxO3 hastens autophagy and shrinks the heart but does not curtail pathological hypertrophy in adult mice. Cardiovasc Res. 2011 Sep 1;91(4):561-2.
429. Cid-Diaz T, Santos-Zas I, Gonzalez-Sanchez J, et al. Obestatin controls the ubiquitin-proteasome and autophagy-lysosome systems in glucocorticoid-induced muscle cell atrophy. J Cachexia Sarcopenia Muscle. 2017 Dec;8(6):974-990.
430. O'Neill BT, Lee KY, Klaus K, et al. Insulin and IGF-1 receptors regulate FoxO-mediated signaling in muscle proteostasis. J Clin Invest. 2016 Sep 1;126(9):3433-46.
431. Maccarinelli F, Gammella E, Asperti M, et al. Mice lacking mitochondrial ferritin are more sensitive to doxorubicin-mediated cardiotoxicity. J Mol Med (Berl). 2014 Aug;92(8):859-69.
432. Wu W, Tian W, Hu Z, et al. ULK1 translocates to mitochondria and phosphorylates FUNDC1 to regulate mitophagy. EMBO Rep. 2014 May;15(5):566-75.
433. Chandran V, Gao K, Swarup V, et al. Inducible and reversible phenotypes in a novel mouse model of Friedreich's Ataxia. Elife. 2017 Dec 19;6.
434. Yamada E, Okada S, Bastie CC, et al. Fyn phosphorylates AMPK to inhibit AMPK activity and AMP-dependent activation of autophagy. Oncotarget. 2016 Nov 15;7(46):74612-74629.
435. Qin Q, Qu C, Niu T, et al. Nrf2-Mediated Cardiac Maladaptive Remodeling and Dysfunction in a Setting of Autophagy Insufficiency. Hypertension. 2016 Jan;67(1):107-17.
436. Ebert SM, Dyle MC, Kunkel SD, et al. Stress-induced skeletal muscle Gadd45a expression reprograms myonuclei and causes muscle atrophy. J Biol Chem. 2012 Aug 10;287(33):27290-301.
437. Zhang D, Zhang W, Li D, et al. GADD45A inhibits autophagy by regulating the interaction between BECN1 and PIK3C3. Autophagy. 2015;11(12):2247-58.
438. He G, Xu W, Tong L, et al. Gadd45b prevents autophagy and apoptosis against rat cerebral neuron oxygen-glucose deprivation/reperfusion injury. Apoptosis. 2016 Apr;21(4):390-403.
439. Zhang J, Wu K, Xiao X, et al. Autophagy as a regulatory component of erythropoiesis. Int J Mol Sci. 2015 Feb 13;16(2):4083-94.
440. Kang C, Xu Q, Martin TD, et al. The DNA damage response induces inflammation and senescence by inhibiting autophagy of GATA4. Science. 2015 Sep 25;349(6255):aaa5612.
441. Li H, Ham A, Ma TC, et al. Mitochondrial dysfunction and mitophagy defect triggered by

heterozygous GBA mutations. Autophagy. 2019 Jan;15(1):113-130.
442. Du TT, Wang L, Duan CL, et al. GBA deficiency promotes SNCA/alpha-synuclein accumulation through autophagic inhibition by inactivated PPP2A. Autophagy. 2015;11(10):1803-20.
443. Osellame LD, Rahim AA, Hargreaves IP, et al. Mitochondria and quality control defects in a mouse model of Gaucher disease--links to Parkinson's disease. Cell Metab. 2013 Jun 4;17(6):941-53.
444. Xu G, Li Z, Ding L, et al. Intestinal mTOR regulates GLP-1 production in mouse L cells. Diabetologia. 2015 Aug;58(8):1887-97.
445. Tang G, Yue Z, Talloczy Z, et al. Autophagy induced by Alexander disease-mutant GFAP accumulation is regulated by p38/MAPK and mTOR signaling pathways. Hum Mol Genet. 2008 Jun 1;17(11):1540-55.
446. Hernandez-Gea V, Ghiassi-Nejad Z, Rozenfeld R, et al. Autophagy releases lipid that promotes fibrogenesis by activated hepatic stellate cells in mice and in human tissues. Gastroenterology. 2012 Apr;142(4):938-46.
447. LaPash Daniels CM, Paffenroth E, Austin EV, et al. Lithium Decreases Glial Fibrillary Acidic Protein in a Mouse Model of Alexander Disease. PLoS One. 2015;10(9):e0138132.
448. Fields J, Dumaop W, Eleuteri S, et al. HIV-1 Tat alters neuronal autophagy by modulating autophagosome fusion to the lysosome: implications for HIV-associated neurocognitive disorders. J Neurosci. 2015 Feb 4;35(5):1921-38.
449. McFarlane MR, Brown MS, Goldstein JL, et al. Induced ablation of ghrelin cells in adult mice does not decrease food intake, body weight, or response to high-fat diet. Cell Metab. 2014 Jul 1;20(1):54-60.
450. Yu AP, Pei XM, Sin TK, et al. [D-Lys3]-GHRP-6 exhibits pro-autophagic effects on skeletal muscle. Mol Cell Endocrinol. 2015 Feb 5;401:155-64.
451. Sun L, Gao J, Zhao M, et al. A novel cognitive impairment mechanism that astrocytic p-connexin 43 promotes neuronic autophagy via activation of P2X7R and down-regulation of GLT-1 expression in the hippocampus following traumatic brain injury in rats. Behav Brain Res. 2015 Sep 15;291:315-324.
452. Jimenez-Sanchez M, Menzies FM, Chang YY, et al. The Hedgehog signalling pathway regulates autophagy. Nat Commun. 2012;3:1200.
453. Zhang LW, McMahon Tobin GA, Rouse RL. Oleic acid and glucose regulate glucagon-like peptide 1 receptor expression in a rat pancreatic ductal cell line. Toxicol Appl Pharmacol. 2012 Oct 15;264(2):274-83.
454. Wu T, Li Y, Huang D, et al. Regulator of G-protein signaling 19 (RGS19) and its partner Galpha-inhibiting activity polypeptide 3 (GNAI3) are required for zVAD-induced autophagy and cell death in L929 cells. PLoS One. 2014;9(4):e94634.
455. Chang SH, Hong SH, Jiang HL, et al. GOLGA2/GM130, cis-Golgi matrix protein, is a novel target of anticancer gene therapy. Mol Ther. 2012 Nov;20(11):2052-63.
456. Marazziti D, Di Pietro C, Golini E, et al. Induction of macroautophagy by overexpression of the Parkinson's disease-associated GPR37 receptor. Faseb j. 2009 Jun;23(6):1978-87.
457. Baksi S, Jana NR, Bhattacharyya NP, et al. Grb2 is regulated by foxd3 and has roles in preventing accumulation and aggregation of mutant huntingtin. PLoS One.

2013;8(10):e76792.
458. Selimi F, Lohof AM, Heitz S, et al. Lurcher GRID2-induced death and depolarization can be dissociated in cerebellar Purkinje cells. Neuron. 2003 Mar 6;37(5):813-9.
459. Liu QA, Shio H. Mitochondrial morphogenesis, dendrite development, and synapse formation in cerebellum require both Bcl-w and the glutamate receptor delta2. PLoS Genet. 2008 Jun 13;4(6):e1000097.
460. Suresh SN, Verma V, Sateesh S, et al. Neurodegenerative diseases: model organisms, pathology and autophagy. J Genet. 2018 Jul;97(3):679-701.
461. Cruces-Sande M, Vila-Bedmar R, Arcones AC, et al. Involvement of G protein-coupled receptor kinase 2 (GRK2) in the development of non-alcoholic steatosis and steatohepatitis in mice and humans. Biochim Biophys Acta Mol Basis Dis. 2018 Dec;1864(12):3655-3667.
462. Zhou J, Freeman TA, Ahmad F, et al. GSK-3alpha is a central regulator of age-related pathologies in mice. J Clin Invest. 2013 Apr;123(4):1821-32.
463. Holla S, Kurowska-Stolarska M, Bayry J, et al. Selective inhibition of IFNG-induced autophagy by Mir155- and Mir31-responsive WNT5A and SHH signaling. Autophagy. 2014 Feb;10(2):311-30.
464. Liang A, Wang Y, Woodard LE, et al. Loss of glutathione S-transferase A4 accelerates obstruction-induced tubule damage and renal fibrosis. J Pathol. 2012 Dec;228(4):448-58.
465. Zhang L, Chen X, Sharma P, et al. HACE1-dependent protein degradation provides cardiac protection in response to haemodynamic stress. Nat Commun. 2014 Mar 11;5:3430.
466. Liu Z, Chen P, Gao H, et al. Ubiquitylation of autophagy receptor Optineurin by HACE1 activates selective autophagy for tumor suppression. Cancer Cell. 2014 Jul 14;26(1):106-20.
467. Teng AC, Miyake T, Yokoe S, et al. Metformin increases degradation of phospholamban via autophagy in cardiomyocytes. Proc Natl Acad Sci U S A. 2015 Jun 9;112(23):7165-70.
468. Biasiotto G, Di Lorenzo D, Archetti S, et al. Iron and Neurodegeneration: Is Ferritinophagy the Link? Mol Neurobiol. 2016 Oct;53(8):5542-74.
469. Stavoe AKH, Holzbaur ELF. Axonal autophagy: Mini-review for autophagy in the CNS. Neurosci Lett. 2019 Apr 1;697:17-23.
470. Wong YC, Holzbaur EL. The regulation of autophagosome dynamics by huntingtin and HAP1 is disrupted by expression of mutant huntingtin, leading to defective cargo degradation. J Neurosci. 2014 Jan 22;34(4):1293-305.
471. Wang W, Wang Q, Wan D, et al. Histone HIST1H1C/H1.2 regulates autophagy in the development of diabetic retinopathy. Autophagy. 2017 May 4;13(5):941-954.
472. Dong LH, Cheng S, Zheng Z, et al. Histone deacetylase inhibitor potentiated the ability of MTOR inhibitor to induce autophagic cell death in Burkitt leukemia/lymphoma. J Hematol Oncol. 2013 Jul 18;6:53.
473. Cao DJ, Wang ZV, Battiprolu PK, et al. Histone deacetylase (HDAC) inhibitors attenuate cardiac hypertrophy by suppressing autophagy. Proc Natl Acad Sci U S A. 2011 Mar 8;108(10):4123-8.
474. Moresi V, Carrer M, Grueter CE, et al. Histone deacetylases 1 and 2 regulate autophagy

flux and skeletal muscle homeostasis in mice. Proc Natl Acad Sci U S A. 2012 Jan 31;109(5):1649-54.
475. Pigna E, Renzini A, Greco E, et al. HDAC4 preserves skeletal muscle structure following long-term denervation by mediating distinct cellular responses. Skelet Muscle. 2018 Feb 24;8(1):6.
476. Yue F, Li W, Zou J, et al. Spermidine Prolongs Lifespan and Prevents Liver Fibrosis and Hepatocellular Carcinoma by Activating MAP1S-Mediated Autophagy. Cancer Res. 2017 Jun 1;77(11):2938-2951.
477. Yang F, Wang F, Liu Y, et al. Sulforaphane induces autophagy by inhibition of HDAC6-mediated PTEN activation in triple negative breast cancer cells. Life Sci. 2018 Nov 15;213:149-157.
478. Moreno-Gonzalo O, Ramirez-Huesca M, Blas-Rus N, et al. HDAC6 controls innate immune and autophagy responses to TLR-mediated signalling by the intracellular bacteria Listeria monocytogenes. PLoS Pathog. 2017 Dec;13(12):e1006799.
479. Nunez-Andrade N, Iborra S, Trullo A, et al. HDAC6 regulates the dynamics of lytic granules in cytotoxic T lymphocytes. J Cell Sci. 2016 Apr 1;129(7):1305-1311.
480. Chen S, Zhang XJ, Li LX, et al. Histone deacetylase 6 delays motor neuron degeneration by ameliorating the autophagic flux defect in a transgenic mouse model of amyotrophic lateral sclerosis. Neurosci Bull. 2015 Aug;31(4):459-68.
481. Lam HC, Cloonan SM, Bhashyam AR, et al. Histone deacetylase 6-mediated selective autophagy regulates COPD-associated cilia dysfunction. J Clin Invest. 2013 Dec;123(12):5212-30.
482. Lee JY, Koga H, Kawaguchi Y, et al. HDAC6 controls autophagosome maturation essential for ubiquitin-selective quality-control autophagy. Embo j. 2010 Mar 3;29(5):969-80.
483. Tan TC, Crawford DH, Jaskowski LA, et al. Excess iron modulates endoplasmic reticulum stress-associated pathways in a mouse model of alcohol and high-fat diet-induced liver injury. Lab Invest. 2013 Dec;93(12):1295-312.
484. Choi H, Merceron C, Mangiavini L, et al. Hypoxia promotes noncanonical autophagy in nucleus pulposus cells independent of MTOR and HIF1A signaling. Autophagy. 2016 Sep;12(9):1631-46.
485. Cho SJ, Yun SM, Jo C, et al. SUMO1 promotes Abeta production via the modulation of autophagy. Autophagy. 2015;11(1):100-12.
486. Wang Y, Ding Y, Li J, et al. Targeting the Enterohepatic Bile Acid Signaling Induces Hepatic Autophagy via a CYP7A1-AKT-mTOR Axis in Mice. Cell Mol Gastroenterol Hepatol. 2017 Mar;3(2):245-260.
487. Carchman EH, Rao J, Loughran PA, et al. Heme oxygenase-1-mediated autophagy protects against hepatocyte cell death and hepatic injury from infection/sepsis in mice. Hepatology. 2011 Jun;53(6):2053-62.
488. Banreti A, Hudry B, Sass M, et al. Hox proteins mediate developmental and environmental control of autophagy. Dev Cell. 2014 Jan 13;28(1):56-69.
489. Sarkar S, Carroll B, Buganim Y, et al. Impaired autophagy in the lipid-storage disorder Niemann-Pick type C1 disease. Cell Rep. 2013 Dec 12;5(5):1302-15.
490. Guo JY, White E. Autophagy is required for mitochondrial function, lipid metabolism,

growth, and fate of KRAS(G12D)-driven lung tumors. Autophagy. 2013 Oct;9(10):1636-8.
491. Motyl T, Gajkowska B, Zarzynska J, et al. Apoptosis and autophagy in mammary gland remodeling and breast cancer chemotherapy. J Physiol Pharmacol. 2006 Nov;57 Suppl 7:17-32.
492. Zhang N, Wu Y, Lyu X, et al. HSF1 upregulates ATG4B expression and enhances epirubicin-induced protective autophagy in hepatocellular carcinoma cells. Cancer Lett. 2017 Nov 28;409:81-90.
493. Kim E, Sakata K, Liao FF. Bidirectional interplay of HSF1 degradation and UPR activation promotes tau hyperphosphorylation. PLoS Genet. 2017 Jul;13(7):e1006849.
494. Jain N, Rai A, Mishra R, et al. Loss of malin, but not laforin, results in compromised autophagic flux and proteasomal dysfunction in cells exposed to heat shock. Cell Stress Chaperones. 2017 Mar;22(2):307-315.
495. Watanabe Y, Tsujimura A, Taguchi K, et al. HSF1 stress response pathway regulates autophagy receptor SQSTM1/p62-associated proteostasis. Autophagy. 2017 Jan 2;13(1):133-148.
496. Dayalan Naidu S, Dikovskaya D, Gaurilcikaite E, et al. Transcription factors NRF2 and HSF1 have opposing functions in autophagy. Sci Rep. 2017 Sep 8;7(1):11023.
497. Luo T, Fu J, Xu A, et al. PSMD10/gankyrin induces autophagy to promote tumor progression through cytoplasmic interaction with ATG7 and nuclear transactivation of ATG7 expression. Autophagy. 2016 Aug 2;12(8):1355-71.
498. Xiao X, Wang W, Li Y, et al. HSP90AA1-mediated autophagy promotes drug resistance in osteosarcoma. J Exp Clin Cancer Res. 2018 Aug 28;37(1):201.
499. Hsueh YS, Chang HH, Chiang NJ, et al. MTOR inhibition enhances NVP-AUY922-induced autophagy-mediated KIT degradation and cytotoxicity in imatinib-resistant gastrointestinal stromal tumors. Oncotarget. 2014 Nov 30;5(22):11723-36.
500. Wu Y, Ma C, Zhao H, et al. Alleviation of endoplasmic reticulum stress protects against cisplatin-induced ovarian damage. Reprod Biol Endocrinol. 2018 Sep 3;16(1):85.
501. Young CN, Sinadinos A, Lefebvre A, et al. A novel mechanism of autophagic cell death in dystrophic muscle regulated by P2RX7 receptor large-pore formation and HSP90. Autophagy. 2015;11(1):113-30.
502. Chen L, Wang FY, Zeng ZY, et al. MicroRNA-199a acts as a potential suppressor of cardiomyocyte autophagy through targeting Hspa5. Oncotarget. 2017 Sep 8;8(38):63825-63834.
503. Haidar M, Asselbergh B, Adriaenssens E, et al. Neuropathy-causing mutations in HSPB1 impair autophagy by disturbing the formation of SQSTM1/p62 bodies. Autophagy. 2019 Jun;15(6):1051-1068.
504. Checler F, Goiran T, Alves da Costa C. Nuclear TP53: An unraveled function as transcriptional repressor of PINK1. Autophagy. 2018;14(6):1099-1101.
505. Li B, Hu Q, Wang H, et al. Omi/HtrA2 is a positive regulator of autophagy that facilitates the degradation of mutant proteins involved in neurodegenerative diseases. Cell Death Differ. 2010 Nov;17(11):1773-84.
506. Yeh ES, Abt MA, Hill EG. Regulation of cell survival by HUNK mediates breast cancer

resistance to HER2 inhibitors. Breast Cancer Res Treat. 2015 Jan;149(1):91-8.
507. Teramoto T, Chiang HS, Takhampunya R, et al. Gamma interferon-inducible lysosomal thioreductase (GILT) ablation renders mouse fibroblasts sensitive to dengue virus replication. Virology. 2013 Jul 5;441(2):146-51.
508. Kurt R, Chandra PK, Aboulnasr F, et al. Chaperone-Mediated Autophagy Targets IFNAR1 for Lysosomal Degradation in Free Fatty Acid Treated HCV Cell Culture. PLoS One. 2015;10(5):e0125962.
509. King KY, Baldridge MT, Weksberg DC, et al. Irgm1 protects hematopoietic stem cells by negative regulation of IFN signaling. Blood. 2011 Aug 11;118(6):1525-33.
510. Troncoso R, Vicencio JM, Parra V, et al. Energy-preserving effects of IGF-1 antagonize starvation-induced cardiac autophagy. Cardiovasc Res. 2012 Feb 1;93(2):320-9.
511. Liu Q, Guan JZ, Sun Y, et al. Insulin-like growth factor 1 receptor-mediated cell survival in hypoxia depends on the promotion of autophagy via suppression of the PI3K/Akt/mTOR signaling pathway. Mol Med Rep. 2017 Apr;15(4):2136-2142.
512. Wang Y, Gan G, Wang B, et al. Cancer-associated Fibroblasts Promote Irradiated Cancer Cell Recovery Through Autophagy. EBioMedicine. 2017 Mar;17:45-56.
513. Grkovic S, O'Reilly VC, Han S, et al. IGFBP-3 binds GRP78, stimulates autophagy and promotes the survival of breast cancer cells exposed to adverse microenvironments. Oncogene. 2013 May 9;32(19):2412-20.
514. Yin H, Zhang S, Sun Y, et al. MicroRNA-34/449 targets IGFBP-3 and attenuates airway remodeling by suppressing Nur77-mediated autophagy. Cell Death Dis. 2017 Aug 10;8(8):e2998.
515. Chen N, Debnath J. IkappaB kinase complex (IKK) triggers detachment-induced autophagy in mammary epithelial cells independently of the PI3K-AKT-MTORC1 pathway. Autophagy. 2013 Aug;9(8):1214-27.
516. Kanamoto M, Tsuchiya Y, Nakao Y, et al. Structural instability of IkappaB kinase beta promotes autophagic degradation through enhancement of Keap1 binding. PLoS One. 2018;13(11):e0203978.
517. Leonardi M, Perna E, Tronnolone S, et al. Activated kinase screening identifies the IKBKE oncogene as a positive regulator of autophagy. Autophagy. 2019 Feb;15(2):312-326.
518. Seki E. TAK1-dependent autophagy: A suppressor of fatty liver disease and hepatic oncogenesis. Mol Cell Oncol. 2014;1(4).
519. Shi J, Wang H, Guan H, et al. IL10 inhibits starvation-induced autophagy in hypertrophic scar fibroblasts via cross talk between the IL10-IL10R-STAT3 and IL10-AKT-mTOR pathways. Cell Death Dis. 2016 Mar 10;7:e2133.
520. Liu H, Mi S, Li Z, et al. Interleukin 17A inhibits autophagy through activation of PIK3CA to interrupt the GSK3B-mediated degradation of BCL2 in lung epithelial cells. Autophagy. 2013 May;9(5):730-42.
521. Lodder J, Denaes T, Chobert MN, et al. Macrophage autophagy protects against liver fibrosis in mice. Autophagy. 2015;11(8):1280-92.
522. Zhang B, Chen H, Ouyang J, et al. SQSTM1-dependent autophagic degradation of PKM2 inhibits the production of mature IL1B/IL-1beta and contributes to LIPUS-mediated anti-inflammatory effect. Autophagy. 2019 Sep 22:1-17.

523. Lotze MT, Buchser WJ, Liang X. Blocking the interleukin 2 (IL2)-induced systemic autophagic syndrome promotes profound antitumor effects and limits toxicity. Autophagy. 2012 Aug;8(8):1264-6.
524. Mo R, Lai R, Lu J, et al. Enhanced autophagy contributes to protective effects of IL-22 against acetaminophen-induced liver injury. Theranostics. 2018;8(15):4170-4180.
525. Yang C, Tong Y, Ni W, et al. Inhibition of autophagy induced by overexpression of mda-7/interleukin-24 strongly augments the antileukemia activity in vitro and in vivo. Cancer Gene Ther. 2010 Feb;17(2):109-19.
526. Xia F, Deng C, Jiang Y, et al. IL4 (interleukin 4) induces autophagy in B cells leading to exacerbated asthma. Autophagy. 2018;14(3):450-464.
527. Kimura A, Ishida Y, Nosaka M, et al. Exaggerated arsenic nephrotoxicity in female mice through estrogen-dependent impairments in the autophagic flux. Toxicology. 2016 Jan 2;339:9-18.
528. Sosa P, Alcalde-Estevez E, Plaza P, et al. Hyperphosphatemia Promotes Senescence of Myoblasts by Impairing Autophagy Through Ilk Overexpression, A Possible Mechanism Involved in Sarcopenia. Aging Dis. 2018 Oct;9(5):769-784.
529. Wang X, Shen C, Liu Z, et al. Nitazoxanide, an antiprotozoal drug, inhibits late-stage autophagy and promotes ING1-induced cell cycle arrest in glioblastoma. Cell Death Dis. 2018 Oct 9;9(10):1032.
530. Ji J, Petropavlovskaia M, Khatchadourian A, et al. Type 2 diabetes is associated with suppression of autophagy and lipid accumulation in beta-cells. J Cell Mol Med. 2019 Apr;23(4):2890-2900.
531. Caberlotto L, Nguyen TP, Lauria M, et al. Cross-disease analysis of Alzheimer's disease and type-2 Diabetes highlights the role of autophagy in the pathophysiology of two highly comorbid diseases. Sci Rep. 2019 Mar 8;9(1):3965.
532. Zhang L, Cardinal JS, Pan P, et al. Splenocyte apoptosis and autophagy is mediated by interferon regulatory factor 1 during murine endotoxemia. Shock. 2012 May;37(5):511-7.
533. Dong H, Tian L, Li R, et al. IFNg-induced Irgm1 promotes tumorigenesis of melanoma via dual regulation of apoptosis and Bif-1-dependent autophagy. Oncogene. 2015 Oct 16;34(42):5363-71.
534. Tomasetti M, Monaco F, Manzella N, et al. MicroRNA-126 induces autophagy by altering cell metabolism in malignant mesothelioma. Oncotarget. 2016 Jun 14;7(24):36338-36352.
535. Riehle C, Wende AR, Sena S, et al. Insulin receptor substrate signaling suppresses neonatal autophagy in the heart. J Clin Invest. 2013 Dec;123(12):5319-33.
536. Xu S, Wang P, Zhang J, et al. Ai-lncRNA EGOT enhancing autophagy sensitizes paclitaxel cytotoxicity via upregulation of ITPR1 expression by RNA-RNA and RNA-protein interactions in human cancer. Mol Cancer. 2019 Apr 18;18(1):89.
537. Yang J, Yu J, Li D, et al. Store-operated calcium entry-activated autophagy protects EPC proliferation via the CAMKK2-MTOR pathway in ox-LDL exposure. Autophagy. 2017 Jan 2;13(1):82-98.
538. Botbol Y, Patel B, Macian F. Common gamma-chain cytokine signaling is required for macroautophagy induction during CD4+ T-cell activation. Autophagy. 2015;11(10):1864-77.
539. Yogev O, Shaulian E. Jun proteins inhibit autophagy and induce cell death. Autophagy.

2010 May;6(4):566-7.
540. Li K, Zhang TT, Hua F, et al. Metformin reduces TRIB3 expression and restores autophagy flux: an alternative antitumor action. Autophagy. 2018;14(7):1278-1279.
541. Du RH, Tan J, Yan N, et al. Kir6.2 knockout aggravates lipopolysaccharide-induced mouse liver injury via enhancing NLRP3 inflammasome activation. J Gastroenterol. 2014 Apr;49(4):727-36.
542. Taguchi K, Fujikawa N, Komatsu M, et al. Keap1 degradation by autophagy for the maintenance of redox homeostasis. Proc Natl Acad Sci U S A. 2012 Aug 21;109(34):13561-6.
543. Jung DY, Chalasani U, Pan N, et al. KLF15 is a molecular link between endoplasmic reticulum stress and insulin resistance. PLoS One. 2013;8(10):e77851.
544. Zeniya M, Morimoto N, Takahashi D, et al. Kelch-Like Protein 2 Mediates Angiotensin II-With No Lysine 3 Signaling in the Regulation of Vascular Tonus. J Am Soc Nephrol. 2015 Sep;26(9):2129-38.
545. Guo JY, Xia B, White E. Autophagy-mediated tumor promotion. Cell. 2013 Dec 5;155(6):1216-9.
546. Baek A, Yoon S, Kim J, et al. Autophagy and KRT8/keratin 8 protect degeneration of retinal pigment epithelium under oxidative stress. Autophagy. 2017 Feb;13(2):248-263.
547. Kongara S, Kravchuk O, Teplova I, et al. Autophagy regulates keratin 8 homeostasis in mammary epithelial cells and in breast tumors. Mol Cancer Res. 2010 Jun;8(6):873-84.
548. Carmignac V, Svensson M, Korner Z, et al. Autophagy is increased in laminin alpha2 chain-deficient muscle and its inhibition improves muscle morphology in a mouse model of MDC1A. Hum Mol Genet. 2011 Dec 15;20(24):4891-902.
549. Eskelinen EL. Roles of LAMP-1 and LAMP-2 in lysosome biogenesis and autophagy. Mol Aspects Med. 2006 Oct-Dec;27(5-6):495-502.
550. Flores-Toro JA, Go KL, Leeuwenburgh C, et al. Autophagy in the liver: cell's cannibalism and beyond. Arch Pharm Res. 2016 Aug;39(8):1050-61.
551. Sung HK, Chan YK, Han M, et al. Lipocalin-2 (NGAL) Attenuates Autophagy to Exacerbate Cardiac Apoptosis Induced by Myocardial Ischemia. J Cell Physiol. 2017 Aug;232(8):2125-2134.
552. Gogiraju R, Hubert A, Fahrer J, et al. Endothelial Leptin Receptor Deletion Promotes Cardiac Autophagy and Angiogenesis Following Pressure Overload by Suppressing Akt/mTOR Signaling. Circ Heart Fail. 2019 Jan;12(1):e005622.
553. Thurston TL, Wandel MP, von Muhlinen N, et al. Galectin 8 targets damaged vesicles for autophagy to defend cells against bacterial invasion. Nature. 2012 Jan 15;482(7385):414-8.
554. Verlhac P, Viret C, Faure M. Dual function of CALCOCO2/NDP52 during xenophagy. Autophagy. 2015;11(6):965-6.
555. Choi JC, Worman HJ. Reactivation of autophagy ameliorates LMNA cardiomyopathy. Autophagy. 2013 Jan;9(1):110-1.
556. Takagawa T, Kitani A, Fuss I, et al. An increase in LRRK2 suppresses autophagy and enhances Dectin-1-induced immunity in a mouse model of colitis. Sci Transl Med. 2018 Jun 6;10(444).
557. Ahuja S, Knudsen L, Chillappagari S, et al. MAP1LC3B overexpression protects against

Hermansky-Pudlak syndrome type-1-induced defective autophagy in vitro. Am J Physiol Lung Cell Mol Physiol. 2016 Mar 15;310(6):L519-31.
558. Chen Y, Sawada O, Kohno H, et al. Autophagy protects the retina from light-induced degeneration. J Biol Chem. 2013 Mar 15;288(11):7506-18.
559. Liu L, McKeehan WL, Wang F, et al. MAP1S enhances autophagy to suppress tumorigenesis. Autophagy. 2012 Feb 1;8(2):278-80.
560. Baldari S, Ubertini V, Garufi A, et al. Targeting MKK3 as a novel anticancer strategy: molecular mechanisms and therapeutical implications. Cell Death Dis. 2015 Jan 29;6:e1621.
561. Rui YN, Xu Z, Chen Z, et al. The GST-BHMT assay reveals a distinct mechanism underlying proteasome inhibition-induced macroautophagy in mammalian cells. Autophagy. 2015;11(5):812-32.
562. Keil E, Hocker R, Schuster M, et al. Phosphorylation of Atg5 by the Gadd45beta-MEKK4-p38 pathway inhibits autophagy. Cell Death Differ. 2013 Feb;20(2):321-32.
563. Inokuchi-Shimizu S, Park EJ, Roh YS, et al. TAK1-mediated autophagy and fatty acid oxidation prevent hepatosteatosis and tumorigenesis. J Clin Invest. 2014 Aug;124(8):3566-78.
564. Xiao Y, Liu H, Yu J, et al. MAPK1/3 regulate hepatic lipid metabolism via ATG7-dependent autophagy. Autophagy. 2016;12(3):592-3.
565. Yang X, Xiang X, Xia M, et al. Inhibition of JNK3 promotes apoptosis induced by BH3 mimetic S1 in chemoresistant human ovarian cancer cells. Anat Rec (Hoboken). 2015 Feb;298(2):386-95.
566. Neumann Y, Bruns SA, Rohde M, et al. Intracellular Staphylococcus aureus eludes selective autophagy by activating a host cell kinase. Autophagy. 2016 Nov;12(11):2069-2084.
567. Barutcu SA, Girnius N, Vernia S, et al. Role of the MAPK/cJun NH2-terminal kinase signaling pathway in starvation-induced autophagy. Autophagy. 2018;14(9):1586-1595.
568. Fu MM, Holzbaur EL. MAPK8IP1/JIP1 regulates the trafficking of autophagosomes in neurons. Autophagy. 2014;10(11):2079-81.
569. Schaeffer V, Goedert M. Stimulation of autophagy is neuroprotective in a mouse model of human tauopathy. Autophagy. 2012 Nov;8(11):1686-7.
570. Cheng J, Liao Y, Xiao L, et al. Autophagy regulates MAVS signaling activation in a phosphorylation-dependent manner in microglia. Cell Death Differ. 2017 Feb;24(2):276-287.
571. Ezquerro S, Fruhbeck G, Rodriguez A. Ghrelin and autophagy. Curr Opin Clin Nutr Metab Care. 2017 Sep;20(5):402-408.
572. Fitzwalter BE, Towers CG, Sullivan KD, et al. Autophagy Inhibition Mediates Apoptosis Sensitization in Cancer Therapy by Relieving FOXO3a Turnover. Dev Cell. 2018 Mar 12;44(5):555-565.e3.
573. Zhang W, Hou J, Wang X, et al. PTPRO-mediated autophagy prevents hepatosteatosis and tumorigenesis. Oncotarget. 2015 Apr 20;6(11):9420-33.
574. Ni HM, Williams JA, Jaeschke H, et al. Zonated induction of autophagy and mitochondrial spheroids limits acetaminophen-induced necrosis in the liver. Redox Biol.

2013 Aug 26;1:427-32.
575. Xu X, Pang J, Chen Y, et al. Macrophage Migration Inhibitory Factor (MIF) Deficiency Exacerbates Aging-Induced Cardiac Remodeling and Dysfunction Despite Improved Inflammation: Role of Autophagy Regulation. Sci Rep. 2016 Mar 4;6:22488.
576. Jara P, Calyeca J, Romero Y, et al. Matrix metalloproteinase (MMP)-19-deficient fibroblasts display a profibrotic phenotype. Am J Physiol Lung Cell Mol Physiol. 2015 Mar 15;308(6):L511-22.
577. Li WD, Hu N, Lei FR, et al. Autophagy inhibits endothelial progenitor cells migration via the regulation of MMP2, MMP9 and uPA under normoxia condition. Biochem Biophys Res Commun. 2015 Oct 23;466(3):376-80.
578. Bhattacharya A, Wei Q, Shin JN, et al. Autophagy Is Required for Neutrophil-Mediated Inflammation. Cell Rep. 2015 Sep 22;12(11):1731-9.
579. Cadwell K, Philips JA. Autophagy meets phagocytosis. Immunity. 2013 Sep 19;39(3):425-7.
580. Collins-Hooper H, Sartori R, Giallourou N, et al. Symmorphosis through dietary regulation: a combinatorial role for proteolysis, autophagy and protein synthesis in normalising muscle metabolism and function of hypertrophic mice after acute starvation. PLoS One. 2015;10(3):e0120524.
581. Lahiri A, Abraham C. Activation of pattern recognition receptors up-regulates metallothioneins, thereby increasing intracellular accumulation of zinc, autophagy, and bacterial clearance by macrophages. Gastroenterology. 2014 Oct;147(4):835-46.
582. Liuzzi JP, Guo L, Yoo C, et al. Zinc and autophagy. Biometals. 2014 Dec;27(6):1087-96.
583. Al-Qusairi L, Prokic I, Amoasii L, et al. Lack of myotubularin (MTM1) leads to muscle hypotrophy through unbalanced regulation of the autophagy and ubiquitin-proteasome pathways. Faseb j. 2013 Aug;27(8):3384-94.
584. Kovacs T, Billes V, Komlos M, et al. The small molecule AUTEN-99 (autophagy enhancer-99) prevents the progression of neurodegenerative symptoms. Sci Rep. 2017 Feb 16;7:42014.
585. Mochizuki Y, Ohashi R, Kawamura T, et al. Phosphatidylinositol 3-phosphatase myotubularin-related protein 6 (MTMR6) is regulated by small GTPase Rab1B in the early secretory and autophagic pathways. J Biol Chem. 2013 Jan 11;288(2):1009-21.
586. Dortet L, Mostowy S, Samba-Louaka A, et al. Recruitment of the major vault protein by InlK: a Listeria monocytogenes strategy to avoid autophagy. PLoS Pathog. 2011 Aug;7(8):e1002168.
587. Wang J, Huang S, Tian R, et al. The protective autophagy activated by GANT-61 in MYCN amplified neuroblastoma cells is mediated by PERK. Oncotarget. 2018 Mar 6;9(18):14413-14427.
588. Kader M, Alaoui-El-Azher M, Vorhauer J, et al. MyD88-dependent inflammasome activation and autophagy inhibition contributes to Ehrlichia-induced liver injury and toxic shock. PLoS Pathog. 2017 Oct;13(10):e1006644.
589. Byrne BG, Dubuisson JF, Joshi AD, et al. Inflammasome components coordinate autophagy and pyroptosis as macrophage responses to infection. MBio. 2013 Feb 12;4(1):e00620-12.
590. Saikh KU, Dankmeyer JL, Zeng X, et al. An increase in intracellular p62/NBR1 and

persistence of Burkholderia mallei and B. pseudomallei in infected mice linked to autophagy deficiency. Immun Inflamm Dis. 2019 Mar;7(1):7-21.

591. Dowdle WE, Nyfeler B, Nagel J, et al. Selective VPS34 inhibitor blocks autophagy and uncovers a role for NCOA4 in ferritin degradation and iron homeostasis in vivo. Nat Cell Biol. 2014 Nov;16(11):1069-79.

592. Rossi S, Stoppani E, Martinet W, et al. The cytosolic sialidase Neu2 is degraded by autophagy during myoblast atrophy. Biochim Biophys Acta. 2009 Aug;1790(8):817-28.

593. Yang H, Ni HM, Ding WX. The double-edged sword of MTOR in autophagy deficiency induced-liver injury and tumorigenesis. Autophagy. 2019 Sep;15(9):1671-1673.

594. Kim M, Jung JY, Choi S, et al. GFRA1 promotes cisplatin-induced chemoresistance in osteosarcoma by inducing autophagy. Autophagy. 2017 Jan 2;13(1):149-168.

595. Jabir MS, Sulaiman GM, Taqi ZJ, et al. Iraqi propolis increases degradation of IL-1beta and NLRC4 by autophagy following Pseudomonas aeruginosa infection. Microbes Infect. 2018 Feb;20(2):89-100.

596. Jounai N, Kobiyama K, Shiina M, et al. NLRP4 negatively regulates autophagic processes through an association with beclin1. J Immunol. 2011 Feb 1;186(3):1646-55.

597. Wlodarska M, Thaiss CA, Nowarski R, et al. NLRP6 inflammasome orchestrates the colonic host-microbial interface by regulating goblet cell mucus secretion. Cell. 2014 Feb 27;156(5):1045-59.

598. Yin H, Yang Q, Cao Z, et al. Activation of NLRX1-mediated autophagy accelerates the ototoxic potential of cisplatin in auditory cells. Toxicol Appl Pharmacol. 2018 Mar 15;343:16-28.

599. Travassos LH, Carneiro LA, Ramjeet M, et al. Nod1 and Nod2 direct autophagy by recruiting ATG16L1 to the plasma membrane at the site of bacterial entry. Nat Immunol. 2010 Jan;11(1):55-62.

600. Poillet L, Pernodet N, Boyer-Guittaut M, et al. QSOX1 inhibits autophagic flux in breast cancer cells. PLoS One. 2014;9(1):e86641.

601. Zhou W, Yao Y, Li J, et al. TIGAR Attenuates High Glucose-Induced Neuronal Apoptosis via an Autophagy Pathway. Front Mol Neurosci. 2019;12:193.

602. Cui Y, Wang Y, Li G, et al. The Nox1/Nox4 inhibitor attenuates acute lung injury induced by ischemia-reperfusion in mice. PLoS One. 2018;13(12):e0209444.

603. Tharkeshwar AK, Trekker J, Vermeire W, et al. A novel approach to analyze lysosomal dysfunctions through subcellular proteomics and lipidomics: the case of NPC1 deficiency. Sci Rep. 2017 Jan 30;7:41408.

604. Ferder IC, Fung L, Ohguchi Y, et al. Meiotic gatekeeper STRA8 suppresses autophagy by repressing Nr1d1 expression during spermatogenesis in mice. PLoS Genet. 2019 May;15(5):e1008084.

605. Wang YQ, Wang L, Zhang MY, et al. Necrostatin-1 suppresses autophagy and apoptosis in mice traumatic brain injury model. Neurochem Res. 2012 Sep;37(9):1849-58.

606. Liu ZQ, Lee JN, Son M, et al. Ciliogenesis is reciprocally regulated by PPARA and NR1H4/FXR through controlling autophagy in vitro and in vivo. Autophagy. 2018;14(6):1011-1027.

607. Mao YQ, Fan XM. Autophagy: A new therapeutic target for liver fibrosis. World J Hepatol. 2015 Aug 8;7(16):1982-6.

608. Bouzas-Rodriguez J, Zarraga-Granados G, Sanchez-Carbente Mdel R, et al. The nuclear receptor NR4A1 induces a form of cell death dependent on autophagy in mammalian cells. PLoS One. 2012;7(10):e46422.
609. Yang C, Cai CZ, Song JX, et al. NRBF2 is involved in the autophagic degradation process of APP-CTFs in Alzheimer disease models. Autophagy. 2017;13(12):2028-2040.
610. Tang T, Gao D, Yang X, et al. Exogenous Netrin-1 Inhibits Autophagy of Ischemic Brain Tissues and Hypoxic Neurons via PI3K/mTOR Pathway in Ischemic Stroke. J Stroke Cerebrovasc Dis. 2019 May;28(5):1338-1345.
611. Shiraki M, Xu X, Iovanna JL, et al. Deficiency of stress-associated gene Nupr1 increases bone volume by attenuating differentiation of osteoclasts and enhancing differentiation of osteoblasts. Faseb j. 2019 Aug;33(8):8836-8852.
612. Ding Z, Liu S, Wang X, et al. Lectin-like oxidized low-density lipoprotein receptor-1 regulates autophagy and Toll-like receptor 4 in the brain of hypertensive mice. J Hypertens. 2015 Mar;33(3):525-33; discussion 533.
613. Romanello V, Scalabrin M, Albiero M, et al. Inhibition of the Fission Machinery Mitigates OPA1 Impairment in Adult Skeletal Muscles. Cells. 2019 Jun 15;8(6).
614. Oakes JA, Davies MC, Collins MO. TBK1: a new player in ALS linking autophagy and neuroinflammation. Mol Brain. 2017 Feb 2;10(1):5.
615. Kim JE, Ko AR, Hyun HW, et al. P2RX7-MAPK1/2-SP1 axis inhibits MTOR independent HSPB1-mediated astroglial autophagy. Cell Death Dis. 2018 May 1;9(5):546.
616. Guo L, Li Y, Tian Y, et al. eIF2alpha promotes vascular remodeling via autophagy in monocrotaline-induced pulmonary arterial hypertension rats. Drug Des Devel Ther. 2019;13:2799-2809.
617. Gan-Or Z, Dion PA, Rouleau GA. Genetic perspective on the role of the autophagy-lysosome pathway in Parkinson disease. Autophagy. 2015;11(9):1443-57.
618. Ding Z, Wang X, Liu S, et al. PCSK9 expression in the ischaemic heart and its relationship to infarct size, cardiac function, and development of autophagy. Cardiovasc Res. 2018 Nov 1;114(13):1738-1751.
619. Taylor A, Schenkel LC, Yokich M, et al. Adaptations to excess choline in insulin resistant and Pcyt2 deficient skeletal muscle. Biochem Cell Biol. 2017 Apr;95(2):223-231.
620. Tousif S, Singh Y, Prasad DV, et al. T cells from Programmed Death-1 deficient mice respond poorly to Mycobacterium tuberculosis infection. PLoS One. 2011 May 12;6(5):e19864.
621. Wang L, Ye N, Lian X, et al. MiR-208a-3p aggravates autophagy through the PDCD4-ATG5 pathway in Ang II-induced H9c2 cardiomyoblasts. Biomed Pharmacother. 2018 Feb;98:1-8.
622. Yang T, Moore M, He F. Pten regulates neural crest proliferation and differentiation during mouse craniofacial development. Dev Dyn. 2018 Feb;247(2):304-314.
623. Torres A, Gubbiotti MA, Iozzo RV. Decorin-inducible Peg3 Evokes Beclin 1-mediated Autophagy and Thrombospondin 1-mediated Angiostasis. J Biol Chem. 2017 Mar 24;292(12):5055-5069.
624. Rami A, Fekadu J, Rawashdeh O. The Hippocampal Autophagic Machinery is Depressed in the Absence of the Circadian Clock Protein PER1 that may Lead to Vulnerability

During Cerebral Ischemia. Curr Neurovasc Res. 2017;14(3):207-214.
625. Jiang L, Hara-Kuge S, Yamashita S, et al. Peroxin Pex14p is the key component for coordinated autophagic degradation of mammalian peroxisomes by direct binding to LC3-II. Genes Cells. 2015 Jan;20(1):36-49.
626. Lu W, Sun J, Yoon JS, et al. Mitochondrial Protein PGAM5 Regulates Mitophagic Protection against Cell Necroptosis. PLoS One. 2016;11(1):e0147792.
627. Talarek JR, Piacentini AN, Konja AC, et al. The MRL/MpJ Mouse Strain Is Not Protected From Muscle Atrophy and Weakness After Rotator Cuff Tear. J Orthop Res. 2019 Nov 7.
628. Yu X, Long YC, Shen HM. Differential regulatory functions of three classes of phosphatidylinositol and phosphoinositide 3-kinases in autophagy. Autophagy. 2015;11(10):1711-28.
629. Hu Y, Huang Y, Yi Y, et al. Single-cell RNA sequencing highlights transcription activity of autophagy-related genes during hematopoietic stem cell formation in mouse embryos. Autophagy. 2017 Apr 3;13(4):770-771.
630. You L, Wang Z, Li H, et al. The role of STAT3 in autophagy. Autophagy. 2015;11(5):729-39.
631. Ma X, Zhang S, He L, et al. MTORC1-mediated NRBF2 phosphorylation functions as a switch for the class III PtdIns3K and autophagy. Autophagy. 2017 Mar 4;13(3):592-607.
632. Yordanov TE, Hipolito VEB, Liebscher G, et al. Biogenesis of lysosome-related organelles complex-1 (BORC) regulates late endosomal/lysosomal size through PIKfyve-dependent phosphatidylinositol-3,5-bisphosphate. Traffic. 2019 Sep;20(9):674-696.
633. Yang T, Ren C, Qiao P, et al. PIM2-mediated phosphorylation of hexokinase 2 is critical for tumor growth and paclitaxel resistance in breast cancer. Oncogene. 2018 Nov;37(45):5997-6009.
634. Barodia SK, Creed RB, Goldberg MS. Parkin and PINK1 functions in oxidative stress and neurodegeneration. Brain Res Bull. 2017 Jul;133:51-59.
635. Shu G, Tang Y, Zhou Y, et al. Zac1 is a histone acetylation-regulated NF-kappaB suppressor that mediates histone deacetylase inhibitor-induced apoptosis. Cell Death Differ. 2011 Dec;18(12):1825-35.
636. Umebayashi H, Mizokami A, Matsuda M, et al. Phospholipase C-related catalytically inactive protein, a novel microtubule-associated protein 1 light chain 3-binding protein, negatively regulates autophagosome formation. Biochem Biophys Res Commun. 2013 Mar 8;432(2):268-74.
637. Huang A, Zhang H, Chen S, et al. miR-34a expands myeloid-derived suppressor cells via apoptosis inhibition. Exp Cell Res. 2014 Aug 15;326(2):259-66.
638. McEwan DG, Dikic I. PLEKHM1: Adapting to life at the lysosome. Autophagy. 2015 Apr 3;11(4):720-2.
639. Mo H, He J, Yuan Z, et al. PLK1 contributes to autophagy by regulating MYC stabilization in osteosarcoma cells. Onco Targets Ther. 2019;12:7527-7536.
640. Gu J, Hu W, Song ZP, et al. Rapamycin Inhibits Cardiac Hypertrophy by Promoting Autophagy via the MEK/ERK/Beclin-1 Pathway. Front Physiol. 2016;7:104.
641. Li P, Yang S, Hu D, et al. Enterovirus 71 VP1 promotes mouse Schwann cell autophagy via ER stressmediated PMP22 upregulation. Int J Mol Med. 2019 Aug;44(2):759-767.

642. Sharara-Chami RI, Zhou Y, Ebert S, et al. Epinephrine deficiency results in intact glucose counter-regulation, severe hepatic steatosis and possible defective autophagy in fasting mice. Int J Biochem Cell Biol. 2012 Jun;44(6):905-13.
643. Li Y, Zong WX, Ding WX. Recycling the danger via lipid droplet biogenesis after autophagy. Autophagy. 2017;13(11):1995-1997.
644. Dupont N, Chauhan S, Arko-Mensah J, et al. Neutral lipid stores and lipase PNPLA5 contribute to autophagosome biogenesis. Curr Biol. 2014 Mar 17;24(6):609-20.
645. Kim KY, Jang HJ, Yang YR, et al. SREBP-2/PNPLA8 axis improves non-alcoholic fatty liver disease through activation of autophagy. Sci Rep. 2016 Oct 21;6:35732.
646. Brown DI, Lassegue B, Lee M, et al. Poldip2 knockout results in perinatal lethality, reduced cellular growth and increased autophagy of mouse embryonic fibroblasts. PLoS One. 2014;9(5):e96657.
647. Luo R, Su LY, Li G, et al. Activation of PPARA-mediated autophagy reduces Alzheimer disease-like pathology and cognitive decline in a murine model. Autophagy. 2019 Mar 22:1-18.
648. Salazar G, Cullen A, Huang J, et al. SQSTM1/p62 and PPARGC1A/PGC-1alpha at the interface of autophagy and vascular senescence. Autophagy. 2019 Aug 28:1-19.
649. Carreira RS, Lee Y, Ghochani M, et al. Cyclophilin D is required for mitochondrial removal by autophagy in cardiac cells. Autophagy. 2010 May;6(4):462-72.
650. Torii S, Yoshida T, Arakawa S, et al. Identification of PPM1D as an essential Ulk1 phosphatase for genotoxic stress-induced autophagy. EMBO Rep. 2016 Nov;17(11):1552-1564.
651. Ito S, Tanaka Y, Oshino R, et al. GADD34 inhibits activation-induced apoptosis of macrophages through enhancement of autophagy. Sci Rep. 2015 Feb 9;5:8327.
652. Cho MH, Cho K, Kang HJ, et al. Autophagy in microglia degrades extracellular beta-amyloid fibrils and regulates the NLRP3 inflammasome. Autophagy. 2014 Oct 1;10(10):1761-75.
653. Mavrakis M, Lippincott-Schwartz J, Stratakis CA, et al. mTOR kinase and the regulatory subunit of protein kinase A (PRKAR1A) spatially and functionally interact during autophagosome maturation. Autophagy. 2007 Mar-Apr;3(2):151-3.
654. Oltulu F, Kocaturk DC, Adali Y, et al. Autophagy and mTOR pathways in mouse embryonic stem cell, lung cancer and somatic fibroblast cell lines. J Cell Biochem. 2019 Oct;120(10):18066-18076.
655. Patergnani S, Marchi S, Rimessi A, et al. PRKCB/protein kinase C, beta and the mitochondrial axis as key regulators of autophagy. Autophagy. 2013 Sep;9(9):1367-85.
656. Chou LF, Cheng YL, Hsieh CY, et al. Effect of Trehalose Supplementation on Autophagy and Cystogenesis in a Mouse Model of Polycystic Kidney Disease. Nutrients. 2018 Dec 25;11(1).
657. Piquereau J, Godin R, Deschenes S, et al. Protective role of PARK2/Parkin in sepsis-induced cardiac contractile and mitochondrial dysfunction. Autophagy. 2013 Nov 1;9(11):1837-51.
658. Sun Y, Grabowski GA. Altered autophagy in the mice with a deficiency of saposin A and saposin B. Autophagy. 2013 Jul;9(7):1115-6.
659. Wang Q, Pan F, Li S, et al. The prognostic value of the proteasome activator subunit gene

family in skin cutaneous melanoma. J Cancer. 2019;10(10):2205-2219.
660. Wen F, Zhuge W, Wang J, et al. Oridonin prevents insulin resistance-mediated cognitive disorder through PTEN/Akt pathway and autophagy in minimal hepatic encephalopathy. J Cell Mol Med. 2019 Sep 30.
661. Yao J, Jia L, Khan N, et al. Deletion of autophagy inducer RB1CC1 results in degeneration of the retinal pigment epithelium. Autophagy. 2015;11(6):939-53.
662. Spalinger MR, Lang S, Vavricka SR, et al. Protein tyrosine phosphatase non-receptor type 22 modulates NOD2-induced cytokine release and autophagy. PLoS One. 2013;8(8):e72384.
663. Cai T, Hirai H, Zhang G, et al. Deletion of Ia-2 and/or Ia-2beta in mice decreases insulin secretion by reducing the number of dense core vesicles. Diabetologia. 2011 Sep;54(9):2347-57.
664. Li D, Huang S, Zhu J, et al. Exosomes from MiR-21-5p-Increased Neurons Play a Role in Neuroprotection by Suppressing Rab11a-Mediated Neuronal Autophagy In Vitro After Traumatic Brain Injury. Med Sci Monit. 2019 Mar 12;25:1871-1885.
665. Nian FS, Li LL, Cheng CY, et al. Rab18 Collaborates with Rab7 to Modulate Lysosomal and Autophagy Activities in the Nervous System: an Overlapping Mechanism for Warburg Micro Syndrome and Charcot-Marie-Tooth Neuropathy Type 2B. Mol Neurobiol. 2019 Sep;56(9):6095-6105.
666. Feng ZZ, Jiang AJ, Mao AW, et al. The Salmonella effectors SseF and SseG inhibit Rab1A-mediated autophagy to facilitate intracellular bacterial survival and replication. J Biol Chem. 2018 Jun 22;293(25):9662-9673.
667. Sidjanin DJ, Park AK, Ronchetti A, et al. TBC1D20 mediates autophagy as a key regulator of autophagosome maturation. Autophagy. 2016 Oct 2;12(10):1759-1775.
668. Yla-Anttila P, Mikkonen E, Happonen KE, et al. RAB24 facilitates clearance of autophagic compartments during basal conditions. Autophagy. 2015;11(10):1833-48.
669. Pei Y, Yue L, Zhang W, et al. Improvement in Mouse iPSC Induction by Rab32 Reveals the Importance of Lipid Metabolism during Reprogramming. Sci Rep. 2015 Nov 12;5:16539.
670. Hegedus K, Takats S, Boda A, et al. The Ccz1-Mon1-Rab7 module and Rab5 control distinct steps of autophagy. Mol Biol Cell. 2016 Oct 15;27(20):3132-3142.
671. Pilli M, Arko-Mensah J, Ponpuak M, et al. TBK-1 promotes autophagy-mediated antimicrobial defense by controlling autophagosome maturation. Immunity. 2012 Aug 24;37(2):223-34.
672. Hirota Y, Yamashita S, Kurihara Y, et al. Mitophagy is primarily due to alternative autophagy and requires the MAPK1 and MAPK14 signaling pathways. Autophagy. 2015;11(2):332-43.
673. Su Q, Liu Y, Lv XW, et al. Inhibition of lncRNA TUG1 upregulates miR-142-3p to ameliorate myocardial injury during ischemia and reperfusion via targeting HMGB1- and Rac1-induced autophagy. J Mol Cell Cardiol. 2019 Aug;133:12-25.
674. Eum KH, Lee M. Crosstalk between autophagy and apoptosis in the regulation of paclitaxel-induced cell death in v-Ha-ras-transformed fibroblasts. Mol Cell Biochem. 2011 Feb;348(1-2):61-8.
675. Singh MK, Martin APJ, Joffre C, et al. Localization of RalB signaling at endomembrane

compartments and its modulation by autophagy. Sci Rep. 2019 Jun 20;9(1):8910.
676. Rifki OF, Bodemann BO, Battiprolu PK, et al. RalGDS-dependent cardiomyocyte autophagy is required for load-induced ventricular hypertrophy. J Mol Cell Cardiol. 2013 Jun;59:128-38.
677. Sarkar S, Ravikumar B, Floto RA, et al. Rapamycin and mTOR-independent autophagy inducers ameliorate toxicity of polyglutamine-expanded huntingtin and related proteinopathies. Cell Death Differ. 2009 Jan;16(1):46-56.
678. Qiao L, Zhang X, Liu M, et al. Ginsenoside Rb1 Enhances Atherosclerotic Plaque Stability by Improving Autophagy and Lipid Metabolism in Macrophage Foam Cells. Front Pharmacol. 2017;8:727.
679. Ermak G, Davies KJA. Chronic high levels of the RCAN1-1 protein may promote neurodegeneration and Alzheimer disease. Free Radic Biol Med. 2013 Sep;62:47-51.
680. Pahari S, Negi S, Aqdas M, et al. Induction of autophagy through CLEC4E in combination with TLR4: an innovative strategy to restrict the survival of Mycobacterium tuberculosis. Autophagy. 2019 Sep 8:1-23.
681. Newman AC, Kemp AJ, Drabsch Y, et al. Autophagy acts through TRAF3 and RELB to regulate gene expression via antagonism of SMAD proteins. Nat Commun. 2017 Nov 16;8(1):1537.
682. Rudat S, Pfaus A, Cheng YY, et al. RET-mediated autophagy suppression as targetable co-dependence in acute myeloid leukemia. Leukemia. 2018 Oct;32(10):2189-2202.
683. Wang J, Yang K, Zhou L, et al. MicroRNA-155 promotes autophagy to eliminate intracellular mycobacteria by targeting Rheb. PLoS Pathog. 2013;9(10):e1003697.
684. Belaid A, Ndiaye PD, Cerezo M, et al. Autophagy and SQSTM1 on the RHOA(d) again: emerging roles of autophagy in the degradation of signaling proteins. Autophagy. 2014 Feb;10(2):201-8.
685. Garcia-Mariscal A, Li H, Pedersen E, et al. Loss of RhoA promotes skin tumor formation and invasion by upregulation of RhoB. Oncogene. 2018 Feb 15;37(7):847-860.
686. Lim J, Park H, Heisler J, et al. Autophagy regulates inflammatory programmed cell death via turnover of RHIM-domain proteins. Elife. 2019 Jul 9;8.
687. Gao C, Chen J, Fan F, et al. RIPK2-Mediated Autophagy and Negatively Regulated ROS-NLRP3 Inflammasome Signaling in GMCs Stimulated with High Glucose. Mediators Inflamm. 2019;2019:6207563.
688. Lin SY, Hsieh SY, Fan YT, et al. Necroptosis promotes autophagy-dependent upregulation of DAMP and results in immunosurveillance. Autophagy. 2018;14(5):778-795.
689. Morel E, Dupont N, Codogno P. Autophagy regulation: RNF2 targets AMBRA1. Cell Res. 2014 Sep;24(9):1029-30.
690. Zhang Y, Kang YM, Tian C, et al. Overexpression of Nrdp1 in the heart exacerbates doxorubicin-induced cardiac dysfunction in mice. PLoS One. 2011;6(6):e21104.
691. Kuang E, Okumura CY, Sheffy-Levin S, et al. Regulation of ATG4B stability by RNF5 limits basal levels of autophagy and influences susceptibility to bacterial infection. PLoS Genet. 2012;8(10):e1003007.
692. Shi J, Surma M, Yang Y, et al. Disruption of both ROCK1 and ROCK2 genes in cardiomyocytes promotes autophagy and reduces cardiac fibrosis during aging. Faseb j. 2019 Jun;33(6):7348-7362.

693. Heijnen HF, van Wijk R, Pereboom TC, et al. Ribosomal protein mutations induce autophagy through S6 kinase inhibition of the insulin pathway. PLoS Genet. 2014;10(5):e1004371.
694. Zhang X, Cai L, Zhao S, et al. CX-F9, a novel RSK2 inhibitor, suppresses cutaneous melanoma cells proliferation and metastasis through regulating autophagy. Biochem Pharmacol. 2019 Oct;168:14-25.
695. Wang J, Wu ML, Cao SP, et al. Cycloastragenol ameliorates experimental heart damage in rats by promoting myocardial autophagy via inhibition of AKT1-RPS6KB1 signaling. Biomed Pharmacother. 2018 Nov;107:1074-1081.
696. Efeyan A, Schweitzer LD, Bilate AM, et al. RagA, but not RagB, is essential for embryonic development and adult mice. Dev Cell. 2014 May 12;29(3):321-9.
697. Grumati P, Morozzi G, Holper S, et al. Full length RTN3 regulates turnover of tubular endoplasmic reticulum via selective autophagy. Elife. 2017 Jun 15;6.
698. Cheng X, Sun Q. RUBCNL/Pacer and RUBCN/Rubicon in regulation of autolysosome formation and lipid metabolism. Autophagy. 2019 Jun;15(6):1120-1121.
699. Pedrozo Z, Torrealba N, Fernandez C, et al. Cardiomyocyte ryanodine receptor degradation by chaperone-mediated autophagy. Cardiovasc Res. 2013 May 1;98(2):277-85.
700. Sinha RA, Singh BK, Zhou J, et al. Loss of ULK1 increases RPS6KB1-NCOR1 repression of NR1H/LXR-mediated Scd1 transcription and augments lipotoxicity in hepatic cells. Autophagy. 2017 Jan 2;13(1):169-186.
701. Dron M, Bailly Y, Beringue V, et al. SCRG1, a potential marker of autophagy in transmissible spongiform encephalopathies. Autophagy. 2006 Jan-Mar;2(1):58-60.
702. Sun Z, Zhou S, Tang J, et al. Sec23a mediates miR-200c augmented oligometastatic to polymetastatic progression. EBioMedicine. 2018 Nov;37:47-55.
703. Lee JA, Yerbury JJ, Farrawell N, et al. SerpinB2 (PAI-2) Modulates Proteostasis via Binding Misfolded Proteins and Promotion of Cytoprotective Inclusion Formation. PLoS One. 2015;10(6):e0130136.
704. Ambrosio S, Sacca CD, Amente S, et al. Lysine-specific demethylase LSD1 regulates autophagy in neuroblastoma through SESN2-dependent pathway. Oncogene. 2017 Nov 30;36(48):6701-6711.
705. Miki Y, Tanji K, Mori F, et al. Autophagy mediators (FOXO1, SESN3 and TSC2) in Lewy body disease and aging. Neurosci Lett. 2018 Sep 25;684:35-41.
706. Zuleger T, Heinzelbecker J, Takacs Z, et al. SGK1 Inhibits Autophagy in Murine Muscle Tissue. Oxid Med Cell Longev. 2018;2018:4043726.
707. Jung S, Choe S, Woo H, et al. Autophagic death of neural stem cells mediates chronic stress-induced decline of adult hippocampal neurogenesis and cognitive deficits. Autophagy. 2019 Jun 24:1-19.
708. Onnis A, Cianfanelli V, Cassioli C, et al. The pro-oxidant adaptor p66SHC promotes B cell mitophagy by disrupting mitochondrial integrity and recruiting LC3-II. Autophagy. 2018;14(12):2117-2138.
709. Hu L, Zhu Z, Hai B, et al. Intragland Shh gene delivery mitigated irradiation-induced hyposalivation in a miniature pig model. Theranostics. 2018;8(16):4321-4331.
710. Yang W, Tian ZK, Yang HX, et al. Fisetin improves lead-induced neuroinflammation,

apoptosis and synaptic dysfunction in mice associated with the AMPK/SIRT1 and autophagy pathway. Food Chem Toxicol. 2019 Dec;134:110824.
711. Sun S, Han X, Li X, et al. MicroRNA-212-5p Prevents Dopaminergic Neuron Death by Inhibiting SIRT2 in MPTP-Induced Mouse Model of Parkinson's Disease. Front Mol Neurosci. 2018;11:381.
712. Li S, Dou X, Ning H, et al. Sirtuin 3 acts as a negative regulator of autophagy dictating hepatocyte susceptibility to lipotoxicity. Hepatology. 2017 Sep;66(3):936-952.
713. Shin HJ, Kim H, Oh S, et al. AMPK-SKP2-CARM1 signalling cascade in transcriptional regulation of autophagy. Nature. 2016 Jun 23;534(7608):553-7.
714. Geiser J, De Lisle RC, Andrews GK. The zinc transporter Zip5 (Slc39a5) regulates intestinal zinc excretion and protects the pancreas against zinc toxicity. PLoS One. 2013;8(11):e82149.
715. Chen R, Zou Y, Mao D, et al. The general amino acid control pathway regulates mTOR and autophagy during serum/glutamine starvation. J Cell Biol. 2014 Jul 21;206(2):173-82.
716. Liu N, Feng J, Lu X, et al. Isorhamnetin Inhibits Liver Fibrosis by Reducing Autophagy and Inhibiting Extracellular Matrix Formation via the TGF-beta1/Smad3 and TGF-beta1/p38 MAPK Pathways. Mediators Inflamm. 2019;2019:6175091.
717. Li N, Li M, Hong W, et al. Brg1 regulates pro-lipogenic transcription by modulating SREBP activity in hepatocytes. Biochim Biophys Acta Mol Basis Dis. 2018 Sep;1864(9 Pt B):2881-2889.
718. Rodriguez-Muela N, Parkhitko A, Grass T, et al. Blocking p62-dependent SMN degradation ameliorates spinal muscular atrophy disease phenotypes. J Clin Invest. 2018 Jul 2;128(7):3008-3023.
719. Zhang P, Guan Y, Chen J, et al. Contribution of p62/SQSTM1 to PDGF-BB-induced myofibroblast-like phenotypic transition in vascular smooth muscle cells lacking Smpd1 gene. Cell Death Dis. 2018 Nov 19;9(12):1145.
720. Cai Q, Sheng ZH. Uncovering the role of Snapin in regulating autophagy-lysosomal function. Autophagy. 2011 Apr;7(4):445-7.
721. Minakaki G, Menges S, Kittel A, et al. Autophagy inhibition promotes SNCA/alpha-synuclein release and transfer via extracellular vesicles with a hybrid autophagosome-exosome-like phenotype. Autophagy. 2018;14(1):98-119.
722. Shibuya Y, Chang CC, Huang LH, et al. Inhibiting ACAT1/SOAT1 in microglia stimulates autophagy-mediated lysosomal proteolysis and increases Abeta1-42 clearance. J Neurosci. 2014 Oct 22;34(43):14484-501.
723. Rudnick ND, Griffey CJ, Guarnieri P, et al. Distinct roles for motor neuron autophagy early and late in the SOD1(G93A) mouse model of ALS. Proc Natl Acad Sci U S A. 2017 Sep 26;114(39):E8294-e8303.
724. Mehta SL, Lin Y, Chen W, et al. Manganese superoxide dismutase deficiency exacerbates ischemic brain damage under hyperglycemic conditions by altering autophagy. Transl Stroke Res. 2011 Mar 1;2(1):42-50.
725. Mariani MM, Malm T, Lamb R, et al. Neuronally-directed effects of RXR activation in a mouse model of Alzheimer's disease. Sci Rep. 2017 Feb 16;7:42270.
726. Wang S, Xia P, Ye B, et al. Transient activation of autophagy via Sox2-mediated

suppression of mTOR is an important early step in reprogramming to pluripotency. Cell Stem Cell. 2013 Nov 7;13(5):617-25.

727. Zhou S, Lei D, Bu F, et al. MicroRNA-29b-3p Targets SPARC Gene to Protect Cardiocytes against Autophagy and Apoptosis in Hypoxic-Induced H9c2 Cells. J Cardiovasc Transl Res. 2019 Aug;12(4):358-365.

728. Shi WN, Cui SX, Song ZY, et al. Overexpression of SphK2 contributes to ATRA resistance in colon cancer through rapid degradation of cytoplasmic RXRalpha by K48/K63-linked polyubiquitination. Oncotarget. 2017 Jun 13;8(24):39605-39617.

729. Ohmuraya M, Sugano A, Hirota M, et al. Role of Intrapancreatic SPINK1/Spink3 Expression in the Development of Pancreatitis. Front Physiol. 2012;3:126.

730. Lepine S, Allegood JC, Park M, et al. Sphingosine-1-phosphate phosphohydrolase-1 regulates ER stress-induced autophagy. Cell Death Differ. 2011 Feb;18(2):350-61.

731. Feng Y, Klionsky DJ. Autophagy regulates DNA repair through SQSTM1/p62. Autophagy. 2017 Jun 3;13(6):995-996.

732. Zhou C, Ma K, Gao R, et al. Regulation of mATG9 trafficking by Src- and ULK1-mediated phosphorylation in basal and starvation-induced autophagy. Cell Res. 2017 Feb;27(2):184-201.

733. Barbero-Camps E, Roca-Agujetas V, Bartolessis I, et al. Cholesterol impairs autophagy-mediated clearance of amyloid beta while promoting its secretion. Autophagy. 2018;14(7):1129-1154.

734. Larrue C, Heydt Q, Saland E, et al. Oncogenic KIT mutations induce STAT3-dependent autophagy to support cell proliferation in acute myeloid leukemia. Oncogenesis. 2019 Jul 16;8(8):39.

735. Wilkinson DS, Jariwala JS, Anderson E, et al. Phosphorylation of LC3 by the Hippo kinases STK3/STK4 is essential for autophagy. Mol Cell. 2015 Jan 8;57(1):55-68.

736. Sha Y, Rao L, Settembre C, et al. STUB1 regulates TFEB-induced autophagy-lysosome pathway. Embo j. 2017 Sep 1;36(17):2544-2552.

737. Liu F, Nie C, Zhao N, et al. MiR-155 Alleviates Septic Lung Injury by Inducing Autophagy Via Inhibition of Transforming Growth Factor-beta-Activated Binding Protein 2. Shock. 2017 Jul;48(1):61-68.

738. Donde A, Sun M, Jeong YH, et al. Upregulation of ATG7 attenuates motor neuron dysfunction associated with depletion of TARDBP/TDP-43. Autophagy. 2019 Jul 7:1-11.

739. Feldman DE, Chen C, Punj V, et al. The TBC1D15 oncoprotein controls stem cell self-renewal through destabilization of the Numb-p53 complex. PLoS One. 2013;8(2):e57312.

740. Chalasani ML, Kumari A, Radha V, et al. E50K-OPTN-induced retinal cell death involves the Rab GTPase-activating protein, TBC1D17 mediated block in autophagy. PLoS One. 2014;9(4):e95758.

741. Dibble CC, Elis W, Menon S, et al. TBC1D7 is a third subunit of the TSC1-TSC2 complex upstream of mTORC1. Mol Cell. 2012 Aug 24;47(4):535-46.

742. Duan W, Guo M, Yi L, et al. Deletion of Tbk1 disrupts autophagy and reproduces behavioral and locomotor symptoms of FTD-ALS in mice. Aging (Albany NY). 2019 Apr 30;11(8):2457-2476.

743. Belaid A, Cerezo M, Chargui A, et al. Autophagy plays a critical role in the degradation

of active RHOA, the control of cell cytokinesis, and genomic stability. Cancer Res. 2013 Jul 15;73(14):4311-22.
744. Nuchel J, Ghatak S, Zuk AV, et al. TGFB1 is secreted through an unconventional pathway dependent on the autophagic machinery and cytoskeletal regulators. Autophagy. 2018;14(3):465-486.
745. Zhang H, McCarty N. Tampering with cancer chemoresistance by targeting the TGM2-IL6-autophagy regulatory network. Autophagy. 2017 Mar 4;13(3):627-628.
746. Huang S, Liu N, Li H, et al. TIA1 interacts with annexin A7 in regulating vascular endothelial cell autophagy. Int J Biochem Cell Biol. 2014 Dec;57:115-22.
747. Sanchez-Jimenez C, Izquierdo JM. T-cell intracellular antigen (TIA)-proteins deficiency in murine embryonic fibroblasts alters cell cycle progression and induces autophagy. PLoS One. 2013;8(9):e75127.
748. Seya T, Matsumoto M, Ebihara T, et al. Functional evolution of the TICAM-1 pathway for extrinsic RNA sensing. Immunol Rev. 2009 Jan;227(1):44-53.
749. Xie JM, Li B, Yu HP, et al. TIGAR has a dual role in cancer cell survival through regulating apoptosis and autophagy. Cancer Res. 2014 Sep 15;74(18):5127-38.
750. Baghdadi M, Yoneda A, Yamashina T, et al. TIM-4 glycoprotein-mediated degradation of dying tumor cells by autophagy leads to reduced antigen presentation and increased immune tolerance. Immunity. 2013 Dec 12;39(6):1070-81.
751. Sun K, Xu L, Jing Y, et al. Autophagy-deficient Kupffer cells promote tumorigenesis by enhancing mtROS-NF-kappaB-IL1alpha/beta-dependent inflammation and fibrosis during the preneoplastic stage of hepatocarcinogenesis. Cancer Lett. 2017 Mar 1;388:198-207.
752. Stohr R, Kappel BA, Carnevale D, et al. TIMP3 interplays with apelin to regulate cardiovascular metabolism in hypercholesterolemic mice. Mol Metab. 2015 Oct;4(10):741-52.
753. Shi CS, Kehrl JH. MyD88 and Trif target Beclin 1 to trigger autophagy in macrophages. J Biol Chem. 2008 Nov 28;283(48):33175-82.
754. Lu Z, Xie D, Chen Y, et al. TLR2 mediates autophagy through ERK signaling pathway in Mycoplasma gallisepticum-infected RAW264.7 cells. Mol Immunol. 2017 Jul;87:161-170.
755. Gao T, Zhang SP, Wang JF, et al. TLR3 contributes to persistent autophagy and heart failure in mice after myocardial infarction. J Cell Mol Med. 2018 Jan;22(1):395-408.
756. Feng Y, Cui C, Liu X, et al. Protective Role of Apocynin via Suppression of Neuronal Autophagy and TLR4/NF-kappaB Signaling Pathway in a Rat Model of Traumatic Brain Injury. Neurochem Res. 2017 Nov;42(11):3296-3309.
757. Murdoch TB, Xu W, Stempak JM, et al. Pattern recognition receptor and autophagy gene variants are associated with development of antimicrobial antibodies in Crohn's disease. Inflamm Bowel Dis. 2012 Sep;18(9):1743-8.
758. Weindel CG, Richey LJ, Bolland S, et al. B cell autophagy mediates TLR7-dependent autoimmunity and inflammation. Autophagy. 2015;11(7):1010-24.
759. Muzes G, Kiss AL, Tulassay Z, et al. Cell-free DNA-induced alteration of autophagy response and TLR9-signaling: Their relation to amelioration of DSS-colitis. Comp Immunol Microbiol Infect Dis. 2017 Jun;52:48-57.

760. Yadav RK, Lee GH, Lee HY, et al. TMBIM6 (transmembrane BAX inhibitor motif containing 6) enhances autophagy and reduces renal dysfunction in a cyclosporine A-induced nephrotoxicity model. Autophagy. 2015;11(10):1760-74.
761. Liu C, Yue R, Yang Y, et al. AIM2 inhibits autophagy and IFN-beta production during M. bovis infection. Oncotarget. 2016 Jul 26;7(30):46972-46987.
762. Chen D, Liu J, Lu L, et al. Emodin attenuates TNF-alpha-induced apoptosis and autophagy in mouse C2C12 myoblasts though the phosphorylation of Akt. Int Immunopharmacol. 2016 May;34:107-113.
763. Ha JY, Kim JS, Kang YH, et al. Tnfaip8l1/Oxi-beta binds to FBXW5, increasing autophagy through activation of TSC2 in a Parkinson's disease model. J Neurochem. 2013 Dec 26.
764. Shin GC, Kang HS, Lee AR, et al. Hepatitis B virus-triggered autophagy targets TNFRSF10B/death receptor 5 for degradation to limit TNFSF10/TRAIL response. Autophagy. 2016 Dec;12(12):2451-2466.
765. Yang M, Liu L, Xie M, et al. Poly-ADP-ribosylation of HMGB1 regulates TNFSF10/TRAIL resistance through autophagy. Autophagy. 2015;11(2):214-24.
766. Nollet M, Santucci-Darmanin S, Breuil V, et al. Autophagy in osteoblasts is involved in mineralization and bone homeostasis. Autophagy. 2014;10(11):1965-77.
767. Xu T, Yan W, Wu Q, et al. MiR-326 Inhibits Inflammation and Promotes Autophagy in Silica-Induced Pulmonary Fibrosis through Targeting TNFSF14 and PTBP1. Chem Res Toxicol. 2019 Nov 18;32(11):2192-2203.
768. Mauro C, Pacifico F, Lavorgna A, et al. ABIN-1 binds to NEMO/IKKgamma and co-operates with A20 in inhibiting NF-kappaB. J Biol Chem. 2006 Jul 7;281(27):18482-8.
769. Douarre C, Sourbier C, Dalla Rosa I, et al. Mitochondrial topoisomerase I is critical for mitochondrial integrity and cellular energy metabolism. PLoS One. 2012;7(7):e41094.
770. Pereira GJ, Antonioli M, Hirata H, et al. Glutamate induces autophagy via the two-pore channels in neural cells. Oncotarget. 2017 Feb 21;8(8):12730-12740.
771. Liu X, Cao H, Li J, et al. Autophagy induced by DAMPs facilitates the inflammation response in lungs undergoing ischemia-reperfusion injury through promoting TRAF6 ubiquitination. Cell Death Differ. 2017 Apr;24(4):683-693.
772. Xu X, Huang E, Tai Y, et al. Nupr1 Modulates Methamphetamine-Induced Dopaminergic Neuronal Apoptosis and Autophagy through CHOP-Trib3-Mediated Endoplasmic Reticulum Stress Signaling Pathway. Front Mol Neurosci. 2017;10:203.
773. Pizon V, Rybina S, Gerbal F, et al. MURF2B, a novel LC3-binding protein, participates with MURF2A in the switch between autophagy and ubiquitin proteasome system during differentiation of C2C12 muscle cells. PLoS One. 2013;8(10):e76140.
774. Yang A, Rajeshkumar NV, Wang X, et al. Autophagy is critical for pancreatic tumor growth and progression in tumors with p53 alterations. Cancer Discov. 2014 Aug;4(8):905-13.
775. Saadi H, Seillier M, Carrier A. The stress protein TP53INP1 plays a tumor suppressive role by regulating metabolic homeostasis. Biochimie. 2015 Nov;118:44-50.
776. Ahmed M, Hwang JS, Lai TH, et al. Co-Expression Network Analysis of AMPK and Autophagy Gene Products during Adipocyte Differentiation. Int J Mol Sci. 2018 Jun 19;19(6).

777. Kenzelmann Broz D, Attardi LD. TRP53 activates a global autophagy program to promote tumor suppression. Autophagy. 2013 Sep;9(9):1440-2.
778. Shengyou Y, Li Y. The effects of siRNA-silenced TRPC6 on podocyte autophagy and apoptosis induced by AngII. J Renin Angiotensin Aldosterone Syst. 2015 Dec;16(4):1266-73.
779. Amantini C, Farfariello V, Cardinali C, et al. The TRPV1 ion channel regulates thymocyte differentiation by modulating autophagy and proteasome activity. Oncotarget. 2017 Oct 31;8(53):90766-90780.
780. Di Nardo A, Wertz MH, Kwiatkowski E, et al. Neuronal Tsc1/2 complex controls autophagy through AMPK-dependent regulation of ULK1. Hum Mol Genet. 2014 Jul 15;23(14):3865-74.
781. Morris CR, Stanton MJ, Manthey KC, et al. A knockout of the Tsg101 gene leads to decreased expression of ErbB receptor tyrosine kinases and induction of autophagy prior to cell death. PLoS One. 2012;7(3):e34308.
782. Kim S, Kim N, Park S, et al. Tanycytic TSPO inhibition induces lipophagy to regulate lipid metabolism and improve energy balance. Autophagy. 2019 Aug 30:1-21.
783. Teixeira CA, Almeida Mdo R, Saraiva MJ. Impairment of autophagy by TTR V30M aggregates: in vivo reversal by TUDCA and curcumin. Clin Sci (Lond). 2016 Sep 1;130(18):1665-75.
784. Lei Y, Wen H, Yu Y, et al. The mitochondrial proteins NLRX1 and TUFM form a complex that regulates type I interferon and autophagy. Immunity. 2012 Jun 29;36(6):933-46.
785. Huang C, Zhang Y, Kelly DJ, et al. Thioredoxin interacting protein (TXNIP) regulates tubular autophagy and mitophagy in diabetic nephropathy through the mTOR signaling pathway. Sci Rep. 2016 Jul 6;6:29196.
786. Tasaki T, Kim ST, Zakrzewska A, et al. UBR box N-recognin-4 (UBR4), an N-recognin of the N-end rule pathway, and its role in yolk sac vascular development and autophagy. Proc Natl Acad Sci U S A. 2013 Mar 5;110(10):3800-5.
787. Zhang D, Han S, Wang S, et al. cPKCgamma-mediated down-regulation of UCHL1 alleviates ischaemic neuronal injuries by decreasing autophagy via ERK-mTOR pathway. J Cell Mol Med. 2017 Dec;21(12):3641-3657.
788. Pan P, Zhang H, Su L, et al. Melatonin Balance the Autophagy and Apoptosis by Regulating UCP2 in the LPS-Induced Cardiomyopathy. Molecules. 2018 Mar 16;23(3).
789. Schulze U, Vollenbroker B, Braun DA, et al. The Vac14-interaction network is linked to regulators of the endolysosomal and autophagic pathway. Mol Cell Proteomics. 2014 Jun;13(6):1397-411.
790. Ju JS, Fuentealba RA, Miller SE, et al. Valosin-containing protein (VCP) is required for autophagy and is disrupted in VCP disease. J Cell Biol. 2009 Dec 14;187(6):875-88.
791. Tavera-Mendoza LE, Westerling T, Libby E, et al. Vitamin D receptor regulates autophagy in the normal mammary gland and in luminal breast cancer cells. Proc Natl Acad Sci U S A. 2017 Mar 14;114(11):E2186-e2194.
792. Li Q, Kan X, Yin J, et al. Chamaejasmine B Induces the Anergy of Vascular Endothelial Cells to VEGFA Pro-angiogenic Signal by Autophagic Regulation of VEGFR2 in Breast Cancer. Front Pharmacol. 2017;8:963.
793. Bastola P, Stratton Y, Kellner E, et al. Folliculin contributes to VHL tumor suppressing

activity in renal cancer through regulation of autophagy. PLoS One. 2013;8(7):e70030.
794. Maiztegui B, Boggio V, Roman CL, et al. VMP1-related autophagy induced by a fructose-rich diet in beta-cells: its prevention by incretins. Clin Sci (Lond). 2017 Apr 25;131(8):673-687.
795. Peng C, Ye J, Yan S, et al. Ablation of vacuole protein sorting 18 (Vps18) gene leads to neurodegeneration and impaired neuronal migration by disrupting multiple vesicle transport pathways to lysosomes. J Biol Chem. 2012 Sep 21;287(39):32861-73.
796. Zhen Y, Li W. Impairment of autophagosome-lysosome fusion in the buff mutant mice with the VPS33A(D251E) mutation. Autophagy. 2015;11(9):1608-22.
797. Dragich JM, Kuwajima T, Hirose-Ikeda M, et al. Autophagy linked FYVE (Alfy/WDFY3) is required for establishing neuronal connectivity in the mammalian brain. Elife. 2016 Sep 20;5.
798. Gong J, Belinsky G, Sagheer U, et al. Pigment Epithelium-derived Factor (PEDF) Blocks Wnt3a Protein-induced Autophagy in Pancreatic Intraepithelial Neoplasms. J Biol Chem. 2016 Oct 14;291(42):22074-22085.
799. Jati S, Kundu S, Chakraborty A, et al. Wnt5A Signaling Promotes Defense Against Bacterial Pathogens by Activating a Host Autophagy Circuit. Front Immunol. 2018;9:679.
800. Chiang MF, Chou PY, Wang WJ, et al. Tumor Suppressor WWOX and p53 Alterations and Drug Resistance in Glioblastomas. Front Oncol. 2013;3:43.
801. Tian PG, Jiang ZX, Li JH, et al. Spliced XBP1 promotes macrophage survival and autophagy by interacting with Beclin-1. Biochem Biophys Res Commun. 2015 Aug 7;463(4):518-23.
802. Huang X, Wu Z, Mei Y, et al. XIAP inhibits autophagy via XIAP-Mdm2-p53 signalling. Embo j. 2013 Aug 14;32(16):2204-16.
803. Scheibye-Knudsen M, Fang EF, Croteau DL, et al. Contribution of defective mitophagy to the neurodegeneration in DNA repair-deficient disorders. Autophagy. 2014 Aug;10(8):1468-9.
804. Bai H, Inoue J, Kawano T, et al. A transcriptional variant of the LC3A gene is involved in autophagy and frequently inactivated in human cancers. Oncogene. 2012 Oct 4;31(40):4397-408.
805. Pan H, Yan Y, Liu C, et al. The role of ZKSCAN3 in the transcriptional regulation of autophagy. Autophagy. 2017 Jul 3;13(7):1235-1238.
806. Matsui T, Fukuda M. Rab12 regulates mTORC1 activity and autophagy through controlling the degradation of amino-acid transporter PAT4. EMBO Rep 2013 May; 14:450-7.
807. Lin M, Chang Y, Xie F, et al. ASPP2 Inhibits the Profibrotic Effects of Transforming Growth Factor-beta1 in Hepatic Stellate Cells by Reducing Autophagy. Dig Dis Sci 2018 Jan; 63:146-54.
808. Maejima Y, Ito Y, Tamura N, et al. Blood coagulation factor Xa promotes the progression of atherosclerosis by enhancing inflammasome formation as a consequence of PAR2-mediated autophagy inhibition. Eur Heart J 2017 Aug; 38:134-.
809. Nishiyama A, Xhang YF, Nakano D. Sglt2 Inhibitor Prevents Renal Fibrosis after Renal Ischemia/Reperfusion Injury in Mice. Journal of Hypertension 2018 Oct; 36:E22-E; doi: 10.1097/01.hjh.0000548076.64133.df.

810. Tekirdag, K.A., Ozturk, et al. Alteration in Autophagic-lysosomal Potential During Aging and Neurological Diseases: The microRNA Perspective. Curr Pathobiol Rep 2013 Oct 1; 1: 247.
